# Bacterial genome adaptation to niches: Divergence of the potential virulence genes in three *Burkholderia *species of different survival strategies

**DOI:** 10.1186/1471-2164-6-174

**Published:** 2005-12-07

**Authors:** H Stanley Kim, Mark A Schell, Yan Yu, Ricky L Ulrich, Saul H Sarria, William C Nierman, David DeShazer

**Affiliations:** 1 The Institute for Genomic Research, 9712 Medical Center Drive, Rockville, MD 20850, USA; 2 Department of microbiology and plant pathology, University of Georgia, Athens, GA 30602, USA; 3 US Army Medical Research Institute of Infectious Diseases (USAMRIID), Fort Detrick, MD, 21702 USA

## Abstract

**Background:**

Two closely related species *Burkholderia mallei *(Bm) and *Burkholderia pseudomallei *(Bp) are serious human health hazards and are potential bio-warfare agents, whereas another closely related species *Burkholderia thailandensis *(Bt) is a non-pathogenic saprophyte. To investigate the genomic factors resulting in such a dramatic difference, we first identified the Bm genes responsive to the mouse environment, and then examined the divergence of these genes in Bp and Bt.

**Results:**

The genes down-expressed, which largely encode cell growth-related proteins, are conserved well in all three species, whereas those up-expressed, which include potential virulence genes, are less well conserved or absent notably in Bt. However, a substantial number of up-expressed genes is still conserved in Bt. Bm and Bp further diverged from each other in a small number of genes resulting from unit number changes in simple sequence repeats (ssr) in the homologs.

**Conclusion:**

Our data suggest that divergent evolution of a small set of genes, rather than acquisition or loss of pathogenic islands, is associated with the development of different life styles in these bacteria of similar genomic contents. Further divergence between Bm and Bp mediated by ssr changes may reflect different adaptive processes of Bm and Bp fine-tuning into their host environments.

## Background

*Burkholderia mallei *(Bm) and *Burkholderia pseudomallei *(Bp) are the causative agents of glanders and melioidosis, respectively, and are serious human health hazards mostly in Southeast Asia, Northern Australia, South and Central America, and the Middle East [1-4]. Melioidosis is characterized by severe pulmonary distress with frequent progression to septicemia and death [1,2]. Glanders is similar in symptoms to melioidosis, however, infections mostly occur in equines and is only occasionally transmitted to humans [3,5]. The two bacteria are listed as category B potential biowarfare agents by the US Centers for Disease Control and Prevention (CDC) due to their high infectivity via the aerosol route, difficulty in diagnosis, painful incapacitating disease symptoms, a required complex therapeutic antibiotic regimen, high mortality, and the historical use of Bm as a biological weapon [6-10]. Multilocus Sequence Typing (MLST) suggests that Bm may have evolved from a single strain of Bp [11].

Recent completion of genome sequencing of *B. mallei *ATCC 23344 and *B. pseudomallei *K96243 have dramatically facilitated research on these pathogens. Both contain two chromosomes and an unusually high number of Simple Sequence Repeats (SSRs) [12,13]. Frameshift, missense, deletion, and insertion mutations due to the differences in SSR repeat numbers were noted between Bm and Bp in some genes [12]. Bm also has a high number of insertion sequences (ISs) dispersed throughout the genome, that resulted in shuffling and deletion of chromosomal fragments.

*Burkholderia thailandensis *(Bt) is closely related to Bm and Bp, but is nonpathogenic to higher animals and humans [14,15]. Like Bp, it is a natural inhabitant of the tropical soil environment. Bm, however, has never been isolated from a non-animal-host environment, suggesting that it is an obligate animal parasite [16,17]. Part of the reason for the inability of Bm to thrive in the non-host environment appears to be its fragility to extreme conditions, including dehydration and heat [17,18]. Although it has retained most of the genes needed for chemotaxis and motility, Bm lacks functional flagella due to mutations in a few key genes [12]. In contrast, Bp and Bt have functional flagella which may well be essential for survival in the soil environment. The genetic defects in Bm that restrict its growth outside the host likely largely resulted from the IS-mediated genome reduction process that accompanied its adaptation to life as an obligate mammalian pathogen [12].

In this study, we investigated the genomic factors that drove Bm, Bp and Bt into lives of different survival strategies. We first profiled gene expression of Bm that had colonized mouse liver and spleen compared to that in cultures, and then examined the divergence of the genes up- or down-expressed *in vivo *across the three species. We show that genes down-expressed in animal are highly conserved in all three, whereas those up-expressed, which are more likely involved in *in vivo *survival, are well conserved between Bm and Bp but less well in Bt. These findings suggest that divergent evolution of a selected set of genes played a role in the development of Bm and Bp as effective pathogens and Bt as a non-pathogenic soil saprophyte. Understanding the function of the proteins encoded by these diverged genes may prove essential to a detailed appreciation of Bm-Bp-specific virulence and provide targets for therapeutics, while the *in vivo *expression data set as a whole provides a glimpse of the overall approach to life employed by these pathogens within the animal host.

## Results

### Sequencing of the Bt genome and comparison among the three species

For comparative genomic analysis with pathogenic Bm and Bp, we produced finished sequence of the closely related nonpathogenic soil bacterium Bt E264 [15]. Bt showed the genomic organization matching to that of Bm and Bp, which contain two chromosomes without plasmids. Chromosomes 1 and 2 contain 3,809,201 bp and 2,914,771 bp, respectively, which are slightly larger than those in Bm [12], but smaller than the Bp counterparts [13]. Automated annotation predicted a total of 5,645 (3,282 in chromosome 1 and 2,363 from chromosome 2) protein-coding genes.

To obtain whole genome comparisons among Bt, Bp, and Bm, we conducted TBLASTN searches with the manually annotated Bm proteome [12] to the nucleotide sequences of Bp and Bt. Using TBLASTN eliminates discrepancies between the manual annotation results between TIGR (Bm) and the Sanger Institute (Bp) and by less reliable auto-annotation of Bt. Amino acid identities of predicted orthologs among the three species were very high; as much as 96.3% and 72.1% of the Bm proteome matched to that of Bp and of Bt, respectively, at least at the level of 80% identity over 80% of alignment length. These proteins of Bm and Bp had high mean values of identity (98.8%) and length match (99.7%). In contrast, Bm and Bt were more divergent, but also showed high homology (mean identity of 94.0% and mean length match of 99.5%).

That Bm, Bp, and Bt have the same genome structure with two chromosomes and have high nucleotide identity at the DNA level indicate that they diverged very recently, most likely between Bt and the Bm-Bp common ancestor followed by the second divergence between Bm and Bp. We noted a number of events of shuffling and deletion or insertion of large DNA segments relative to one another among all three organisms. Such genome modifications were more prevalent between Bp and Bm than between Bp and Bt (see Additional file 1). We previously showed that a high number of IS elements present in Bm are responsible for the extensive shuffling and deletion of the genome relative to the Bp genome [12]. The number of IS elements in Bt and Bp are lower (171 in Bm, 48 in Bp, and 102 in Bt), and most of the syntenic fragments in Bt and Bp are not flanked by IS elements as is the case for Bm, indicating a much reduced role of the IS elements in genome modifications of Bp and Bt.

### *In vivo *expression of Bm genes in the mouse spleen and liver

**Figure 1:**
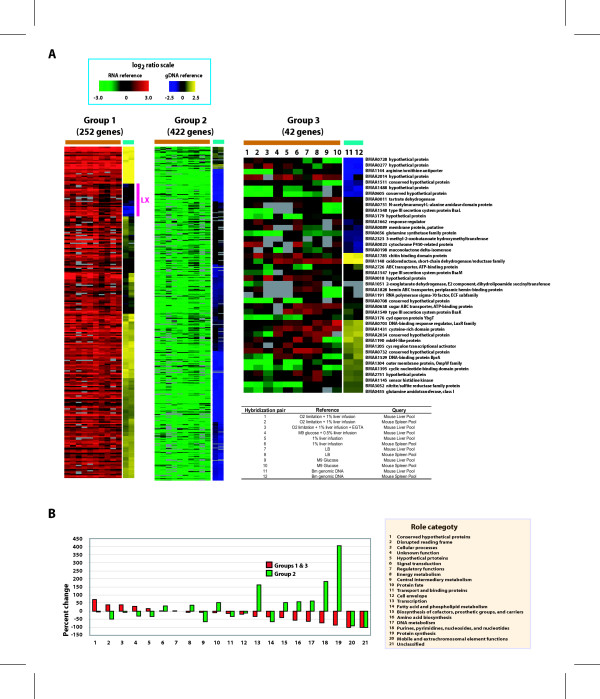
Expression of *B. mallei *(Bm) genes *in vivo *profiled in contrast to that in cultures and to Bm genomic DNA. A. Heat map representation of gene expression profiles of the three distinct groups and a description of each hybridization pair. The two bars in the box at the top indicate colors corresponding to the range of the observed expression ratios on a log_2 _scale for the data generated with two different references. B. Over- or under-representation of role categories in the *in vivo*-responsive genes. Percent changes in the proportion of each role category relative to its original proportion in the genome are shown.

We infected three mice with Bm and profiled Bm gene expression in cells recovered from livers and spleens three days after infection using microarrays (Fig. 1A). We found 716 genes that were significantly up- or down-expressed *in vivo *(i.e. in the liver or spleen) compared to six *in vitro *culture conditions in at least one of the 10 different hybridizations based on the 95% confidence level (Additional file 3; see Methods). Despite the differences in the reference cultures in which one may mimic the host environment closer than another, most of these *in vivo*-responsive genes fell into two distinct groups: 1) those (252) up-expressed relative to *in vitro *culture conditions in all 10 hybridizations and 2) those (422) down-expressed relative to *in vitro *cultures in all 10 hybridizations. A small number of genes (42) were up- or down expressed depending on the compared culture conditions. While these results simply show that there is a large difference in the gene expression environments between the animal body and the cultures, the data of consistently up- or down-expressed genes support the integrity of the microarray data themselves. Using quantitative real-time reverse transcription-polymerase chain reaction (qRT-PCR), we have confirmed the microarray data for thirteen randomly picked genes for one comparison (1% liver infusion media vs. mouse spleen; Additional file 2).

By using Bm genomic DNA as reference for the *in vivo *RNA microarray analysis, the relative levels of the mRNAs to each other were obtained. When compared to the genomic DNA reference, expression levels of most genes in group 1 were high relative to the normalized mid-level of the reference, while most of those in group 2 were low (Fig. 1A, Additional file 3). Genes in group 3 showed both high and low levels of expression. There was little difference in individual gene expression levels between the liver and the spleen environment.

Among group 1 (up-expressed) genes were a set of 25 with low expression levels, i.e. lower than the genomic DNA reference (denoted as LX in Fig. 1A and Additional file 3). Nearly all are on chromosome 2, and their weak expression, unlike most other group 1 genes, makes them unique and interesting. These include the genes coding for a serine protease, a fusaric acid resistance protein, and a drug resistance transporter. However, others encode proteins with no predictable function or identified domains (i.e., hypothetical or conserved hypothetical proteins), not allowing speculation on their roles in pathogenesis.

Nearly 80% of group 2 (down-expressed) genes are found on chromosome 1 and are homologous to housekeeping genes coding for relatively well-characterized proteins involved in cell replication [e.g. DNA polymerase III subunits, DNA gyrase, DnaA, glucose inhibited cell division protein, FtsZ, >30 ribosomal proteins, elongation factors Ts and Tu, and several t-RNA synthetases (for *arg*, *cys*, *leu *and *glu*)]. Genes coding for critical enzymes of central carbohydrate energy metabolism are also significantly down-expressed compared to the *in vitro *cultures. These include those of the Embden-Meyerhoff (glycolytic) pathway (e.g., fructose bis-phosphatase, pyruvate dehydrogenase, and acetyl CoA synthetase) and the TCA cycle (e.g., citrate synthase, oxoglutarate dehydrogenase, and isocitrate dehydrogenase). Genes coding for ATPase subunits are also strongly down-expressed. Overall, there is a 400% enrichment of genes encoding protein synthesis functions and a nearly 200% enrichment of genes encoding biosynthesis of nucleic acid building blocks and transcription in group 2 compared to the whole genome (Fig. 1B). Taken together, this data suggests that bacterial growth and multiplication rate in livers and spleens are dramatically reduced relative to *in vitro *culture conditions.

**Figure 2:**
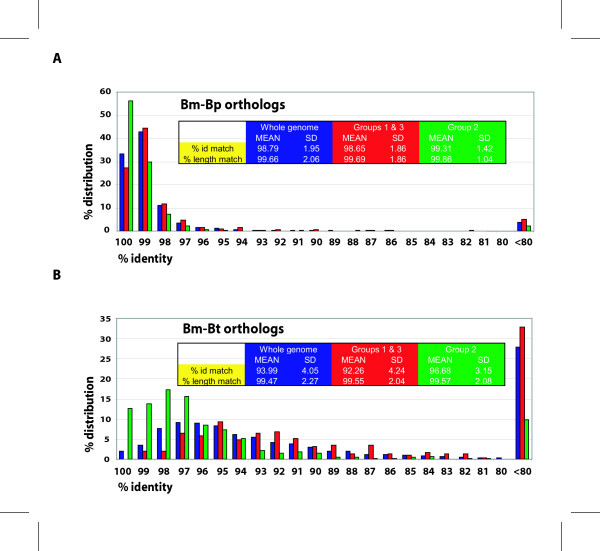
Comparative genomic analyses of *B. mallei*, *B. pseudomallei*, and *B. thailandensis *with the whole genomes and with *in vivo*-responsive genes. The data are based on the TBLASTN scores generated with Bm proteome and nucleotide sequences of Bp or Bt. A. Comparison between *B. mallei *and *B. pseudomallei*. Genes of both % identity and % length equal to or above 80 and all below the cut off as a group are shown with % distribution. Comparison statistics (Mean and Standard Deviation) are also shown in the table present inside the graph. The blue, red, and green colors are used in the table to match the bars in the graph that they have statistics for. B. Comparison between *B. mallei *and *B. thailandensis*.

When these group 2 genes from Bm were compared with their orthologs in Bp and Bt, the mean identities were similar or slightly higher than the genome means (99.3% vs. 98.8% for the Bm-Bp comparison; 96.7% vs. 94.0% for the Bm-Bt comparison) (Fig. 2AB). However, there were significant increases in the genes of top % identity (see % distribution increases for these genes in Fig. 2AB). This is consistent with the fact that these genes are mostly involved in the basic housekeeping functions in the cell, such as protein synthesis, transcription, biosynthesis of nucleic acid building blocks, amino acid biosynthesis, etc.

In contrast, many of group 1 genes are found on chromosome 2 (63%), and this group is enriched in genes that have no predictable function or identifiable motifs (i.e. hypothetical proteins and conserved hypothetical proteins), or have frame-shift mutations (Fig. 1B). The preponderance of genes of unknown functions in this group suggests that many aspects of the molecular basis of pathogenesis and *in vivo *survival remain to be elucidated.

Genes that belong to the category of cellular process also are enriched in group 1, and they appear to include many of those encoding functions needed to infect and survive in the host. For example, genes that may be involved in iron sequestration in free-iron-limited host cells, such as those encoding a cation ABC transporter, an iron compound ABC transporter, a hemin ABC transporter, and a TonB protein, are included. There are also genes that may encode detoxification or toxin-resistance functions, e.g., EmrB/QacA family drug resistance transporters, a hydrophobe/amphiphile efflux family protein, an RND family efflux transporter, a fosmidomycin resistance protein, and a NodT family RND efflux system. Genes encoding glutathione-independent formaldehyde dehydrogenase and formate dehydrogenase, which may be involved in formaldehyde detoxification, are also present. A potential source of formaldehyde *in vivo *is unclear, although one possibility is choline metabolism. Genes encoding enzymes involved in anaerobic respiration, such as nitrate reductase, outer membrane nitrite reductase, and formate dyhydrogenase, are present. From this observation, we suspect that anaerobic metabolism may be important in mouse livers and spleens. Others include, a potential virulence gene coding for a HlyB family hemolysin activator protein which may be involved in exporting hemolysin(s) and genes within the type III secretion system (TTSS) loci.

Group 3 genes show varied levels of expression in response to different culture conditions, indicating that some culture conditions may mimic or generate the transcription activating signals present in mouse spleen and liver. This variable expression data provide clues to regulatory stimuli and perhaps some insight into the roles of these genes.

When the orthologs of groups 1 and 3 together as up-expressed genes were compared between Bm and Bp, the mean identity of the orthologs was similar to the genome average (98.7 vs. 98.8) and the % distribution of the genes of different identity levels also was similar (Fig. 2A). However, when Bt was compared with Bm, the mean identity declined slightly from the genome average of 94.0 to 92.3. More importantly, the % distribution for the top % identities (i.e. 100 through 96) was significantly decreased, while that for the lower % identities increased. This indicates that these genes in Bt and Bm have diverged more than the rest of the genome, while Bp and Bm do not show such differential divergence.

### Divergence of the *in vivo*-responsive genes in the three species

**Figure 3:**
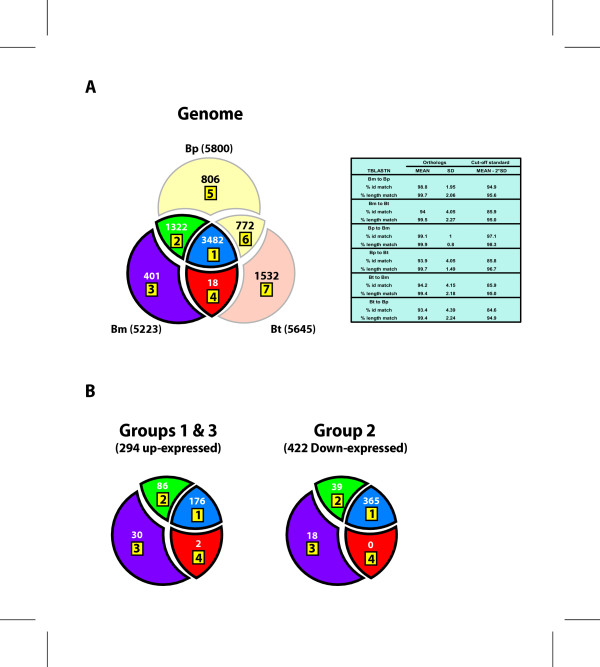
Comparison among *B. mallei*, *B. pseudomallei*, and *B. thailandensis *with a divergence cut-off of two-times of standard deviation from the mean values of identity and length match. Venn diagrams show the numbers of genes common or diverged or unique to each genome. Genes in the diagrams are shown in Additional files 4, 5 and 6. A. Comparisons generated based on the TBLASTN scores with the proteome of one genome to the nucleotide sequences of other genomes. Segments labeled 1, 2, 3, 4 are based on the Bm proteome, while segments 5 and 6 are based on the Bp proteome and segment 7 is based on the Bt proteome. B. Comparisons with *in vivo*-responsive group 1 and 3 genes and group 2 genes (see Figure 1).

To distinguish diverged genes from those conserved between two compared genomes, we defined an arbitrary standard for significant divergence; i.e. when the % identity or the % length of the match are lower than the genome-wide means by more than two standard deviations (SDs). While a universal standard that applies to all genes equally does not exist, using this standard we tentatively organized the genes into sub-groups of relative divergence (Fig. 3A; a complete list is given in Additional file 4). When only the *in vivo *expression data was used, 60.5% (178 genes) of the group 1 and 3 genes (*in vivo*-up-expressed) and 86.5% (365 genes) of the group 2 (down-expressed) genes are conserved between Bm and Bt (Fig. 3B). In contrast, 89.4% (262 genes) of the genes in the groups 1 and 3 and 95.7% (404 genes) of the genes in the group 2 are conserved between Bm and Bp (Fig. 3B; Additional file 5).

The data indicate that Bt also shares a number of the up-expressed genes (178), while there also are many that are diverged significantly or absent (116). The 178 up-expressed genes include a number of genes that may be involved in survival in the host (e.g., TTSS-2 genes, iron uptake genes, anaerobic respiration genes, LPS biosynthesis genes, degradative enzymes, etc.).

The 86 up-expressed genes that are conserved in Bm and Bp but not as well in Bt are of special interest because they may contain the genes that contribute to the distinction of Bp and Bm as animal pathogens from non-pathogenic Bt. Genes in this group include those encoding putative detoxification or resistance function for toxins (e.g., BMA1038 putative penicillin amidase and BMA0952 NodT family RND efflux system), secondary metabolite biosynthesis (e.g., BMA1123 peptide synthetase and BMAA1202 polyketide synthase), some TTSS genes (e.g., BMAA1617 putative *hrp *protein and BMAA1619 hypothetical protein), and cell envelope synthesis genes (e.g., BMAA0751 N-acetylmuramoyl-L-alanine amidase domain protein, BMAA1498 putative O-antigen acetylase, BMAA1986 ADP-heptose-LPS heptosyltransferase II, BMAA1987 glycosyl transferase).

There are 30 up-expressed genes that appear to have diverged in Bm even relative to their Bp ortholog. Twenty one of these have frame-shift mutations relative to their counterparts in Bp resulting in rather dramatic changes in the proteins that they code for, while eight have only subtle in-frame mutations and one is completely absent in Bp (Additional file 6). At least some of these possibly code for functional Bm-unique proteins. Seven of the eight genes with in-frame mutations do not have assigned predicted functions, but one (BMA0605) is weakly related to hemerythrin-coding gene in *Ralstonia solanacearm*, the product of which is involved in oxygen transfer and/or storage. One of the eight (BMAA1526) is related to the *bapA *gene in *Borrelia burgdorferi*. The *bapA *gene present in many *B. burgdorferi *isolates is linked to the virulence-involved *erp *locus and was shown to be co-expressed with the locus [19]. While the exact function is unknown, it is suspected that *bapA *may also perform an important function for *B. furgdorferi *virulence, based on its genetic pairing with the *erp *genes and immunological evidence [20]. BMAA0610, which codes for di-haem cytochrome C peroxidase family protein and is only present in Bm, is related to enzymes in *Pseudomonas aeruginosa *and *Neisseria gonorrhoeae *that are located in the periplasm where their likely function is to provide protection against toxic peroxides [21,22]. In *N. gonorrhoeae*, the gene was shown to be induced during oxygen-limiting growth.

## Discussion

Our analysis comparing the Bm genes up- or down-expressed *in vivo *compared to cultures across the three genomes of Bm, Bp, and Bt revealed that most of these genes are highly conserved in the three species. Only a fraction of them, mostly those from up-expressed genes which include potential *in vivo*-survival genes, have gone through measurable divergence while adapting to their specific niches. Genes down-expressed *in vivo *mostly encode cell growth functions, and this suggests that the growth rate of the Bm in mouse organs after two days of infection is significantly slower than that in the late log phase cultures (see Materials and Methods). Further, high conservation of these genes among the three species suggests that these so called house keeping genes do not have a significant role during niche-adaptation processes. Among the *in vivo*-up-expressed genes, those diverged in Bt but conserved in Bm and Bp may include the common set that have contributed to the development of Bm and Bp to animal pathogens, while those not conserved as well even in Bp represent the genes involved in fine-tuning of Bm to its specific equine niches. It is of note that Bm preferentially establishes a chronic infection in the equines, while in other mammalian hosts it causes an acute infection and rapid death of the animals.

**Figure 4:**
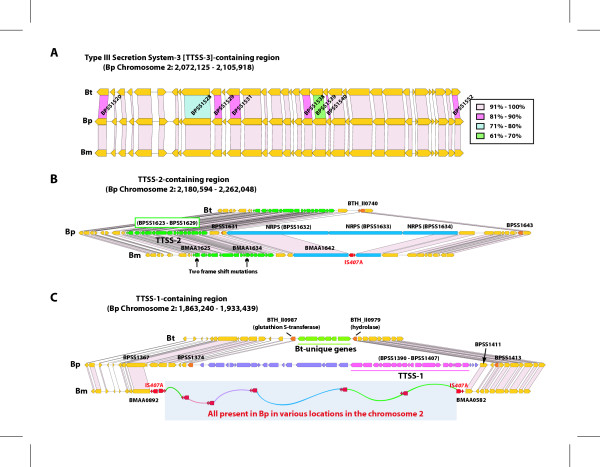
Comparison of the Type III Secretion Systems (TTSSs) and the surrounding regions in *B. mallei *(Bm)*, B. pseudomallei *(Bp), and *B. thailandensis *(Bt). The orthologous genes in the three species are denoted with connecting lines. A. Comparison of TTSS-3 locus among the three species. The % amino acid identity was determined using TBLASTN from Bp proteins, and is color coded accordingly. B. Comparison of TTSS-2 and its vicinity. Large deletions in the genes coding for non ribosomal peptide synthases (NRPSs) in Bm and Bt are shown. Two frame shift mutations in the two genes in the TTSS-2 of Bm also are shown. C. Comparison of the regions around TTSS-1. The fragment containing TTSS-1 and the surrounding genes that are only present in Bp, and the replacement fragments of this in Bt and Bm are shown.

There are 86 up-expressed genes that are conserved in Bm and Bp but not as well or absent in Bt. Genes in this group include those possibly encoding detoxification or resistance function for toxins, secondary metabolite biosynthesis, cell envelope synthesis genes, etc. Also included in this group are the genes that belong to the animal pathogen-type type III secretion system (TTSS-3) cluster, which were actually shown essential for virulence in Bp and Bm in hamster [23,24]. Intriguingly, all three species, including Bt, contain the full set of gene members of the cluster (Fig. 4A). However, a few Bt genes are significantly divergent from the orthologs of Bm and Bp, which are essentially identical to each other. Whether the divergence of the genes in this cluster has implications in the avirulence nature of Bt remains to be investigated.

Although most Bm genes diverged from the Bp orthologs appear to be degenerated, there are a few that may code for Bm-specific functional proteins (see Additional file 6). Many of them do not have assigned predicted functions, but those with the annotated functions are either suspected to be involved in pathogenesis (i.e. BMAA1526 *bapA*) or associated with the survival under low-oxygen conditions (i.e. BMA0605 hemerythrin-coding gene), which are characteristic of the host environment. It is intriguing that di-haem cytochrome C peroxidase (BMAA0610), which is present only in Bm, also is associated with the oxygen-limited conditions. Considering that both BapA and di-haem cytochrome C peroxidase are extra-cytoplasmic proteins, it would be interesting to investigate whether modifications to these proteins may be examples of antigenic variation in Bm, which is widely observed in cell surface virulence factor-coding genes in various pathogenic bacteria to avoid detection by the host immune response [25,26].

The three species, Bm, Bp and Bt, represent three states of ecological niche adaptation of *Burkholderia*: 1) obligate pathogen, 2) opportunistic pathogen, and 3) saprophyte, respectively. Our comparative genomic analyses using Bm *in vivo*-responsive genes focused on studying the divergence of the core genes involved in survival in the animal host. On the other hand, there are genes that are not present in Bm but play important roles in specific phenotypic features in Bp and Bt, such as environmental survival of Bp and Bt or broader host range of Bp. There are 806 Bp-unique genes (Fig 3A). In the TTSS plant pathogen type locus (TTSS-2) [24] of Bp, there are three non-ribosomal peptide synthase (NRPS) genes following the TTSS-2 locus. The organization of the whole region in Bp suggests a model in which the three NRPSs produce a toxin or toxins temporally coordinated with the expression of the TTSS-2 genes. However, the NRPS genes are completely or partially missing in Bt and Bm, respectively (Fig. 4B). In Bt, it appears that two genes, which flank the region containing the three NRPS genes and eight others, were internally fused deleting the intervening region. High homology between these genes appears to have contributed to this deletion mutation events via homologous recombination. In the case of Bm, the NRPS deletion appears to be mediated by two IS elements, by tranposing into the first and the third NRPS genes and deleting the intervening region by a homologous recombination event. In addition to this deletion mutation, Bm also contains two frame-shift mutations in two of the genes (BMAA1625 and BMAA1634) in TTSS-2 cluster (Fig. 4B). Both mutations occurred at the SSR sites close to the 5'end of the genes, most likely destroying the ability of the genes to produce functional proteins. These mutations in both systems (NRPS and TTSS) in Bm suggest that this region is not essential for Bm pathogenicity, but rather remains as a relic of its Bp origin.

In the case of the third type III secretion system (TTSS-1) in Bp, the genes are not present in Bm nor in Bt (Figure 4C). In Bt, there are six Bt-unique genes instead of the TTSS-1 cluster. The GC content of the genes is not different from the neighboring genes, suggesting that these genes are indigenous. In contrast, the genes of TTSS-1 in Bp have a distinctly lower GC content indicating that they may have been recently acquired. In Bm, a large region syntenic to the TTSS-1 cluster in Bp appears to have been deleted through an IS-mediated recombination. This deletion is accompanied by insertion of five IS-encompassed fragments that were collected from across chromosome 2. Although TTSS-1 is not required for glanders, involvement of the system in melioidosis can not be ruled out.

**Figure 5:**
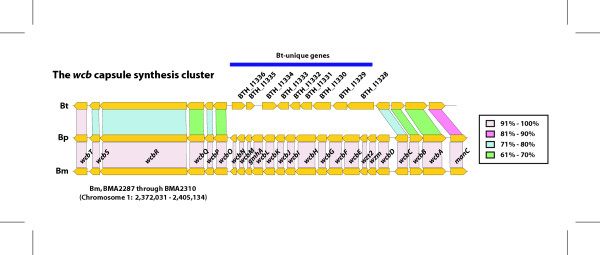
Comparison of the *wcb *capsule synthesis region in *B. mallei *(Bm), *B. pseudomallei *(Bp), and *B. thailandensis *(Bt). The orthologous genes in the three species are denoted with connecting lines. The % amino acid identity was determined using TBLASTN, and is color coded accordingly.

Besides TTSS-3, the genes in the capsule synthesis region (*wcb *genes, BMA2287 through 2310) also have been shown to be essential for the virulence in both Bm and Bp [23,27] (Fig. 5). However, these genes are not revealed in our *in vivo *expression data due to their high expression under *in vitro *conditions. While comparing these genes among the three species, we found that there are significant differences in Bt in both gene content and identity (Figure 5). In Bt, we found that nine Bt-unique predicted capsule synthesis genes are present instead of 14 Bm-Bp homologs in the middle portion of the locus and that the amino acid identity of the genes present in the two surrounding syntenic regions is much lower than the genome average. These observations suggest a different capsule is produced by Bt compared to Bm and Bp, but this has not been shown experimentally. Those 14 genes present only in Bm and Bp have distinct GC content profiles relative to the rest of the Bm and Bp genomes (data not shown), suggesting their horizontal acquisition in the Bm-Bp common ancestor perhaps resulting in higher pathogenic potential.

Bacteria that belong to *Burkholderia *keep rich repertoire of gene contents on the bigger-than-usual genome size ranging from ~4 to 9 mb. This large genetic resource with flexibility in the genome may have enabled them to adapt to a broad spectrum of environments (i.e. soil, plants, water, sea water, humans, animals, hospital environments), exhibiting enormous diversity [28]. That Bt shares many potential virulence genes with Bm and Bp suggests that it descended from a pathogenic ancestor and that it may also be a modern pathogen whose non-mammalian host remains unidentified. This point is supported by the observations that Bt can kill or paralyze nematodes when they were immersed in the Bt cultures [29,30]. The details of the relationship of genome contents, virulence, and specific niche adaptation will become clearer as more *Burkholderia *genomes of diverse life style are sequenced and available for comparative genomic analyses.

## Methods

### Sequencing

The genome of Bt (*B. thailandensis *E264) was sequenced and assembled using the random shotgun method [31]. The entire genome sequence and annotation have been deposited in the GenBank database (accession nos. CP000086 and CP000085 for chromosomes I and II, respectively).

### Coding sequence (CDS) prediction and gene identification

ORFs likely to encode proteins (CDSs) were identified by GLIMMER [32]. Identified CDSs were annotated by manual curation of the outputs of a variety of similarity searches. Searches of the predicted coding regions were performed with BLASTP, as previously described [33]. The protein-protein matches were aligned with blast_extend_repraze, a modified Smith-Waterman [34] algorithm that maximally extends regions of similarity across frameshifts. Gene identification is facilitated by searching against a database of nonredundant bacterial proteins (nraa) developed at TIGR and curated from the public archives GenBank, Genpept, PIR, and SwissProt. Searches matching entries in nraa have the corresponding role, gene common name, percent identity and similarity of match, pairwise sequence alignment, and taxonomy associated with the match assigned to the predicted coding region and stored in the database. CDSs were also analyzed with two sets of Hidden Markov Models (HMMs) constructed for a number of conserved protein families from PFAM [35] and TIGRFAM [36]. Regions of the genome without CDSs and CDSs without a database match were reevaluated by using BLASTX as the initial search, and CDSs were extrapolated from regions of alignment. Finally, each putatively identified gene was assigned to one of 113 role categories adapted from Riley [37].

### Construction of a DNA microarray of Bm

The final version of the manual annotation on the *Burkholderia mallei *genome identifies a total of 5,223 (4,954 without transposase genes from a large copy number of IS elements in the genome) coding sequences (CDSs) that are mapped to two separate chromosomes. We designed primer pairs for the 4,744 CDSs (with only one copy of transposase genes from each type of ISs) by feeding each CDS into Primer 3.0 [38]. Primers were then synthesized in 96-well microtiter plates with corresponding forward and reverse primers in alternate plates for simplified reaction set-up. These primers were used to amplify microarray probes from genomic DNA. PCR amplicons were printed in triplicate on Corning UltraGAPS™ aminosilane coated microscope slides (Corning Inc., Acton, MA) using a high precision spotting robot (Intelligent Automation Systems, MA). All the processes follow TIGR standard operating procedures [39].

### Mouse infection, bacterial RNA preparation, and labeling

Female BALB/c mice were obtained from Charles River Laboratories (National Cancer Institute, Frederick, MD) and were 6- to 8-weeks-old at the time of use. Three mice were injected intraperitoneally with 1.5 × 10^7 ^*B. mallei *ATCC 23344 (21 times the 50% lethal dose) and provided with rodent feed and water ad libitum and maintained on a 12-h light cycle. Two days postinfection the mice were euthanized in a CO2 chamber and spleens and livers were removed and homogenized in 1 ml of Trizol™ (Invitrogen Corp., Carlsbad, CA). RNA was purified following the recommended protocol from the manufacturer. Research was conducted in compliance with the Animal Welfare Act and other federal statutes and regulations relating to animals and experiments involving animals and adhered to principles stated in the *Guide for the Care and Use of Laboratory Animals *[40]. The facility where this research was conducted is fully accredited by the Association for Assessment and Accreditation of Laboratory Animal Care International.

Two types of reference samples were used in this study to effectively profile the gene expression *in vivo*: Bm genomic DNA and Bm RNA samples from various cultures. Genomic DNA works as a universal reference and makes possible the comparison of gene expression levels among genes within an experiment [41].

Bm genomic DNA was prepared from an LB-grown Bm culture grown to mid-log phase (OD_600 _= 1.0) using DNeasy Tissue kit (QIAGEN Inc., Valencia, CA). Genomic DNA was digested with *Sau*3AI (New England Biolabs, Beverly, MA) and purified with a QIAquick PCR purification kit (QIAGEN Inc., Valencia, CA) before labeling and hybridization. Digested genomic DNA (2 μg) was labeled with amino-allyl-dUTP (Amersham-Pharmacia, Piscataway, NJ) using random primers in the presence of Klenow enzyme (Invitrogen Corp., Carlsbad, CA), followed by coupling to the Cy3 or Cy5 esters (Amersham-Pharmacia, Piscataway, NJ).

The six media used for Bm cultures were Luria-Bertani broth (LB) (Difco), M9 supplemented with glucose at 0.5% (M9 glucose), M9 glucose with 0.5% liver infusion (Difco), 1% liver infusion, 1% liver infusion with limited O^2 ^supply, 1% liver infusion with limited O^2 ^supply and with 10 mM of the Ca^2+ ^-chelating agent EGTA (Sigma-Aldrich, St. Louis, MO). Cultures were grown up to late-log phase (OD_600 _= 0.9 for M9; OD_600 _= 1.5 for media with liver infusion) at 37°C with moderate shaking. Aliquots of cultures were withdrawn and rapidly mixed with 1.5 volumes of RNAprotect Bacteria Reagent (QIAGEN Inc., Valencia, CA) to prevent the degradation of RNA. Cells were immediately harvested and RNA prepared using the RNeasy kit (QIAGEN Inc., Valencia, CA) according the manufacturer's protocols.

RNA from the same organ types from three mice was pooled to compensate for potential individual variation. These pooled RNA samples, which contain both Bm and the host RNA, were used for the experiments without further purification of the Bm RNA, since RNA from uninfected mouse did not hybridize efficiently to the Bm microarray (data not shown). The samples were paired with culture RNA samples or genomic DNA for the hybridization reactions shown in Figure 1. A total of 24 hybridization reactions or 12 different comparisons were conducted, each of which was replicated in flip-dye pairs. Fluorescently labeled probes from RNA were prepared by an indirect labeling method which consists of synthesis of amino-allyl-dUTP-labelled (Amersham-Pharmacia, Piscataway, NJ) cDNA from total RNA with random priming, followed by coupling of Cy3- or Cy5 dyes to the aminoallyl residues in the cDNA. Fluorescent probes were cleaned with QIAquick PCR purification kit (QIAGEN Inc., Valencia, CA) using the instructions provided by the manufacturer before conducting hybridization reactions.

### Slide Hybridization, scanning, and image analysis

In order to block non-specific background during hybridization, slides were first prehybridized in 5 × SSC, 0.1% SDS and 1% bovine serum albumin at 42°C for 45 minutes as previously described [42]. Slides were then washed in water and isopropanol (Sigma, Saint Louis, MO) and dried before hybridization. Fluorescent probes were dried after purification and resuspended in hybridization buffer containing 50% formamide, 5 × SSC, and 0.1% SDS. Cy-3 and Cy-5 labeled probes were combined and hybridized to the slides overnight at 42°C in a humid chamber. Following hybridization, slides were washed sequentially in 2 × SSC and 0.1% SDS at 42°C for 5 min., in 0.1 × SSC and 0.1% SDS at room temperature for 5 min., and twice in 0.1 × SSC at room temperature for 2.5 min., and air dried. Hybridized slides were scanned using the Axon GenePix 4000B microarray scanner and the independent TIFF images from each channel were analyzed using TIGR Spotfinder ([43], TIGR, Rockville, MD) to assess relative expression levels. Data from TIGR Spotfinder were stored in AGED, a relational database designed to effectively capture microarray data.

### Data Normalization and analysis

Normalization is necessary to adjust for differences in labeling and detection efficiencies of the fluorescent labels and for differences in the quantity of starting RNA. Data was normalized using a local regression technique LOWESS (LOcally WEighted Scatterplot Smoothing) for hybridizations with RNA-based samples using a software tool MIDAS ([43], TIGR, Rockville, MD), while total intensity normalization was used for the hybridizations with genomic DNA samples. The resulting data was averaged from triplicate genes on each array and from duplicate flip-dye arrays for each experiment.

Differentially expressed genes at the 95% confidence level were determined using intensity-dependent Z-scores (with *Z *= 1.96) as implemented in MIDAS. The resulting lists of the genes were examined further by cross comparison between experiments using TIGR MEV [43], TIGR, Rockville, MD) using Euclidean distance and hierarchical clustering with average linkage clustering method.

### Microarray validation: RT-PCR analysis

Differential expression of selected genes was assessed by SYBR^® ^Green real-time quantitative reverse transcription-polymerase chain reaction (qRT-PCR) by using the ΔC_T _method implemented in the ABI 7900 (Applied Biosystems, Foster City, CA) with primers designed based on the coding sequences (Table S5). qRT-PCR reactions were performed using the same pooled *in vivo *sample (i.e. infected mouse spleen) and one of the culture samples (i.e. 1% liver infusion) used for microarray hybridization. Absolute transcript levels of the relevant transcripts were estimated and the two data sets were normalized based on the microarray data of BMA0713, which showed only a minor difference between the two conditions (i.e. log_2 _ratio of 0.20; Additional file 2). The resulting log_2_(mouse spleen/1% liver infusion) ratios were compared to the corresponding estimates derived from the microarray assays.

### Microarray data Availability

Microarray expression data presented in this manuscript are available through ArrayExpress [44] with accession numbers A-MEXP-206 (array design) and E-MEXP-334 (experimental data).

## Authors' contributions

HSK conceived of the study, carried out experimental design and analysis for the microarray experiments, comparative genomic analysis, and manuscript preparation. MAS, RLU, and DD contributed to sample preparation for the microarray experiments and participated in manuscript preparation. YY conducted all of the microarray hybridizations and SHS contributed to some of them. WCN participated in the design and coordination of the study and manuscript preparation. All authors have read and approved the manuscript.

## Supplementary Material

Additional File 1 Global relatedness of the genomes of Bm, Bp, and Bt. Comparative genomics display tool ACT (Wellcome Trust Sanger Institute) was used to depict the syntenic fragments present between Bp and Bm and Bp and Bt based on BLASTN data. Click here for file

Additional File 2 RT-PCR confirmation of microarray data. Microarray and RT-PCR data of brain infusion vs. mouse spleen (see Fig. 1A) is compared using the thirteen randomly picked genes. For each gene, primer sequences, expected amplicon size, and annotation are shown. Click here for file

Additional File 3 Genes significantly up- or down expressed in mouse spleen and liver compared to cultures as shown in Fig. 1A. Click here for file

Additional File 4 Comparison of the whole genomes in Fig. 3A. Click here for file

Additional File 5 Genes up- or down-expressed *in vivo *in Fig. 3B. Click here for file

Additional File 6 The 30 in vivo-expressed genes that are degenerated or unique to Bm in Fig. 3B. Click here for file
