# Supplementary material for: Bacterial genome adaptation to niches: Divergence of the potential virulence genes in three Burkholderia species of different survival strategies
Source: BMC Genomics. 2005 Dec 7;6:174. doi: 10.1186/1471-2164-6-174 (PMC1343551; doi:10.1186/1471-2164-6-174)
Supplement: Additional File 3 — Genes significantly up- or down expressed in mouse spleen and liver compared to cultures as shown in Fig. 1A. [file 1471-2164-6-174-S3.pdf]

Sup_Table_1. Genes significantly up- or down expressed in mouse spleen and liver compared to cultures as shown in Fig. 1A.

Gene	Description	Hybridization group in fig. 1A											
		1	2	3	4	5	6	7	8	9	10	11	12
**Group 1**													
BMAA0450	conserved hypothetical protein	3.35	2.34	3.34	3.41	2.98	3.29	2.34	1.86	3.06	2.76	3.78	3.33
BMAA0468	glutathione-independent formaldehyde dehydrogenase	3.73	4.29	3.76	3.24	3.85	4.42	3.08	3.82	4.37	4.37	3.68	3.87
BMAA1846	hypothetical protein	3.00	2.44	3.46	3.32	3.37	3.60	4.03	3.17	3.50	2.68	4.33	4.22
BMA0024	aldolase, class II	2.98	2.93	2.42	2.95	2.44	3.15	2.76	2.61	2.82	2.88	2.29	2.21
BMA0357	conserved domain protein	1.37	1.63	1.17	1.70	1.43	1.39	1.66	2.05	1.30	1.61	2.19	2.33
BMAA0763	hypothetical protein	2.72	2.26	2.45	2.34	2.29	2.72	2.88	2.72	2.08	1.97	2.36	2.41
BMA0576	YdjC-like family protein	0.62	0.94	1.03	1.12	1.16	1.35	1.69	1.69	1.29	0.62	2.45	2.31
BMAA0841	conserved hypothetical protein, degenerate	2.36	2.00	2.64	2.54	2.41	2.64	2.27	1.69	2.89	2.09	2.45	2.33
BMA3300	hypothetical protein	3.10	2.37	3.08	2.69	2.51	2.74	2.38	1.95	2.82	2.65	2.70	2.22
BMAA1323	outer membrane lipoprotein, OmpA/SmpA/OmlA family	2.45	2.39	2.64	2.03	2.34	2.86	1.50	1.10	2.12	2.45	2.50	1.94
BMAA1042	conserved hypothetical protein	2.18	2.10	1.90	2.33	2.25	2.04	2.27	2.25	2.27	1.90	1.98	2.68
BMAA1354	hypothetical protein	2.72	2.98	2.49	3.12	2.86	3.51	2.98	2.90	2.78	2.52	2.08	2.69
BMAA0326	hypothetical protein	1.80	1.49	1.71	2.60	1.53	1.96	2.21	2.76	2.23	1.75	2.98	3.35
BMA3297	oxidoreductase, FAD-binding family protein	2.65	3.41	2.96	3.16	2.69	3.43	2.39	2.94	2.79	3.05	3.17	2.68
BMA0967	hypothetical protein	2.35	3.44	2.38	2.63	2.17	3.23	2.07	3.25	2.39	3.13	3.18	2.48
BMAA0271	conserved domain protein	3.60	3.82	4.11	4.08	3.39	4.24	2.95	2.90	3.78	3.40	3.04	2.57
BMAA1128	ABC transporter, periplasmic substrate-binding protein	2.85	3.33	2.89	3.23	2.48	3.85	2.38	2.57	3.00	2.90	3.02	2.56
BMA2777	general secretory pathway protein K	2.23	1.94	1.99	1.91	1.71	1.71	2.26	1.74	1.78	1.75	2.97	3.02
BMAA1801	TonB protein, putative	2.42	3.26	2.46	3.27	2.41	3.12	2.64	2.83	1.96	1.94	2.96	2.87
BMAA1619	hypothetical protein	2.08	2.04	2.13	2.53	2.10	2.84	2.06	NaN	1.96	2.03	2.53	2.51
BMAA1970	conserved hypothetical protein	1.61	1.71	NaN	1.71	NaN	2.14	2.08	1.95	1.68	1.48	2.61	2.53
BMA0328	cation ABC transporter, periplasmic cation-binding protein, putative	2.47	2.66	2.43	2.59	1.95	2.46	2.41	2.35	2.19	2.10	2.85	2.60
BMA2738	outer membrane protein, OmpW family	3.22	3.59	2.17	0.87	1.93	1.56	0.55	0.91	2.03	2.22	2.90	2.47
BMA1684	hydrolase, alpha/beta fold family	1.74	2.02	1.78	2.31	1.39	2.18	1.97	1.76	1.89	1.90	2.63	2.77
BMAA1524	BapC protein	1.76	1.35	2.17	2.11	2.24	2.21	2.47	1.85	1.45	0.85	2.66	2.68
BMAA0180	isochorismatase family protein	1.29	0.94	1.62	1.50	1.57	1.43	1.84	1.00	1.93	1.08	2.74	2.67
BMAA2000	2-hydroxy-3-oxopropionate reductase	2.41	2.24	2.27	1.67	2.39	2.47	2.17	1.54	2.03	1.73	2.73	2.72
BMAA2045	major facilitator family transporter	2.29	2.64	2.53	2.93	2.08	2.74	2.62	2.67	2.81	2.36	2.79	2.80
BMA1944-LX	conserved hypothetical protein	2.00	0.89	2.15	1.82	1.97	0.86	0.76	0.60	1.64	1.16	-0.74	0.22
BMA0016-LX	hypothetical protein	1.11	0.99	NaN	1.12	1.33	1.32	0.79	1.02	1.08	0.33	-0.52	0.11
BMA2880-LX	3-oxoacyl-(acyl-carrier-protein) synthase III, putative	1.20	1.51	NaN	1.78	1.39	1.20	0.52	0.67	1.30	1.20	-0.59	0.05
BMA1754-LX	CoA transferase, CAIB/BAIF family	1.11	1.39	1.31	1.46	2.53	1.78	0.71	1.00	1.69	1.34	-0.01	0.00
BMAA0595-LX	fusaric acid resistance domain protein	3.42	3.53	3.21	2.40	3.02	3.27	2.02	1.38	2.12	2.63	0.02	-0.21
BMAA0059-LX	conserved hypothetical protein	2.88	2.45	2.39	2.15	2.84	2.60	1.41	0.59	2.13	1.80	-0.19	-0.15
BMAA0394-LX	lipoprotein, putative	1.85	1.60	1.48	2.24	2.02	2.20	1.31	0.69	1.83	1.55	-0.19	-0.16
BMA0985-LX	hypothetical protein	2.35	1.78	2.55	2.10	2.43	2.27	1.92	1.39	2.10	2.00	-0.12	-0.12
BMAA0651-LX	H-NS histone family protein	1.84	0.66	1.80	1.69	2.00	1.91	0.70	0.66	2.33	1.51	-0.17	-0.09
BMAA0073-LX	hypothetical protein	1.72	1.24	1.32	0.67	2.12	1.36	0.49	0.40	1.46	0.84	-0.41	-0.21
BMAA0098-LX	hypothetical protein	1.28	1.65	NaN	NaN	NaN	1.99	1.08	1.15	NaN	1.54	-0.33	-0.16
BMAA1383-LX	hypothetical protein	NaN	1.37	NaN	1.23	NaN	1.39	1.07	0.77	1.58	NaN	-0.27	0.07
BMA2847-LX	flagellar biosynthetic protein FlhB	1.16	1.00	1.94	0.81	1.44	1.25	0.38	0.18	1.06	1.01	-0.16	-0.40
BMAA0838-LX	hypothetical protein	1.76	1.52	NaN	NaN	NaN	2.10	0.51	0.51	1.41	1.78	-0.30	-0.64
BMAA1164-LX	membrane protein, putative	1.94	1.85	2.48	1.56	1.93	2.56	1.40	1.42	1.76	1.52	-0.02	-0.64
BMAA0589-LX	conserved hypothetical protein	2.00	1.01	2.15	0.81	1.82	1.17	0.22	0.30	0.27	0.75	-0.58	-0.57

BMAA1568-LX	serine protease, kumamolysin	2.20	1.47	2.08	1.98	1.25	1.16	1.09	1.19	2.10	2.00	-0.54	-0.46
BMAA0618-LX	hypothetical protein	1.70	0.59	1.15	0.67	1.59	0.91	0.40	0.22	0.96	0.86	-1.80	-1.77
BMAA0454-LX	conserved hypothetical protein	2.35	2.22	2.21	1.86	2.60	2.58	1.88	1.79	2.46	2.15	-1.15	-1.27
BMAA0594-LX	hypothetical protein	2.06	1.47	2.15	1.68	2.28	2.02	0.95	1.04	1.71	1.98	-0.91	-1.16
BMAA0922-LX	drug resistance transporter, EmrB/QacA family	2.96	2.46	2.59	2.78	2.20	3.05	1.03	1.01	1.42	2.18	-1.27	-1.57
BMA1634-LX	conserved hypothetical protein	1.96	1.30	2.31	2.21	2.28	1.70	1.45	0.69	1.96	1.64	-1.36	-1.36
BMAA0985-LX	hypothetical protein	3.42	2.84	2.42	2.05	2.66	2.54	0.61	2.35	2.20	3.00	-1.52	-1.30
BMAA0280-LX	membrane protein, putative	3.05	2.50	2.56	2.32	3.08	2.51	0.83	0.47	1.87	2.10	-0.93	-0.68
BMAA1842-LX	hypothetical protein	2.15	1.88	2.39	2.73	2.06	2.34	2.26	2.21	2.34	1.67	-1.00	-0.87
BMA2679	ABC transporter, ATP-binding protein	1.48	0.75	NaN	0.81	2.04	2.80	1.21	1.07	NaN	2.07	1.66	0.49
BMAA0646	hypothetical protein	1.35	1.72	0.59	1.48	1.16	2.24	0.32	1.23	0.91	1.90	1.80	0.59
BMAA1252	hypothetical protein	1.44	3.02	0.54	1.43	1.46	2.86	0.67	2.95	1.03	2.48	1.43	0.49
BMA3328	flagellar hook protein FlgE	1.28	2.54	1.23	1.09	1.23	2.59	1.16	2.16	1.30	2.29	1.20	0.46
BMAA1517	transcriptional regulator, araC family	0.43	0.78	0.58	NaN	NaN	0.99	1.39	0.95	0.19	0.07	1.11	0.39
BMA0988	cytochrome c family protein	1.01	1.57	0.77	1.64	0.78	1.44	1.66	2.11	1.28	1.67	1.12	0.66
BMAA1419	proline racemase, putative	0.87	1.48	NaN	1.42	0.83	1.82	1.32	1.56	1.28	1.41	1.04	0.61
BMAA0689	hypothetical protein	1.85	2.14	2.57	2.60	2.04	2.93	2.35	1.68	2.39	1.77	1.07	0.72
BMAA0410	Rhs element Vgr protein	2.66	2.21	3.17	2.44	2.63	2.63	2.19	1.11	2.76	2.19	1.04	0.80
BMAA1540	type III secretion system protein BsaS	0.30	1.07	0.38	0.85	0.62	1.34	0.75	1.94	0.36	0.95	1.08	0.77
BMAA1625	type III secretion inner membrane protein, authentic frameshift	0.38	1.01	NaN	2.08	-0.01	2.10	1.32	1.64	NaN	0.71	1.26	0.75
BMA0305	conserved hypothetical protein, authentic point mutation	1.77	1.74	1.72	1.73	2.08	2.28	1.91	1.11	1.89	1.63	1.24	0.83
BMAA1202	polyketide synthase, putative, degenerate	NaN	0.88	NaN	-0.03	0.26	1.28	1.29	1.39	NaN	0.75	1.26	0.85
BMAA1887	hypothetical protein	2.47	2.60	2.39	2.62	2.38	3.05	1.82	1.96	2.30	2.39	1.17	0.85
BMAA1390	conserved hypothetical protein	0.40	1.63	NaN	NaN	1.01	2.25	0.88	1.60	1.09	1.49	0.85	-0.50
BMA0017	hypothetical protein	1.98	1.88	2.77	2.18	2.75	2.95	1.65	1.30	1.99	1.54	0.67	0.22
BMA0224	hypothetical protein	2.92	3.12	2.57	3.36	2.81	3.34	2.01	2.71	2.84	2.71	0.76	0.17
BMAA0323	hypothetical protein	1.01	1.58	NaN	NaN	NaN	1.64	0.90	1.13	NaN	1.35	0.89	0.23
BMA1854	Ser/Thr protein phosphatase family protein	2.62	2.40	2.94	2.82	2.70	2.98	2.50	2.27	2.83	2.28	0.92	0.71
BMA0040	conserved hypothetical protein	2.75	2.44	2.26	2.79	2.46	2.83	0.55	0.82	2.68	2.17	0.83	0.71
BMA1900	carboxymuconolactone decarboxylase family protein	-0.27	NaN	NaN	NaN	0.03	NaN	1.10	1.71	NaN	0.16	0.81	NaN
BMA2908	conserved hypothetical protein	2.07	2.06	2.68	2.98	1.67	2.30	2.02	1.56	2.22	0.98	0.81	0.69
BMA3068	hypothetical protein	2.61	2.39	2.52	2.73	2.96	2.84	1.33	1.12	2.42	1.93	0.74	0.78
BMA0887	di-haem cytochrome c peroxidase family protein	2.65	2.10	3.02	3.24	3.00	2.79	2.97	2.29	3.11	1.72	0.67	0.87
BMA3224	conserved hypothetical protein	0.79	0.94	NaN	1.02	0.63	1.29	1.02	0.28	0.51	0.45	0.66	0.70
BMAA1151	conserved hypothetical protein	2.11	1.44	1.71	1.95	1.99	2.10	2.43	1.58	2.19	1.25	0.62	0.76
BMA3246	homoserine O-acetyltransferase	3.10	2.79	2.51	2.75	2.46	2.44	1.00	0.99	2.22	2.01	0.52	0.52
BMAA0848	4-hydroxyphenylpyruvate dioxygenase, putative	1.54	0.23	NaN	2.03	1.56	2.07	1.95	1.49	NaN	1.36	0.59	0.45
BMA2589	flavodoxin domain protein	1.48	1.42	NaN	1.20	1.38	1.12	1.21	0.63	1.46	1.08	0.79	0.43
BMA3012	hypothetical protein	2.85	2.56	2.16	2.61	2.53	3.04	1.38	1.21	2.89	2.90	0.71	0.37
BMAA0628	hypothetical protein	2.13	1.79	3.08	1.69	2.47	2.29	2.15	1.63	2.99	2.25	0.73	0.39
BMAA2018	conserved hypothetical protein	2.00	1.85	2.12	2.15	2.37	2.58	2.12	1.41	2.27	1.59	0.73	0.57
BMA1621	cysteine synthase/cystathionine beta-synthase family protein	1.17	1.79	1.76	1.72	1.36	2.35	1.59	1.08	1.68	1.28	0.33	-0.06
BMA1058	drug resistance transporter, EmrB/QacA family	1.87	1.21	2.05	1.97	1.60	1.28	1.30	0.95	2.03	1.70	0.28	0.20
BMA1473	outer membrane porin, putative	0.63	1.09	NaN	1.20	1.02	1.32	1.44	0.83	1.31	1.04	0.22	0.18
BMA2247	hypothetical protein	1.33	1.81	NaN	1.11	2.09	1.64	0.69	0.77	0.73	1.21	0.25	0.11
BMAA0259	extracellular nuclease, putative	0.83	1.35	NaN	NaN	NaN	1.33	1.17	1.06	NaN	1.22	0.16	0.08
BMA0824	poly(3-hydroxybutyrate) depolymerase	1.30	1.26	0.95	1.15	1.75	1.66	0.88	0.61	1.38	1.26	0.19	0.26
BMA0702	hypothetical protein	3.32	3.32	2.68	3.18	2.96	3.05	1.86	2.69	2.79	2.80	0.14	0.34
BMAA1184	conserved hypothetical protein	-0.03	0.13	NaN	0.77	NaN	0.62	1.22	1.38	NaN	NaN	0.18	0.35

BMAA1486	O-methyltransferase family protein	0.20	0.16	0.22	2.19	1.87	1.66	1.42	0.59	1.74	1.90	0.48	0.23
BMAA1520	type III secretion chaperone BicP	0.78	0.55	0.51	1.24	1.25	1.23	1.25	1.04	0.31	0.22	0.38	0.42
BMAA2047	molybdopterin oxidoreductase family protein	2.28	1.70	1.91	2.50	2.30	2.03	1.79	1.63	2.49	2.17	0.39	0.31
BMA0014	hypothetical protein	2.89	3.05	2.46	2.12	2.97	2.06	1.40	3.19	1.37	0.87	0.29	1.68
BMA0267	conserved domain protein, truncation	4.45	4.37	2.67	3.06	3.35	3.50	1.61	1.87	2.59	2.76	0.49	1.64
BMA1044	conserved hypothetical protein	3.11	2.84	1.59	2.64	3.05	2.25	1.12	2.20	2.94	2.81	0.04	1.60
BMAA0474	oxidoreductase, FAD/FMN-binding	3.31	3.53	1.00	1.89	3.22	2.43	0.23	2.36	2.93	3.17	0.11	1.92
BMA1175	cob(I)alamin adenosyltransferase	2.95	3.53	1.39	2.45	2.14	1.59	1.11	2.49	2.08	2.40	0.68	2.10
BMAA1104	oxidoreductase, short chain dehydrogenase/reductase family	1.79	1.97	0.77	2.27	1.73	1.49	0.86	2.23	1.88	2.00	0.68	1.87
BMAA1162	hypothetical protein	2.02	2.32	1.13	2.22	2.27	1.84	1.37	2.25	1.92	1.59	0.69	1.88
BMAA0451	lipoprotein, putative	1.83	2.01	2.10	2.42	2.13	1.96	1.54	1.65	1.74	1.71	0.25	0.97
BMAA0775	hypothetical protein	0.86	1.44	NaN	1.67	1.04	1.38	0.76	1.82	NaN	1.62	0.16	1.06
BMAA0566	transcriptional regulator, AraC family	1.83	1.20	NaN	NaN	NaN	1.20	0.16	0.02	0.94	1.31	0.64	1.01
BMA1198	ribose ABC transporter, periplasmic ribose-binding protein	0.96	1.63	-0.26	1.30	0.36	1.07	0.40	2.10	NaN	1.19	0.70	1.13
BMA1637	membrane protein, putative	1.67	1.69	1.78	2.19	1.98	1.61	1.73	1.88	2.14	1.56	0.60	1.15
BMAA1434	hypothetical protein	1.91	1.43	1.48	1.77	2.26	1.76	1.77	1.29	1.69	1.46	0.61	1.11
BMAA0386	D-serine dehydratase, authentic frameshift	1.65	1.65	1.47	2.14	1.58	1.75	1.42	1.62	2.13	1.32	0.47	0.92
BMAA1912	conserved hypothetical protein	0.61	0.79	1.77	1.28	1.52	1.10	0.91	0.37	0.81	0.49	0.49	1.02
BMAA1044	efflux transporter, RND family, MFP subunit	1.32	1.07	NaN	NaN	1.01	0.87	0.75	1.45	NaN	0.93	0.46	1.25
BMAA1935	hypothetical protein	1.58	1.64	NaN	1.47	2.00	1.09	NaN	1.55	1.91	1.16	0.49	1.20
BMAA1660	sensory box sensor histidine kinase	1.13	2.14	1.05	2.05	1.16	0.78	-0.25	1.40	1.53	2.23	-0.47	0.92
BMAA1116	conserved hypothetical protein	2.01	1.77	2.16	2.09	1.99	1.69	1.76	1.62	2.20	1.55	0.27	0.69
BMAA0560	arsenical resistance transcriptional regulator	1.93	1.53	2.42	2.07	2.03	1.79	2.27	1.65	2.26	1.70	0.09	0.48
BMAA0955	hypothetical protein	1.64	1.69	1.50	2.07	2.21	2.30	1.61	1.55	1.96	1.55	0.09	0.47
BMA1732	nitrate reductase, beta subunit	0.51	0.53	0.59	1.96	2.27	1.91	1.08	1.19	2.19	1.64	0.05	0.57
BMAA1475	porin, degenerate	2.31	2.00	1.93	2.08	1.74	1.73	0.91	0.98	2.20	1.73	0.02	0.61
BMA0642	conserved hypothetical protein, degenerate	1.37	1.42	NaN	2.24	2.51	1.35	1.10	2.18	2.18	1.60	-0.45	0.51
BMAA1498	O-antigen acetylase, putative	2.91	2.35	2.39	2.77	2.50	2.15	1.78	2.25	2.53	2.11	-0.25	0.69
BMAA1757	hemolysin activator protein, HlyB family	1.59	1.26	0.49	1.35	1.80	1.07	-0.17	0.76	1.05	1.43	-0.32	0.60
BMAA0673	hypothetical protein	1.01	0.76	1.14	1.34	2.04	1.34	1.24	0.40	2.32	1.30	-0.28	0.39
BMA1239	alkanesulfonate monooxygenase	2.14	1.78	NaN	2.22	1.96	1.76	1.29	1.12	1.65	1.63	-0.11	0.47
BMAA0752	hypothetical protein	2.29	1.44	1.82	2.22	2.38	2.30	2.08	1.26	1.71	1.06	-0.17	0.38
BMAA1945	hypothetical protein	3.38	3.17	2.81	2.59	2.95	2.79	1.27	1.25	2.33	2.59	-0.07	0.35
BMAA1995	conserved domain protein	2.33	2.28	2.10	3.23	2.26	2.62	2.11	2.04	2.33	2.40	-0.01	0.34
BMA1038	penicillin amidase, putative	1.35	1.50	1.43	1.39	1.71	1.67	1.16	1.08	1.63	1.80	1.46	2.15
BMAA0957	conserved hypothetical protein	1.30	1.52	0.90	2.05	1.33	1.79	1.50	1.76	1.18	1.63	1.64	2.28
BMAA0154	sulfotransferase domain protein	2.71	2.88	1.83	3.30	3.14	2.53	2.12	2.44	2.85	2.24	1.19	2.10
BMAA1109	hypothetical protein	2.24	2.02	2.05	2.59	2.19	2.23	2.45	2.42	2.23	1.87	1.35	1.96
BMA0101	8-amino-7-oxononanoate synthase	2.50	3.16	1.69	2.96	2.50	1.97	1.35	3.14	2.37	2.80	1.17	2.42
BMAA2006	flavin reductase domain protein	2.11	2.49	0.60	2.35	2.12	1.79	1.12	2.17	2.20	2.25	1.28	2.47
BMAA0493	conserved hypothetical protein	2.79	2.94	2.53	2.93	2.38	3.10	1.52	1.74	2.57	2.60	2.12	1.22
BMAA0503	hemK protein	0.85	1.22	1.85	1.66	1.18	2.13	1.81	1.99	1.87	1.84	2.15	1.30
BMA2282	amino acid permease, truncation	2.54	2.71	2.46	1.56	2.03	2.58	0.76	0.92	2.03	0.95	1.87	1.41
BMAA0731	hypothetical protein	1.27	1.81	1.34	1.52	0.81	1.86	1.77	2.42	0.50	0.90	1.87	1.38
BMA1639	methyltransferase, putative/adenylsulfate kinase	2.08	2.98	2.20	1.48	2.14	2.88	1.75	2.75	1.69	2.37	1.77	1.39
BMAA1683	formate dehydrogenase, alpha subunit, selenocysteine-containing, authentic point mutation	2.38	2.25	1.60	0.89	1.79	1.90	1.61	1.51	2.40	2.44	1.71	1.37
BMAA0664	EAL/GGDEF domain protein	2.29	1.42	3.14	2.59	2.26	3.01	2.64	2.42	2.84	2.57	1.88	1.66
BMAA0321	conserved hypothetical protein	3.13	3.13	3.09	3.13	3.01	3.81	2.42	2.33	3.16	2.99	1.76	1.68
BMAA0372	hypothetical protein	1.08	0.92	NaN	0.88	2.24	1.78	1.95	1.83	1.20	1.28	1.69	1.73

BMAA0895	hypothetical protein	2.56	2.60	2.23	2.64	2.06	2.48	1.74	1.85	1.87	2.53	1.69	1.69
BMAA1624	conserved hypothetical protein	1.47	1.39	NaN	1.90	NaN	1.69	1.65	1.72	1.74	0.97	1.60	1.57
BMA0320	xanthine/uracil permease family protein, truncation	3.10	3.27	2.73	3.33	2.48	3.72	2.40	2.22	2.72	2.72	1.65	1.48
BMA2860	chemotaxis response regulator	1.50	2.30	1.31	1.50	1.40	2.56	1.36	1.99	1.09	1.98	1.67	1.48
BMA0030	ElaA family protein	1.34	1.33	1.10	1.70	1.52	1.83	1.92	1.56	1.50	1.26	1.74	1.54
BMAA1841	glyoxalase family protein	1.50	1.03	1.54	1.56	1.10	1.37	2.08	1.46	1.28	1.10	1.74	1.52
BMA1561	hypothetical protein	0.79	1.09	NaN	1.41	NaN	1.75	1.42	1.50	1.11	1.18	1.77	2.15
BMA0678	isoleucine biosynthesis transcriptional activator	2.78	2.79	2.69	2.64	2.54	2.62	2.46	1.88	2.31	2.35	1.86	2.16
BMA3132	molybdopterin oxidoreductase family protein	1.42	1.54	1.78	1.90	1.68	1.76	2.00	1.85	1.90	1.72	1.84	2.20
BMA2131	6-phosphogluconolactonase	2.32	2.01	1.94	2.24	2.27	2.67	1.87	1.87	1.58	1.27	2.03	2.28
BMAA0363	sigma-54 dependent DNA-binding transcriptional regulator	2.47	2.31	2.16	2.28	2.26	2.15	2.82	2.63	2.51	2.24	1.96	2.22
BMA1194	carbohydrate kinase, FGGY family	1.41	1.94	1.57	1.43	1.56	2.19	1.87	1.93	1.23	1.55	2.14	1.88
BMA2576	phenylacetic acid degradation protein PaaD	2.03	2.80	1.88	2.98	1.97	3.01	1.80	2.00	1.68	2.65	2.10	1.99
BMAA0242	acyl-CoA dehydrogenase domain protein	2.02	1.89	1.88	1.95	2.24	2.17	2.16	1.64	2.20	1.95	2.14	1.95
BMA1638	peptide synthetase-domain protein	1.84	1.88	2.03	2.43	1.76	2.93	2.36	2.32	1.56	2.03	2.08	2.09
BMAA1062	conserved hypothetical protein	2.13	2.11	2.36	2.56	2.35	2.51	2.94	2.28	2.24	1.92	2.05	2.04
BMA0912	iolD protein	1.35	1.58	0.92	1.26	1.09	1.83	1.52	1.77	1.24	1.29	2.08	2.03
BMAA0641	hydrolase	1.70	1.65	1.76	2.11	1.55	2.46	2.29	1.96	2.02	1.78	2.09	2.02
BMAA1428	lactate permease family protein	2.28	2.32	2.43	2.45	2.21	2.59	1.90	2.24	2.61	2.74	2.10	2.03
BMA2157	conserved hypothetical protein	1.49	2.14	1.76	2.21	1.86	1.57	1.78	1.77	NaN	1.73	1.86	1.92
BMAA0825	hypothetical protein	NaN	1.29	NaN	NaN	NaN	1.61	1.50	1.78	NaN	0.98	1.84	1.87
BMAA1397	conserved hypothetical protein	1.65	2.05	1.59	2.18	2.05	2.50	2.16	2.02	2.04	1.60	1.84	1.86
BMA2222	phenazine biosynthesis protein phzF, putative	1.20	1.64	1.24	1.90	1.50	2.23	1.90	1.64	1.25	1.41	1.63	1.93
BMAA0562	hypothetical protein	1.90	1.99	2.38	2.35	2.40	2.58	2.05	-0.34	1.74	1.73	1.62	1.98
BMA1505	allantoicase	2.45	2.63	2.15	2.04	2.33	2.68	1.21	1.51	2.32	2.49	1.80	1.98
BMAA1248	serine metalloprotease MrpA	1.61	1.59	1.33	1.79	1.56	1.98	1.67	1.35	1.85	1.27	1.77	2.03
BMAA1865	conserved hypothetical protein	2.20	2.00	2.52	2.30	2.43	2.18	1.66	1.45	2.40	1.57	1.73	1.95
BMAA0188	transcriptional regulator CatR	1.74	2.31	1.16	1.73	1.82	2.65	1.85	1.52	1.88	1.86	2.24	1.56
BMAA0776	transcriptional regulator, LysR family	NaN	1.38	NaN	1.51	1.42	1.82	2.08	1.33	NaN	1.02	2.25	1.79
BMAA0261	conserved hypothetical protein	1.38	1.24	1.32	1.04	1.79	1.56	1.97	1.52	1.44	0.76	1.98	1.79
BMA1113	conserved hypothetical protein	3.89	3.29	2.56	2.60	3.64	3.82	1.71	1.27	3.31	2.97	1.99	1.67
BMA1123	peptide synthetase, putative	1.06	1.57	1.02	1.45	1.80	1.95	1.45	1.29	1.44	1.72	2.10	1.71
BMA0906	thioesterase family protein	1.28	1.53	1.16	0.77	0.68	1.52	1.79	2.19	1.22	1.32	2.02	1.72
BMAA1983	hypothetical protein	2.19	1.68	2.59	1.76	2.71	2.15	2.43	1.89	1.98	2.01	2.05	1.76
BMA3158	AMP-binding domain protein	0.88	1.16	0.95	1.68	1.34	1.53	1.93	1.40	0.84	0.92	1.38	1.26
BMAA1871	propionate catabolism operon regulatory protein	1.29	2.36	1.23	1.70	1.58	2.09	1.53	2.49	1.86	2.24	1.30	1.20
BMAA1149	hypothetical protein	1.91	1.65	2.71	NaN	2.57	2.24	1.93	1.20	2.04	2.05	1.42	1.05
BMA0575	hypothetical protein	1.24	1.57	1.58	1.40	1.39	1.71	1.65	1.26	1.51	1.16	1.45	1.13
BMAA1895	conserved domain protein	2.25	1.58	1.63	2.36	1.53	1.95	0.90	1.26	2.14	1.49	1.50	1.17
BMAA1384	hypothetical protein	2.31	2.37	3.27	2.75	2.70	3.06	2.43	1.16	2.98	1.81	1.30	1.01
BMAA1617	hrp protein, putative	0.31	0.36	0.34	NaN	0.22	1.01	1.38	0.83	NaN	0.38	1.25	1.09
BMA1959	MOSC domain protein	2.73	2.59	2.88	2.47	2.36	2.63	1.60	1.16	1.96	2.04	1.18	0.95
BMAA1690	luciferase-like monooxygenase	1.46	1.84	1.73	1.96	2.50	2.30	1.87	1.81	1.71	1.73	1.18	0.96
BMAA1423	malate/L-lactate dehydrogenase family protein	0.29	0.59	-0.15	0.71	0.29	0.84	1.74	0.87	0.05	0.50	1.16	1.18
BMA2110	conserved hypothetical protein	1.62	1.54	1.61	NaN	1.26	1.76	1.64	1.68	1.63	1.20	1.14	1.11
BMAA0462	rhamnosyltransferase II	3.22	2.99	3.11	3.28	3.27	3.16	2.23	2.36	2.98	2.63	1.11	1.11
BMAA0619	transcriptional regulator, MarR family	1.76	1.80	1.79	2.00	1.59	2.04	2.19	2.02	1.34	1.46	1.17	1.11
BMAA1902	conserved hypothetical protein	1.51	1.85	NaN	1.75	2.73	1.82	2.24	1.50	2.37	1.72	1.17	1.07
BMA0989	hypothetical protein	2.57	1.76	2.78	2.53	1.89	1.80	1.51	1.20	2.37	1.94	0.99	1.08

BMA2152	LysE family protein	3.22	3.14	2.51	2.82	3.06	2.77	1.65	2.55	2.82	3.07	0.96	1.11
BMAA1011	hypothetical protein	0.25	0.41	0.84	0.65	0.73	0.60	1.18	1.12	0.96	0.52	0.93	1.11
BMA0697	cobyric acid synthase CobQ	0.94	1.37	1.12	1.79	1.93	1.96	1.97	1.56	1.21	1.37	1.06	1.13
BMA0011	hypothetical protein	1.57	0.91	NaN	1.14	1.79	1.05	1.80	0.83	2.16	1.20	1.01	1.15
BMAA1925	hypothetical protein	0.50	0.14	0.66	0.58	0.79	1.22	1.39	0.67	0.83	0.26	1.03	1.16
BMAA0753	SCO1/SenC family protein	0.68	0.87	0.47	0.94	0.93	1.21	1.39	0.97	0.86	0.79	1.02	1.01
BMAA0449	conserved hypothetical protein	2.09	2.37	1.39	2.57	2.07	2.73	1.43	1.65	1.44	1.95	1.04	0.94
BMAA1920	quinone oxidoreductase, putative	2.20	2.28	2.09	2.72	2.38	2.37	1.67	1.88	2.14	2.38	0.98	0.94
BMAA1932	transcriptional regulator, LysR family	NaN	1.21	NaN	NaN	0.16	1.17	0.87	0.78	NaN	1.25	1.00	0.97
BMA2134	amino acid ABC transporter, periplasmic amino acid-binding protein	2.05	1.51	1.61	1.96	1.76	1.72	1.05	0.92	2.02	1.59	0.81	0.96
BMA1683	phosphoesterase, putative	1.77	2.18	1.61	2.36	1.84	2.34	2.13	2.03	1.52	1.81	0.78	0.92
BMAA1187	RNA polymerase sigma-70 factor, ECF subfamily	1.76	1.03	NaN	NaN	0.62	1.42	1.40	1.60	NaN	0.87	0.81	0.92
BMAA0923	rhamnosyltransferase family protein	2.79	2.49	2.50	2.89	2.60	2.71	2.28	1.88	2.47	1.95	0.89	0.89
BMAA1211	hypothetical protein	3.36	3.12	3.08	3.29	2.97	3.09	2.39	2.60	2.88	2.90	0.85	0.90
BMAA1032	hypothetical protein	0.83	1.11	-0.12	1.10	1.27	1.37	0.47	1.57	1.09	1.18	0.86	1.01
BMAA1973	conserved hypothetical protein	2.18	2.27	1.45	2.18	1.82	2.35	1.73	2.18	1.96	2.01	0.86	1.05
BMA0687	iron compound ABC transporter, ATP-binding protein	1.34	1.60	0.64	1.72	1.57	1.19	1.08	1.97	1.22	1.23	0.90	1.60
BMA0930	fosmidomycin resistance protein	3.22	2.77	2.64	2.79	2.97	2.36	1.32	1.70	2.67	2.36	0.97	1.56
BMAA0577	2-hydroxy-3-oxopropionate reductase	1.11	1.08	0.32	1.59	1.64	1.67	0.50	0.76	1.30	1.74	1.03	1.48
BMAA0755	outer membrane nitrite reductase, putative	2.56	2.56	2.78	2.95	2.80	2.53	2.49	2.47	2.94	2.48	0.97	1.44
BMAA0616	hypothetical protein	1.10	0.95	NaN	0.41	2.09	1.21	1.12	1.05	1.31	0.60	0.93	1.30
BMA2007	hypothetical protein	2.38	2.60	1.55	2.86	2.32	2.16	1.15	1.60	2.12	1.94	0.78	1.18
BMA3168	transcriptional regulator, GntR family	0.56	1.00	0.86	NaN	1.19	1.21	1.02	0.79	0.32	1.01	0.83	1.17
BMA1163	precorrin-2 C20-methyltransferase	1.33	1.16	1.33	1.68	1.38	1.53	2.32	1.79	1.53	1.21	0.88	1.22
BMAA1526	BapA protein	1.40	1.10	1.41	1.95	1.98	1.95	2.08	1.49	0.98	0.58	0.91	1.19
BMAA1622	conserved hypothetical protein	2.07	1.57	2.05	1.74	1.89	1.66	2.19	1.27	1.61	1.31	0.91	1.16
BMAA0597	hypothetical protein	1.92	1.71	2.35	2.40	2.21	1.90	1.78	1.56	2.13	1.62	1.12	1.32
BMA0156	HpcH/HpaI aldolase family protein	1.36	1.41	0.98	1.59	1.47	1.36	1.54	2.09	1.46	1.21	1.05	1.26
BMAA2031	sulfate permease family protein	1.75	1.91	1.46	2.10	1.67	1.91	1.18	2.29	1.54	2.27	1.04	1.35
BMA3323	flagella basal body P-ring formation protein FlgA	2.69	2.32	2.52	2.64	2.31	2.71	2.52	2.42	2.02	2.07	1.52	1.43
BMAA0610	di-haem cytochrome c peroxidase family protein	1.43	1.31	NaN	NaN	NaN	1.88	1.43	0.88	NaN	1.42	1.50	1.50
BMAA1133	transcriptional regulator, AraC family	1.73	1.30	NaN	1.50	1.74	1.71	1.53	1.15	1.85	0.86	1.45	1.43
BMAA1875	hypothetical protein	2.21	2.39	1.96	1.73	1.90	2.36	1.50	1.00	1.96	1.48	1.41	1.46
BMA0551	conserved hypothetical protein	2.34	1.76	2.04	2.52	2.55	2.26	2.74	2.01	2.68	1.96	1.50	1.31
BMA2177	branched-chain amino acid ABC transporter, permease protein, putative	2.61	2.87	1.78	2.30	2.41	2.82	1.57	1.54	1.56	2.43	1.45	1.29
BMA2978	membrane protein, putative	1.90	2.48	1.36	1.60	1.58	2.29	1.05	1.20	1.16	1.51	1.58	1.36
BMAA1927	conserved domain protein	2.54	0.78	3.06	1.86	2.33	2.32	1.51	0.87	2.75	1.19	1.54	1.37
BMA3058	carotenoid 9,10-9',10' cleavage dioxygenase, putative	1.83	1.85	1.61	2.03	1.95	2.03	2.14	1.74	2.12	1.57	1.27	1.38
BMAA1190	fumarylacetoacetate hydrolase family protein	0.78	0.75	-0.20	0.13	0.55	0.52	1.15	0.86	NaN	0.58	1.14	1.40
BMA0952	RND efflux system, outer membrane lipoprotein, NodT family	1.13	1.35	1.03	1.48	0.98	1.57	1.94	2.03	1.43	1.18	1.20	1.44
BMAA1621	regulatory protein HrpB	2.10	2.29	1.91	2.61	1.93	2.37	2.44	2.46	1.88	2.05	1.18	1.47
BMAA1986	ADP-heptose--LPS heptosyltransferase II, putative	1.35	1.70	2.02	2.06	1.51	1.81	1.79	1.64	1.59	1.27	1.32	1.54
BMAA0367	acetyltransferase, GNAT family	0.29	0.23	-0.08	0.17	0.36	0.16	0.71	1.40	-0.01	-0.01	1.35	1.46
BMA2855	chemotaxis protein CheD	1.23	2.02	1.47	2.16	1.44	1.96	1.42	1.70	2.29	1.78	1.30	1.49
BMAA1987	glycosyl transferase, group 2 family protein	1.20	1.53	0.69	1.75	1.88	1.47	1.46	1.74	1.11	1.10	1.31	1.47
BMAA0407	conserved hypothetical protein	0.98	1.39	0.80	1.25	1.38	1.14	0.61	1.75	1.20	1.55	1.07	1.68
BMAA0798	mulitcopper oxidase domain protein	2.52	2.29	1.97	2.75	2.37	2.30	1.86	1.83	2.08	2.06	1.05	1.78
BMAA1045	hydrophobe/amphiphile efflux family protein	2.17	1.79	2.18	2.30	2.38	1.88	1.79	1.35	2.65	1.46	1.12	1.59
BMAA1091	regulatory protein NasS, putative	1.14	1.77	1.03	1.48	1.88	1.80	1.05	0.69	0.84	1.14	1.29	1.71

BMAA0522	hypothetical protein	2.76	2.42	2.17	2.63	2.96	2.62	2.36	2.34	2.41	2.10	1.18	1.75
BMAA0852	aminotransferase, class V	2.13	1.44	1.18	2.61	NaN	2.15	1.77	1.66	1.32	1.56	1.17	1.74
BMAA1098	acetylpolyamine aminohydrolase, putative	1.65	1.85	NaN	1.34	1.80	1.58	1.03	1.56	1.61	1.41	1.22	1.75
BMAA1372	conserved hypothetical protein	1.62	1.86	1.35	1.96	1.68	1.81	1.61	2.20	1.71	1.97	1.19	1.68
BMAA0369	indole-3-acetamide hydrolase-related protein	2.14	2.44	2.13	2.35	2.01	2.55	2.32	2.30	2.01	2.14	1.51	1.78
BMAA0630	aldehyde dehydrogenase (NADP) family protein	0.91	0.68	1.18	1.27	0.90	0.97	1.61	1.21	0.88	0.70	1.53	1.84
BMA0258	hypothetical protein	0.99	1.16	1.50	1.63	NaN	1.50	1.93	1.60	1.09	1.03	1.54	1.68
BMAA1368	conserved hypothetical protein	2.03	2.01	NaN	1.37	1.46	1.66	1.72	1.85	2.04	1.79	1.47	1.70
BMAA2016	hypothetical protein	0.98	1.44	NaN	1.60	1.89	2.12	2.23	1.68	1.81	1.27	1.49	1.73
BMAA0785	sensor histidine kinase	1.25	0.97	0.77	1.10	1.06	1.05	1.70	1.47	0.85	1.00	1.43	1.66
BMAA0855	ABC transporter, periplasmic substrate-binding protein	2.43	2.67	3.05	3.19	2.44	3.16	2.65	2.14	2.09	2.60	1.46	1.58
BMAA2082	hypothetical protein	1.93	1.71	1.93	2.12	2.04	2.06	1.46	1.81	2.00	2.02	1.42	1.59
**Group2**													
BMA2331	heat-inducible transcription repressor HrcA	-1.92	-1.98	-1.42	-2.28	-1.15	-1.46	-1.00	-0.86	-1.40	-1.27	0.71	1.10
BMA0335	maltose/mannitol ABC transporter, permease protein, putative	-1.47	-0.51	-2.00	-2.78	-1.15	-1.22	-1.67	-2.04	-1.60	-1.36	0.69	0.88
BMA0317	RND efflux system, outer membrane lipoprotein, NodT family	-1.15	-1.01	-0.40	-2.42	-1.22	-1.19	-1.11	-1.19	-1.74	-1.74	0.73	0.92
BMA1120	histidinol-phosphate aminotransferase, putative, authentic point mutation	NaN	-0.50	NaN	-3.75	-0.95	-1.88	-1.79	-2.02	-1.16	-1.37	0.76	0.87
BMA1380	molybdopterin converting factor, subunit 2	-0.46	-1.77	-0.74	-0.92	-0.81	-1.38	-0.24	-0.84	0.01	-0.16	0.62	0.75
BMA2326	chaperone protein DnaK	-2.14	-1.31	-1.93	-0.99	-1.34	-1.17	-1.36	-0.81	-1.06	-0.92	0.57	0.84
BMA0310	serine protease	-0.95	-0.71	-1.05	-1.04	-0.48	-0.74	-0.23	-0.34	-2.42	-2.63	0.58	0.80
BMAA1232	hypothetical protein	-1.16	-0.33	-2.60	-2.15	-0.74	-0.93	-0.98	-0.62	-2.68	-2.44	0.59	NaN
BMA2841	membrane protein, putative	-2.77	-2.69	-1.70	-1.67	-2.39	-2.92	-1.82	-1.43	-3.98	-3.63	0.87	0.22
BMA1555	ribosomal protein S2	-4.04	-3.32	-1.57	-1.95	-2.97	-2.50	-1.77	-1.42	-2.28	-1.92	1.00	0.55
BMA1355	lipoprotein NlpD, putative	-2.02	-1.74	-1.04	-1.07	-1.06	-1.34	-0.74	-0.33	-0.45	-1.00	0.86	0.62
BMA1736	DNA-binding response regulator NarL	-1.85	-2.39	-0.93	NaN	-0.57	-1.23	-0.01	-0.05	NaN	0.02	0.84	NaN
BMAA0424	membrane protein, putative	-2.23	-1.28	-2.74	-1.16	-2.99	-1.21	-1.14	-0.91	-2.41	-1.24	0.89	0.83
BMAA1092	GTP cyclohydrolase I	-1.70	-0.56	-2.01	NaN	-1.24	-0.04	-0.50	-0.53	NaN	-0.83	0.91	0.91
BMA1708	cysteine desulfurase	-1.04	-1.25	-0.76	-1.75	-1.15	-0.82	-1.20	-0.92	-1.95	-1.95	0.42	0.68
BMA0189	2-polyprenylphenol 6-hydroxylase	-1.85	-1.93	-0.36	-0.94	-0.74	-1.45	-0.26	-0.74	-0.41	-0.73	0.42	0.63
BMA3005	site-specific recombinase, phage integrase family	NaN	NaN	NaN	-1.50	-2.33	-2.99	-2.25	-2.23	NaN	-2.15	NaN	NaN
BMA0533	acyl carrier protein	-4.18	-4.21	-3.32	-4.15	-4.79	-4.41	-3.70	-3.11	-3.89	-3.96	0.47	0.54
BMA1303	conserved hypothetical protein	NaN	-1.49	NaN	NaN	-1.22	-1.70	-0.51	-1.16	NaN	-1.21	0.53	0.54
BMAA0802	isovaleryl-CoA dehydrogenase	-1.67	-2.23	-3.05	-2.98	-1.29	-1.87	-2.25	-2.25	-0.42	-0.51	0.55	0.57
BMA3195	cytochrome c oxidase assembly protein ctaG, putative	-1.73	-2.29	-0.91	-2.88	-1.21	-2.06	-1.59	-1.99	-0.55	-1.36	0.53	0.35
BMAA1496	amino acid ABC transporter, periplasmic amino acid-binding protein	-1.10	-0.58	-1.00	-1.16	-1.48	-1.64	-0.65	-0.78	-1.37	-1.58	0.67	0.39
BMAA0339	RNA polymerase sigma factor RpoD	-2.34	-2.96	-1.37	-2.24	-2.04	-2.94	-2.25	-2.38	-2.26	-2.51	1.04	1.56
BMA2001	chaperonin, 60 kDa	-0.90	-3.26	-1.80	-0.78	-1.52	-0.82	-2.09	-1.43	-1.82	-1.67	1.42	1.28
BMA0097	oxidoreductase, short chain dehydrogenase/reductase family	-1.59	-1.48	-1.94	-0.50	-0.84	-0.75	0.27	0.04	-0.14	-0.06	1.44	1.33
BMA2536	conserved hypothetical protein	NaN	-1.42	NaN	NaN	NaN	-1.89	-1.61	-1.14	NaN	-1.43	NaN	NaN
BMA2633	ribosomal protein S10	-2.58	-2.80	NaN	-2.73	-2.66	-3.31	-2.20	-2.19	-2.80	-2.95	1.20	0.96
BMAA1745	conserved hypothetical protein	-2.25	-2.73	-1.38	-1.50	-1.91	-2.16	-0.96	-0.47	-1.72	-0.89	1.33	1.05
BMA2303	glycosyl transferase, group 1 family protein	-0.83	-1.66	-1.36	-2.14	-1.60	-2.35	-2.19	-2.27	-1.76	-1.56	-2.61	-2.76
BMA0497	site-specific recombinase, phage integrase family, truncation	-2.56	-2.50	-2.36	-3.14	-2.28	-2.48	-2.77	-2.49	-3.74	-2.76	-2.57	-2.90
BMA2884	hypothetical protein	-0.84	-0.78	0.15	-0.78	-2.64	-2.61	-2.54	-2.50	NaN	-0.63	-2.62	-2.93
BMA2523	ribosomal protein L21	-5.59	-5.31	-3.46	-4.43	-5.09	-4.71	-3.79	-3.27	-5.03	-4.39	-2.77	-2.99
BMA2647	preprotein translocase, SecE subunit	-3.79	-2.65	NaN	-4.07	-4.50	-3.84	-3.25	-2.98	-4.24	-3.54	-2.57	-3.09
BMA2952	ATP synthase F0, C subunit	-3.84	-4.39	-3.43	-3.34	-4.10	-4.11	-3.21	-2.89	-4.18	-3.98	-2.75	-3.20
BMAA0727	GTP cyclohydrolase I	NaN	-1.06	NaN	NaN	NaN	-1.23	-2.04	-1.60	-3.43	-2.88	-2.20	-2.06
BMAA1286	outer membrane porin, putative	-2.60	-1.92	-2.48	-3.21	-3.21	-3.36	-2.16	-1.94	-3.28	-2.93	-2.14	-2.06

BMA1829	NADH dehydrogenase I, A subunit	-3.64	-3.10	-2.91	-2.64	-3.21	-2.61	-2.39	-2.26	-3.30	-2.63	-1.88	-2.15
BMA2302	capsular polysaccharide biosynthesis protein, putative	-1.31	-0.90	-0.08	-2.05	-1.71	-2.13	-2.42	-2.32	-2.03	-1.46	-1.84	-2.19
BMA1319	polyhydroxyalkanoate synthesis repressor PhaR	-3.70	-2.59	-3.51	-3.28	-3.63	-3.11	-2.33	-2.06	-3.54	-2.71	-1.97	-2.23
BMA1414	phosphate starvation-inducible protein	-3.21	-3.20	-3.70	-3.70	-4.15	-3.57	-2.02	-2.30	-3.08	-2.50	-1.94	-2.26
BMA2082	outer membrane protein, OmpA family	-4.05	-3.16	-3.28	-3.56	-4.03	-3.48	-2.78	-2.24	-4.04	-3.69	-1.95	-2.17
BMA2607	ribosomal protein S4	-2.49	-3.63	-1.04	-2.79	-3.23	-3.99	-2.78	-2.80	-3.58	-3.03	-1.91	-2.20
BMA2956	ATP synthase F1, gamma subunit	-3.21	-2.90	-2.01	-3.71	-4.25	-4.25	-3.21	-3.03	-3.57	-3.04	-1.92	-2.06
BMA2918	transcriptional regulator, MarR family	-3.01	-2.86	-2.52	-3.59	-3.94	-3.91	-2.48	-2.52	-2.91	-2.87	-1.99	-2.05
BMAA0017	D-beta-hydroxybutyrate dehydrogenase	-2.23	-2.63	-2.25	-2.44	-2.66	-2.55	-2.13	-2.22	-2.66	-2.26	-2.04	-2.04
BMA1512	ISBma1, transposase	-1.42	-1.39	-1.81	-2.17	-2.50	-2.53	-2.16	-1.99	-2.32	-1.98	-2.05	-2.12
BMA1835	ribosomal protein S15	-2.82	-3.01	-2.36	-2.18	-3.08	-2.83	-2.21	-1.89	-2.94	-1.96	-2.04	-2.10
BMA1843	2-isopropylmalate synthase, authentic frameshift	-2.45	-2.59	-1.29	-2.47	-2.61	-2.61	-2.08	-1.95	-2.27	-2.02	-2.01	-2.14
BMAA0445	Rhs element Vgr protein	NaN	NaN	NaN	NaN	-1.29	-0.74	-1.92	-1.64	-1.48	-1.36	-1.99	-2.13
BMA2618	ribosomal protein S8	-5.00	-4.30	NaN	-3.99	-4.64	-4.84	-3.23	-3.28	-4.63	-3.95	-1.87	-2.30
BMA1333	adenylosuccinate synthetase	-2.97	-2.01	-2.41	-2.97	-3.06	-2.89	-2.00	-1.38	-3.17	-2.59	-1.83	-2.37
BMA2957	ATP synthase F1, beta subunit	-2.62	-2.50	-1.96	-3.03	-2.97	-3.15	-2.53	-2.88	-3.69	-2.42	-1.87	-2.37
BMA2621	ribosomal protein L24	-2.99	-3.70	-0.97	-3.04	-3.76	-4.12	-3.07	-3.36	-3.91	-3.13	-2.03	-2.42
BMAA1350	CBS domain protein	-3.04	-2.21	-2.69	-2.55	-2.72	-2.58	-1.77	-1.60	-3.31	-2.60	-1.95	-2.44
BMA0742	nitrogen regulatory protein P-II	-1.81	-1.78	NaN	-2.38	-2.02	-2.20	-1.46	-1.29	-2.30	-2.28	-2.03	-2.30
BMAA1749	succinate dehydrogenase, cytochrome b556 subunit	-2.81	-3.82	-3.03	-2.79	-3.67	-3.83	-2.21	NaN	-2.03	-2.16	-2.07	-2.24
BMA0376	conserved hypothetical protein	-1.94	-1.77	-1.40	-1.60	-1.98	-1.72	-1.36	-0.70	-2.24	-1.65	-2.50	-2.48
BMA2630	ribosomal protein L23	-3.59	-3.40	NaN	-3.00	-3.90	-3.92	-2.71	-2.94	-3.23	-1.49	-2.44	-2.46
BMA2689	conserved hypothetical protein	-2.58	-2.46	-2.39	-2.58	-2.52	-2.15	-1.51	-1.81	-2.40	-1.72	-2.43	-2.45
BMAA0081	conserved domain protein	-1.12	-0.24	-2.06	-1.42	-1.97	-1.69	-2.11	-0.57	-1.94	-1.34	-2.43	-2.44
BMAA1646	peptide synthetase, putative	-0.32	-0.19	-0.33	-1.73	0.08	-0.35	-1.81	-1.29	-1.41	-1.22	-2.43	-2.25
BMA2306	capsular polysaccharide export inner-membrane protein	-1.34	-2.01	-1.14	-2.10	-2.78	-2.63	-2.96	-2.42	-2.51	-2.16	-2.22	-2.30
BMA1984	O-antigen acetylase, putative	-1.61	-0.90	-0.76	-1.70	-2.74	-2.35	-2.69	-1.98	-2.23	-1.80	-2.23	-2.40
BMAA1261	bacterial extracellular solute-binding protein, family 5	-1.61	-1.41	NaN	-1.09	-1.50	-1.87	-1.06	-0.79	-1.07	-1.31	-2.23	-2.42
BMA0436	OmpA family protein	-3.18	-4.05	-2.32	-2.66	-3.63	-4.34	-3.57	-2.65	-3.91	-3.09	-2.10	-2.50
BMA2975	transcriptional regulator, merR family	-1.69	-1.71	-2.10	-1.73	-1.21	-1.32	-0.32	-0.99	-1.88	-1.30	-2.11	-2.56
BMAA1514	hypothetical protein	-1.28	-0.84	NaN	NaN	NaN	-0.84	-1.80	-1.22	-1.72	-1.25	-2.24	-2.54
BMA1327	conserved hypothetical protein	-4.71	-2.87	-4.47	-4.31	-4.06	-4.06	-2.59	-2.66	-4.99	-3.61	-2.20	-2.65
BMAA1798	bacterioferritin	-4.81	-3.39	-4.44	-3.77	-5.23	-3.91	-2.92	-2.28	-3.00	-2.25	-2.18	-2.65
BMAA0548	hypothetical protein	-5.60	-6.36	-3.13	-6.71	-6.92	-6.74	-6.67	-5.39	-6.71	-5.10	0.47	-1.44
BMAA0309	AMP nucleosidase	-1.36	-1.46	-1.65	-2.43	-1.44	-0.76	-1.92	-0.56	-1.90	-2.16	0.12	-0.85
BMA2097	conserved hypothetical protein TIGR00255	-2.74	-2.44	NaN	-1.75	-1.69	-2.02	-1.38	-1.14	NaN	-1.23	0.13	-0.38
BMA0704	conserved hypothetical protein	-3.28	-2.48	-2.91	-2.81	-1.96	-1.76	-0.78	-0.46	-2.55	-2.07	0.05	-0.46
BMA2703	twin-arginine translocation protein, TatA/E family	-3.26	-2.50	-3.49	-3.04	-3.10	-3.27	-1.97	-2.21	-2.53	-2.90	0.00	-0.38
BMA1485	DNA-binding response regulator RisA	-2.87	-2.24	-2.28	-2.27	-1.87	-1.32	-0.61	-0.61	-1.68	-1.44	0.29	-0.50
BMA0626	conserved hypothetical protein	-1.09	-1.34	NaN	NaN	-1.75	-1.88	-0.35	-0.55	NaN	0.03	0.10	-0.56
BMA2624	ribosomal protein L29	-3.14	-2.99	NaN	-2.32	-3.04	-3.15	-1.72	-1.39	-2.07	-2.42	0.21	-0.54
BMAA0699	hypothetical protein	-2.53	-1.86	-2.50	-0.90	-2.10	-1.76	-0.62	-0.45	NaN	-1.31	0.19	-0.62
BMA0390	conserved hypothetical protein	-1.74	-1.30	-0.64	-1.26	-0.97	-1.56	-0.24	-0.77	-1.29	-1.54	0.08	0.31
BMA1365	transcription termination factor Rho	-1.15	-2.34	-0.72	-1.72	-2.05	-2.01	-1.20	-1.72	-2.25	-1.95	0.08	0.25
BMA1090	integration host factor, alpha subunit	-0.82	-1.27	-0.51	-1.02	-1.35	-1.93	-1.01	-1.53	-1.01	-1.11	-0.05	0.30
BMA1348	nucleoside diphosphate kinase	-3.58	-3.04	-2.29	-2.73	-3.38	-3.35	-2.72	-2.30	-3.31	-2.58	-0.11	NaN
BMA0083	conserved hypothetical protein	-1.25	-1.24	-1.71	-1.38	-1.30	-1.39	-0.92	-1.43	-1.65	-1.52	-0.11	0.34
BMA1545	UDP-3-O-3-hydroxymyristoyl glucosamine N-acyltransferase	-1.79	-1.34	NaN	-1.57	-1.31	-1.95	-0.55	-0.79	NaN	-1.20	NaN	NaN
BMA2083	conserved hypothetical protein	-2.74	-3.07	-2.22	-1.98	-2.31	-2.88	-2.04	-2.14	-2.52	-3.07	-0.03	0.46

BMA0377	ribosomal protein S20	-2.55	-2.81	NaN	-2.13	-2.64	-2.67	-1.91	-0.78	-2.92	-2.33	0.26	0.54
BMA2640	DNA-directed RNA polymerase, beta subunit	-1.56	-2.07	-0.18	-2.23	-2.24	-2.74	-1.90	-1.67	-2.47	-2.79	0.21	0.46
BMAA0998	hypothetical protein	-0.74	-1.03	-2.01	-2.91	-0.45	-1.14	-1.47	-1.20	-0.02	-0.72	0.16	0.44
BMA0292	conserved hypothetical protein	-2.47	-2.22	NaN	-1.79	-1.88	-2.58	-1.11	-1.05	NaN	-1.57	-0.46	NaN
BMAA1046	RND efflux system, outer membrane lipoprotein, NodT family	-1.04	-1.08	NaN	-2.69	-1.38	-1.91	-1.23	-1.09	-2.43	-2.19	NaN	0.70
BMA2610	ribosomal protein L36	-4.27	-3.37	-2.39	-3.17	-3.92	-3.86	-2.38	-1.76	-3.01	-2.86	0.40	-0.13
BMA0864	H-NS histone family protein	-1.43	-1.35	NaN	NaN	-1.54	-1.92	-0.06	-0.05	NaN	NaN	0.53	-0.54
BMA0188	conserved hypothetical protein	-2.03	-1.74	-1.36	NaN	-1.43	-1.77	-1.10	-0.94	NaN	-1.27	0.51	NaN
BMA2905	H-NS histone family protein	-2.33	-1.09	-2.20	-3.12	-1.95	-2.63	-2.15	-1.89	-1.93	-1.97	NaN	NaN
BMA1145	arginine deiminase	-4.18	-3.10	-3.70	-0.36	-1.63	-1.33	0.02	-0.22	-0.02	-0.08	0.20	0.10
BMAA1189	transcriptional regulator, AraC family	-0.51	-0.15	-2.03	0.18	-2.65	-0.96	-0.79	0.17	-1.62	-0.59	0.18	0.08
BMAA1315	hypothetical protein	-1.77	-1.46	-2.20	-3.41	-0.97	-1.21	-1.31	-1.07	NaN	-0.81	0.21	0.17
BMA0375	ornithine carbamoyltransferase	-2.51	-2.82	-2.26	-2.33	-1.66	-1.99	-0.88	-1.60	-1.98	-2.32	0.26	0.34
BMA1530	TGS domain protein	NaN	-1.74	NaN	NaN	-1.01	-0.96	NaN	-0.31	NaN	-1.46	0.28	0.29
BMA2471	glyoxalase family protein	NaN	-1.66	NaN	NaN	-0.47	-1.65	-1.16	-1.26	NaN	-1.27	0.24	0.25
BMA2728	thiamin biosynthesis ThiG	-2.31	-2.22	-2.18	-1.42	-1.92	-2.07	-0.75	-1.06	-1.38	-2.18	0.23	0.25
BMA2550	UDP-N-acetylmuramate--alanine ligase	-2.24	-2.15	NaN	-1.79	-1.55	-1.60	-1.08	-0.93	NaN	-1.26	0.32	NaN
BMA0546	pyridoxal phosphate biosynthetic protein PdxJ	-1.11	-1.21	-1.39	-1.46	-1.22	-1.77	-1.15	-1.05	NaN	-0.20	0.35	NaN
BMAA0899	cold-shock domain family protein	-2.71	-2.00	-2.44	-3.07	-3.43	-3.30	-2.66	-2.38	-3.38	-3.38	0.35	0.05
BMA2558	cell division protein FtsL, putative	NaN	-1.83	NaN	-1.02	-1.56	-2.21	-1.28	-0.28	-0.60	-1.54	0.37	NaN
BMAA1318	hypothetical protein	-1.61	-1.44	-1.74	-1.33	-0.15	-0.81	-0.55	-0.30	NaN	-0.24	0.39	0.35
BMAA0302	hypothetical protein	-5.55	-4.59	-5.17	-3.57	-5.54	-3.34	-1.94	-1.64	-3.61	-3.20	0.55	0.13
BMA3120	ribose-phosphate pyrophosphokinase	-2.01	-1.51	NaN	-1.90	-2.66	-2.62	-1.82	-1.48	-1.76	-1.53	0.45	0.11
BMAA0505	glutamyl-tRNA reductase	-2.68	-2.38	-1.46	-2.33	-1.74	-2.02	-1.01	-0.83	-0.90	-0.94	0.42	0.20
BMAA1555	hypothetical protein	-2.87	-1.32	-1.70	-2.35	NaN	-0.92	-1.07	-0.42	-2.49	-1.90	0.43	0.16
BMA0976	conserved hypothetical protein	-3.56	-3.55	-3.54	-3.83	NaN	-0.20	-2.80	-2.42	NaN	-0.01	-0.29	-0.09
BMA1985	polysaccharide ABC transporter, ATP-binding protein	-0.85	-0.31	-0.03	-1.27	-1.41	-2.04	-1.95	-1.63	-1.30	-1.52	-0.29	-0.03
BMA2722	conserved hypothetical protein	-1.06	-1.58	-1.06	-1.29	-1.19	-1.88	-1.06	-1.20	-1.36	-1.10	-0.22	0.00
BMA1703	ferredoxin, 2Fe-2S	NaN	-1.29	NaN	-3.01	-1.56	-1.85	-1.73	-1.46	-2.03	-2.76	-0.29	-0.27
BMA2446	6-phosphogluconate dehydratase	-1.29	-0.88	-1.91	-1.49	-1.67	-2.06	-1.13	-0.82	-1.38	-0.81	-0.31	-0.18
BMA1417	glycosyl transferase, group 1 family protein	NaN	-1.40	NaN	-1.91	-1.34	-1.85	-1.64	-1.35	NaN	-1.29	-0.20	-0.30
BMA0373	conserved hypothetical protein	-3.43	-3.32	-2.78	-2.28	-2.96	-2.92	-1.69	-1.47	-2.28	-2.24	-0.14	-0.24
BMA0619	cyclic nucleotide-binding domain protein	-1.62	-2.12	-1.57	0.10	-1.45	-2.25	-0.55	-0.90	-0.22	-0.11	-0.17	-0.26
BMA1440	conserved hypothetical protein	NaN	-1.07	NaN	-2.00	NaN	-0.16	-0.88	-1.12	NaN	NaN	-0.17	NaN
BMA1404	ribosomal protein S6	-2.62	-3.56	-0.59	-2.64	-3.26	-3.50	-2.32	-2.36	-3.29	-2.63	-0.24	-0.37
BMA2002	chaperonin, 10 kDa	-1.06	-0.71	-0.96	-2.37	-3.62	-2.19	-2.41	-1.77	-3.98	-2.64	-0.23	NaN
BMA2994	glycine cleavage system H protein	-3.92	-3.59	-3.59	-2.67	-2.01	-2.12	-2.02	-1.80	-2.39	-2.26	-0.25	-0.43
BMA1364	thioredoxin	-2.49	-0.81	-1.70	-2.32	-2.36	-2.28	-1.75	-1.63	-1.99	-1.97	-0.26	-0.41
BMA3219	conserved hypothetical protein	-2.31	-1.80	-2.23	-1.53	-1.79	-2.63	-1.20	-1.29	-0.83	-1.37	-0.26	-0.40
BMA2281	ATP-dependent Clp protease, ATP-binding subunit ClpA	-2.92	-2.64	-3.45	-2.98	-2.42	-2.29	-2.25	-1.72	-2.96	-2.27	-0.14	-0.41
BMAA0896	ribosomal protein S21	NaN	NaN	NaN	NaN	-1.41	-1.02	-0.70	-0.51	-2.41	-2.00	-0.18	-0.45
BMA0337	maltose/mannitol ABC transporter, ATP-binding protein, putative	-0.78	-0.77	-1.80	-3.27	-1.57	-1.70	-2.03	-2.32	-2.07	-2.14	-0.10	0.00
BMA1489	2C-methyl-D-erythritol 2,4-cyclodiphosphate synthase	-1.32	-1.18	-1.13	-1.77	-1.36	-1.78	-1.12	-0.52	-1.47	-1.70	-0.10	0.11
BMA0400	ribosomal protein L19	-2.55	-3.12	-0.79	-2.80	-3.80	-3.66	-2.77	-2.20	-2.65	-1.89	0.06	0.07
BMA1498	PspA/IM30 family protein	-1.42	-1.23	-1.20	-0.83	-0.87	-1.19	-0.72	-1.03	-2.78	-2.07	0.07	0.05
BMA1660	UDP-2,3-diacylglucosamine hydrolase	-1.15	-1.35	-0.35	-1.16	-0.33	-0.91	NaN	-1.01	NaN	-0.99	-0.01	0.10
BMA0699	ParA family protein	-1.57	-1.52	-1.69	-1.97	-1.84	-2.03	-0.97	-0.81	-1.81	-1.68	-0.02	0.02
BMA0673	DNA polymerase III, chi subunit, putative	-1.52	-0.96	NaN	-1.34	-1.84	-1.22	-0.72	-0.52	NaN	-1.04	-0.03	0.03
BMA1996	conserved hypothetical protein TIGR00250	NaN	-2.29	-1.78	NaN	NaN	-1.35	-1.12	-1.14	NaN	NaN	-0.04	NaN

BMA1649	hypothetical protein	-1.44	-0.85	-0.79	-1.66	-1.04	-0.89	-0.48	-0.52	-1.92	-0.58	0.03	0.00
BMA1702	conserved hypothetical protein	NaN	-0.61	NaN	-1.58	-0.73	-1.40	-1.30	-1.12	NaN	-1.67	0.02	NaN
BMA2174	rhodanese-like domain protein	-1.52	-1.49	NaN	-1.69	-1.96	-2.01	-0.98	-1.80	NaN	-1.31	0.02	NaN
BMA2474	barstar family protein	-1.78	-1.67	-0.90	-1.81	-1.63	-2.36	-1.45	-1.24	-2.03	-2.31	0.01	0.00
BMA1533	SPFH domain/band 7 family protein	-2.03	-2.30	-2.78	-1.61	-3.24	-2.31	-1.72	-1.23	-3.19	-2.03	-0.05	-0.17
BMA2229	peptidyl-prolyl cis-trans isomerase, FKBP-type	-3.34	-3.25	-2.64	-2.40	-2.38	-2.57	-1.38	-1.40	-2.26	-2.08	-0.03	-0.15
BMA2181	urease accessory protein UreD	-1.29	-0.41	-1.95	-0.79	-1.46	-0.19	-0.91	-0.42	-1.66	-0.54	0.07	-0.23
BMA2609	ribosomal protein S13	-3.41	-2.65	-2.64	-2.77	-3.62	-2.87	-2.66	-2.36	-3.38	-2.88	0.10	-0.25
BMA2616	ribosomal protein L18	-3.14	-2.84	NaN	-2.54	-3.18	-3.00	-1.88	-2.16	-2.85	-2.32	0.04	-0.28
BMA3379	glucosamine--fructose-6-phosphate aminotransferase, isomerizing	-1.77	-1.24	NaN	-1.38	-1.34	-1.79	-0.87	-0.61	NaN	-1.18	0.04	NaN
BMA2074	conserved hypothetical protein TIGR00244	-1.00	-1.41	0.08	-1.16	-0.95	-1.59	-0.98	-1.07	-1.55	-2.19	0.11	-0.11
BMA2518	pyrophosphatase, MutT/nudix family	-1.89	-2.13	-1.04	-2.89	-1.46	-1.96	-2.20	-1.92	-2.17	-2.14	0.17	-0.12
BMAA0361	hypothetical protein	-1.85	-1.37	-1.97	-1.89	-1.67	-1.59	-0.53	-0.43	-1.70	-1.58	0.12	-0.04
BMAA1592	hipB domain protein	-1.64	-1.47	-0.46	NaN	-1.24	-1.35	-0.01	-0.13	NaN	-1.03	0.15	-0.06
BMAA2098	glutamine ABC transporter, periplasmic glutamine-binding protein	-2.39	-2.22	-2.27	-3.00	-2.90	-3.28	-1.32	-0.74	-3.04	-2.69	-0.67	-0.02
BMA1857	transcriptional reguator, TetR family	-2.83	-1.69	-3.07	-2.44	-2.36	-2.32	-1.27	-0.77	-2.72	-2.63	-1.03	-0.35
BMA1522	GMP synthase	-1.58	-1.91	-0.80	-1.16	-1.20	-1.73	-0.33	-0.32	-1.31	-1.13	-1.18	-0.86
BMA0002	DNA polymerase III, beta subunit	-2.63	-2.74	-2.14	-2.28	-2.25	-2.64	-2.03	-1.72	-2.40	-2.18	-1.18	-0.96
BMA0792	hypothetical protein	-2.72	-1.30	-3.58	NaN	-1.33	-1.02	NaN	-0.08	NaN	NaN	NaN	NaN
BMA2629	ribosomal protein L2	-2.17	-2.70	-0.54	-2.76	-2.63	-2.79	-2.66	-2.94	-2.31	-2.72	-1.11	-0.98
BMA2066	antioxidant, AhpC/Tsa family	-4.98	-4.45	-3.88	-4.95	-4.35	-4.01	-3.76	-3.50	-5.06	-4.77	-1.06	-0.98
BMA2380	cytochrome b561, putative	-1.27	-0.47	NaN	-0.57	-0.83	-1.25	-1.03	-0.35	NaN	-1.13	-1.07	-0.95
BMA1819	NADH dehydrogenase I, K subunit	-3.31	-3.40	NaN	-1.74	-3.59	-3.62	-2.68	-2.27	NaN	-1.85	-1.07	NaN
BMAA0292	tautomerase enzyme family protein	NaN	-1.47	NaN	-1.65	NaN	-1.32	-0.45	-0.46	NaN	NaN	-1.07	-0.96
BMA1602	NLP/P60 family protein	-1.97	-2.51	-2.14	-2.15	-2.56	-3.01	-2.05	-2.04	-3.53	-2.89	-0.94	-0.88
BMA1340	hfq protein	-3.41	-1.31	-2.45	-2.37	-3.55	-2.73	-1.79	-1.08	-2.25	-2.47	-0.97	-0.83
BMA1847	acetolactate synthase, small subunit	-2.31	-2.38	-1.34	-2.58	-2.04	-1.97	-1.75	-1.16	-2.98	-2.21	-0.99	-0.86
BMA2280	ATP-dependent Clp protease adaptor protein ClpS	-2.79	-2.34	-2.44	-2.67	-2.51	-2.05	-1.94	-1.59	-2.45	-2.11	-1.01	-0.90
BMAA1490	beta-ketoadipyl CoA thiolase	-1.43	-1.47	NaN	-1.42	-0.30	-0.75	-0.93	-0.97	NaN	-0.61	-1.02	-0.88
BMA2642	ribosomal protein L7/L12	-1.32	-1.87	-0.09	-2.33	-2.50	-2.97	-2.47	-2.55	-2.51	-2.15	-1.15	-0.67
BMAA1744	citrate synthase I	-2.19	-3.37	-1.99	-2.52	-3.30	-3.69	-2.05	-2.59	-2.67	-2.43	-1.15	-0.76
BMA3342	alkylphosphonate utilization operon protein PhnA, putative	-1.47	-1.92	-0.75	-1.40	-1.28	-1.42	-0.78	-1.12	-0.86	-1.18	-0.59	-0.78
BMA0926	UTP-glucose-1-phosphate uridylyltransferase	-3.01	-3.18	-2.82	-2.80	-2.89	-2.56	-2.40	-2.02	-3.41	-2.70	-0.66	-0.80
BMA1300	putrescine ABC transporter, ATP-binding protein	-1.44	-1.50	-1.62	-1.43	-1.68	-2.31	-1.69	-1.70	-2.17	-2.11	-0.70	-0.82
BMA2442	adenylosuccinate lyase	-2.08	-2.48	-0.85	-1.97	-1.88	-2.47	-1.43	-1.48	-1.69	-1.85	-0.69	-0.80
BMA3370	LemA family protein	-2.48	-2.44	-2.90	NaN	-1.95	-2.27	-0.72	-0.98	NaN	-0.31	-0.67	-0.76
BMA2604	ribosomal protein L17	-2.19	-2.81	-0.46	-2.19	-3.72	-3.58	-2.72	-2.04	-2.81	-2.36	-0.75	-0.84
BMA2619	ribosomal protein S14	-3.85	-3.72	NaN	-3.33	-3.92	-4.20	-3.01	-2.87	-3.87	-3.48	-0.70	-0.88
BMA3213	PTS system, fructose-specific IIA component	-1.95	-2.16	-1.04	-2.33	-1.52	-1.96	-1.79	-1.68	-1.42	-1.80	-0.76	-0.96
BMA1656	cysteinyl-tRNA synthetase	-3.25	-2.10	-2.18	-2.86	-2.52	-2.16	-2.07	-1.36	-2.67	-2.27	-0.80	-0.91
BMA1301	putrescine ABC transporter, periplasmic putrescine-binding protein	-1.58	-1.34	-1.24	-1.88	-2.55	-2.58	-1.45	-1.73	-3.42	-3.34	-0.85	-0.91
BMA1402	ribosomal protein S18	-3.46	-3.23	-1.19	-2.90	-3.63	-3.29	-2.37	-1.93	-4.14	-3.10	-0.86	-0.92
BMA0625	conserved hypothetical protein	-1.27	-1.60	-0.88	NaN	-1.97	-2.48	-0.25	-0.42	NaN	0.09	-0.86	-0.90
BMA2958	ATP synthase F1, epsilon subunit	-1.86	-1.96	-0.25	-2.26	-3.14	-3.17	-2.28	-2.46	-2.05	-2.48	-0.84	-0.90
BMAA1462	transcriptional regulator, GntR family	-1.40	-1.18	-1.84	-2.24	-1.52	-1.42	-1.36	-1.16	-1.19	-0.97	-0.87	-0.88
BMA0536	RNA polymerase sigma-H factor	-2.75	-2.25	-2.44	-2.26	-2.32	-2.11	-1.66	-1.54	-2.40	-2.51	-0.93	-0.77
BMA1883	uracil phosphoribosyltransferase	-1.92	-1.58	-2.79	-1.58	-1.92	-2.20	-0.97	-0.38	-1.65	-1.48	-0.88	-0.73
BMA0672	cytosol aminopeptidase	-1.08	-0.63	NaN	-1.40	-1.64	-1.51	-0.63	-0.65	-0.29	-1.57	-0.78	-0.75
BMA1721	pyruvate dehydrogenase, E1 component	-3.30	-2.75	-1.18	-1.64	-2.27	-2.11	-1.21	-1.25	-2.02	-2.16	-0.80	-0.72

BMA2606	dna-directed rna polymerase alpha chain	-1.63	-2.70	-0.84	-2.19	-2.80	-3.32	-2.10	-3.11	-2.69	-2.40	-0.81	-0.73
BMA2382	YceI-like family protein	-3.03	-2.56	-2.64	-3.01	-4.00	-3.75	-2.67	-2.03	-3.78	-3.14	-0.85	-0.75
BMA2955	ATP synthase F1, alpha subunit	-1.08	-2.06	-0.48	-1.44	-1.81	-2.18	-2.70	-2.66	-2.20	-1.15	-0.83	-0.78
BMA3090	thiamine biosynthesis protein ThiC	-1.34	-1.25	-1.27	-1.48	-1.83	-2.13	-0.98	-1.08	-2.32	-2.16	-0.82	-0.82
BMA3252	response regulator	-2.04	-1.87	-2.36	-1.93	-1.48	-1.72	-1.31	-1.19	-1.11	-1.17	-0.79	-0.63
BMA0467	5-methyltetrahydropteroyltriglutamate--homocysteine S-methyltransferase	NaN	NaN	NaN	NaN	NaN	-0.51	-1.90	-2.23	-4.65	-4.16	-0.81	-0.61
BMAA0303	organic hydroperoxide resistance protein	-0.84	-1.02	-0.06	-3.16	-0.97	-1.11	-0.91	-1.18	-0.71	-1.30	-0.82	-0.63
BMA1433	conserved hypothetical protein	-2.07	-1.59	-1.65	-2.38	-2.35	-2.34	-0.98	-0.70	-1.81	-1.76	-0.69	-0.68
BMA0435	DNA gyrase, A subunit	-2.66	-2.83	-0.98	-2.22	-2.22	-2.53	-2.08	-1.98	-2.15	-1.70	-0.75	-0.70
BMA3263	hypothetical protein	-2.24	-2.17	-1.90	-0.43	-1.34	-1.66	-0.40	-0.65	NaN	-0.93	-0.73	-0.68
BMA0486	isocitrate dehydrogenase, NADP-dependent	-2.79	-2.02	-2.83	-2.95	-3.26	-2.45	-2.19	-1.84	-3.77	-2.91	-0.75	-0.58
BMA2271	superoxide dismutase	-2.96	-2.86	-3.47	-2.41	-2.98	-2.65	-2.65	-2.14	-3.12	-2.15	-0.68	-0.60
BMA3255	DnaK suppressor protein	-3.73	-3.47	-3.53	-3.90	-3.60	-3.77	-2.43	-1.84	-3.11	-3.41	-0.70	-0.60
BMA2544	AhpC/TSA family protein	-1.73	-2.04	-2.25	-2.19	-2.34	-2.31	-1.11	-1.19	-1.81	-1.58	-0.64	-0.63
BMAA0660	amino acid ABC transporter, periplasmic amino acid-binding protein, putative	-1.70	-1.56	-2.60	-1.96	-1.92	-2.17	-1.16	-1.06	-1.44	-1.75	-0.63	-0.59
BMAA1794	acetyl-coenzyme A synthetase	-3.19	-2.76	-2.98	-2.87	-1.71	-2.17	-1.44	-1.36	NaN	-2.45	-0.62	NaN
BMA3205	protein-export protein SecB	-3.03	-2.74	-2.18	-2.83	-3.26	-3.30	-1.88	-2.22	-2.37	-2.23	-0.77	-0.50
BMA2632	ribosomal protein L3	-2.73	-2.10	-0.51	-2.00	-2.69	-2.66	-2.55	-2.94	-2.72	-2.23	-0.73	-0.42
BMAA0318	decarboxylase family protein	-1.50	-1.99	-1.37	-1.74	-1.62	-2.28	-1.57	-1.70	-1.61	-1.45	-0.70	-0.40
BMA0550	translation elongation factor P	-3.45	-2.98	-1.79	-1.87	-2.30	-3.19	-2.04	-1.46	-1.34	-1.37	-0.52	-0.23
BMA2791	cold-shock domain family protein	-2.65	-2.94	-2.44	-3.32	-2.95	-3.45	-2.86	-0.59	-3.42	-3.45	-0.53	-0.21
BMA0469	fructose-1,6-bisphosphatase	-2.66	-2.51	-2.43	-2.00	-1.97	-2.06	-1.07	-0.92	-1.47	-1.44	-0.48	-0.34
BMA1328	lipoprotein, putative	-2.63	-2.46	-2.24	-2.28	-1.95	-1.93	-1.65	-1.22	-2.61	-2.41	-0.42	-0.33
BMA3211	phosphoenolpyruvate-protein phosphotransferase	-2.83	-2.54	-2.41	-3.00	-2.48	-2.53	-2.15	-2.23	-2.10	-1.84	-0.42	-0.35
BMA1263	ribonuclease R	-2.46	-2.42	-2.18	-2.13	-1.52	-1.82	-1.35	-1.99	-1.08	-1.30	-0.45	-0.28
BMA2545	cell division protein FtsZ	-2.72	-2.52	-3.22	-2.79	-2.87	-2.91	-1.81	-1.62	-2.08	-1.52	-0.42	-0.26
BMA0275	succinyl-CoA synthase, beta subunit	-1.96	-1.79	-1.88	-1.67	-2.16	-2.36	-2.34	-1.50	-1.90	-1.42	-0.39	-0.20
BMA3210	HesA/MoeB/ThiF family protein	-1.71	-1.42	NaN	-1.01	-0.85	-1.40	-1.37	-1.47	NaN	NaN	-0.39	NaN
BMA0904	conserved hypothetical protein	-1.31	-0.92	-1.12	-1.50	-1.46	-1.16	-0.63	-0.30	-1.48	-1.39	-0.36	-0.30
BMA2457	outer membrane lipoprotein, putatitve	-2.15	-2.34	-1.59	-2.21	-2.69	-3.11	-1.59	-1.60	-1.88	-2.59	-0.38	-0.28
BMA3400	ribosomal protein L34	-1.75	-1.88	NaN	-1.17	-1.83	-1.80	-0.80	-0.89	-1.16	-1.63	-0.36	-0.25
BMA1745	molybdopterin-binding protein	-2.18	-1.24	NaN	-1.67	-1.45	-2.07	-0.97	-1.15	-1.79	-1.85	-0.55	-0.35
BMA0770	carbamoyl-phosphate synthase, small subunit	-2.56	-3.20	-0.72	-0.57	-1.05	-1.72	0.03	-0.31	-0.27	-0.47	-0.55	-0.32
BMAA0525	thiol:disulfide interchange protein DsbC, putative	-1.79	-1.55	-1.71	-1.73	-1.95	-2.33	-1.22	-1.50	-2.48	-2.13	-0.55	-0.31
BMA0771	homoserine/threonine efflux protein, putative	-1.78	-1.82	-0.44	-0.36	-0.54	-0.95	-0.28	-0.19	NaN	0.22	-0.54	-0.40
BMAA0537	hypothetical protein	-2.04	-1.67	NaN	-1.62	-1.64	-1.79	-1.02	-0.68	NaN	-1.79	-0.57	-0.40
BMA2417	flavohemoprotein	-3.91	-3.34	-2.51	-0.69	-2.61	-2.73	-1.44	-1.19	-0.25	-0.81	-0.53	-0.51
BMA2840	5,10-methylenetetrahydrofolate reductase	-1.93	-1.66	NaN	NaN	-1.44	-1.93	-0.62	-0.71	-2.03	-2.62	-0.56	-0.53
BMA0623	peptidase, U32 family	-3.39	-3.26	-2.71	-0.17	-3.41	-3.78	-0.13	-0.78	-0.17	NaN	-0.41	-0.52
BMAA0365	H-NS histone family protein	-2.86	-2.24	-2.95	-3.07	-3.02	-3.11	-2.32	-1.60	-2.52	-2.50	-0.38	-0.48
BMA0084	arginyl-tRNA synthetase	-1.39	-2.00	-1.97	-1.43	-1.36	-1.59	-1.55	-1.23	-1.96	-1.40	-0.47	-0.51
BMA1777	iron compound ABC transporter, periplasmic iron-compound-binding protein, putative	NaN	NaN	NaN	NaN	-0.88	-1.63	-0.85	-0.90	-1.69	-1.65	NaN	NaN
BMAA0256	conserved hypothetical protein	-2.31	-2.26	-2.46	-1.20	-1.23	-1.00	-0.86	-0.77	-1.48	-0.86	-0.47	-0.46
BMAA0996	gamma-glutamyltranspeptidase	-3.05	-2.40	-4.11	-3.40	-2.56	-2.18	-2.58	-1.85	-3.46	-2.79	-0.46	-0.42
BMA2277	adenylate kinase	-2.45	-2.25	-1.99	-2.86	-2.58	-2.75	-1.85	-1.63	-2.15	-1.99	-0.32	-0.42
BMAA1797	hydro-lyase, Fe-S type, tartrate/fumarate family	-2.06	-2.26	-1.62	-1.76	-2.18	-2.75	-1.11	-0.85	NaN	-0.21	-0.39	-0.41
BMA1749	conserved hypothetical protein	-2.01	-1.86	-0.91	-0.94	-1.89	-2.44	-0.53	-0.69	-0.67	-1.05	-0.19	-0.66
BMA1752	conserved hypothetical protein	-1.29	-1.42	-1.05	-1.75	-1.65	-1.63	-1.14	-0.34	-1.77	-1.62	-0.12	-0.65
BMA0541	GTP-binding protein LepA	-0.48	-1.77	-0.07	-1.97	-1.33	-2.03	-0.81	-1.39	-2.04	-0.59	-0.17	-0.55

BMA0741	glutamine dependent NAD+ synthetase	-1.73	-1.76	-0.87	-1.18	-1.09	-1.59	-0.68	-0.56	-1.49	-1.61	-0.13	-0.51
BMA0333	maltose/mannitol ABC transporter, periplasmic maltose/mannitol-binding protein, putative	NaN	NaN	-2.72	-2.63	-1.59	-1.87	-1.82	-1.67	-2.00	-2.02	-0.08	NaN
BMA2068	conserved hypothetical protein	-1.57	-1.90	-0.67	-1.40	-0.85	-1.38	-0.66	-0.76	-1.41	-1.62	-0.09	-0.50
BMA1554	translation elongation factor Ts	-3.86	-3.24	-1.80	-1.68	-2.69	-2.58	-1.89	-1.57	-2.28	-1.96	-0.32	-0.71
BMAA0237	hypothetical protein	-1.53	-0.99	NaN	-0.90	-1.09	-0.76	-0.97	-0.86	-1.29	-1.25	-0.26	-0.71
BMA1426	lipoprotein, putative	-2.06	-1.41	-1.45	-1.80	-1.78	-0.97	-1.45	-0.67	-1.82	-1.16	-0.29	-0.57
BMA0366	conserved hypothetical protein TIGR00150	-2.06	-2.13	NaN	-1.86	-1.56	-1.98	-0.99	-0.99	-1.29	-1.81	-0.31	-0.53
BMA3212	phosphocarrier protein HPr	-1.46	-1.06	-1.47	-1.39	-1.52	-1.38	-1.11	-0.99	-1.62	-1.34	-0.31	-0.49
BMA2717	BolA/YrbA family protein	NaN	NaN	NaN	-1.46	-1.76	-2.18	-1.12	-0.96	NaN	-1.38	-0.28	-0.48
BMA2058	conserved hypothetical protein	-0.07	NaN	NaN	-1.80	-0.18	-0.30	-1.08	-0.32	NaN	0.08	-0.27	NaN
BMAA0162	hypothetical protein	-1.75	-1.40	-2.47	NaN	-1.18	-1.48	-0.60	-0.49	NaN	-0.32	-0.26	-0.46
BMA2095	DNA-directed RNA polymerase, omega subunit	-4.40	-3.86	-2.71	-3.77	-3.33	-2.98	-2.37	-1.55	-2.91	-2.92	-0.38	-0.60
BMA1912	ABC transporter, permease/ATP-binding protein	-2.02	-1.19	-1.52	-2.17	-3.22	-2.80	-2.03	-0.82	-2.56	-1.36	-0.36	-0.58
BMA0429	cytidylate kinase	NaN	-0.66	NaN	NaN	-0.78	-1.63	-1.12	-1.00	NaN	NaN	-0.37	NaN
BMA2628	ribosomal protein S19	-4.54	-4.30	-1.16	-3.73	-4.76	-4.15	-3.29	-3.10	-4.25	-3.70	-0.37	-0.57
BMA0890	hypothetical protein	-2.44	-1.95	-2.49	-2.37	-3.08	-2.62	-1.92	-1.27	-2.60	-1.88	-0.38	-0.66
BMAA1370	oxidoreductase, aldo/keto reductase family	-1.38	-0.86	-1.74	-1.41	-0.65	-0.83	-0.66	-0.53	-1.40	-1.52	-0.41	-0.63
BMA3207	rhodanese-like domain protein	-4.15	-3.03	-4.11	-3.25	-4.75	-3.80	-3.36	-1.81	-4.30	-2.85	-0.54	-0.66
BMA3251	sensor histidine kinase	-2.71	-2.26	-2.38	-2.28	-1.90	-2.00	-0.98	-1.00	NaN	-1.21	-0.47	-0.70
BMA2440	transcriptional regulator, LysR family	-3.97	-3.56	-3.34	-3.29	-4.02	-3.85	-2.69	-2.47	-3.47	-3.11	-0.48	-0.72
BMA2623	ribosomal protein S17	-5.58	-5.16	NaN	-4.62	-5.48	-5.40	-3.95	-3.54	-4.72	-4.03	-0.49	-0.74
BMAA0329	geranyltranstransferase	-2.35	-2.18	-1.45	-2.42	-2.39	-2.35	-1.61	-1.35	-1.98	-2.02	-0.51	-0.73
BMAA0719	DNA-binding protein	-3.32	-2.84	-2.41	-3.67	-1.92	-2.63	-1.65	-1.54	-2.85	-2.87	-0.41	-0.72
BMA2913	DNA-binding protein HU, form B	-2.79	-3.25	-2.64	-3.13	-3.01	-3.38	-3.77	-2.74	-4.15	-1.76	-0.42	-0.80
BMA2612	preprotein translocase, SecY subunit	-2.34	-2.15	0.02	-2.28	-2.73	-2.95	-2.66	-2.73	-2.99	-2.63	-0.44	-0.85
BMAA2110	NADPH-dependent FMN reductase domain protein	-1.20	-1.59	-2.15	-2.16	-1.55	-1.70	-1.36	-1.46	-2.06	-1.40	-0.48	-0.88
BMA0715	Orn/Lys/Arg decarboxylase	-1.33	-1.23	NaN	-1.00	-1.97	-2.21	-0.79	-0.89	-1.50	-0.84	-1.47	-1.06
BMA1021	type-1 fimbrial protein, authentic frameshift	-2.63	-2.10	-1.04	-2.08	-2.44	-3.05	-2.13	-2.35	-2.38	-2.71	-1.66	-1.46
BMA1435	hypothetical protein	-0.36	-0.47	-0.37	-1.53	-0.29	-0.38	-0.81	-0.92	-2.32	-1.63	-1.62	-1.43
BMA1094	ribosomal protein L35	-3.45	-3.64	-2.79	-3.26	-3.69	-3.74	-3.02	-2.76	-3.68	-3.31	-1.53	-1.53
BMA0714	deoxycytidine triphosphate deaminase, putative	-2.20	-1.70	NaN	-1.94	-2.67	-3.05	-1.42	-1.69	-1.88	-2.72	-1.54	-1.57
BMAA0195	ubiquinol oxidase, subunit I	-1.85	-0.58	NaN	NaN	-1.73	-2.43	-1.21	-1.40	NaN	NaN	-1.57	-1.56
BMA1743	glutamine synthetase, type I	-1.39	-1.96	-1.49	-2.52	-2.82	-3.14	-1.80	-1.85	-2.24	-1.80	-1.49	-1.67
BMAA0773	transcriptional regulator, TetR family, putative	-2.62	-2.41	-2.58	-2.51	-2.37	-2.55	-1.50	-1.30	-2.38	-2.65	-1.54	-1.64
BMA1395	antioxidant, AhpC/Tsa family	-1.77	-1.61	-1.25	-1.58	-2.21	-2.08	-0.95	-0.73	-1.96	-1.78	-1.46	-1.61
BMA2953	ATP synthase F0, B subunit	-2.82	-2.77	-1.90	-2.66	-3.73	-3.28	-3.09	-2.56	-3.69	-2.97	-1.44	-1.66
BMA2842	adenosylhomocysteinase	-1.63	-2.42	-1.43	-2.24	-2.17	-2.26	-2.23	-2.31	-1.58	-0.96	-1.40	-1.66
BMAA1515	hypothetical protein	-0.71	-0.98	-0.02	-0.87	-0.03	-1.18	-1.45	-1.58	-1.64	-1.60	-1.42	-1.64
BMA2423	conserved hypothetical protein	-2.50	-2.13	-2.57	-2.42	-1.91	-2.09	-1.55	-1.43	-1.77	-1.62	-1.37	-1.70
BMA1037	hypothetical protein	NaN	-0.54	NaN	NaN	NaN	-0.57	-1.48	-1.05	NaN	-0.80	-1.34	-1.70
BMA2637	ribosomal protein S12	-3.66	-3.37	-2.07	-2.83	-3.57	-3.34	-2.64	-2.57	-3.81	-2.99	-1.34	-1.72
BMAA0234	hypothetical protein	-3.13	-2.56	NaN	-3.34	-2.71	-3.00	-1.87	-1.81	-1.98	-2.85	-1.31	-1.60
BMA1999	rubredoxin	-2.52	-1.05	-1.82	-1.31	-2.00	-1.51	-0.67	-0.69	-2.03	-1.92	-1.37	-1.56
BMA0528	ribosomal protein L32	-4.31	-3.82	-2.76	-2.98	-3.32	-3.42	-2.46	-2.17	-2.95	-2.84	-1.38	-1.60
BMAA1575	hypothetical protein	NaN	-1.19	-1.65	-1.47	NaN	-0.55	-0.50	-0.57	NaN	-1.46	-1.41	-1.57
BMA2202	translation initiation factor IF-1	-0.15	NaN	NaN	-0.09	0.09	-0.88	-0.91	-1.40	NaN	NaN	-1.49	-1.26
BMAA1723	tRNA pseudouridine synthase A	-0.90	-1.06	NaN	-1.44	-0.67	-1.57	-0.46	-0.32	-0.38	-1.06	-1.38	-1.24
BMA2524	octaprenyl-diphosphate synthase	-2.29	-2.43	-2.12	-2.19	-2.47	-2.48	-1.63	-2.13	-1.77	-1.89	-1.30	-1.30
BMA3113	ATP-dependent protease La domain protein	-2.60	-2.07	-2.99	-2.92	-3.03	-3.06	-2.39	-1.45	-2.64	-1.27	-1.26	-1.22

BMAA0064	conserved hypothetical protein	NaN	-0.98	NaN	NaN	NaN	-0.73	-2.23	-2.20	NaN	-1.62	-1.27	-1.24
BMA0213	lactoylglutathione lyase	-2.41	-2.50	-2.77	-3.13	-3.61	-3.33	-2.75	-1.51	-3.45	-2.13	-1.32	-1.26
BMAA1141	homoprotocatechuate degradative operon repressor	NaN	NaN	NaN	-2.59	NaN	-1.16	-1.26	-0.99	NaN	-1.61	NaN	NaN
BMA0221	magnesium and cobalt efflux protein CorC	-2.31	-2.55	-1.85	-2.15	-2.08	-2.31	-1.17	-0.71	-2.39	-2.10	-1.32	-1.17
BMAA1838	conserved hypothetical protein	NaN	-0.72	NaN	NaN	NaN	-1.08	-0.91	-0.78	NaN	-1.72	NaN	NaN
BMA1786	conserved hypothetical protein	-3.27	-1.61	-2.12	-2.96	-3.58	-3.07	-2.93	-2.84	-3.70	-3.19	-1.40	-1.48
BMA3009	lipoprotein, putative	-0.77	-0.82	NaN	-2.18	-1.54	-1.78	-1.48	-1.15	NaN	NaN	-1.39	-1.43
BMA0797	conserved hypothetical protein	-1.95	-1.96	-2.48	-1.57	-1.25	-1.64	-0.56	-0.76	-1.24	-1.48	-1.48	-1.36
BMA1259	transcriptional regulator, GntR family	-2.22	-2.02	-1.31	-2.77	-2.94	-2.94	-2.04	-1.70	NaN	-2.12	-1.54	-1.36
BMA2453	leucyl-tRNA synthetase	-2.31	-2.25	-0.38	-1.77	-2.02	-2.40	-1.31	-0.84	-1.84	-1.90	-1.52	-1.38
BMA2634	translation elongation factor Tu	-3.23	-2.57	-3.68	-3.81	-2.86	-4.68	-3.84	-2.84	-3.87	-3.03	-1.47	-1.43
BMA3333	flagellar protein FlgJ	-1.81	-1.01	-1.93	-0.68	-1.51	-1.08	-0.98	-0.67	-2.00	-1.25	-1.48	-1.43
BMAA1781	conserved hypothetical protein	-1.81	-1.43	-2.65	-1.41	-2.25	-1.19	-1.75	-1.57	-2.01	-1.36	-1.44	-1.41
BMA0527	conserved hypothetical protein	-2.65	-2.16	NaN	-1.99	-1.84	-2.50	-1.75	-1.38	-2.04	-1.79	-1.21	-1.42
BMA1767	peptidyl-tRNA hydrolase, putative	-1.76	-0.83	NaN	-2.21	-1.95	-1.33	-0.69	-0.58	-1.71	-1.44	-1.26	-1.41
BMA2643	ribosomal protein L10	-4.27	-3.43	-2.29	-3.68	-4.29	-4.27	-3.89	-3.48	-4.24	-3.54	-1.27	-1.48
BMA0752	hypothetical protein	-3.52	-2.78	-2.89	-3.02	-2.95	-2.98	-1.58	-1.60	-2.77	-1.51	-1.28	-1.50
BMA3341	hypothetical protein	NaN	-0.88	NaN	NaN	-1.13	-0.71	-0.80	-1.06	NaN	-1.66	-1.28	-1.49
BMAA1963	radical SAM domain protein	-1.41	-1.75	-0.40	-2.13	-2.08	-2.40	-1.28	-1.11	-2.16	-1.91	-1.32	-1.38
BMA3109	ribosomal subunit interface protein	-5.26	-5.31	-3.40	-5.41	-5.78	-5.47	-3.83	-3.00	-5.61	-4.75	-1.22	-1.94
BMA0433	phosphoserine aminotransferase	-2.58	-2.08	NaN	-2.40	-2.22	-1.70	-1.41	-1.68	-2.28	-1.70	-1.32	-1.87
BMA1726	oligopeptidase A	-3.05	-2.72	-2.38	-2.06	-2.27	-2.44	-1.79	-2.01	-2.22	-1.65	-1.36	-1.84
BMA2620	ribosomal protein L5	-3.44	-3.22	-0.93	-3.38	-3.99	-4.35	-3.62	-3.87	-4.32	-3.64	-1.26	-1.75
BMA1988	dTDP-4-dehydrorhamnose 3,5-epimerase	-2.46	-2.04	-1.16	-2.98	-3.02	-2.82	-2.98	-2.29	-2.84	-2.68	-1.29	-1.76
BMA3138	RNA polymerase sigma-32 factor	-3.61	-3.49	-3.85	-2.69	-2.27	-2.30	-1.90	-2.09	-2.83	-2.25	-1.31	-1.75
BMAA0871	hypothetical protein	-5.48	-4.77	-5.26	-3.04	-5.05	-3.96	-2.82	-2.09	-3.55	-3.72	-1.21	-1.80
BMA1981	O-antigen methyl transferase, putative	-1.27	-0.97	-0.61	-2.36	-2.64	-2.43	-2.16	-1.84	-2.59	-1.53	-1.46	-2.13
BMA1813	conserved hypothetical protein	-1.42	-1.54	-1.46	-2.29	-1.59	-1.88	-1.43	-1.71	-1.50	-1.26	-1.51	-2.07
BMA2635	translation elongation factor G	-3.72	-3.06	-2.22	-3.59	-4.04	-3.48	-3.39	-3.29	-4.43	-3.40	-1.45	-2.04
BMA2705	membrane protein, putative	-2.26	-1.83	-1.62	-2.59	-3.31	-3.28	-2.27	-1.68	-2.92	-2.62	-1.45	-2.05
BMA2231	ribosomal protein L28	-4.11	-3.97	-2.37	-3.13	-4.61	-3.90	-3.26	-2.55	-4.12	-3.05	-1.70	-2.24
BMA0256	conserved hypothetical protein	NaN	NaN	NaN	-1.67	NaN	-1.78	-1.04	-0.61	-1.22	-1.60	-1.68	-2.09
BMA2690	conserved hypothetical protein	NaN	-2.84	NaN	-3.03	-2.13	-2.86	-2.44	-2.07	NaN	-2.17	-1.69	-2.08
BMA3125	pantetheine-phosphate adenylyltransferase	-2.19	-1.34	-1.70	-2.11	-2.75	-2.73	-1.11	-1.11	-2.70	-2.37	-1.68	-2.01
BMA2625	ribosomal protein L16	-3.69	-3.87	-1.87	-2.94	-4.19	-3.61	-2.87	-3.02	-3.87	-2.95	-1.77	-2.10
BMA3396	hypothetical protein	-1.65	-2.15	-1.69	-1.37	-1.55	-1.82	-2.01	-1.74	-1.87	-1.35	-1.83	-2.05
BMA0428	ribosomal protein S1	-2.92	-3.22	-3.67	-3.49	-3.80	-3.32	-3.23	-2.80	-4.74	-2.66	-1.79	-1.80
BMA2075	serine hydroxymethyltransferase	-3.27	-2.87	-2.84	-3.56	-4.16	-4.22	-2.98	-2.82	-4.65	-1.06	-1.74	-1.80
BMA0403	ribosomal protein S16	-2.80	-3.22	-1.72	-2.85	-3.64	-3.12	-2.00	-1.97	-2.93	-2.85	-1.78	-1.74
BMA2622	ribosomal protein L14	-3.31	-4.48	NaN	-3.50	-4.01	-4.43	-3.48	-3.75	-4.23	-3.70	-1.82	-1.71
BMA2341	ribosomal protein L13	-4.91	-4.54	-3.52	-4.21	-4.41	-4.91	-4.02	-3.75	-4.13	-4.12	-1.75	-1.64
BMA1947	heat shock protein HtpG	-3.44	-2.96	-2.33	-1.85	-2.51	-2.47	-2.41	-2.21	-2.55	-2.05	-1.67	-1.54
BMA2298	capsular polysaccharide biosynthesis protein, putative	-2.99	-2.83	-3.44	-2.92	-3.33	-3.37	-2.85	-2.73	-3.37	-2.98	-1.65	-1.53
BMA0351	tryptophan 2,3-dioxygenase family protein	-1.10	-1.16	-1.02	-2.75	-1.23	-1.30	-1.46	-1.39	-0.17	-0.24	-1.74	-1.54
BMA2005	transcriptional regulator family protein	-1.70	-1.54	NaN	-2.12	-1.01	-1.52	-1.43	-1.66	-1.78	-1.41	-1.73	-1.53
BMA3178	cytochrome d ubiquinol oxidase, subunit I	-3.10	-3.55	-2.61	-0.57	-3.90	-3.85	-2.02	-1.59	-0.38	-0.56	-1.72	-1.56
BMA3292	ferredoxin--NADP reductase	-1.80	-1.55	-1.88	-2.42	-2.60	-3.03	-2.10	-1.66	-3.15	-0.54	-1.64	-1.64
BMA1935	cytochrome C4 family protein, authentic frameshift	-2.43	-2.59	-2.49	-2.92	-2.71	-3.10	-2.35	-2.18	-2.80	-2.29	-1.65	-1.70
BMAA0002	phage integrase family protein	-2.13	-1.22	-1.94	-2.78	-2.30	-2.60	-2.72	-2.36	-0.82	-1.20	-1.63	-1.73

BMA0798	hypothetical protein	-2.44	-1.90	-1.84	-2.23	-1.26	-1.83	-1.94	-1.61	-1.28	-2.71	-1.63	-1.86
BMA1547	outer membrane protein, OMP85 family	-3.28	-3.16	-2.24	-2.94	-3.06	-3.48	-2.52	-2.48	-3.23	-2.49	-1.67	-1.84
BMA0606	Rrf2 family protein	-2.81	-2.31	-2.34	NaN	-1.98	-2.00	-0.83	-0.67	NaN	-0.25	-1.57	-1.78
BMA1690	3-deoxy-8-phosphooctulonate synthase	-1.89	-2.43	-1.18	-2.60	-2.48	-2.80	-1.94	-1.73	-2.59	-1.79	-1.57	-1.80
BMAA0040	hypothetical protein	-1.38	-0.70	-1.57	-1.32	-1.22	-0.94	-1.29	-1.49	-1.62	-1.45	-1.53	-1.87
BMA0371	ebsC protein, putative	-2.45	-1.95	-0.71	NaN	-0.83	-1.05	-0.84	-1.01	-0.61	-0.24	-1.47	-1.88
BMA2944	glucose-inhibited division protein A	-3.02	-1.78	NaN	-2.80	-3.03	-2.92	-2.51	-2.08	-2.90	-2.26	-1.42	-1.87
BMAA0937	transcriptional regulator, ArsR family	-2.74	-1.64	NaN	NaN	NaN	-1.20	-1.02	-1.08	NaN	NaN	-1.46	-1.81
BMAA1984	sigma-54 dependent transcriptional regulator, authentic point mutation	-0.40	NaN	NaN	NaN	NaN	0.11	-0.40	-0.30	NaN	-1.54	-1.49	-1.80
BMA1487	antioxidant, AhpC/Tsa family	-4.08	-4.05	-4.00	-4.01	-4.40	-4.81	-3.37	-3.58	-3.99	-3.46	-1.94	-1.80
BMA1117	transposase, IS3 family, truncation	NaN	-1.63	NaN	-1.87	NaN	-1.42	-1.93	-1.71	NaN	NaN	-2.01	-1.88
BMAA0416	hypothetical protein	-1.29	-1.45	-1.67	-2.58	-1.80	-2.03	-3.02	-2.26	-2.51	-2.02	-1.98	-1.88
BMA1818	NADH dehydrogenase I, L subunit	-1.85	-2.01	NaN	-2.00	-2.42	-2.71	-1.86	-2.08	NaN	NaN	-1.82	-1.91
BMA2321	2-amino-4-hydroxy-6-hydroxymethyldihydropteridine pyrophosphokinase	-1.75	-1.77	-1.58	-1.80	-1.65	-1.72	-1.15	-1.19	-2.16	-1.69	-1.85	-1.91
BMA2649	translation elongation factor Tu	-1.96	-3.26	-2.61	-2.86	-3.61	-4.92	-3.34	-3.44	-3.22	-2.21	-1.86	-1.89
BMA1673	peptidyl-prolyl cis-trans isomerase, FKBP-type, authentic frameshift	-3.67	-3.00	-3.89	-4.15	-2.59	-3.03	-2.30	-2.21	-3.75	-3.13	-1.91	-1.88
BMA3124	ferredoxin	NaN	-1.22	-1.50	-1.92	-2.21	-2.33	-0.99	-1.59	-2.99	-2.68	-1.88	-1.94
BMAA2111	sigma-70 factor, putative	-1.78	-1.77	-1.47	-1.88	-1.60	-2.00	-1.94	-1.49	-1.31	-1.82	-1.89	-1.93
BMA1099	conserved hypothetical protein	-4.22	-2.30	-2.55	-3.05	-3.67	-3.70	-2.66	-2.24	-2.17	-3.08	-0.50	-1.33
BMA1787	transcriptional regulator, putative	-2.39	-4.88	-1.72	-2.19	-4.84	-6.50	-3.20	-3.24	-2.55	-2.42	-0.57	-1.41
BMA1723	DNA-binding response regulator, LuxR family	-2.70	-2.02	-2.93	-2.69	-2.88	-2.74	-1.85	-1.40	-2.98	-3.00	-0.40	-1.01
BMA2343	iron-sulfur cluster assembly accessory protein	-1.74	-0.34	NaN	-2.49	-1.34	-0.48	-2.13	-0.78	-2.93	-1.47	-0.39	-1.16
BMA2707	phosphoribosyl-AMP cyclohydrolase	-1.42	-0.94	-1.42	-1.95	-2.09	-1.83	-1.73	-1.31	-2.33	-1.56	-0.51	-1.17
BMA2872	ribosomal protein S21	-3.24	-3.03	NaN	-2.77	-3.33	-3.51	-2.30	-2.10	-2.04	-2.74	-0.65	-1.14
BMA0315	efflux transporter, RND family, MFP subunit	-1.78	-1.34	NaN	-2.43	-2.00	-1.88	-1.30	-0.95	-2.74	-2.16	-0.54	-1.07
BMAA0238	hypothetical protein	NaN	-1.03	NaN	-1.84	NaN	-1.82	-1.11	-1.10	NaN	-1.99	-0.54	-1.01
BMAA0883	iron permease, FTR1 family	-0.69	-0.27	NaN	NaN	-0.36	-0.55	-1.63	-1.35	-2.28	-2.11	-0.55	-1.03
BMA1714	transcriptional regulator, IclR family	-1.14	-1.47	NaN	-2.08	0.07	-0.96	-0.80	-0.73	NaN	-1.05	-0.64	-0.91
BMAA0031	transcriptional regulator, Crp/Fnr family	-3.22	-2.36	-3.18	-2.48	-2.47	-2.59	-1.95	-1.75	NaN	-1.13	-0.59	-0.95
BMA2279	cold-shock domain family protein	-5.34	-4.67	-6.46	-6.21	-6.67	-5.60	-4.48	-3.93	-5.19	-4.94	-0.57	-0.87
BMA2627	ribosomal protein L22	-3.60	-3.72	NaN	-3.29	-3.32	-3.31	-2.46	-2.31	-3.63	-3.28	-0.52	NaN
BMAA0853	hypothetical protein	-2.02	-1.14	-2.05	NaN	-0.73	-1.44	-0.57	-0.36	-0.52	-0.99	-0.53	-0.94
BMA2500	3-dehydroquinate dehydratase, type II	-1.05	-1.22	NaN	-1.52	-1.64	-2.05	-1.55	-1.58	-1.62	-1.33	-0.67	-0.97
BMA2706	phosphoribosyl-ATP pyrophosphohydrolase	NaN	-0.25	NaN	-1.38	-1.34	-1.88	-1.44	-1.12	NaN	-1.47	-0.65	-0.98
BMAA2035	stress response protein	-3.81	-3.62	-3.43	NaN	-3.04	-2.14	-1.28	-0.71	NaN	-0.39	-0.64	-1.04
BMA2917	conserved hypothetical protein	-0.62	-0.36	NaN	-2.14	-1.67	-1.59	-1.36	-1.50	-2.43	-1.52	-0.83	-1.64
BMAA0873	hypothetical protein	-1.01	-0.54	-0.81	NaN	-0.79	-0.03	-1.71	-1.06	NaN	-0.84	-0.70	-1.68
BMAA1790	conserved hypothetical protein, truncation	NaN	-0.77	NaN	-2.26	-1.29	-1.28	-1.57	-0.95	NaN	-1.59	-0.68	-1.55
BMA2927	phenylalanine-4-hydroxylase	-1.33	-1.51	-1.36	-2.04	-1.53	-1.94	-1.37	-1.54	-1.15	-1.28	-0.88	-1.23
BMA0549	sigma-54 dependent DNA-binding response regulator	-2.53	-1.56	-2.11	-2.08	-1.12	-1.85	-1.26	-0.85	NaN	-0.94	-0.79	-1.24
BMAA0875	oxidoreductase, aldo/keto reductase family	-1.28	-0.92	-1.71	-2.17	-0.90	-1.20	-2.03	-1.58	-1.71	-1.58	-0.78	-1.22
BMA0683	conserved hypothetical protein	-3.42	-2.86	-2.19	-2.47	-3.08	-2.82	-1.76	-1.73	-2.79	-1.04	-0.86	-1.12
BMA2050	D-lactate dehydrogenase	-1.46	-0.92	-2.26	-1.05	-1.64	-1.77	-1.42	-1.23	-2.23	-1.40	-0.90	-1.10
BMA2709	phosphoribosylformimino-5-aminoimidazole carboxamide ribotide isomerase	-1.92	-2.17	-2.59	-2.87	-2.43	-2.42	-2.40	-2.43	-3.58	-2.26	-0.82	-1.09
BMA0001	chromosomal replication initiator protein DnaA	-2.76	-2.83	-1.69	-1.53	-2.40	-2.60	-1.22	-1.05	-1.72	-1.91	-0.78	-1.05
BMA0776	ribosomal RNA large subunit methyltransferase J	NaN	-1.85	-1.62	NaN	-1.50	-2.58	-1.09	NaN	-0.86	-2.68	NaN	NaN
BMA1060	ribosome-binding factor A	-2.65	-2.47	NaN	-1.81	-1.58	-1.56	-1.25	-0.94	NaN	-1.86	-0.74	-1.07
BMAA0038	hypothetical protein	NaN	-1.29	NaN	-0.98	-1.53	-1.55	-1.30	-0.78	-1.45	-1.46	-0.78	-1.14
BMA1826	NADH dehydrogenase I, D subunit	-4.45	-3.42	NaN	-2.78	-3.28	-3.51	-2.48	-2.48	NaN	-1.67	-0.77	NaN

BMA0192	conserved hypothetical protein	-1.69	-1.31	NaN	NaN	-1.48	-2.06	-1.08	-1.59	NaN	-1.23	-0.77	-1.16
BMAA0866	hypothetical protein	-4.71	-3.36	-3.63	-3.37	-4.29	-3.69	-2.60	-2.15	-3.44	-3.46	NaN	NaN
BMA1453	peptidyl-prolyl cis-trans isomerse D, putative	-1.64	-1.43	-0.22	-1.49	-1.96	-2.37	-1.77	-1.34	-2.40	-1.88	-0.75	-1.18
BMAA1546	hypothetical protein	-1.99	-1.30	NaN	NaN	NaN	-0.75	-1.10	-0.47	NaN	-1.20	-0.75	NaN
BMAA0943	conserved hypothetical protein	-3.40	-2.94	-3.02	-3.10	-2.46	-2.52	-1.32	-1.33	-2.63	-2.19	-0.87	-1.02
BMA1378	Rrf2 family protein	-2.56	-2.63	-2.01	-3.36	-2.03	-2.38	-2.14	-1.76	-2.50	-2.58	-0.95	-1.04
BMA2509	ribonucleoside-diphosphate reductase, beta subunit	-1.24	-2.00	-1.60	-2.37	-2.94	-3.15	-2.24	-2.41	-3.22	-2.77	-0.97	-1.08
BMAA1805	hypothetical protein	-0.96	-0.28	NaN	-1.17	-0.53	-0.70	-1.21	-1.21	-4.11	-3.68	-0.96	-1.11
BMA1820	NADH dehydrogenase I, J subunit	-2.11	-1.80	NaN	-1.24	-2.31	-2.57	-1.85	-1.61	NaN	-1.13	-1.02	-1.47
BMA2695	stringent starvation protein A	-2.04	-2.31	-0.91	-2.08	-2.56	-2.79	-2.28	-2.34	-2.96	-1.98	-1.00	-1.48
BMA2712	imidazoleglycerol-phosphate dehydratase	-2.06	-1.44	-1.99	-2.03	-2.23	-2.33	-1.37	-1.20	-2.27	-2.03	-0.89	-1.44
BMA2469	transketolase	-3.27	-2.48	-3.13	-2.79	-3.17	-1.92	-1.79	-1.47	-3.27	-1.92	-0.93	-1.46
BMA3381	conserved hypothetical protein	-1.87	-1.29	-1.43	-2.11	-1.96	-1.97	-1.07	-0.56	-1.77	-1.41	-0.95	-1.42
BMA2754	conserved hypothetical protein	-2.29	-1.84	-2.70	-2.30	-3.14	-2.18	-1.92	-1.39	-3.13	-2.11	-1.00	-1.40
BMA1325	competence lipoprotein ComL	-3.75	-3.20	-3.46	-3.19	-3.66	-3.58	-2.88	-1.90	-3.77	-3.25	-0.99	-1.33
BMA2926	pterin-4-alpha-carbinolamine dehydratase, putative	-1.78	-1.47	-1.95	-1.94	-1.68	-1.82	-0.92	-0.99	-0.95	-1.24	-0.95	-1.35
BMA0094	isocitrate dehydrogenase kinase/phosphatase	-2.56	-2.67	NaN	-1.52	-0.93	-1.87	-0.73	-1.55	NaN	-1.12	-0.96	NaN
BMA2173	protein-L-isoaspartate O-methyltransferase, putative	-2.33	-1.57	NaN	NaN	-2.21	-2.29	-1.29	-1.27	NaN	-1.08	NaN	NaN
BMAA0024	hypothetical protein	-3.45	-1.69	-4.15	-4.60	NaN	-1.67	-2.35	-1.77	-4.04	-3.56	-0.96	-1.36
BMA1603	hypothetical protein	-3.04	-1.76	-2.26	-1.34	-1.95	-1.78	-1.51	-1.20	-2.32	-2.52	-1.12	-1.55
BMA1465	ATP-dependent Clp protease, proteolytic subunit ClpP	-2.79	-3.30	-2.96	-3.06	-2.45	-2.99	-1.91	-2.09	-3.37	-3.21	-1.18	-1.64
BMAA1507	hypothetical protein	-3.01	-3.24	-2.69	-2.01	-2.88	-2.91	-1.05	-1.18	-2.44	-2.33	-1.22	-1.57
BMA2535	conserved hypothetical protein	-1.91	-2.20	-2.03	-2.28	-1.98	-2.61	-1.94	-1.98	-2.34	-1.74	-1.15	-1.18
BMA2787	ipgF protein, putative	-0.90	-0.83	-0.69	-2.13	-1.30	-1.64	-1.46	-1.14	-1.70	-1.53	-1.14	-1.14
BMA2078	tolQ protein	-0.77	-0.53	NaN	NaN	-1.27	-0.95	-1.83	-1.44	NaN	-1.68	-1.11	-1.20
BMA2587	orotate phosphoribosyltransferase	-2.56	-2.27	-2.08	-1.53	-2.45	-2.48	-1.83	-0.98	-1.42	-1.30	-1.09	-1.20
BMAA0018	acetoacetate decarboxylase	-1.78	-2.06	-0.79	NaN	-1.32	-1.89	-1.73	-2.03	-2.14	-1.34	-1.09	-1.16
BMA2089	outer membrane porin, putative	-3.06	-3.01	-3.85	-3.14	-3.54	-3.37	-2.28	-2.51	-2.56	-2.21	-1.05	-1.12
BMA2232	ribosomal protein L33	-4.68	-4.68	-2.51	-3.41	-5.24	-4.48	-3.02	-2.70	-4.33	-3.77	-1.05	-1.12
BMA3088	conserved hypothetical protein	-2.62	-1.41	NaN	NaN	-4.64	-3.04	NaN	-1.48	NaN	-1.58	-1.02	-1.18
BMA2522	ribosomal protein L27	-3.79	-4.34	-2.41	-3.47	-4.38	-4.35	-3.37	-2.83	-3.51	-3.45	-1.04	-1.22
BMA0599	fatty acid desaturase domain protein	-2.39	-2.58	-1.89	NaN	-2.65	-2.74	-0.30	-0.59	NaN	-0.01	-0.99	-1.23
BMAA0519	GTP-binding protein YchF	-2.48	-2.73	NaN	-2.02	-2.29	-2.63	-1.35	-1.40	NaN	-1.58	-0.99	-1.20
BMA1369	ribosomal protein L31	-5.84	-5.13	-3.91	-4.35	-5.90	-5.52	-3.79	-3.58	-5.55	-3.98	-1.07	-1.27
BMA2611	translation initiation factor IF-1	-5.04	-3.99	-1.85	-3.87	-4.66	-4.50	-2.94	-2.48	-3.83	-3.91	-1.04	-1.27
BMA2696	ubiquinol-cytochrome c reductase, cytochrome c1	-1.92	-2.29	-2.39	-3.26	-2.25	-2.43	-2.36	-2.63	-2.78	-2.41	-1.09	-1.30
BMA0743	outer membrane porin, putative	NaN	NaN	NaN	-3.35	NaN	-0.75	-1.61	-1.65	-1.87	-1.68	-1.10	-1.35
BMA2946	sporulation initiation inhibitor protein Soj	-1.96	-1.08	-1.39	-2.21	-2.49	-2.03	-2.12	-1.17	-1.74	-1.57	-1.10	-1.35
BMAA1353	outer membrane porin OpcP	-2.60	-4.34	-2.89	-2.56	-3.19	-5.43	-3.49	-2.65	-2.91	-1.88	-1.11	-1.39
BMA1717	D-alanyl-D-alanine endopeptidase, putative	-2.18	-2.59	-2.37	-2.48	-2.65	-3.21	-1.99	-2.11	-2.42	-2.27	-1.24	-1.34
BMAA0422	conserved hypothetical protein	-0.97	-0.53	-0.96	-1.33	-0.76	-1.31	-1.30	-1.15	-1.25	-1.43	-1.23	-1.33
BMA1052	2-oxoglutarate dehydrogenase, E1 component	-3.81	-3.87	-3.23	-3.19	-3.24	-3.73	-2.74	-2.36	-3.34	-2.91	-1.17	-1.29
BMAA2113	arsenate reductase	-1.74	-1.35	-1.26	-1.90	-2.09	-2.22	-2.70	-1.83	NaN	-1.85	-1.19	-1.33
**Group 3**													
BMAA0728	hypothetical protein	0.30	1.02	-0.36	-0.16	0.02	-0.10	-1.17	-0.34	-2.14	-1.67	-1.99	-2.17
BMAA0277	hypothetical protein	1.48	0.54	1.87	0.17	1.15	0.31	-0.34	-1.16	0.86	0.11	-1.54	-1.34
BMA1144	arginine/ornithine antiporter	-2.75	-2.99	-2.97	0.77	-0.47	-0.85	-0.07	-0.10	-0.33	0.53	-1.54	-1.70
BMAA2014	hypothetical protein	1.56	1.65	NaN	1.41	2.00	1.00	-0.46	1.97	1.35	1.29	-1.87	0.13
BMAA1511	conserved hypothetical protein	0.47	0.01	0.01	-0.28	0.16	-0.21	-0.16	-0.92	-3.45	-3.23	-1.00	-0.70

BMAA1488	hypothetical protein	-2.61	-2.29	-2.85	0.61	-1.24	-1.22	-0.16	-0.30	NaN	0.59	-0.94	-1.27
BMA0605	conserved hypothetical protein	-3.90	-2.84	-4.09	-0.15	-3.86	-3.17	-1.39	-1.01	NaN	0.63	-1.15	-1.06
BMAA0011	tartrate dehydrogenase	0.43	0.59	0.41	0.90	0.87	0.82	-0.35	-0.40	1.24	1.58	0.02	-0.34
BMAA0751	N-acetylmuramoyl-L-alanine amidase domain protein	-0.16	0.95	NaN	NaN	NaN	1.00	0.68	0.18	-2.57	-2.40	0.01	-0.08
BMAA1548	type III secretion system protein BsaL	-2.65	-2.34	-2.64	NaN	NaN	0.10	0.51	0.22	-1.81	-1.57	0.05	-0.12
BMA3179	hypothetical protein	-1.91	-1.29	-2.47	-0.02	-2.19	-2.00	-0.42	-0.45	0.22	0.39	-0.23	-0.16
BMAA1662	response regulator	0.73	1.34	0.32	0.66	0.54	1.15	0.39	-0.32	NaN	1.11	-0.62	-0.37
BMAA0089	membrane protein, putative	0.52	0.26	1.36	NaN	NaN	1.31	-0.73	-0.76	NaN	0.82	-0.38	-0.62
BMA0656	glutamine synthetase family protein	-1.25	-1.57	-2.18	-2.66	0.44	0.24	-0.96	-1.13	-0.89	-0.84	-0.71	-0.68
BMA2323	3-methyl-2-oxobutanoate hydroxymethyltransferase	0.08	0.86	NaN	NaN	-0.35	-0.04	NaN	-0.05	NaN	0.08	-0.66	-0.77
BMAA0023	cytochrome P450-related protein	1.89	1.66	NaN	0.45	NaN	1.97	-0.78	-0.06	NaN	1.09	-0.51	-0.97
BMAA0198	muconolactone delta-isomerase	0.36	NaN	-0.15	-2.33	NaN	0.18	-0.68	-0.91	0.84	-0.11	-0.52	-0.90
BMAA1785	chitin binding domain protein	0.91	2.02	0.98	1.21	0.94	1.85	0.49	0.89	-0.65	-0.37	2.38	2.22
BMA1148	oxidoreductase, short-chain dehydrogenase/reductase family	-0.39	-1.06	0.23	1.85	1.60	1.26	1.80	1.68	1.81	1.47	2.39	2.73
BMA2726	ABC transporter, ATP-binding protein	0.19	0.40	-1.31	0.06	-0.33	-1.16	-1.84	-0.09	-0.35	0.06	-0.19	0.88
BMAA1547	type III secretion system protein BsaM	-0.77	-0.78	-1.43	NaN	0.76	0.63	0.86	0.96	-0.54	-0.29	0.37	0.20
BMA0018	hypothetical protein	1.38	0.70	2.16	0.21	1.69	1.14	1.23	0.36	-0.35	-0.46	0.39	-0.29
BMA1051	2-oxoglutarate dehydrogenase, E2 component, dihydrolipoamide succinyltransferase	-1.25	-0.74	NaN	NaN	NaN	0.03	0.91	1.11	NaN	0.31	NaN	NaN
BMAA1828	hemin ABC transporter, periplasmic hemin-binding protein	0.51	1.04	NaN	NaN	NaN	1.08	1.42	1.04	-1.99	-1.92	0.48	NaN
BMA1191	RNA polymerase sigma-70 factor, ECF subfamily	NaN	0.08	NaN	NaN	NaN	0.49	-0.36	0.41	-5.11	-2.93	0.18	NaN
BMAA0708	conserved hypothetical protein	-3.15	-2.86	-3.81	0.95	-2.07	-1.97	-0.22	-0.23	NaN	0.34	0.28	-0.18
BMAA0638	sugar ABC transporter, ATP-binding protein	0.63	0.89	-0.39	NaN	0.51	1.62	1.36	1.27	1.56	1.45	0.57	0.59
BMAA1549	type III secretion system protein BsaK	-0.63	-0.39	-0.12	NaN	1.79	1.31	1.80	1.30	0.41	-0.32	0.79	0.42
BMA3176	cyd operon protein YbgT	-1.42	-1.64	-1.60	-0.75	-1.29	-1.41	0.29	0.12	0.38	-0.64	0.61	0.24
BMAA0703	DNA-binding response regulator, LuxR family	0.29	0.58	-0.33	0.56	1.09	1.03	0.62	1.52	1.14	2.41	1.31	1.63
BMAA1431	cysteine-rich domain protein	0.30	-0.04	-0.53	1.36	1.68	1.24	1.97	1.01	1.84	1.65	1.48	1.58
BMAA2034	conserved hypothetical protein	-0.44	-0.81	-1.74	1.53	0.28	-0.26	1.40	1.67	1.58	NaN	1.57	1.83
BMA1190	mbtH-like protein	0.22	1.17	0.38	0.43	0.41	1.99	0.85	1.60	-2.63	-1.53	1.83	1.34
BMA1205	cys regulon transcriptional activator	1.02	0.70	NaN	0.52	1.46	0.11	0.22	0.04	-0.53	-0.88	1.10	1.38
BMAA0732	conserved hypothetical protein	1.36	1.36	0.85	1.34	0.83	2.03	2.04	1.57	-0.09	-0.48	1.04	1.26
BMAA1529	DNA-binding protein BprA	-0.69	-1.10	-1.72	1.19	1.23	0.81	1.06	0.73	-1.44	-1.56	0.75	1.40
BMA1304	outer membrane protein, OmpW family	-3.04	-2.92	-2.60	0.77	-3.11	-3.20	-0.23	-0.79	0.64	0.66	1.11	0.95
BMAA1395	cyclic nucleotide-binding domain protein	-2.03	-1.19	-2.39	-0.86	-2.06	-1.05	0.15	0.40	-2.17	-1.44	0.99	1.02
BMA2751	hypothetical protein	-0.35	1.03	-0.88	0.30	-0.31	1.40	0.45	1.56	-0.68	0.57	0.93	0.84
BMAA1145	sensor histidine kinase	-0.04	0.49	-0.68	0.71	0.03	0.42	0.93	1.52	0.25	0.69	0.98	0.90
BMA3052	nitrite/sulfite reductase family protein	-1.77	-1.58	-0.96	-1.53	-1.86	-1.93	0.68	0.55	0.51	0.48	0.79	0.78
BMA0455	glutamine amidotransferase, class I	-0.97	-1.44	-0.62	-1.05	0.37	-0.76	0.12	-0.08	NaN	-0.57	0.82	0.82
