# Supplementary material for: Bacterial genome adaptation to niches: Divergence of the potential virulence genes in three Burkholderia species of different survival strategies
Source: BMC Genomics. 2005 Dec 7;6:174. doi: 10.1186/1471-2164-6-174 (PMC1343551; doi:10.1186/1471-2164-6-174)
Supplement: Additional File 4 — Comparison of the whole genomes in Fig. 3A. [file 1471-2164-6-174-S4.pdf]

Sup_Table_2. Comparison of the whole genomes in Fig. 3A.

Segment 1- Conserved in Bm, Bp, and Bt

		to Bp		to Bt	
**Gene**	**Description**	**% length**	**% identity**	**% length**	**% identity**
BMA0001	chromosomal replication initiator protein DnaA	100	99	100	96
BMA0002	DNA polymerase III, beta subunit	100	100	100	99
BMA0003	DNA gyrase, B subunit	100	100	100	99
BMA0006	carboxymuconolactone decarboxylase family protein	100	98	100	99
BMA0010	hypothetical protein	100	99	100	92
BMA0011	hypothetical protein	100	100	100	91
BMA0014.1	hypothetical protein	100	99	96	94
BMA0018	hypothetical protein	100	99	100	95
BMA0019	FHA domain protein	100	100	100	94
BMA0020	protein kinase domain protein	100	99	100	90
BMA0023	conserved hypothetical protein	100	99	100	90
BMA0024	aldolase, class II	100	98	100	91
BMA0027	polysaccharide biosynthesis family protein	100	100	100	96
BMA0028	glycosyl transferase, group 1 family protein	100	99	100	94
BMA0029	mannose-1-phosphate guanylyltransferase/mannose-6-phosphate isomerase	100	99	100	92
BMA0030	ElaA family protein	100	99	100	90
BMA0032	glycosyl transferase, group 1 family protein	100	99	100	93
BMA0037	sigma-54 dependent transcriptional regulator	100	99	100	97
BMA0039	beta-mannosidase-related protein	100	99	100	91
BMA0040	conserved hypothetical protein	100	100	100	94
BMA0041	conserved hypothetical protein	100	99	100	95
BMA0042	acyl-CoA dehydrogenase domain protein	100	99	100	96
BMA0043	acyl carrier protein, putative	100	100	100	95
BMA0044	conserved hypothetical protein	100	99	100	96
BMA0045	conserved hypothetical protein	100	100	100	98
BMA0046	polysaccharide biosynthesis glycosyltransferase, putative	100	99	100	92
BMA0047	capsular polysaccharide biosynthesis/export periplasmic protein	100	99	100	96
BMA0048	glycosyl transferase, group 1 family protein	100	99	100	93
BMA0052	lipoic acid synthetase	100	99	100	99
BMA0053	lipoate-protein ligase B	100	98	100	90
BMA0055	glycine cleavage system transcriptional activator	100	100	98	93
BMA0056	conserved hypothetical protein	100	100	100	99
BMA0057	D-amino acid aminotransferase	100	100	96	95
BMA0058	D-alanyl-D-alanine carboxypeptidase family protein	100	99	100	98
BMA0060	conserved hypothetical protein	100	99	100	94
BMA0061	ferredoxin, 2Fe-2S	100	99	100	96
BMA0062	membrane protein, putative	100	98	100	93
BMA0063	lipoprotein, putative	100	98	99	91
BMA0064	ABC transporter, periplasmic substrate-binding protein	100	100	100	95
BMA0065	ABC transporter, ATP-binding protein	100	99	100	96
BMA0066	ABC transporter, permease protein	100	99	100	97
BMA0067	2`,3`-cyclic-nucleotide 2`-phosphodiesterase	100	99	100	95
BMA0068	conserved hypothetical protein	100	99	100	87
BMA0069	biotin--acetyl-CoA-carboxylase ligase	100	98	100	90

BMA0070	ranscriptional activator, Baf family	100	99	100	94
BMA0072	cytidyltransferase-related domain protein	100	100	99	95
BMA0073	conserved hypothetical protein	100	100	100	99
BMA0074	conserved hypothetical protein, authentic point mutation	100	99	100	95
BMA0075	conserved hypothetical protein	100	98	99	88
BMA0076	enoyl-CoA hydratase/isomerase family protein	100	99	100	97
BMA0077	fumarylacetoacetate hydrolase family protein	100	99	100	97
BMA0081	5-methyltetrahydrofolate--homocysteine methyltransferase, authentic point mutation	100	99	100	95
BMA0083	conserved hypothetical protein	100	100	100	99
BMA0084	arginyl-tRNA synthetase	100	99	100	97
BMA0085	sporulation-related repeat protein	100	100	100	96
BMA0086	thiol:disulfide interchange protein DsbA	100	100	100	98
BMA0087	oxidoreductase, short-chain dehydrogenase/reductase family	100	99	100	96
BMA0088	3-alpha-hydroxysteroid dehydrogenase, putative	100	100	100	89
BMA0089	transcriptional regulator, MarR family	100	100	100	90
BMA0091	peptide ABC transporter, periplasmic peptide-binding protein	100	99	100	96
BMA0092	peptide ABC transporter, ATP-binding protein	100	100	100	94
BMA0093	metallo-beta-lactamase family protein	100	99	99	91
BMA0094	isocitrate dehydrogenase kinase/phosphatase	100	100	100	97
BMA0095	beta carbonic anhydrase	100	100	100	94
BMA0096	3-ketoacyl-CoA thiolase	100	100	100	96
BMA0097	oxidoreductase, short chain dehydrogenase/reductase family	100	98	100	92
BMA0098	conserved hypothetical protein	100	100	100	92
BMA0100	adenosylmethionine-8-amino-7-oxononanoate aminotransferase	100	99	100	97
BMA0101	8-amino-7-oxononanoate synthase	100	100	100	94
BMA0102	dethiobiotin synthetase	100	99	100	87
BMA0103	biotin synthase	100	99	100	98
BMA0104	cutC family protein	100	99	100	94
BMA0106	alkaline phosphatase family protein	100	99	100	93
BMA0107	alkaline phosphatase family protein	100	99	100	96
BMA0109	conserved domain protein	99	96	99	89
BMA0113	conserved hypothetical protein	100	100	100	95
BMA0114	glyoxalase family protein	100	100	100	94
BMA0115	ADA regulatory protein	100	99	100	95
BMA0117	glutamate--cysteine ligase	100	99	100	97
BMA0118	RNA polymerase sigma factor RpoD, putative	100	99	100	87
BMA0120.1	hypothetical protein	100	98	100	88
BMA0128	portal protein, PBSX family	100	98	97	98
BMA0133	ISBma1, transposase	100	99	100	98
BMA0135	conserved hypothetical protein	100	100	100	98
BMA0136	patatin-like phospholipase	100	100	100	95
BMA0137	glyoxylate reductase	100	99	100	96
BMA0138	transcriptional regulator, LysR family	100	100	100	98
BMA0139	DNA topoisomerase III	100	99	100	98
BMA0140	conserved hypothetical protein	100	100	100	93
BMA0141	DNA processing protein DprA, putative	100	99	100	92
BMA0142	polypeptide deformylase	100	99	100	97
BMA0143	methionyl-tRNA formyltransferase	100	99	100	92
BMA0144	homoserine/threonine efflux protein, putative	100	100	100	94

BMA0145	heat shock protein HtpX, putative	100	100	100	98
BMA0146	sun protein	100	100	100	94
BMA0147	conserved hypothetical protein	100	99	100	97
BMA0148	nitrogen regulation protein NtrY, putative	100	99	100	98
BMA0149	DNA-binding response regulator	100	99	100	97
BMA0152	exsB protein	100	99	100	96
BMA0153	conserved hypothetical protein	100	100	100	96
BMA0154	6-pyruvoyl tetrahydrobiopterin synthase, putative	100	99	100	98
BMA0156	HpcH/HpaI aldolase family protein	100	100	100	95
BMA0157	conserved hypothetical protein	100	98	98	86
BMA0158	rod shape-determining protein RodA	100	100	100	98
BMA0159	penicillin-binding protein 2	100	97	100	91
BMA0160	rod shape-determining protein MreD	100	99	100	97
BMA0161	rod shape-determining protein MreC	100	100	100	93
BMA0162	rod shape-determining protein MreB	100	100	100	100
BMA0163	glutamyl-tRNA(Gln) amidotransferase, C subunit	100	100	100	96
BMA0164	glutamyl-tRNA(Gln) amidotransferase, A subunit	100	99	100	98
BMA0165	glutamyl-tRNA(Gln) amidotransferase, B subunit	100	99	100	97
BMA0166	conserved hypothetical protein	100	100	100	88
BMA0167	exodeoxyribonuclease III	100	100	100	97
BMA0170	conserved hypothetical protein	100	99	100	92
BMA0171	esterase, putative	100	100	100	94
BMA0172	conserved hypothetical protein	100	100	100	92
BMA0173	drug resistance transporter, EmrB/QacA family	100	99	100	94
BMA0174	transcriptional regulator, MarR family	100	100	100	96
BMA0175	intracellular protease, PfpI family	100	99	100	96
BMA0176	D-serine deaminase, putative	100	98	100	96
BMA0177	transcriptional regulator, putative	100	99	100	94
BMA0179	endoribonuclease L-PSP, putative	100	100	100	100
BMA0180	GTP cyclohydrolase family protein	100	99	100	97
BMA0181	threonine ammonia-lyase, biosynthetic	100	99	100	96
BMA0183	oxidoreductase, FAD-binding	100	99	100	97
BMA0184	conserved hypothetical protein	100	99	100	97
BMA0185	conserved hypothetical protein	100	100	100	95
BMA0186	ubiquinone/menaquinone biosynthesis methlytransferase UbiE	100	100	100	100
BMA0187	conserved hypothetical protein	100	99	100	91
BMA0188	conserved hypothetical protein	100	100	100	93
BMA0189	2-polyprenylphenol 6-hydroxylase	100	100	100	98
BMA0191	conserved hypothetical protein	100	97	100	90
BMA0192	conserved hypothetical protein	100	100	100	98
BMA0193	aspartyl-tRNA synthetase	100	100	100	98
BMA0194	dATP pyrophosphohydrolase	100	100	100	95
BMA0195	cardiolipin synthetase II	100	99	100	95
BMA0196	transcriptional regulator, TetR family	100	100	100	100
BMA0197	acyl-CoA dehydrogenase domain protein	100	99	100	98
BMA0198	3-hydroxyacyl-CoA dehydrogenase/enoyl-CoA hydratase/isomerase family protein	100	99	100	95
BMA0199	thiolase family protein	100	99	100	97
BMA0200	enoyl-CoA hydratase/isomerase family protein	100	100	100	95
BMA0201	formate dehydrogenase accessory protein	100	99	100	95

BMA0202	conserved hypothetical protein	100	100	100	94
BMA0203	thioesterase domain protein	100	99	100	99
BMA0204	ABC transporter, ATP-binding/permease protein	100	100	100	96
BMA0206	nucleotidyltransferase family protein	100	100	100	94
BMA0207	conserved hypothetical protein	100	99	100	93
BMA0208	organic solvent tolerance protein, putative	100	100	100	96
BMA0209	survival protein SurA, putative	100	99	100	96
BMA0210	4-hydroxythreonine-4-phosphate dehydrogenase	100	97	100	91
BMA0211	dimethyladenosine transferase	100	100	99	95
BMA0213	lactoylglutathione lyase	100	100	100	96
BMA0215	conserved hypothetical protein	100	99	100	95
BMA0216	1-acyl-sn-glycerol-3-phosphate acyltransferase, putative	100	100	99	96
BMA0217	HAD-superfamily hydrolase	100	99	100	95
BMA0218	glycyl-tRNA synthetase, beta subunit	100	100	100	95
BMA0219	glycyl-tRNA synthetase, alpha chain	100	100	100	98
BMA0220	apolipoprotein N-acyltransferase	100	99	100	93
BMA0221	magnesium and cobalt efflux protein CorC	100	100	100	98
BMA0222	ChaC-related protein	100	99	100	94
BMA0223	conserved hypothetical protein TIGR00043	100	99	100	92
BMA0225	PhoH family protein	100	99	100	96
BMA0226	tRNA-i(6)A37 modification enzyme MiaB	100	100	100	96
BMA0227	transcriptional regulator, LysR family	100	99	100	93
BMA0228	major facilitator family transporter	100	99	100	94
BMA0229	DNA-binding protein	100	99	100	95
BMA0230	3,4-dihydroxy-2-butanone 4-phosphate synthase	100	100	100	95
BMA0231	HAD-superfamily hydrolase	100	100	100	95
BMA0239	glycerol uptake facilitator protein	100	100	100	97
BMA0240	glycerol kinase	100	100	100	98
BMA0241	glycerol-3-phosphate dehydrogenase	100	99	100	96
BMA0242	lipoprotein, putative	100	100	100	88
BMA0243	hypothetical protein	100	100	100	100
BMA0244	glycerol-3-phosphate regulon repressor	100	100	100	97
BMA0245	gamma-glutamyltransferase	100	99	100	94
BMA0246	conserved hypothetical protein	100	100	100	96
BMA0247	hypothetical protein	100	99	100	89
BMA0248	ATP-dependent RNA helicase RhlE	100	100	100	95
BMA0249	cytochrome c family protein	100	100	100	98
BMA0250	cytochrome c4, putative	100	100	100	96
BMA0252	copper resistance protein, putative	100	100	100	95
BMA0254	DgoA protein	100	99	100	97
BMA0259	Ser/Thr protein phosphatase family protein	100	99	100	96
BMA0260	sensor histidine kinase	100	100	100	91
BMA0261	response regulator	100	99	100	93
BMA0262	hypothetical protein	100	100	100	86
BMA0263	hypothetical protein	100	100	100	88
BMA0264	methyl-accepting chemotaxis protein	100	99	100	92
BMA0269	major facilitator family transporter	100	99	100	97
BMA0270	sensor histidine kinase	100	96	100	91
BMA0271	DNA-binding response regulator	100	99	100	94

BMA0272	recA protein	100	100	100	98
BMA0275	succinyl-CoA synthase, beta subunit	100	100	100	98
BMA0276	succinyl-CoA synthase, alpha subunit	100	99	100	98
BMA0277	integral membrane protein, TerC family	100	99	100	94
BMA0278	type IV pilin, putative	100	99	100	91
BMA0279	O-antigen polymerase family protein	100	99	100	94
BMA0281	conserved hypothetical protein	100	100	100	89
BMA0282	TonB domain protein	100	98	100	93
BMA0283	molybdenum cofactor biosynthesis protein C, c-term	100	100	100	88
BMA0288	conserved hypothetical protein	100	100	100	93
BMA0289	hydrolase, alpha/beta fold family	100	99	100	96
BMA0290	conserved hypothetical protein	100	100	100	100
BMA0291	ADP-heptose--LPS heptosyltransferase II	100	99	100	98
BMA0292	conserved hypothetical protein	100	100	100	100
BMA0293	branched-chain amino acid aminotransferase	100	100	100	99
BMA0294	AzlC family protein	100	98	100	94
BMA0295	membrane protein, putative	100	100	100	95
BMA0295.1	phosphoglycerate kinase	100	99	100	98
BMA0298	pyruvate kinase	100	100	100	99
BMA0299	fructose-bisphosphate aldolase, class II	100	100	100	100
BMA0300	phosphoribosylaminoimidazole-succinocarboxamide synthase	100	100	100	97
BMA0301	phosphoribosylaminoimidazole carboxylase, catalytic subunit	100	100	100	97
BMA0302	phosphoribosylaminoimidazole carboxylase, ATPase subunit	100	99	100	95
BMA0303	Sua5/YciO/YrdC/YwlC family protein	100	100	100	93
BMA0304	conserved hypothetical protein	100	99	100	96
BMA0305	conserved hypothetical protein, authentic point mutation	100	99	100	94
BMA0307	D-alanyl-D-alanine carboxypeptidase/D-alanyl-D-alanine-endopeptidase	100	100	100	95
BMA0308	DNA-binding response regulator	100	100	100	99
BMA0309	sensor histidine kinase	100	99	100	97
BMA0310	serine protease	100	99	100	97
BMA0311	conserved hypothetical protein	100	98	100	89
BMA0312	conserved hypothetical protein	100	97	100	93
BMA0313	conserved hypothetical protein	100	100	100	95
BMA0314	transcriptional regulator, TetR family	100	99	100	98
BMA0315	efflux transporter, RND family, MFP subunit	100	100	100	95
BMA0316	hydrophobe/amphiphile efflux family protein	100	99	100	99
BMA0317	RND efflux system, outer membrane lipoprotein, NodT family	100	99	100	95
BMA0319	ISBma1, transposase, authentic point mutation	100	99	100	98
BMA0320	xanthine/uracil permease family protein, truncation	100	99	100	95
BMA0323	transcriptional regulator, AraC family	100	99	100	94
BMA0324	glutathione-dependent formaldehyde dehydrogenase	100	99	100	98
BMA0325	esterase, putative	100	99	100	91
BMA0326	cation ABC transporter, permease protein, putative	100	100	100	96
BMA0327	cation ABC transporter, ATP-binding protein, putative	100	99	99	94
BMA0328	cation ABC transporter, periplasmic cation-binding protein, putative	100	99	100	93
BMA0329	transcriptional regulator, putative	100	100	96	93
BMA0330	oxidoreductase, short-chain dehydrogenase/reductase family	100	99	100	96
BMA0331	carbohydrate kinase, PfkB family	100	99	100	94
BMA0333	maltose/mannitol ABC transporter, periplasmic maltose/mannitol-binding protein, putative	100	100	100	95

BMA0334	maltose/mannitol ABC transporter, permease protein, putative	100	99	100	95
BMA0335	maltose/mannitol ABC transporter, permease protein, putative	100	99	100	94
BMA0336	HAD-superfamily hydrolase	100	98	100	90
BMA0337	maltose/mannitol ABC transporter, ATP-binding protein, putative	100	99	100	95
BMA0338	conserved hypothetical protein	100	99	99	88
BMA0342	transcriptional regulator, putative	100	99	100	96
BMA0343	xylulokinase	100	99	100	93
BMA0344	mannitol dehydrogenase family protein	100	98	100	93
BMA0345	transcriptional regulator, LysR family	100	97	100	92
BMA0346	benzoylformate decarboxylase	100	99	100	93
BMA0347	aldehyde dehydrogenase family protein	100	100	100	95
BMA0348	2-dehydropantoate 2-reductase	100	99	100	89
BMA0350	4-hydroxybenzoate transporter, putative	100	99	100	94
BMA0351	tryptophan 2,3-dioxygenase family protein	100	99	100	96
BMA0352	kynureninase, putative	100	99	100	96
BMA0353	cyclase, putative	100	99	100	93
BMA0354	transcriptional regulator, AsnC family	100	100	100	97
BMA0355	flavin reductase domain protein	100	100	100	97
BMA0356	peptide methionine sulfoxide reductase	100	100	100	91
BMA0358	cyclopropane fatty acid synthase family protein	100	100	100	97
BMA0359	pyridoxamine 5'-phosphate oxidase	100	99	100	95
BMA0360	HesA/MoeB/ThiF family protein	100	100	100	96
BMA0362	conserved hypothetical protein	100	99	100	93
BMA0363	membrane protein, putative	100	99	100	93
BMA0365	N-acetylmuramoyl-L-alanine amidase	100	100	100	97
BMA0366	conserved hypothetical protein TIGR00150	100	100	100	94
BMA0367	iron-sulfur cluster binding protein, putative	100	99	100	95
BMA0368	methylated-DNA-protein-cysteine methyltransferase	100	99	100	91
BMA0369	tyrosine recombinase XerD	100	99	100	95
BMA0370	AMP-binding enzyme domain protein	100	99	100	89
BMA0371	ebsC protein, putative	100	100	100	98
BMA0372	membrane protein, putative	100	99	98	94
BMA0373	conserved hypothetical protein	100	100	100	98
BMA0374	UDP-N-acetylenolpyruvoylglucosamine reductase	100	99	100	91
BMA0375	ornithine carbamoyltransferase	100	100	100	98
BMA0377	ribosomal protein S20	100	100	100	100
BMA0378	integral membrane protein MviN	100	99	100	97
BMA0380	3-hydroxyacyl-CoA dehydrogenase	100	99	100	97
BMA0382	thymidylate synthase	100	100	100	95
BMA0383	sigma-54 dependent DNA-binding transcriptional regulator	100	99	100	96
BMA0387	dihydrofolate reductase	100	100	99	95
BMA0389	pmbA protein	100	99	100	98
BMA0390	conserved hypothetical protein	100	98	100	91
BMA0391	molybdopterin biosynthesis mog protein	100	99	100	96
BMA0392	oligoribonuclease	100	99	100	96
BMA0393	peptidase, M48 family	100	99	100	96
BMA0394	conserved hypothetical protein TIGR00157	100	98	100	94
BMA0395	lipoprotein, putative	100	100	100	90
BMA0396	CobD/CbiB family protein, putative	100	100	100	97

BMA0397	pyrophosphatase, MutT/nudix family	100	100	100	92
BMA0400	ribosomal protein L19	100	100	100	100
BMA0401	tRNA (guanine-N1)-methyltransferase	100	100	100	98
BMA0402	16S rRNA processing protein RimM	100	98	100	92
BMA0403	ribosomal protein S16	100	100	100	100
BMA0404	L-sorbosone dehydrogenase, authentic point mutation	100	99	100	91
BMA0406	radical SAM domain protein	100	99	100	92
BMA0407	leucine-responsive regulatory protein	100	99	100	99
BMA0408	D-amino acid dehydrogenase, small subunit	100	99	100	97
BMA0410	electron transfer flavoprotein, alpha subunit	100	100	100	99
BMA0411	electron transfer flavoprotein, beta subunit	100	100	100	100
BMA0412	D-methionine ABC transporter, periplasmic D-methionine-binding protein	100	98	100	91
BMA0413	D-methionine ABC transporter, permease protein	100	99	100	99
BMA0414	D-methionine ABC transporter, ATP-binding protein	100	100	100	98
BMA0415	hydrolase, alpha/beta fold family	100	98	100	86
BMA0416	histone deacetylase family protein	100	99	100	99
BMA0418	cysteine synthase B	100	99	100	97
BMA0420	competence protein ComE	100	100	100	94
BMA0421	ADP-L-glycero-D-mannoheptose-6-epimerase	100	99	100	97
BMA0422	ADP-heptose synthase	100	99	99	97
BMA0423	UDP-glucose 6-dehydrogenase	100	99	100	97
BMA0424	TPR domain protein	100	100	100	97
BMA0427	integration host factor, beta subunit	100	100	100	99
BMA0428	ribosomal protein S1	100	100	100	100
BMA0429	cytidylate kinase	100	99	100	97
BMA0430	prephenate dehydrogenase/3-phosphoshikimate 1-carboxyvinyltransferase	100	99	100	97
BMA0432	chorismate mutase/prephenate dehydratase	100	100	100	98
BMA0433	phosphoserine aminotransferase	100	100	97	95
BMA0434	conserved hypothetical protein	100	100	100	98
BMA0435	DNA gyrase, A subunit	100	99	100	98
BMA0436	OmpA family protein	100	100	100	98
BMA0437	3-demethylubiquinone-9 3-methyltransferase	100	100	99	97
BMA0438	phosphoglycolate phosphatase	100	98	100	94
BMA0443	ISBma1, transposase, truncation	100	98	100	98
BMA0444	conserved hypothetical protein	100	99	100	95
BMA0444.1	hypothetical protein	100	100	100	98
BMA0446	molybdate transport repressor domain protein	100	99	100	92
BMA0448	formate dehydrogenase, gamma subunit	100	100	100	94
BMA0449	formate dehydrogenase, beta subunit	100	100	100	93
BMA0450	formate dehydrogenase, alpha subunit	100	99	100	96
BMA0451	formate dehydrogenase, delta subunit	100	100	100	93
BMA0452	pentachlorophenol 4-monooxygenase, putative	100	99	100	93
BMA0453	TPR domain protein	100	99	100	92
BMA0454	citrate-proten symporter	100	99	100	98
BMA0455	glutamine amidotransferase, class I	100	99	100	91
BMA0457	amidase family protein	100	100	100	95
BMA0458	disulfide bond formation protein DsbB	100	100	100	95
BMA0460	xanthine dehydrogenase accessory protein XdhC, putative	100	100	99	94
BMA0461	adenosine deaminase	100	99	100	94

BMA0462	xanthine/uracil permease family protein	100	100	100	97
BMA0463	guanine deaminase	100	99	100	94
BMA0464	membrane protein, putative	100	99	100	95
BMA0465	lipoprotein, putative	100	100	100	94
BMA0466	aminopeptidase N	100	99	100	95
BMA0467	5-methyltetrahydropteroyltriglutamate--homocysteine S-methyltransferase	100	99	100	94
BMA0468	transcriptional regulator MetR	100	100	100	98
BMA0469	fructose-1,6-bisphosphatase	100	100	100	98
BMA0470	ISBma1, transposase	100	98	100	98
BMA0474	hypothetical protein	100	100	100	100
BMA0476	conserved hypothetical protein	100	99	99	91
BMA0477	TonB-dependent receptor	100	99	100	94
BMA0478	conserved hypothetical protein	100	99	100	87
BMA0480	chorismate mutase	100	98	100	90
BMA0486	isocitrate dehydrogenase, NADP-dependent	100	100	100	98
BMA0487	mulitcopper oxidase domain protein	100	98	100	88
BMA0489	conserved hypothetical protein, authentic point mutation	100	98	100	87
BMA0490	transcriptional regulator, ArsR family	100	100	100	91
BMA0492	thioesterase family protein	100	100	100	98
BMA0493	fumarate hydratase, class II	100	99	100	97
BMA0502	multidrug resistance protein NorM, putative	100	99	100	97
BMA0503	conserved hypothetical protein	100	100	100	98
BMA0506	creA protein	100	100	100	96
BMA0509	ferredoxin	100	99	100	95
BMA0510	nicotinate phosphoribosyltransferase	100	100	100	97
BMA0511	conserved hypothetical protein	100	100	100	95
BMA0512	membrane protein, putative	100	99	99	97
BMA0513	glyoxylate reductase	100	100	99	96
BMA0514	rmuC domain protein	100	99	100	96
BMA0515	acetyltransferase, GNAT family	100	99	100	92
BMA0516	membrane protein, putative	100	100	100	100
BMA0517	molybdopterin biosynthesis moeA protein	100	98	100	89
BMA0518	molybdopterin-guanine dinucleotide biosynthesis protein	100	99	100	90
BMA0519	molybdenum cofactor biosynthesis protein A	100	99	100	95
BMA0521	ribosomal large subunit pseudouridine synthase C	100	100	100	98
BMA0523	iron-sulfur cluster-binding protein, Rieske family	100	100	100	93
BMA0524	peptidase, U7 family protein	100	100	100	97
BMA0525	tetrapyrrole methylase family protein	100	99	100	98
BMA0526	Maf family protein	100	100	100	91
BMA0527	conserved hypothetical protein	100	100	100	94
BMA0528	ribosomal protein L32	100	100	100	96
BMA0529	fatty acid/phospholipid synthesis protein PlsX	100	100	100	98
BMA0530	3-oxoacyl-(acyl-carrier-protein) synthase III	100	99	100	96
BMA0531	malonyl CoA-acyl carrier protein transacylase	100	100	100	96
BMA0532	3-oxoacyl-(acyl-carrier-protein) reductase	100	100	100	96
BMA0533	acyl carrier protein	100	100	100	100
BMA0534	3-oxoacyl-(acyl-carrier-protein) synthase II	100	99	100	96
BMA0536	RNA polymerase sigma-H factor	100	99	100	98
BMA0537	sigma factor algU regulatory protein MucA, putative	100	99	100	94

BMA0538	sigma factor algU regulatory protein MucB	100	99	100	95
BMA0539	serine protease, MucD	100	99	100	96
BMA0540	conserved hypothetical protein	100	97	98	95
BMA0541	GTP-binding protein LepA	100	100	100	99
BMA0542	signal peptidase I	100	100	100	97
BMA0544	GTP-binding protein Era	100	100	100	98
BMA0545	Recombination protein O	100	99	100	95
BMA0546	pyridoxal phosphate biosynthetic protein PdxJ	100	99	100	97
BMA0547	holo-(acyl-carrier-protein) synthase	100	100	100	97
BMA0548	glycosyl hydrolase, family 3	100	99	100	93
BMA0549	sigma-54 dependent DNA-binding response regulator	100	100	100	97
BMA0550	translation elongation factor P	100	100	100	96
BMA0551	conserved hypothetical protein	100	98	98	92
BMA0552	excinuclease ABC, C subunit	100	99	100	91
BMA0553	CDP-diacylglycerol--glycerol-3-phosphate 3-phosphatidyltransferase	100	100	100	99
BMA0558	conserved hypothetical protein	100	100	100	97
BMA0559	transcriptional regulator, LysR family	100	100	100	93
BMA0560	conserved hypothetical protein	100	100	100	96
BMA0561	acetyltransferase, GNAT family	100	99	100	88
BMA0562	membrane protein, putative	100	99	100	98
BMA0563	conserved hypothetical protein	100	100	100	97
BMA0564	SCO1/SenC family protein	100	100	100	91
BMA0565	trehalose-phosphatase	100	98	100	91
BMA0566	alpha,alpha-trehalose-phosphate synthase	100	99	100	96
BMA0567	ABC transporter, permease/ATP-binding protein	100	99	100	96
BMA0568	glycosyl transferase, group 1 family protein	100	100	100	99
BMA0571	oxidoreductase, FAD-binding family protein	100	99	100	92
BMA0572	amino acid ABC transporter, periplasmic amino acid-binding protein, putative	100	100	100	96
BMA0575	hypothetical protein	100	99	100	96
BMA0576	YdjC-like family protein	100	98	100	95
BMA0577	radical SAM domain protein	100	100	100	99
BMA0578	syl transferase, group 2 family protein	100	99	100	97
BMA0580	luciferase-like monooxygenase	100	99	99	96
BMA0581	hydrolase, alpha/beta fold family	100	99	99	90
BMA0582	pyridoxal kinase	100	99	100	97
BMA0584	phospholipase C	100	99	100	97
BMA0588	arginine/ornithine ABC transporter, permease protein, putative	100	99	100	96
BMA0589	arginine/ornithine ABC transporter, ATP-binding protein, putative	100	99	100	96
BMA0590	transcriptional regulator, AraC family	100	99	100	98
BMA0591	succinylornithine transaminase	100	100	100	97
BMA0592	arginine N-succinyltransferase, alpha chain	100	98	100	96
BMA0593	arginine N-succinyltransferase, beta chain	100	99	100	95
BMA0594	succinylglutamate 5-semialdehyde dehydrogenase	100	99	100	93
BMA0595	succinylarginine dihydrolase	100	100	100	95
BMA0596	succinylglutamate desuccinylase	100	99	100	90
BMA0598	amino acid ABC transporter, periplasmic amino acid-binding protein	100	100	100	99
BMA0599	fatty acid desaturase domain protein	100	99	100	94
BMA0600	ubiquinol oxidase, subunit IV	100	100	100	97
BMA0603	ubiquinol oxidase, subunit II	100	98	100	96

BMA0606	Rrf2 family protein	100	98	99	95
BMA0607	conserved hypothetical protein	100	98	100	90
BMA0608	conserved hypothetical protein	100	98	100	87
BMA0609	NifU-like domain protein	100	100	100	87
BMA0610	cysteine desulfurase SufS	100	99	100	91
BMA0614	membrane protein, putative	100	99	100	93
BMA0617	methyl-accepting chemotaxis protein, putative	100	99	100	91
BMA0618	oxygen-independent coproporphyrinogen III oxidase	100	99	100	95
BMA0619	cyclic nucleotide-binding domain protein	100	100	100	99
BMA0620	membrane protein, putative	100	99	100	86
BMA0622	conserved hypothetical protein	100	99	100	95
BMA0623	peptidase, U32 family	100	99	100	97
BMA0624	conserved hypothetical protein	100	98	100	96
BMA0625	conserved hypothetical protein	99	100	98	90
BMA0626	conserved hypothetical protein	100	99	100	88
BMA0627	radical SAM domain protein	100	100	100	93
BMA0629	anaerobic ribonucleoside-triphosphate reductase	100	100	100	98
BMA0630	membrane protein, putative	100	99	100	89
BMA0631	membrane protein, putative	98	99	97	89
BMA0633	nitric oxide reductase norZ, putative	100	99	100	96
BMA0635	alkane-1 monooxygenase	100	99	100	95
BMA0636	deoxyribodipyrimidine photolyase	100	99	99	87
BMA0637	adenylylsulfate kinase	100	100	96	88
BMA0641	autoinducer-binding transcriptional regulator, LuxR family	100	99	100	97
BMA0643	conserved hypothetical protein	100	98	100	90
BMA0645	histidine ammonia-lyase	100	99	97	97
BMA0646	histidine utilization repressor	100	100	100	96
BMA0647	urocanate hydratase	100	100	100	95
BMA0649	imidazolonepropionase	100	99	100	96
BMA0650	formiminoglutamate deiminase	100	99	100	94
BMA0652	N-formylglutamate amidohydrolase	100	97	100	92
BMA0655	glutamine amidotransferase, class I	100	98	100	90
BMA0656	glutamine synthetase family protein	100	99	100	98
BMA0657	aminotransferase, class III, truncation	100	97	99	93
BMA0662	cys regulon transcriptional activator	100	100	100	98
BMA0663	sulfite reductase (NADPH) hemoprotein beta-component	100	100	100	98
BMA0664	conserved hypothetical protein	100	100	100	90
BMA0665	phosphoadenosine phosphosulfate reductase, putative	100	100	100	90
BMA0666	sulfate adenylyltransferase, subunit 2	100	100	100	98
BMA0667	sulfate adenylyltransferase, subunit 1	100	99	100	96
BMA0668	uroporphyrin-III C-methyltransferase	100	100	100	92
BMA0669	cbiX protein	100	99	98	88
BMA0670	membrane protein, putative	100	99	100	98
BMA0671	permease, putative	100	100	100	98
BMA0672	cytosol aminopeptidase	100	99	100	96
BMA0673	DNA polymerase III, chi subunit, putative	100	99	100	98
BMA0675	cytochrome c family protein	100	100	100	93
BMA0677	dihydroxy-acid dehydratase	100	100	100	97
BMA0678	isoleucine biosynthesis transcriptional activator	97	99	100	93

BMA0679	prolipoprotein diacylglyceryl transferase	100	100	100	98
BMA0680	conserved hypothetical protein	100	100	100	94
BMA0681	conserved hypothetical protein	100	100	100	95
BMA0682	conserved hypothetical protein	100	100	100	99
BMA0683	conserved hypothetical protein	100	100	100	96
BMA0685	vitamin B12 receptor BtuB, putative	100	99	100	86
BMA0686	iron compound ABC transporter, permease protein	100	100	100	95
BMA0687	iron compound ABC transporter, ATP-binding protein	100	100	99	91
BMA0688	nicotinate-nucleotide--dimethylbenzimidazole phosphoribosyltransferase	100	100	99	95
BMA0689	cobalamin-5-phosphate synthase	100	100	100	96
BMA0693	vitamin B12 transport protein BtuF, putative	100	99	100	89
BMA0694	alpha-ribazole-5`-phosphate phosphatase	100	99	100	90
BMA0695	cobalamin biosynthesis protein CobD	100	97	100	92
BMA0696	cobinamide kinase / cobinamide phosphate guanyltransferase	100	99	100	94
BMA0697	cobyric acid synthase CobQ	100	99	100	93
BMA0698	DoxD-like family protein	100	100	100	92
BMA0699	ParA family protein	100	100	100	98
BMA0700	aspartate 1-decarboxylase	100	100	100	97
BMA0701	pantoate--beta-alanine ligase	100	100	100	94
BMA0703	segregation and condensation protein A	100	99	100	98
BMA0704	conserved hypothetical protein	100	100	100	95
BMA0705	outer membrane protein, OMP85 family	100	99	100	93
BMA0706	conserved hypothetical protein	100	99	100	92
BMA0708	O-methyltransferase family protein	100	99	100	88
BMA0709	methionyl-tRNA synthetase	100	99	100	93
BMA0711	ompA family protein	100	100	100	99
BMA0712	ParA family protein	100	100	100	97
BMA0713	superoxide dismutase, Cu-Zn	100	99	100	96
BMA0714	deoxycytidine triphosphate deaminase, putative	100	100	100	99
BMA0715	Orn/Lys/Arg decarboxylase	100	100	100	99
BMA0716	hypothetical protein	100	100	100	95
BMA0717	HAD-superfamily hydrolase	100	100	99	90
BMA0718	argininosuccinate lyase	100	100	100	98
BMA0719	fimbrial usher protein	100	99	100	90
BMA0722	conserved hypothetical protein	100	99	100	87
BMA0723	conserved hypothetical protein	100	100	100	90
BMA0725	conserved hypothetical protein	100	99	100	96
BMA0726	methlytransferase, UbiE/COQ5 family	100	98	100	92
BMA0729	phosphoenolpyruvate carboxylase	100	99	100	96
BMA0730	porphobilinogen deaminase	100	100	100	96
BMA0731	uroporphyrinogen-III synthase HemD/uroporphyrinogen III methylase HemX, putative	100	99	100	95
BMA0732	conserved hypothetical protein	100	99	100	97
BMA0733	major facilitator family transporter	100	99	100	95
BMA0734	oxidoreductase, short-chain dehydrogenase/reductase family	100	100	100	93
BMA0735	aldehyde dehydrogenase family protein	100	100	100	95
BMA0736	inorganic pyrophosphatase	100	99	100	98
BMA0741	glutamine dependent NAD+ synthetase	100	99	100	95
BMA0742	nitrogen regulatory protein P-II	100	100	100	100
BMA0743	outer membrane porin, putative	100	100	100	97

BMA0744	amino acid ABC transporter, ATP-binding protein	100	100	100	96
BMA0745	amino acid ABC transporter, permease protein	100	100	100	99
BMA0746	amino acid ABC transporter, permease protein	100	99	100	97
BMA0747	amino acid ABC transporter, periplasmic amino acid-binding protein	100	100	100	97
BMA0749	hypothetical protein	100	100	100	92
BMA0750	DNA-binding response regulator OmpR	100	95	100	94
BMA0751	osmolarity sensor protein EnvZ, putative	100	99	100	94
BMA0752	hypothetical protein	100	100	100	97
BMA0754	ABC transporter, permease protein	100	100	100	98
BMA0755	ABC transporter, ATP-binding protein	100	100	100	99
BMA0759	glutathione S-transferase domain protein	100	98	100	95
BMA0760	proline/betaine transporter	100	99	100	97
BMA0761	conserved hypothetical protein	100	100	100	98
BMA0762	DNA polymerase III, epsilon subunit	100	100	98	94
BMA0763	ribonuclease HI	100	100	100	98
BMA0764	conserved hypothetical protein	100	100	100	99
BMA0765	hydroxyacylglutathione hydrolase	100	99	100	94
BMA0766	membrane-bound lytic murein transglycosylase D, putative	100	99	100	96
BMA0768	hypothetical protein	100	97	100	88
BMA0769	transcriptional regulator, AraC family	100	98	100	88
BMA0770	carbamoyl-phosphate synthase, small subunit	100	99	100	97
BMA0771	homoserine/threonine efflux protein, putative	100	100	100	98
BMA0772	carbamoyl-phosphate synthase, large subunit	100	100	100	98
BMA0773	transcription elongation factor GreA	100	100	100	93
BMA0774	membrane protein, putative	100	99	100	91
BMA0775	conserved hypothetical protein	100	100	100	96
BMA0776	ribosomal RNA large subunit methyltransferase J	100	100	100	97
BMA0777	cell division protein FtsH	100	99	100	99
BMA0778	dihydropteroate synthase	100	99	100	92
BMA0779	phosphoglucosamine mutase	100	99	100	97
BMA0780	phosphate ABC transporter, periplasmic phosphate-binding protein	100	100	100	97
BMA0781	phosphate ABC transporter, permease protein	100	100	100	97
BMA0782	phosphate ABC transporter, permease protein	100	99	100	96
BMA0783	phosphate ABC transporter, ATP-binding protein	100	100	100	99
BMA0784	phosphate transport system regulatory protein PhoU	100	100	100	98
BMA0785	DNA-binding response regulator PhoB	100	100	100	100
BMA0786	histidine protein kinase PhoR	100	100	100	98
BMA0788	polyphosphate kinase	100	99	100	98
BMA0789	exopolyphosphatase	100	99	100	98
BMA0791	conserved hypothetical protein	100	99	100	91
BMA0796	phosphohistidine phosphatase SixA	100	99	100	94
BMA0797	conserved hypothetical protein	100	100	100	99
BMA0798	hypothetical protein	100	98	97	94
BMA0799	lipoprotein, putative	100	98	100	88
BMA0800	DNA-damage-inducible protein F, truncation	100	99	100	93
BMA0802	acetyl-coenzyme A synthetase	100	99	100	92
BMA0803	lipoprotein NlpD, putative	100	100	100	91
BMA0804	conserved hypothetical protein, truncation	100	99	100	89
BMA0807	conserved domain protein	100	96	100	97

BMA0809	conserved hypothetical protein	100	97	100	95
BMA0811	transcriptional regulator, MerR family	100	99	100	86
BMA0824	poly(3-hydroxybutyrate) depolymerase	100	99	100	87
BMA0852	conserved hypothetical protein	100	99	100	93
BMA0854	conserved hypothetical protein	100	100	100	97
BMA0855	Rhs element Vgr protein	100	99	100	93
BMA0856	conserved hypothetical protein	100	99	100	90
BMA0863	lipoprotein, putative	100	99	100	88
BMA0864	H-NS histone family protein	100	100	100	98
BMA0866	hydrolase	100	98	99	88
BMA0867	major facilitator family transporter, authentic point mutation	100	99	100	92
BMA0870	transcriptional regulator, LysR family	100	100	100	98
BMA0873	conserved hypothetical protein	100	99	100	95
BMA0874	conserved hypothetical protein	100	100	100	92
BMA0875	fimbrial assembly chaperone, putative	100	99	100	88
BMA0877	conserved hypothetical protein	100	99	100	93
BMA0878	sensor histidine kinase/response regulator	100	99	100	89
BMA0879	DNA-binding response regulator	100	100	100	95
BMA0885	conserved hypothetical protein	100	99	100	93
BMA0886	acid phosphatase AcpA, putative	100	100	100	95
BMA0887	di-haem cytochrome c peroxidase family protein	100	99	99	95
BMA0888	conserved hypothetical protein	100	98	100	86
BMA0890	hypothetical protein	100	100	100	91
BMA0891	NUDIX domain protein	100	98	98	90
BMA0892	glutaminyl-tRNA synthetase	100	100	100	97
BMA0892.1	hypothetical protein	100	100	100	94
BMA0895	alanyl-tRNA synthetase	100	99	100	98
BMA0896	transcriptional regulator, LysR family	100	100	100	96
BMA0900	membrane protein, putative, truncation	100	97	100	89
BMA0903	pyrophosphatase, MutT/nudix family	100	100	100	91
BMA0904	conserved hypothetical protein	100	100	100	97
BMA0905	alcohol dehydrogenase, iron-containing family protein	100	99	100	96
BMA0906	thioesterase family protein	100	100	100	97
BMA0907	branched-chain amino acid ABC transporter, permease protein, putative	100	99	100	91
BMA0909	branched-chain amino acid ABC transporter, ATP-binding protein, putative	100	99	100	93
BMA0910	iolB protein	100	99	100	94
BMA0911	iolE protein	100	100	100	97
BMA0912	iolD protein	100	99	100	93
BMA0913	iolC protein	100	100	100	96
BMA0914	sugar ABC transporter, ATP-binding protein, putative	100	100	100	97
BMA0915	sugar ABC transporter, permease protein, putative	100	100	100	98
BMA0916	sugar ABC transporter, periplasmic sugar-binding protein, putative	100	100	100	97
BMA0918	myo-inositol dehydrogenase	100	100	100	93
BMA0919	myo-inositol dehydrogenase	100	99	100	96
BMA0920	conserved hypothetical protein	100	100	100	100
BMA0920.1	hypothetical protein	100	100	100	89
BMA0922	DNA-binding response regulator	100	100	100	100
BMA0923	LysM domain protein	100	99	100	92
BMA0924	sensor histidine kinase	100	99	100	96

BMA0925	lipoprotein, putative	100	100	100	90
BMA0926	UTP-glucose-1-phosphate uridylyltransferase	100	100	100	98
BMA0927	valyl-tRNA synthetase	100	99	100	97
BMA0928	DNA helicase II	100	99	100	98
BMA0929	MTA/SAH nucleosidase	100	100	100	93
BMA0930	fosmidomycin resistance protein	100	100	100	96
BMA0932	conserved hypothetical protein	100	99	100	95
BMA0933	membrane protein, putative	100	100	100	98
BMA0934	fusaric acid resistance protein, putative	100	99	100	96
BMA0936	transcriptional regulator	100	99	100	98
BMA0936.1	hypothetical protein	100	100	100	89
BMA0939	oxidoreductase, FAD-binding	100	100	100	95
BMA0941	flavodoxin	100	100	100	97
BMA0942	ribonuclease BN	100	99	100	94
BMA0943	conserved hypothetical protein	100	100	100	95
BMA0944	CBS domain protein	100	100	100	98
BMA0946	chorismate synthase	100	99	100	98
BMA0953	AcrB/AcrD/AcrF family protein	100	99	100	96
BMA0954	efflux transporter, RND family, MFP subunit	100	99	100	92
BMA0955	transcriptional regulator, TetR family	100	100	100	96
BMA0956	voltage-gated chloride channel/CBS domain protein	100	99	100	96
BMA0957	membrane protein, putative	100	100	100	95
BMA0958	transcriptional regulator, LysR family	100	99	100	95
BMA0960	conserved hypothetical protein	100	99	100	87
BMA0961	2-dehydro-3-deoxygluconokinase	100	99	100	93
BMA0962	major facilitator family transporter	100	99	100	96
BMA0963	glyoxylate reductase, authentic point mutation	100	99	100	89
BMA0965	transcriptional regulator, LacI family	100	99	100	93
BMA0966	membrane protein, putative	100	100	100	96
BMA0967	hypothetical protein	100	99	100	94
BMA0968	conserved hypothetical protein	100	98	98	94
BMA0969	polyhydroxybutyrate depolymerase, truncation	100	99	100	90
BMA0970	conserved hypothetical protein	100	98	100	91
BMA0972	transcriptional regulator, AsnC family	100	100	100	98
BMA0973	hypothetical protein	100	98	100	93
BMA0974	hypothetical protein	100	100	100	88
BMA0976	conserved hypothetical protein	100	99	100	98
BMA0977	conserved hypothetical protein	100	100	100	93
BMA0978	cyd operon protein YbgT	100	100	100	86
BMA0979	magnesium and cobalt transport protein CorA	100	99	100	95
BMA0980	conserved hypothetical protein	99	97	99	96
BMA0986	mechanosensitive ion channel family	100	99	100	95
BMA0987	regulatory protein nosR, putative	100	99	100	94
BMA0988	cytochrome c family protein	100	99	100	90
BMA0990	hypothetical protein	100	100	100	87
BMA0991	protein disulfide isomerase NosL, putative	100	98	100	90
BMA0992	copper ABC transporter, permease protein NosY, putative	100	99	100	94
BMA0993	copper ABC transporter, ATP-binding protein	100	100	100	91
BMA0994	copper ABC transporter, periplasmic copper-binding protein, putative	100	99	100	93

BMA0995	nitrous-oxide reductase	100	99	100	96
BMA0996	ApbE family protein	100	99	100	91
BMA0997	ABC transporter, ATP-binding protein	100	98	100	91
BMA0998	transporter, putative	100	99	100	93
BMA1001	dihydroxyacetone kinase	100	100	100	90
BMA1003	conserved domain protein	100	99	100	87
BMA1004	sodium/hydrogen exchanger	100	99	100	88
BMA1005	FAD binding domain protein	100	100	100	93
BMA1006	hypothetical protein	100	100	100	93
BMA1016	ISBma1, transposase	100	99	100	99
BMA1043	lipoprotein, putative	100	99	100	94
BMA1044	conserved hypothetical protein	100	99	100	96
BMA1046	conserved hypothetical protein	100	99	100	93
BMA1047	conserved domain protein	100	100	99	92
BMA1049	ATPase, AFG1 type	100	100	100	98
BMA1050	2-oxoglutarate dehydrogenase, E3 component, dihydrolipoamide dehydrogenase	100	99	100	97
BMA1051	2-oxoglutarate dehydrogenase, E2 component, dihydrolipoamide succinyltransferase	100	99	100	97
BMA1052	2-oxoglutarate dehydrogenase, E1 component	100	99	100	99
BMA1053	GTP-binding protein TypA	100	100	100	98
BMA1055	transcriptional regulator, MarR family	100	100	100	95
BMA1056	RND efflux system, outer membrane lipoprotein, NodT family	100	99	100	94
BMA1057	multidrug resistance protein, putative	100	99	100	94
BMA1058	drug resistance transporter, EmrB/QacA family	100	100	100	99
BMA1059	tRNA pseudouridine synthase B	100	100	100	94
BMA1060	ribosome-binding factor A	100	100	100	99
BMA1061	translation initiation factor IF-2	100	99	100	98
BMA1062	N utilization substance protein A	100	100	100	99
BMA1063	conserved hypothetical protein	100	99	100	99
BMA1064	RNA pseudouridylate synthase family protein	100	99	100	96
BMA1067	transcptional regulator, LysR family	100	100	100	97
BMA1069	conserved domain protein	100	100	100	100
BMA1072	hypothetical protein	100	100	100	95
BMA1073	conserved hypothetical protein	100	99	100	90
BMA1073.1	hypothetical protein	100	98	100	91
BMA1073.2	hypothetical protein	100	100	100	100
BMA1075	conserved hypothetical protein	100	100	100	96
BMA1080	lipoprotein, putative	100	99	100	97
BMA1085	conserved hypothetical protein	100	100	100	92
BMA1088	lipoprotein, putative	100	99	100	91
BMA1089	transcriptional regulator, merR family	100	100	99	95
BMA1090	integration host factor, alpha subunit	100	99	100	97
BMA1091	phenylalanyl-tRNA synthetase, beta subunit	100	100	100	96
BMA1092	phenylalanyl-tRNA synthetase, alpha subunit	100	100	100	99
BMA1093	ribosomal protein L20	100	100	100	100
BMA1094	ribosomal protein L35	100	100	100	100
BMA1095	translation initiation factor IF-3	100	99	100	99
BMA1096	threonyl-tRNA synthetase	100	100	100	98
BMA1098	GTP pyrophosphokinase	100	99	100	95
BMA1099	conserved hypothetical protein	100	99	98	96

BMA1100	hydrolase, alpha/beta fold family	100	99	100	93
BMA1103	transcriptional regulator, LysR family	100	99	100	94
BMA1104	conserved hypothetical protein	100	99	100	87
BMA1105	polysaccharide deacetylase family protein	100	97	99	89
BMA1106	oxidoreductase, short-chain dehydrogenase/reductase family	100	97	100	91
BMA1107	3-oxoadipate CoA-succinyl transferase beta subunit	100	100	100	96
BMA1108	3-oxoadipate CoA-succinyl transferase alpha subunit	100	100	100	97
BMA1109	transcriptional regulator, LuxR family	100	100	100	100
BMA1111	hypothetical protein	100	99	100	90
BMA1114	thioesterase family protein	100	100	99	97
BMA1115	oxidoreductase, short-chain dehydrogenase/reductase family	100	100	100	98
BMA1116	electron transfer flavoprotein-ubiquinone oxidoreductase, putative	100	100	100	98
BMA1125	outer membrane porin OpcP, putative	100	99	100	86
BMA1128	membrane protein, putative	100	100	100	88
BMA1129	chemotaxis protein CheW	100	100	100	94
BMA1130	methyl-accepting chemotaxis protein	100	99	100	90
BMA1134	long-chain-fatty-acid--CoA ligase, putative	100	100	100	94
BMA1136	conserved hypothetical protein	100	99	100	93
BMA1138	ABC transporter, periplasmic glycine/betaine-binding protein, putative	100	99	100	93
BMA1139	ABC transporter, permease protein	100	99	100	91
BMA1140	ABC transporter, ATP-binding protein	100	100	100	97
BMA1141	ABC transporter, permease protein	100	100	100	95
BMA1144	arginine/ornithine antiporter	100	100	100	96
BMA1145	arginine deiminase	100	100	100	97
BMA1146	ornithine carbamoyltransferase, catabolic	100	99	100	98
BMA1147	carbamate kinase	100	99	100	95
BMA1148	oxidoreductase, short-chain dehydrogenase/reductase family	100	99	100	92
BMA1149	conserved hypothetical protein	100	99	100	87
BMA1150	transcriptional regulator, LysR family	100	100	100	97
BMA1151	glutathione S-transferase , putative	100	98	100	94
BMA1154	transcriptional regulator, MarR family	100	100	99	93
BMA1157	precorrin-4 C11-methyltransferase	100	99	100	95
BMA1158	precorrin-6x reductase	100	99	100	89
BMA1159	cobalamin biosynthesis protein CbiD	100	99	99	96
BMA1160	precorrin-6Y C5,15-methyltransferase (decarboxylating)	100	100	98	93
BMA1161	nitrite/sulfite reductase domain protein	100	99	100	88
BMA1162	precorrin-8X methylmutase	100	99	100	94
BMA1163	precorrin-2 C20-methyltransferase	100	98	100	93
BMA1164	cbiG protein/precorrin-3B C17-methyltransferase	100	99	100	88
BMA1165	glycosyl hydrolase, family 18	100	99	100	95
BMA1165.1	hypothetical protein	100	99	99	90
BMA1167	carboxylesterase, putative	100	100	100	96
BMA1171	cobalamin synthesis protein/P47K family protein	100	99	100	91
BMA1172	high affinity nickel transporter	100	100	100	96
BMA1175	cob(I)alamin adenosyltransferase	100	100	100	98
BMA1177	conserved hypothetical protein	100	99	100	96
BMA1178	TonB-dependent siderophore receptor	100	99	100	94
BMA1179	l-ornithine 5-monooxygenase	100	98	100	89
BMA1183	cyclic peptide ABC transporter, ATP-binding protein	100	99	100	98

BMA1185	iron compound ABC transporter, periplasmic iron-compound-binding protein	100	99	99	92
BMA1186	ferric iron reductase protein FhuF	100	99	98	90
BMA1187	iron compound ABC transporter, permease protein	100	99	100	92
BMA1188	iron compound ABC transporter, ATP-binding protein	100	99	100	88
BMA1189	syringomycin biosynthesis enzyme, putative	100	100	100	95
BMA1190	mbtH-like protein	100	100	100	91
BMA1191	RNA polymerase sigma-70 factor, ECF subfamily	100	99	100	97
BMA1193	oxidoreductase, short chain dehydrogenase/reductase family	100	100	100	93
BMA1195	oxidoreductase, zinc-binding dehydrogenase family	100	99	100	94
BMA1196	ribose ABC transporter, permease protein	100	97	100	89
BMA1197	ribose ABC transporter, ATP-binding protein	100	99	100	92
BMA1198	ribose ABC transporter, periplasmic ribose-binding protein	100	99	100	92
BMA1199	transcriptional regulator, AraC family	100	98	100	94
BMA1205	cys regulon transcriptional activator	100	100	100	99
BMA1206	sulfate ABC transporter, ATP-binding protein	100	99	100	97
BMA1207	sulfate ABC transporter, permease protein	100	98	100	97
BMA1208	sulfate ABC transporter, permease protein	100	99	99	96
BMA1209	sulfate ABC transporter, periplasmic sulfate-binding protein	100	99	100	95
BMA1211	lexA repressor	100	100	100	99
BMA1215	universal stress protein family	100	100	100	96
BMA1218	nodulation ABC transporter NodI	100	99	100	94
BMA1219	ABC transporter, permease NodJ	100	100	100	96
BMA1220	hypothetical protein	100	98	99	96
BMA1221	transporter, putative	100	99	100	96
BMA1222	oxidoreductase, aldo/keto reductase family	100	99	100	96
BMA1223	conserved hypothetical protein	100	99	100	95
BMA1224	transcriptional regulator, putative	100	100	98	94
BMA1226	sodium:dicarboxylate symporter family protein	100	99	100	97
BMA1227	dihydrouridine synthase	100	99	100	95
BMA1230	ISBma1, transposase, interruption-N	100	99	100	98
BMA1233.1	hypothetical protein	100	98	100	95
BMA1236	molybdenum-pterin binding protein, putative	100	100	100	97
BMA1237	aliphatic sulfonate ABC transporter, ATP-binding protein	100	99	98	91
BMA1238	aliphatic sulfonate ABC transporter, permease protein	100	100	100	91
BMA1239	alkanesulfonate monooxygenase	100	100	100	94
BMA1241	hypothetical protein	100	100	100	97
BMA1242	hypothetical protein	100	100	100	97
BMA1244	enoyl-CoA hydratase/isomerase family protein	100	99	100	94
BMA1245	multidrug resistance protein, SMR family	100	100	100	91
BMA1246	conserved hypothetical protein	100	100	98	95
BMA1250	pyrophosphatase, MutT/nudix family	100	100	100	100
BMA1251	leucyl/phenylalanyl-tRNA--protein transferase	100	98	100	94
BMA1252	arginine-tRNA-protein transferase, putative	100	99	100	96
BMA1253	dihydroorotate dehydrogenase	100	99	100	95
BMA1254	amino acid ABC transporter, periplasmic amino acid-binding protein	100	100	100	97
BMA1255	amino acid ABC transporter, permease protein	100	100	100	97
BMA1258	oxidoreductase, Gfo/Idh/MocA family	100	99	100	93
BMA1259	transcriptional regulator, GntR family	100	100	100	99
BMA1260	ribose 5-phosphate isomerase	100	99	100	96

BMA1261	N-acetylmuramoyl-L-alanine amidase domain protein	100	99	100	95
BMA1262	RNA methyltransferase, TrmH family, group 3	100	100	100	97
BMA1263	ribonuclease R	100	99	100	97
BMA1269	hypothetical protein	100	99	100	87
BMA1270	sulfate permease family protein	100	99	100	94
BMA1271	conserved hypothetical protein	100	100	100	94
BMA1272	acetyltransferase, GNAT family	100	100	100	92
BMA1274	major facilitator family transporter	100	100	100	95
BMA1275	transcriptional regulator, TetR family	100	99	100	90
BMA1276	long-chain-fatty-acid--CoA ligase	100	100	100	95
BMA1277	conserved hypothetical protein	100	97	100	90
BMA1281	conserved hypothetical protein	100	99	100	91
BMA1282	sigma-54 dependent transcriptional regulator	100	99	100	98
BMA1283	conserved hypothetical protein	100	99	100	92
BMA1285	TPR domain protein	100	99	100	89
BMA1289	conserved hypothetical protein	100	100	100	97
BMA1290	type II/III secretion system protein	100	99	100	95
BMA1291	conserved hypothetical protein	100	99	100	89
BMA1292	conserved hypothetical protein	100	100	100	94
BMA1293	peptidase, putative	100	99	100	92
BMA1293.1	hypothetical protein	100	97	100	95
BMA1298	putrescine ABC transporter, permease protein	100	100	100	96
BMA1299	putrescine ABC transporter, permease protein	100	99	99	97
BMA1300	putrescine ABC transporter, ATP-binding protein	100	99	100	97
BMA1301	putrescine ABC transporter, periplasmic putrescine-binding protein	100	99	100	97
BMA1302	Mn2+/Fe2+ transporter, NRAMP family, authentic point mutation	100	99	100	93
BMA1303	conserved hypothetical protein	100	100	100	97
BMA1304	outer membrane protein, OmpW family	100	100	100	99
BMA1305	oxidoreductase, 2-nitropropane dioxygenase family	100	100	100	94
BMA1306	aldehyde dehydrogenase	100	99	100	94
BMA1308	ABC transporter, ATP-binding protein	100	99	100	99
BMA1309	AsmA family protein	100	99	100	89
BMA1310	ABC transporter, ATP-binding protein	100	99	100	91
BMA1311	membrane protein, putative	100	99	100	96
BMA1312	hypothetical protein	100	100	100	91
BMA1314	cystathionine beta-lyase	100	99	100	96
BMA1316	beta-ketothiolase	100	99	100	97
BMA1317	carbohydrate kinase, PfkB family	100	98	99	95
BMA1318	MiaB-like tRNA modifying enzyme YliG, TIGR01125	100	99	100	96
BMA1319	polyhydroxyalkanoate synthesis repressor PhaR	100	100	100	98
BMA1320	acetoacetyl-CoA reductase	100	100	100	98
BMA1321	acetyl-CoA acetyltransferase	100	99	100	98
BMA1322	poly(R)-hydroxyalkanoic acid synthase, class I	100	99	100	93
BMA1323	conserved hypothetical protein TIGR00726	100	100	100	89
BMA1324	ribosomal large subunit pseudouridine synthase D	100	99	100	94
BMA1325	competence lipoprotein ComL	100	98	100	98
BMA1326	ATP-dependent helicase, putative	100	99	100	93
BMA1327	conserved hypothetical protein	100	98	100	95
BMA1328	lipoprotein, putative	100	99	100	88

BMA1329	transcription accessory protein, TEX	100	99	100	97
BMA1331	potassium uptake protein	100	99	100	98
BMA1332	phosphoribosyl transferase domain protein	100	99	100	98
BMA1333	adenylosuccinate synthetase	100	99	100	98
BMA1335	ATP phosphoribosyltransferase regulatory subunit	100	100	100	98
BMA1337	ftsH protease activity modulator HflC	100	100	100	91
BMA1338	ftsH protease activity modulator HflK	100	99	100	93
BMA1339	GTP-binding protein HflX	100	99	100	97
BMA1340	hfq protein	100	100	100	100
BMA1341	GTPase family protein	100	100	100	98
BMA1342	lipoprotein, putative	100	99	100	97
BMA1343	conserved hypothetical protein	100	100	100	92
BMA1344	histidyl-tRNA synthetase	100	99	100	96
BMA1345	1-hydroxy-2-methyl-2-(E)-butenyl 4-diphosphate synthase	100	100	100	97
BMA1346	conserved hypothetical protein	100	99	100	87
BMA1347	radical SAM enzyme, Cfr family	100	100	100	98
BMA1348	nucleoside diphosphate kinase	100	100	100	97
BMA1349	membrane protein, putative	100	99	100	99
BMA1350	ribosomal RNA large subunit (uracil-5-)-methyltransferase	100	99	100	95
BMA1350.1	endonuclease/exonuclease/phosphatase family superfamily	100	100	99	90
BMA1353	conserved hypothetical protein	100	100	100	97
BMA1354	RNA polymerase sigma factor	100	100	100	96
BMA1355	lipoprotein NlpD, putative	100	100	100	93
BMA1356	protein-L-isoaspartate O-methyltransferase	100	99	100	90
BMA1357	acid phosphatase SurE	100	99	100	95
BMA1358	CAIB/BAIF family protein	100	99	100	96
BMA1359	recombination protein RecR	100	99	100	98
BMA1360	conserved hypothetical protein TIGR00103	100	100	100	97
BMA1364	thioredoxin	100	100	100	99
BMA1365	transcription termination factor Rho	100	99	100	99
BMA1366	transcriptional regulator, MerR family	100	99	100	95
BMA1367	multidrug resistance protein, putative	100	98	100	94
BMA1368	conserved hypothetical protein	100	98	100	95
BMA1369	ribosomal protein L31	100	100	100	97
BMA1370	membrane protein, putative	100	99	100	95
BMA1371	multidrug resistance protein NorM, putative	100	99	100	93
BMA1373	carboxymuconolactone decarboxylase family protein	100	99	100	92
BMA1374	transcriptional regulator, MerR family	100	100	99	88
BMA1376	conserved hypothetical protein	100	99	100	94
BMA1377	ATP-dependent Clp protease, ATP-binding subunit ClpB	100	99	100	98
BMA1378	Rrf2 family protein	100	100	100	92
BMA1379	conserved hypothetical protein	100	100	100	95
BMA1380	molybdopterin converting factor, subunit 2	100	99	100	92
BMA1381	molybdopterin converting factor, subunit 1	100	100	100	98
BMA1382	molybdopterin biosynthesis moeA protein	100	98	100	92
BMA1384	threonine synthase	100	100	100	95
BMA1385	homoserine dehydrogenase	100	100	100	97
BMA1387	conserved hypothetical protein	100	100	100	96
BMA1388	dolichyl-phosphate-mannose-protein mannosyltransferase family protein	100	99	100	95

BMA1389	membrane protein, putative	100	100	100	100
BMA1390	aminotransferase, DegT/DnrJ/EryC1/StrS family	100	99	100	96
BMA1391	glycosyl transferase, group 2 family protein	100	100	100	99
BMA1392	formyltransferase, putative	100	100	100	93
BMA1393	conserved hypothetical protein	100	99	100	98
BMA1394	polysaccharide deacetylase family protein	100	99	100	96
BMA1395	antioxidant, AhpC/Tsa family	100	98	100	93
BMA1396	PhoH family protein	100	99	100	95
BMA1398	phosphate transporter family protein	100	100	100	100
BMA1399	conserved hypothetical protein	100	100	100	99
BMA1400	replicative DNA helicase	100	100	100	99
BMA1401	ribosomal protein L9	100	100	100	97
BMA1402	ribosomal protein S18	100	100	100	100
BMA1403	primosomal replication protein n	100	98	100	96
BMA1404	ribosomal protein S6	100	100	100	100
BMA1407	Ser/Thr protein phosphatase family protein	100	100	98	95
BMA1408	cytochrome c oxidase family protein	100	99	100	90
BMA1413	asparaginase family protein	100	100	100	94
BMA1414	phosphate starvation-inducible protein	100	100	100	97
BMA1416	lipoprotein, putative	100	100	100	98
BMA1417	glycosyl transferase, group 1 family protein	100	100	100	98
BMA1418	conserved hypothetical protein	100	99	97	90
BMA1419	mechanosensitive ion channel family protein	100	99	100	93
BMA1420	hypothetical protein	100	99	100	99
BMA1421	ankyrin repeat protein	100	96	100	93
BMA1422	hydrolase, TatD family	100	100	100	93
BMA1423	phosphinothricin acetyltransferase	100	98	100	98
BMA1424	DNA polymerase III, delta prime subunit	100	100	100	97
BMA1425	thymidylate kinase	100	99	100	96
BMA1426	lipoprotein, putative	100	100	100	94
BMA1427	conserved hypothetical protein	100	100	100	94
BMA1428	conserved hypothetical protein	100	100	100	95
BMA1429	acetyltransferase, GNAT family	100	100	100	95
BMA1430	esterase	100	100	100	95
BMA1431	oxidoreductase, zinc-binding dehydrogenase family protein	100	99	100	93
BMA1432	conserved hypothetical protein	100	100	100	95
BMA1433	conserved hypothetical protein	100	100	100	98
BMA1435	hypothetical protein	100	99	100	93
BMA1436	thiolase family protein	100	99	100	97
BMA1437	pyrazinamidase/nicotinamidase	100	99	100	92
BMA1438	fatty oxidation complex, alpha subunit, putative	100	99	100	95
BMA1439	medium-chain-fatty-acid--CoA ligase	100	99	100	96
BMA1440	conserved hypothetical protein	100	99	100	95
BMA1441	methionine-R-sulfoxide reductase	100	100	100	96
BMA1442	intracellular septation protein A	100	100	100	98
BMA1443	BolA-like protein	100	98	100	91
BMA1444	peptidyl-prolyl cis-trans isomerase domain protein	100	99	100	98
BMA1446	phosphoribosylformylglycinamidine synthase	100	99	100	96
BMA1447	D-amino acid dehydrogenase, small subunit, putative	100	99	100	95

BMA1448	carbohydrate kinase, putative	100	99	100	93
BMA1449	glucose-6-phosphate isomerase	100	99	100	97
BMA1450	ABC transporter, ATP-binding protein	100	99	100	95
BMA1451	acyl-CoA thioesterase I	100	99	100	93
BMA1453	peptidyl-prolyl cis-trans isomerse D, putative	100	99	100	95
BMA1458	hypothetical protein	100	100	100	88
BMA1460	ribosomal subunit interface protein YfiA, putative	100	100	99	87
BMA1463	ATP-dependent protease La	100	100	100	99
BMA1464	ATP-dependent Clp protease, ATP-binding subunit ClpX	100	99	100	99
BMA1465	ATP-dependent Clp protease, proteolytic subunit ClpP	100	99	100	99
BMA1466	trigger factor	100	99	100	96
BMA1468	glycerate kinase 1	100	99	100	89
BMA1469	transcriptional regulator, MarR family	100	100	100	93
BMA1471	2-dehydropantoate 2-reductase	100	98	100	91
BMA1472	transcriptional regulator, LuxR family	100	98	100	91
BMA1473	outer membrane porin, putative	100	99	100	86
BMA1474	membrane protein, putative	100	99	100	87
BMA1475	histone deacetylase family protein	100	99	100	93
BMA1476	endonuclease Nuc	100	100	100	89
BMA1482	phytoene synthase, putative	100	98	99	87
BMA1483	twin-arginine translocation pathway signal sequence domain protein	100	98	100	91
BMA1484	conserved hypothetical protein	100	99	100	91
BMA1484.1	hypothetical protein	100	100	100	94
BMA1485	DNA-binding response regulator RisA	100	100	100	100
BMA1486	sensor histidine kinase RisS	100	99	100	97
BMA1487	antioxidant, AhpC/Tsa family	100	100	100	100
BMA1488	alkyl hydroperoxide reductase D	100	100	100	94
BMA1489	2C-methyl-D-erythritol 2,4-cyclodiphosphate synthase	100	100	100	98
BMA1490	4-diphosphocytidyl-2C-methyl-D-erythritol synthase	100	99	100	97
BMA1491	transcription-repair coupling factor	100	99	100	98
BMA1493	acetylornithine deacetylase	100	100	100	96
BMA1494	threonine dehydratase catabolic	100	99	100	95
BMA1497	penicillin-binding protein, 1A family	100	99	100	96
BMA1498	PspA/IM30 family protein	100	99	100	99
BMA1499	hypothetical protein	100	99	100	90
BMA1501	large conductance mechanosensitive channel protein	100	100	100	100
BMA1502	hypothetical protein	100	100	100	99
BMA1504	ureidoglycolate hydrolase	100	100	100	89
BMA1505	allantoicase	100	100	100	94
BMA1507	conserved hypothetical protein	100	99	100	90
BMA1508	polysaccharide deacetylase family protein	100	99	100	97
BMA1509	hydantoin racemase	100	96	95	94
BMA1510	NCS1 nucleoside transporter family protein	100	99	100	95
BMA1511	transcriptional regulator, GntR family	100	100	100	96
BMA1512	ISBma1, transposase	100	99	100	99
BMA1513	muramoyltetrapeptide carboxypeptidase	100	100	100	95
BMA1514	cytidine/deoxycytidylate deaminase family protein	100	100	100	96
BMA1515	conserved hypothetical protein	100	100	100	98
BMA1522	GMP synthase	100	99	100	98

BMA1524	inosine-5'-monophosphate dehydrogenase	100	100	100	98
BMA1526	conserved hypothetical protein	100	98	100	90
BMA1528	conserved hypothetical protein	100	99	100	90
BMA1529	membrane protein, putative	100	100	100	97
BMA1530	TGS domain protein	100	100	100	91
BMA1531	conserved hypothetical protein	100	100	100	98
BMA1532	SsrA-binding protein	100	100	100	99
BMA1533	SPFH domain/band 7 family protein	100	100	100	98
BMA1534	conserved hypothetical protein	100	100	100	94
BMA1535	phosphoenolpyruvate synthase	100	100	100	99
BMA1537	phytochelatin synthase, putative	100	99	99	89
BMA1538	serine protease, subtilase family	100	99	100	91
BMA1539	conserved hypothetical protein	100	100	100	98
BMA1540	RNA methyltransferase, TrmH family	100	100	99	93
BMA1541	ribonuclease HII	100	99	100	96
BMA1542	lipid-A-disaccharide synthase	100	98	100	95
BMA1543	acyl-(acyl-carrier-protein)--UDP-N-acetylglucosamine O-acyltransferase	100	100	100	98
BMA1544	beta-hydroxyacyl-(acyl-carrier-protein) dehydratase FabZ	100	100	100	98
BMA1545	UDP-3-O-3-hydroxymyristoyl glucosamine N-acyltransferase	100	99	100	95
BMA1546	outer membrane protein, OmpH/HlpA family	100	99	100	98
BMA1547	outer membrane protein, OMP85 family	100	99	100	99
BMA1548	membrane-associated zinc metalloprotease, putative	100	99	100	95
BMA1549	1-deoxy-D-xylulose 5-phosphate reductoisomerase	100	99	100	96
BMA1550	phosphatidate cytidylyltransferase	100	99	100	95
BMA1551	undecaprenyl diphosphate synthase	100	99	100	98
BMA1552	ribosome recycling factor	100	100	100	95
BMA1553	uridylate kinase	100	100	100	99
BMA1554	translation elongation factor Ts	100	100	100	97
BMA1555	ribosomal protein S2	100	100	100	97
BMA1556	methionine aminopeptidase, type I	100	100	100	97
BMA1557	protein-P-II uridylyltransferase	100	99	100	97
BMA1558	pseudouridine synthase family protein	100	99	100	97
BMA1559	polypeptide deformylase	100	100	100	97
BMA1560	DNA ligase, NAD-dependent	100	99	100	95
BMA1562	conserved hypothetical protein	100	98	100	92
BMA1563	chromosome segregation protein SMC	100	99	100	97
BMA1564	membrane protein, putative	100	98	100	94
BMA1565	aromatic aminotransferase, putative	100	99	100	93
BMA1566	2,3,4,5-tetrahydropyridine-2,6-dicarboxylate N-succinyltransferase	100	100	100	99
BMA1567	conserved hypothetical protein	100	98	100	96
BMA1568	succinyl-diaminopimelate desuccinylase	100	99	100	97
BMA1569	hemK family protein	100	100	100	96
BMA1570	cardiolipin synthetase, putative	100	100	100	96
BMA1571	glutathione peroxidase	100	100	100	98
BMA1572	ABC transporter, ATP-binding protein	100	99	100	96
BMA1574	DNA repair protein RadA	100	100	100	99
BMA1575	alanine racemase	100	100	100	96
BMA1576	transporter, putative	100	100	100	97
BMA1577	phosphomethylpyrimidine kinase	100	99	100	93

BMA1579	conserved hypothetical protein	100	99	100	86
BMA1580	phage SPO1 DNA polymerase domain protein	100	98	100	95
BMA1581	ribosomal-protein-alanine acetyltransferase	100	100	99	96
BMA1582	glycoprotease family protein	100	100	100	90
BMA1583	acyl-CoA-binding protein	100	100	100	97
BMA1584	ATP-dependent RNA helicase RhlE, putative	100	100	100	95
BMA1586	isocitrate lyase	100	100	100	99
BMA1587	universal stress protein family	100	100	100	97
BMA1588	transcriptional regulator, LysR family	100	100	100	98
BMA1589	haloacid dehalogenase, type II	100	100	100	94
BMA1590	malate synthase A	100	99	100	96
BMA1593	protein of unknown function	100	100	99	92
BMA1594	transcriptional regulator, AraC family	100	100	100	94
BMA1596	acetyltransferase, GNAT family	100	99	100	96
BMA1600	glutamyl-tRNA synthetase	100	99	100	94
BMA1601	patatin-like phospholipase	100	99	100	94
BMA1602	NLP/P60 family protein	100	99	100	95
BMA1603	hypothetical protein	100	96	100	90
BMA1604	ABC transporter, ATP-binding protein	100	100	100	93
BMA1605	ABC transporter, permease protein	100	99	100	96
BMA1606	ABC transporter, permease protein	100	100	100	98
BMA1607	ABC transporter, periplasmic substrate-binding protein	100	99	100	91
BMA1608	enoyl-(acyl-carrier-protein) reductase	100	100	100	98
BMA1609	conserved hypothetical protein	100	100	100	96
BMA1612	molybdopterin oxidoreductase family protein	100	99	100	92
BMA1617	transcriptional regulator, TetR family	100	99	100	91
BMA1618	methionine aminopeptidase, type I	100	100	100	91
BMA1619	conserved hypothetical protein	100	100	100	94
BMA1620	argininosuccinate lyase domain protein	100	99	100	89
BMA1621	cysteine synthase/cystathionine beta-synthase family protein	100	100	100	95
BMA1622	kinase, putative	100	100	100	89
BMA1623	sulfate adenylyltransferase, subunit 2	100	100	100	96
BMA1624	glycosyl transferase, group 2 family protein	100	98	100	91
BMA1625	phosphoserine aminotransferase	100	99	100	92
BMA1626	acyltransferase family protein	100	99	100	94
BMA1627	conserved hypothetical protein	100	96	100	87
BMA1628	transketolase, C-terminal subunit	100	99	100	90
BMA1629	transketolase, N-terminal subunit	100	100	100	93
BMA1630	hypothetical protein	100	99	97	88
BMA1631	hypothetical protein	100	99	100	95
BMA1632	hypothetical protein	100	98	100	96
BMA1633	dioxygenase, TauD/TfdA	100	99	100	95
BMA1634	conserved hypothetical protein	100	99	100	89
BMA1636	conserved hypothetical protein	100	99	100	93
BMA1637	membrane protein, putative	100	99	100	92
BMA1638	peptide synthetase-domain protein	100	99	100	91
BMA1643	multidrug efflux RND membrane fusion protein MexE	100	98	100	87
BMA1644	AcrB/AcrD/AcrF family protein	100	99	100	96
BMA1645	hypothetical protein	100	99	100	88

BMA1646	hypothetical protein	100	100	100	98
BMA1647	outer membrane autotransporter domain protein	100	99	99	88
BMA1648	conserved hypothetical protein	100	98	99	87
BMA1652	aspartokinase, alpha and beta subunits	100	100	100	99
BMA1653	cell cycle protein mesJ	100	99	100	88
BMA1654	acetyl-CoA carboxylase, carboxyl transferase, alpha subunit	100	100	100	97
BMA1655	DNA-3-methyladenine glycosylase	100	99	100	99
BMA1656	cysteinyl-tRNA synthetase	100	100	100	96
BMA1657	TPR domain protein	100	100	100	94
BMA1658	peptidyl-prolyl cis-trans isomerase A	100	100	100	99
BMA1659	peptidyl-prolyl cis-trans isomerase B	100	100	100	99
BMA1660	UDP-2,3-diacylglucosamine hydrolase	100	99	100	94
BMA1662	serine O-acetyltransferase	100	100	100	94
BMA1663	RNA methyltransferase, TrmH family, group 1	100	99	99	92
BMA1664	inositol-1-monophosphatase	100	100	100	97
BMA1671	DNA mismatch repair protein MutS	100	99	100	95
BMA1672	conserved hypothetical protein	100	99	100	87
BMA1674	conserved hypothetical protein	100	99	100	94
BMA1676	metallo-beta-lactamase family protein	100	99	100	96
BMA1677	lipoprotein, putative	100	99	100	96
BMA1678	dihydrodipicolinate synthase	100	100	100	97
BMA1679	conserved hypothetical protein	100	98	100	88
BMA1680	tryptophanyl-tRNA synthetase	100	100	100	99
BMA1681	peptidase, M50 family	100	99	100	98
BMA1682	Sua5/YciO/YrdC/YwlC family protein	100	100	100	98
BMA1683	phosphoesterase, putative	100	100	100	96
BMA1684	hydrolase, alpha/beta fold family	100	99	100	95
BMA1685	conserved hypothetical protein	100	100	100	89
BMA1686	bacterial transferase hexapeptide repeat protein	100	98	100	96
BMA1687	33 kda chaperonin	100	99	100	95
BMA1688	septum formation initiator family protein	100	100	100	91
BMA1689	enolase	100	100	100	99
BMA1690	3-deoxy-8-phosphooctulonate synthase	100	100	100	98
BMA1691	CTP synthase	100	100	100	98
BMA1692	conserved hypothetical protein	100	99	100	96
BMA1693	DNA internalization-related competence protein ComEC/Rec2	100	99	100	87
BMA1694	hydrolase, TatD family	100	99	100	94
BMA1695	ABC transporter, ATP-binding protein	100	99	100	96
BMA1696	lipoprotein releasing system transmembrane protein, putative	100	99	100	99
BMA1698	single-stranded-DNA-specific exonuclease RecJ	100	100	100	98
BMA1699	peptide chain release factor 2, programmed frameshift	100	98	100	90
BMA1700	lysyl-tRNA synthetase	100	99	100	96
BMA1701	conserved hypothetical protein	100	100	100	95
BMA1702	conserved hypothetical protein	100	100	100	97
BMA1703	ferredoxin, 2Fe-2S	100	100	100	95
BMA1704	chaperone protein HscA	100	99	100	96
BMA1705	co-chaperone Hsc20	100	100	100	95
BMA1706	iron-sulfur cluster assembly accessory protein	100	99	100	100
BMA1707	FeS cluster assembly scaffold IscU	100	100	100	97

BMA1708	cysteine desulfurase	100	99	100	98
BMA1709	iron-sulfur cluster assembly transcription factor IscR	100	100	100	98
BMA1710	low molecular weight protein-tyrosine-phosphatase	100	100	100	93
BMA1711	hypothetical protein	100	100	100	89
BMA1712	iron-sulfur cluster-binding protein, putative	100	99	100	94
BMA1713	conserved hypothetical protein	100	99	100	95
BMA1714	transcriptional regulator, IclR family	100	99	100	99
BMA1716	phasin family protein	100	100	100	98
BMA1717	D-alanyl-D-alanine endopeptidase, putative	100	99	100	96
BMA1719	pyruvate dehydrogenase, E3 component, dihydrolipoamide dehydrogenase	100	99	100	94
BMA1721	pyruvate dehydrogenase, E1 component	100	100	100	96
BMA1722	sensory box histidine kinase	100	99	100	97
BMA1723	DNA-binding response regulator, LuxR family	100	100	100	98
BMA1724	methylenetetrahydrofolate dehydrogenase/methenyltetrahydrofolate cyclohydrolase	100	99	100	98
BMA1726	oligopeptidase A	100	99	100	96
BMA1727	DNA polymerase IV	100	98	96	91
BMA1729	nitrate/nitrite transporter	100	99	100	97
BMA1730	nitrate/nitrite transporter NarK	100	99	100	97
BMA1731	nitrate reductase, alpha subunit	100	99	100	98
BMA1732	nitrate reductase, beta subunit	100	99	100	98
BMA1733	nitrate reductase, delta subunit	100	99	100	95
BMA1734	nitrate reductase, gamma subunit	100	100	100	98
BMA1735	nitrate/nitrite sensory protein NarX, putative	100	100	100	96
BMA1736	DNA-binding response regulator NarL	100	100	100	96
BMA1738	exodeoxyribonuclease III	100	99	100	97
BMA1740	nitrogen regulation protein NR(I)	100	100	100	96
BMA1741	nitrogen regulation protein	100	99	100	97
BMA1743	glutamine synthetase, type I	100	100	100	99
BMA1744	conserved hypothetical protein	100	100	100	97
BMA1745	molybdopterin-binding protein	100	100	99	97
BMA1746	membrane protein, putative	100	99	100	96
BMA1748	membrane protein, putative	100	99	100	96
BMA1749	conserved hypothetical protein	100	100	100	97
BMA1750	ATP-dependent helicase HrpA	100	99	100	95
BMA1751	amino-acid N-acetyltransferase	100	100	100	99
BMA1752	conserved hypothetical protein	100	100	99	97
BMA1753	major facilitator family transporter	100	99	100	93
BMA1755	acyl-CoA dehydrogenase domain protein	100	98	100	92
BMA1756	transcriptional regulator, LysR family	100	99	100	96
BMA1761	lipoprotein, putative	100	100	100	97
BMA1762	lipoprotein, putative	100	100	100	89
BMA1763	membrane protein, putative	100	99	100	93
BMA1767	peptidyl-tRNA hydrolase, putative	100	100	100	94
BMA1769	exodeoxyribonuclease V, beta subunit	100	99	100	93
BMA1769.1	exodeoxyribonuclease V, gamma subunit	100	99	100	94
BMA1772	membrane protein, putative	100	99	100	96
BMA1774	amino acid permease	100	99	100	96
BMA1775	hypothetical protein	100	98	100	94
BMA1777	iron compound ABC transporter, periplasmic iron-compound-binding protein, putative	100	99	100	95

BMA1778	iron compound ABC transporter, permease protein, putative	100	99	100	92
BMA1779	iron compound ABC transporter, ATP-binding protein, putative	100	99	100	96
BMA1780	transcriptional regulator, AsnC family	100	100	100	99
BMA1783	conserved hypothetical protein	100	98	100	92
BMA1784	Rrf2 family protein	100	100	100	92
BMA1785	hypothetical protein	99	98	99	86
BMA1786	conserved hypothetical protein	100	99	100	95
BMA1794	conserved hypothetical protein	100	100	100	98
BMA1796	membrane protein, putative	100	100	100	97
BMA1797	conserved domain protein	100	99	100	91
BMA1798	conserved hypothetical protein	100	99	100	95
BMA1799	conserved hypothetical protein	100	100	100	96
BMA1801	aromatic aminotransferase	100	99	100	96
BMA1803	enoyl-CoA hydratase/isomerase family protein	100	100	100	96
BMA1804	phosphoglycerate mutase, putative	100	98	100	97
BMA1805	phosphotransferase enzyme family protein	100	100	100	93
BMA1806	acyl-CoA dehydrogenase domain protein	100	99	100	95
BMA1809	MaoC family protein	100	100	100	93
BMA1810	MoaC domain protein	100	99	98	93
BMA1811	acyl-CoA dehydrogenase domain protein	100	99	100	92
BMA1812	acyl-CoA dehydrogenase domain protein	100	99	100	96
BMA1813	conserved hypothetical protein	100	100	100	97
BMA1814	pyrophosphatase, MutT/nudix family	100	99	100	96
BMA1815	membrane protein, putative	100	99	100	93
BMA1816	NADH dehydrogenase I, N subunit	100	100	100	98
BMA1817	NADH dehydrogenase I, M subunit	100	100	100	97
BMA1819	NADH dehydrogenase I, K subunit	100	100	100	100
BMA1820	NADH dehydrogenase I, J subunit	100	100	100	97
BMA1821	NADH dehydrogenase I, I subunit	100	100	100	98
BMA1822	NADH dehydrogenase I, H subunit	100	100	100	99
BMA1823	NADH dehydrogenase I, G subunit	100	99	100	96
BMA1824	NADH dehydrogenase I, F subunit	100	99	100	99
BMA1825	NADH dehydrogenase I, E subunit	100	100	100	100
BMA1826	NADH dehydrogenase I, D subunit	100	100	100	99
BMA1827	NADH dehydrogenase I, C subunit	100	100	100	98
BMA1828	NADH-quinone oxidoreductase, B subunit	100	100	100	100
BMA1829	NADH dehydrogenase I, A subunit	100	99	100	99
BMA1831	preprotein translocase, SecG subunit	100	100	100	98
BMA1832	triosephosphate isomerase	100	99	100	91
BMA1833	oxidoreductase, zinc-binding dehydrogenase family protein	100	100	100	95
BMA1834	polyribonucleotide nucleotidyltransferase	100	99	100	98
BMA1835	ribosomal protein S15	100	100	100	100
BMA1836	amino acid-binding protein, putative	100	100	99	92
BMA1838	lipoprotein, putative	100	100	100	95
BMA1839	carbonic anhydrase	100	99	100	95
BMA1840	sulfate transporter, putative	100	99	100	94
BMA1844	CDP-diacylglycerol--serine O-phosphatidyltransferse	100	99	100	99
BMA1845	phosphatidylserine decarboxylase-related protein	100	99	100	98
BMA1846	ketol-acid reductoisomerase	100	100	100	99

BMA1847	acetolactate synthase, small subunit	100	100	100	100
BMA1848	acetolactate synthase, large subunit, biosynthetic type	100	99	100	98
BMA1850	RNA polymerase sigma-70 factor, ECF subfamily	100	99	96	98
BMA1851	conserved hypothetical protein	100	99	100	95
BMA1852	conserved hypothetical protein	100	99	100	92
BMA1853	conserved hypothetical protein	100	98	99	88
BMA1854	Ser/Thr protein phosphatase family protein	100	100	100	97
BMA1855	glycosyl transferase, group 1 family protein	100	100	100	97
BMA1856	diacylglycerol kinase	100	99	100	93
BMA1857	transcriptional reguator, TetR family	100	99	100	99
BMA1858	decarboxylase family protein	100	100	100	96
BMA1859	glucose 1-dehydrogenase	100	99	100	94
BMA1861	membrane protein, putative	100	99	100	91
BMA1862	major facilitator family transporter	100	99	100	90
BMA1863	ISBma1, transposase	100	98	100	98
BMA1864	amidase family protein	100	99	100	92
BMA1865	conserved hypothetical protein	100	99	100	92
BMA1866	conserved hypothetical protein	100	100	100	95
BMA1867	protozoan/cyanobacterial globin family protein	100	98	100	94
BMA1869	conserved hypothetical protein	100	100	100	96
BMA1870	sugE protein	100	100	100	93
BMA1872	DNA-binding response regulator KdpE	100	100	100	98
BMA1874	K+-transporting ATPase, C subunit	100	100	99	94
BMA1875	K+-transporting ATPase, B subunit	100	99	100	96
BMA1876	K+-transporting ATPase, A subunit	100	99	100	93
BMA1877	potassium-transporting ATPase, KdpF subunit	100	100	100	100
BMA1879	quinone oxidoreductase	100	100	100	95
BMA1881	methylglyoxal synthase	100	100	100	96
BMA1882	oxidoreductase, short chain dehydrogenase/reductase family	100	99	100	96
BMA1883	uracil phosphoribosyltransferase	100	100	100	99
BMA1884	conserved hypothetical protein TIGR01033	100	100	100	97
BMA1885	phosphoribosylamine--glycine ligase	100	99	100	98
BMA1886	coproporphyrinogen III oxidase, aerobic	100	99	100	95
BMA1887	nicotinate (nicotinamide) nucleotide adenylyltransferase	100	100	100	94
BMA1888	iojap domain protein	100	100	100	94
BMA1889	conserved hypothetical protein TIGR00246	100	100	100	97
BMA1890	maf protein	100	100	100	94
BMA1891	ribonuclease G	100	99	100	98
BMA1892	xanthine/uracil permease family protein	100	99	100	96
BMA1893	conserved domain protein	100	99	100	95
BMA1897	conserved hypothetical protein	100	100	100	91
BMA1899	RNA polymerase sigma-70 factor, ECF family	100	99	99	92
BMA1900	carboxymuconolactone decarboxylase family protein	100	100	100	95
BMA1901	hypothetical protein	100	99	100	86
BMA1902	major facilitator superfamily protein	100	98	100	96
BMA1905	lipopolysaccharide core biosynthesis glycosyltransferase	100	100	100	90
BMA1907	lipopolysaccharide core biosynthesis heptosyltransferase	100	99	100	89
BMA1908	glycosyl transferase, group 1 family protein	100	98	100	87
BMA1909	membrane protein, putative	100	100	100	91

BMA1910	heptosyltransferase family protein	100	100	99	90
BMA1911	lipooligosaccharide glycosyl transferase G	100	99	99	89
BMA1912	ABC transporter, permease/ATP-binding protein	100	100	100	98
BMA1913	DNA polymerase III, alpha subunit, form 1	100	99	100	97
BMA1914	rhodanese-like domain protein	100	99	100	92
BMA1915	tRNA synthetases class I domain protein	100	100	100	95
BMA1917	ATP-dependent RNA helicase RhlE	100	100	100	97
BMA1920.1	conserved domain protein	100	100	99	89
BMA1922	phosphoribosylglycinamide formyltransferase 2	100	99	100	95
BMA1923	conserved hypothetical protein	100	100	100	92
BMA1926	conserved hypothetical protein	100	99	100	95
BMA1927	glycosyl hydrolase, family 15	100	100	100	96
BMA1928	intracellular PHB depolymerase	100	99	100	95
BMA1929	transcriptional regulator, TetR family	100	100	100	96
BMA1930	iron-sulfur cluster-binding protein	100	99	99	88
BMA1931	endonuclease III	100	100	100	95
BMA1933	conserved hypothetical protein	100	99	100	96
BMA1934	ISBma1, transposase	100	98	100	98
BMA1935	cytochrome C4 family protein, authentic frameshift	100	99	100	96
BMA1937	conserved hypothetical protein	100	100	100	97
BMA1938	benzoate transport protein	100	99	100	95
BMA1940	transaldolase	100	99	100	95
BMA1941	glyoxalase family protein	100	97	100	92
BMA1942	sodium:solute symporter family protein	100	100	100	97
BMA1943	membrane protein, putative	100	100	100	93
BMA1945	conserved hypothetical protein	100	100	99	91
BMA1946	chorismate lyase family protein	100	100	99	90
BMA1947	heat shock protein HtpG	100	100	100	98
BMA1949	transcriptional regulator, GntR family	100	100	100	97
BMA1950	conserved hypothetical protein	100	98	98	86
BMA1951	membrane protein, putative	100	99	100	95
BMA1952	aminotransferase family protein	100	99	100	95
BMA1956	DNA-binding protein	100	99	100	88
BMA1957	class II aldolase/adducin domain protein	100	99	100	94
BMA1958	dihydrodipicolinate synthase, putative	100	100	100	93
BMA1959	MOSC domain protein	100	99	100	92
BMA1960	hydrolase, alpha/beta fold family	100	99	100	92
BMA1961	transcriptional regulator, LysR family	100	100	99	91
BMA1963	copper resistance protein, putative	100	99	98	93
BMA1964	ribosomal small subunit pseudouridine synthase A	100	100	100	95
BMA1965	conserved hypothetical protein	100	99	100	92
BMA1966	CDP-6-deoxy-delta-3,4-glucoseen reductase, putative	100	100	100	98
BMA1967	acetylornithine aminotransferase	100	99	100	97
BMA1968	acetyltransferase, GNAT family	100	99	97	88
BMA1969	conserved hypothetical protein	100	99	100	95
BMA1970	branched-chain amino acid ABC transporter, ATP-binding protein	100	99	100	97
BMA1971	branched-chain amino acid ABC transporter, ATP-binding protein	100	100	100	99
BMA1972	branched-chain amino acid ABC transporter, permease protein	100	100	100	96
BMA1973	branched-chain amino acid ABC transporter, permease protein	100	99	100	98

BMA1976	polysaccharide synthase family protein	100	100	100	96
BMA1980	glycosyl transferase, putative	100	99	100	89
BMA1981	O-antigen methyl transferase, putative	100	99	100	96
BMA1982	glycosyl transferase, putative	100	99	100	96
BMA1983	NAD-dependent epimerase/dehydratase family protein	100	99	100	97
BMA1984	O-antigen acetylase, putative	100	100	100	95
BMA1985	polysaccharide ABC transporter, ATP-binding protein	100	99	100	97
BMA1986	polysaccharide ABC transporter, permease protein, putative	100	100	100	97
BMA1987	dTDP-4-dehydrorhamnose reductase	100	100	100	95
BMA1988	dTDP-4-dehydrorhamnose 3,5-epimerase	100	100	100	97
BMA1989	glucose-1-phosphate thymidylyltransferase	100	99	100	96
BMA1990	dTDP-glucose 4,6-dehydratase	100	99	100	97
BMA1991	bis(5'-nucleosyl)-tetraphosphatase (symmetrical)	100	99	100	95
BMA1992	acyltransferase family protein	100	100	100	92
BMA1993	aspartate carbamoyltransferase, 44 kDa non-catalytic chain	100	100	100	98
BMA1994	aspartate carbamoyltransferase	100	100	100	99
BMA1995	pyrimidine operon regulatory protein PyrR	100	100	100	96
BMA1996	conserved hypothetical protein TIGR00250	100	100	100	95
BMA1997	conserved hypothetical protein	100	100	100	99
BMA1998	hypothetical protein	100	99	100	91
BMA1999	rubredoxin	100	100	100	100
BMA2000	phosphomethylpyrimidine kinase ThiD, putative	100	100	100	100
BMA2001	chaperonin, 60 kDa	99	99	99	99
BMA2002	chaperonin, 10 kDa	100	100	100	98
BMA2005	transcriptional regulator family protein	100	100	99	90
BMA2006	alcohol dehydrogenase, zinc-containing	100	100	100	94
BMA2009	activator protein, putative	100	100	100	93
BMA2010	outer membrane protein, OmpW family	100	99	100	96
BMA2011	lipoprotein, putative	100	99	100	96
BMA2012	hypothetical protein	100	99	100	93
BMA2018	metallo-beta-lactamase family protein	100	99	100	95
BMA2019	DNA-binding protein	100	99	98	88
BMA2020	acetyltransferase, GNAT family	100	98	100	90
BMA2021	LysE family protein	100	99	100	94
BMA2022	glycerophosphoryl diester phosphodiesterase family protein	100	99	100	95
BMA2023	drug resistance transporter, EmrB/QacA subfamily	100	99	100	95
BMA2024	membrane protein, putative	100	100	100	94
BMA2026	serine-type carboxypeptidase family protein	100	99	100	95
BMA2028	D-(-)-3-hydroxybutyrate-oligomer hydrolase	100	99	100	94
BMA2031	DSBA-like thioredoxin domain protein	100	100	100	97
BMA2032	transcriptional regulator, LysR family	100	99	100	95
BMA2033	iron compound ABC transporter, permease protein	100	97	100	96
BMA2035	iron compound ABC transporter, ATP-binding protein	100	99	100	99
BMA2036	TonB-dependent siderophore receptor family protein	100	98	100	94
BMA2038	conserved hypothetical protein	100	99	100	95
BMA2039	conserved hypothetical protein	100	99	100	96
BMA2041	xanthine dehydrogenase, C-terminal subunit	100	99	100	96
BMA2042	xanthine dehydrogenase, N-terminal subunit	100	99	100	94
BMA2043	transporter, putative	100	100	99	96

BMA2045	membrane protein, putative	100	99	100	94
BMA2046	transcriptional regulator, AraC family	100	99	100	95
BMA2047	alanine--glyoxylate aminotransferase	100	100	100	97
BMA2049	transcriptional regulator, LysR family	100	100	100	98
BMA2050	D-lactate dehydrogenase	100	99	100	98
BMA2051	nitroreductase family protein	100	100	100	96
BMA2052	major facilitator family transporter	100	99	100	95
BMA2053	transporter, putative	100	99	100	94
BMA2055	fumarylacetoacetase	100	99	100	92
BMA2056	homogentisate 1,2-dioxygenase	100	100	100	98
BMA2057	4-hydroxyphenylacetate transporter, putative	100	99	100	96
BMA2058	conserved hypothetical protein	96	98	96	100
BMA2059	FAD-binding monooxygenase, PheA/TfdB family	100	99	100	95
BMA2060	metallo-beta-lactamase family protein	100	100	100	97
BMA2061	EAL domain protein	100	100	96	96
BMA2062	conserved hypothetical protein	100	99	100	91
BMA2063	transcriptional regulator, IclR family	100	100	100	94
BMA2064	glutaryl-CoA dehydrogenase	100	100	100	96
BMA2066	antioxidant, AhpC/Tsa family	100	100	100	98
BMA2067	conserved hypothetical protein	100	99	100	97
BMA2068	conserved hypothetical protein	100	100	100	93
BMA2069	hypothetical protein	100	100	100	92
BMA2071	conserved hypothetical protein	100	100	100	88
BMA2074	conserved hypothetical protein TIGR00244	100	100	100	98
BMA2075	serine hydroxymethyltransferase	100	100	100	98
BMA2076	oxidoreductase, short-chain dehydrogenase/reductase family	100	100	100	96
BMA2077	conserved hypothetical protein TIGR00051	100	99	100	91
BMA2078	tolQ protein	100	100	100	99
BMA2079	tolR protein	100	99	100	97
BMA2080	conserved hypothetical protein	100	100	100	94
BMA2081	tolB protein	100	99	100	97
BMA2082	outer membrane protein, OmpA family	100	100	100	98
BMA2083	conserved hypothetical protein	100	99	100	98
BMA2088	ISBma1, transposase, truncation	100	98	100	99
BMA2089	outer membrane porin, putative	100	100	100	96
BMA2090	ISBma1, transposase	100	98	100	98
BMA2091	transcription elongation factor GreB	100	100	100	95
BMA2094	guanosine-3`,5`-bis(diphosphate) 3`-pyrophosphohydrolase	100	100	100	98
BMA2095	DNA-directed RNA polymerase, omega subunit	100	100	99	100
BMA2096	guanylate kinase	100	100	100	96
BMA2097	conserved hypothetical protein TIGR00255	100	100	100	98
BMA2098	ribonuclease PH	100	100	95	96
BMA2099	HAM1 protein	100	100	100	93
BMA2100	oxygen-independent coproporphyrinogen III oxidase, putative	100	100	100	96
BMA2105	TonB domain protein	100	99	100	92
BMA2106	metabolite:proton symporter family protein	100	99	100	95
BMA2107	hypothetical protein	100	100	100	90
BMA2108	lipopolysaccharide heptosyltransferase I	100	98	100	93
BMA2109	voltage gated chloride channel family protein	100	98	100	87

BMA2109.1	lipoprotein, putative	100	100	100	97
BMA2110	conserved hypothetical protein	100	100	100	88
BMA2111	cell division topological specificity factor MinE	100	100	100	100
BMA2112	septum site-determining protein MinD	100	100	100	98
BMA2113	septum site-determining protein MinC	100	99	100	91
BMA2116	conserved hypothetical protein	100	98	100	92
BMA2118	seryl-tRNA synthetase	100	100	100	99
BMA2119	conserved hypothetical protein	100	100	100	89
BMA2120	ATPase, AAA family protein	100	100	97	98
BMA2121	outer membrane lipoprotein carrier protein LolA	100	100	100	95
BMA2122	cell division protein FtsK	100	99	100	97
BMA2123	thioredoxin-disulfide reductase	100	100	100	97
BMA2125	membrane protein, putative	100	99	100	95
BMA2126	maltose ABC transporter, ATP-binding protein, putative	100	99	100	99
BMA2127	maltose ABC transporter, permease protein, putative	100	100	100	97
BMA2128	maltose ABC transporter, permease protein, putative	100	99	100	97
BMA2129	maltose ABC transporter, periplasmic maltose-binding protein	100	100	100	98
BMA2130	glucose-6-phosphate 1-dehydrogenase	100	100	100	98
BMA2131	6-phosphogluconolactonase	100	99	100	97
BMA2132	glucokinase/transcriptional regulator, RpiR family, fusion	100	99	100	98
BMA2134	amino acid ABC transporter, periplasmic amino acid-binding protein	100	99	100	97
BMA2135	amino acid ABC transporter, permease protein, His/Glu/Gln/Arg/opine family	100	99	100	94
BMA2136	amino acid ABC transporter, ATP-binding protein	100	99	100	94
BMA2139	drug resistance transporter, Bcr/CflA family	100	97	100	92
BMA2141	hypothetical protein	100	100	100	94
BMA2142	glutamate-1-semialdehyde-2,1-aminomutase	100	100	100	99
BMA2143	riboflavin biosynthesis protein RibD	100	98	100	96
BMA2144	riboflavin synthase, alpha subunit	100	100	100	92
BMA2145	bifunctional riboflavin biosynthesis protein RibBA	100	99	100	97
BMA2146	6,7-dimethyl-8-ribityllumazine synthase	100	100	100	100
BMA2147	N utilization substance protein B	100	100	100	100
BMA2148	aspartate aminotransferase	100	100	100	98
BMA2149	transglycosylase SLT domain protein	100	100	100	94
BMA2151	3-octaprenyl-4-hydroxybenzoate carboxy-lyase	100	99	100	97
BMA2152	LysE family protein	100	98	100	93
BMA2154	serine/threonine dehydratase family protein	100	99	100	94
BMA2158	membrane protein, putative	100	100	100	96
BMA2159	conserved hypothetical protein	100	98	97	93
BMA2160	crcB protein	100	99	100	96
BMA2163	serine protease, subtilase family	100	99	100	90
BMA2167	tRNA (guanine-N(7)-)-methyltransferase	100	99	100	88
BMA2168	undecaprenol kinase, putative	100	100	100	94
BMA2169	conserved hypothetical protein	100	100	100	97
BMA2170	conserved hypothetical protein, UPF0153 family	100	100	100	92
BMA2171	oxygen-independent coproporphyrinogen III oxidase	100	99	100	96
BMA2173	protein-L-isoaspartate O-methyltransferase, putative	100	99	100	97
BMA2174	rhodanese-like domain protein	100	99	100	94
BMA2175	omega-amino acid--pyruvate aminotransferase	100	99	100	97
BMA2176	branched-chain amino acid ABC transporter, periplasmic branched-chain amino acid-binding prote	100	99	100	97

BMA2177	branched-chain amino acid ABC transporter, permease protein, putative	100	99	100	94
BMA2178	branched-chain ABC transporter, permease protein, putative	100	99	100	96
BMA2179	branched-chain amino acid ABC tranpsorter, ATP binding protein, putative	100	100	98	93
BMA2180	branched-chain amino acid ABC transporter, ATP-binding protein, putative	100	99	100	96
BMA2181	urease accessory protein UreD	100	100	100	92
BMA2182	urease, gamma subunit	100	100	100	98
BMA2183	urease, beta subunit	100	100	100	94
BMA2184	urease, alpha subunit	100	99	100	97
BMA2186	urease accessory protein UreF	100	100	100	94
BMA2187	urease accessory protein UreG	100	100	100	95
BMA2188	3-deoxy-D-manno-octulosonic-acid transferase	100	99	100	93
BMA2189	conserved hypothetical protein	100	99	100	96
BMA2190	heptosyltransferase I	100	99	100	96
BMA2191	phosphoglucomutase/phosphomannomutase family protein	100	99	100	97
BMA2192	membrane protein, putative	100	100	100	96
BMA2193	glycosyl transferase, group 2 family protein	100	99	100	94
BMA2194	glycosyl transferase, group 1 family protein	100	100	100	97
BMA2195	UDP-glucose 4-epimerase	100	99	100	97
BMA2196	glycosyl transferase family protein	100	100	100	98
BMA2205	rubredoxin	100	100	100	95
BMA2207	ABC transporter, ATP-binding protein	100	99	100	96
BMA2208	DNA topoisomerase IV, subunit B	100	100	100	99
BMA2209	DNA topoisomerase IV, A subunit	100	99	100	98
BMA2211	conserved hypothetical protein	100	99	100	96
BMA2212	transcriptional regulator, LysR family	100	99	100	96
BMA2213	membrane protein, putative	100	99	100	94
BMA2219	acetylpolyamine aminohydrolase	100	99	99	90
BMA2220	chromate transport protein, putative	100	99	100	94
BMA2221	EAL/GGDEF domain protein	100	100	100	96
BMA2222	phenazine biosynthesis protein phzF, putative	100	99	100	92
BMA2223	endoribonuclease, L-PSP family	100	100	100	98
BMA2228	hydroxymethylbutenyl pyrophosphate reductase	100	100	100	99
BMA2229	peptidyl-prolyl cis-trans isomerase, FKBP-type	100	99	100	96
BMA2230	DNA repair protein RadC	100	100	100	86
BMA2231	ribosomal protein L28	100	100	100	100
BMA2232	ribosomal protein L33	100	100	100	100
BMA2233	L-aspartate oxidase	100	99	100	97
BMA2235	nicotinate-nucleotide pyrophosphorylase	100	99	100	94
BMA2236	quinolinate synthetase complex, A subunit	100	100	100	94
BMA2237	fatty acid desaturase family protein	100	99	100	96
BMA2238	mechanosensitive ion channel family protein	100	99	100	95
BMA2239	NOL1/NOP2/sun family protein	100	99	100	97
BMA2240	phosphoribosylglycinamide formyltransferase	100	99	100	96
BMA2241	riboflavin biosynthesis protein RibF	100	99	100	97
BMA2242	isoleucyl-tRNA synthetase	100	99	100	97
BMA2243	lipoprotein signal peptidase	100	100	100	99
BMA2244	phosphopantothenoylcysteine decarboxylase/phosphopantothenate--cysteine ligase	100	99	100	96
BMA2245	deoxyuridine 5`-triphosphate nucleotidohydrolase	100	100	99	96
BMA2246	membrane protein, putative	100	99	100	91

BMA2248	membrane protein, putative	100	97	100	92
BMA2251	conserved hypothetical protein	100	98	100	90
BMA2252	translation elongation factor G	100	99	100	98
BMA2256	oxidoreductase, aldo/keto reductase family	100	99	100	93
BMA2257	transcriptional regulator, GntR family	100	100	100	99
BMA2259	drug resistance transporter, EmrB/QacA family	100	100	100	97
BMA2261	EAL domain protein	100	99	100	97
BMA2262	conserved hypothetical protein	100	99	100	90
BMA2264	2-dehydropantoate 2-reductase	100	99	100	89
BMA2265	membrane protein, putative	100	99	100	92
BMA2266	transcriptional regulator, putative	100	100	100	89
BMA2267	chromate transport protein	100	99	100	93
BMA2271	superoxide dismutase	100	99	100	98
BMA2272	exodeoxyribonuclease VII, large subunit	100	99	100	95
BMA2273	tetraacyldisaccharide 4'-kinase	100	99	100	97
BMA2274	conserved hypothetical protein	100	98	100	94
BMA2275	3-deoxy-D-manno-octulosonate cytidylyltransferase	100	100	100	97
BMA2277	adenylate kinase	100	100	100	97
BMA2279	cold-shock domain family protein	100	100	100	100
BMA2280	ATP-dependent Clp protease adaptor protein ClpS	100	100	100	98
BMA2281	ATP-dependent Clp protease, ATP-binding subunit ClpA	100	100	100	98
BMA2282	amino acid permease, truncation	100	99	100	96
BMA2284.1	hypothetical protein	100	100	100	91
BMA2286	hypothetical protein	100	100	100	86
BMA2287	8-amino-7-oxononanoate synthase, putative	100	100	100	95
BMA2311	yafJ protein, putative	100	100	100	97
BMA2312	mechanosensitive ion channel YggB	100	99	100	95
BMA2313	dedA family protein	100	99	100	95
BMA2314	mismatch repair protein MutL	100	100	100	93
BMA2315	tRNA delta(2)-isopentenylpyrophosphate transferase	100	99	100	93
BMA2316	conserved hypothetical protein	100	97	100	88
BMA2317	phosphoribosylformylglycinamidine cyclo-ligase	100	99	100	98
BMA2318	conserved hypothetical protein	100	99	100	98
BMA2319	HAD-superfamily subfamily IB hydrolase, putative	100	100	100	99
BMA2320	polyA polymerase	100	99	100	95
BMA2321	2-amino-4-hydroxy-6-hydroxymethyldihydropteridine pyrophosphokinase	100	99	100	94
BMA2322	deoxynucleoside kinase family protein	100	99	100	97
BMA2323	3-methyl-2-oxobutanoate hydroxymethyltransferase	100	99	100	97
BMA2324	chorismate binding enzyme	100	100	100	90
BMA2325	chaperone protein DnaJ	100	99	100	97
BMA2326	chaperone protein DnaK	100	100	100	97
BMA2328	co-chaperone GrpE	100	100	100	95
BMA2329	heat shock protein 15	100	100	100	96
BMA2330	ferrochelatase	100	99	100	97
BMA2331	heat-inducible transcription repressor HrcA	100	100	100	98
BMA2332	inorganic polyphosphate/ATP-NAD kinase	100	99	100	97
BMA2333	DNA repair protein RecN	100	99	100	98
BMA2334	glutamate-ammonia-ligase adenylyltransferase	100	99	100	96
BMA2335	conserved hypothetical protein	100	99	100	95

BMA2336	hydrolase, carbon-nitrogen family	100	99	100	97
BMA2337	tldD protein	100	100	100	97
BMA2339	phospho-2-dehydro-3-deoxyheptonate aldolase	100	99	100	99
BMA2341	ribosomal protein L13	100	99	100	99
BMA2342	ribosomal protein S9	100	99	100	96
BMA2343	iron-sulfur cluster assembly accessory protein	100	100	100	100
BMA2344	transcriptional regulator, AraC family	100	99	100	91
BMA2345	glutathione S-transferase, putative	100	100	100	93
BMA2346	glutathione S-transferase, putative	100	99	100	95
BMA2347	conserved hypothetical protein	100	99	100	90
BMA2348	tyrosyl-tRNA synthetase	100	99	100	98
BMA2349	D-tyrosyl-tRNA(Tyr) deacylase	100	100	100	100
BMA2350	phosphoglycerate mutase, putative	100	98	100	92
BMA2352	conserved hypothetical protein	100	99	100	95
BMA2353	Holliday junction DNA helicase RuvB	100	100	100	98
BMA2354	Holliday junction DNA helicase RuvA	100	100	100	97
BMA2355	crossover junction endodeoxyribonuclease RuvC	100	100	100	98
BMA2356	phosphoribosylaminoimidazolecarboxamide formyltransferase/IMP cyclohydrolase	100	99	100	98
BMA2357	factor-for-inversion stimulation protein	100	100	100	100
BMA2358	dihydrouridine synthase	100	100	100	96
BMA2359	2-octaprenyl-6-methoxyphenol hydroxylase	100	99	100	93
BMA2360	Xaa-Pro aminopeptidase	100	98	100	95
BMA2361	glutathione S-transferase	100	100	100	98
BMA2363	glutamate synthase domain protein	100	99	100	97
BMA2364	tRNA (5-methylaminomethyl-2-thiouridylate)-methyltransferase	100	99	100	94
BMA2365	NUDIX domain protein	100	100	100	92
BMA2366	NAD(P) transhydrogenase, alpha-1 subunit	100	100	100	98
BMA2367	NAD(P) transhydrogenase, alpha-2 subunit	100	100	100	97
BMA2368	NAD(P) transhydrogenase, beta subunit	100	99	100	98
BMA2370	conserved hypothetical protein	100	100	100	97
BMA2371	conserved hypothetical protein	100	99	100	94
BMA2374	THUMP domain protein	100	100	100	96
BMA2375	membrane proten, putative	100	99	100	98
BMA2378	paraquat-inducible protein B	100	99	100	97
BMA2379	paraquat-inducible protein A, authentic frameshift	100	97	100	90
BMA2380	cytochrome b561, putative	100	100	100	97
BMA2381	conserved hypothetical protein	100	99	100	96
BMA2382	YceI-like family protein	100	100	100	98
BMA2383	membrane protein, putative	100	97	99	89
BMA2384	protein-export membrane protein SecF	100	98	100	97
BMA2385	protein-export membrane protein SecD	100	99	100	99
BMA2386	preprotein translocase, YajC subunit	100	99	100	97
BMA2387	queuine tRNA-ribosyltransferase	100	100	100	97
BMA2388	S-adenosylmethionine:tRNA ribosyltransferase-isomerase	100	98	100	94
BMA2389	ATP-dependent DNA helicase RecG	100	99	100	97
BMA2390	oxidative stress regulatory protein OxyR	100	99	100	98
BMA2391	catalase/peroxidase HPI	100	99	100	95
BMA2394	ferritin-like domain protein	100	100	100	99
BMA2396	4-hydroxybenzoate polyprenyl transferase	100	100	99	93

BMA2410	pyrroline-5-carboxylate reductase	100	99	100	94
BMA2411	conserved hypothetical protein TIGR00044	96	99	95	93
BMA2412	glycolate oxidase, iron-sulfur subunit	100	99	100	97
BMA2413	glycolate oxidase, subunit GlcE	100	99	100	97
BMA2414	glycolate oxidase, subunit GlcD	100	99	100	98
BMA2415	glycolate oxidase, subunit GlcD, putative	100	99	100	97
BMA2416	ATP:cob(I)alamin adenosyltransferase, putative	100	100	97	94
BMA2417	flavohemoprotein	100	99	100	96
BMA2420	OsmC/Ohr family protein	100	100	100	97
BMA2422	dihydroorotase, homodimeric type	100	100	100	96
BMA2423	conserved hypothetical protein	100	100	100	95
BMA2434	glutamate/aspartate ABC transporter, ATP-binding protein	100	100	100	99
BMA2435	glutamate/aspartate ABC transporter, permease protein	100	100	100	98
BMA2436	glutamate/aspartate ABC transporter, permease protein	100	100	100	99
BMA2437	glutamate/aspartate ABC transporter, periplasmic glutamate/aspartate-binding protein, truncation	100	99	100	99
BMA2438	ISBma1, transposase	100	98	100	98
BMA2439	glutamate dehydrogenase	100	100	100	98
BMA2440	transcriptional regulator, LysR family	100	100	100	96
BMA2441	nitrate transporter, putative	100	99	100	96
BMA2442	adenylosuccinate lyase	100	99	100	96
BMA2443	thermoresistant gluconokinase	100	100	100	97
BMA2444	gluconate permease, putative	100	100	100	97
BMA2445	4-hydroxy-2-oxoglutarate aldolase/2-deydro-3-deoxyphosphogluconate aldolase	100	100	100	96
BMA2446	6-phosphogluconate dehydratase	100	99	100	97
BMA2447	transcriptional regulator, RpiR family	100	98	100	90
BMA2449	membrane protein, putative	100	99	100	97
BMA2450	gamma-glutamyl phosphate reductase	100	99	100	96
BMA2451	DNA polymerase III, delta subunit	100	100	100	97
BMA2452	lipoprotein, putative	100	99	100	92
BMA2453	leucyl-tRNA synthetase	100	99	100	97
BMA2455	MotA/TolQ/ExbB proton channel family protein	100	100	100	95
BMA2456	dihydrodipicolinate reductase	100	99	100	97
BMA2457	outer membrane lipoprotein, putatitve	100	99	100	91
BMA2458	ferric uptake regulator	100	100	100	99
BMA2460	allantoicase	100	99	100	97
BMA2461	C4-dicarboxylate transport protein	100	99	100	95
BMA2462	transcriptional regulator, GntR family	100	100	100	97
BMA2464	transcriptional regulator, LysR family	100	99	100	91
BMA2465	carbonic anhydrases	100	98	98	92
BMA2466	cyanate hydratase	100	99	100	93
BMA2467	transporter, putative	100	99	100	92
BMA2468	glyceraldehyde-3-phosphate dehydrogenase, type I	100	99	100	99
BMA2469	transketolase	100	99	100	96
BMA2471	glyoxalase family protein	100	99	100	95
BMA2472	conserved hypothetical protein TIGR00046	100	98	100	92
BMA2474	barstar family protein	100	100	100	100
BMA2476	ribonuclease SA	100	99	100	93
BMA2477	NADP-dependent malic enzyme	100	99	100	98
BMA2478	thiamine-monophosphate kinase	100	99	100	96

BMA2479	phosphatidylglycerophosphatase A, putative	100	100	100	98
BMA2480	competence/damage-inducible protein CinA	100	100	100	97
BMA2481	orotidine 5'-monophosphate decarboxylase	100	100	100	94
BMA2482	conserved hypothetical protein	100	99	100	94
BMA2483	oxidoreductase, short chain dehydrogenase/reductase family	100	100	100	92
BMA2484	L-arabinose ABC transporter, permease protein	100	100	100	97
BMA2485	L-arabinose ABC transporter, ATP-binding protein	100	99	100	96
BMA2486	L-arabinose ABC transporter, periplasmic L-arabinose-binding protein	100	99	100	97
BMA2487	oxidoreductase, short-chain dehydrogenase/reductase family	100	99	100	92
BMA2488	KDPG/KHG aldolase, putative	100	100	100	92
BMA2489	2-dehydro-3-deoxygalactonokinase	100	100	100	93
BMA2490	transcriptional regulator, IclR family	100	100	100	97
BMA2491	hypothetical protein	100	100	100	91
BMA2492	serine-type carboxypeptidase family protein	100	99	100	94
BMA2493	monofunctional biosynthetic peptidoglycan transglycosylase	100	99	100	92
BMA2494	shikimate 5-dehydrogenase	100	100	100	93
BMA2495	ribonuclease II (RNB) family protein	100	99	100	95
BMA2496	conserved hypothetical protein	100	99	100	97
BMA2497	UDP-N-acetylmuramate:L-alanyl-gamma-D-glutamyl-meso-diaminopimelate ligase	100	99	99	96
BMA2498	conserved hypothetical protein	100	99	100	93
BMA2499	thioredoxin family protein, putative	100	98	100	87
BMA2500	3-dehydroquinate dehydratase, type II	100	100	100	98
BMA2501	acetyl-CoA carboxylase, biotin carboxyl carrier protein	100	99	100	94
BMA2502	acetyl-CoA carboxylase, biotin carboxylase	100	99	100	99
BMA2503	ribosomal protein L11 methyltransferase	100	99	100	97
BMA2505	thiol peroxidase	100	100	100	95
BMA2506	ribokinase, putative	100	99	100	97
BMA2507	outer membrane lipoprotein	100	100	100	100
BMA2509	ribonucleoside-diphosphate reductase, beta subunit	100	99	100	99
BMA2510	ribonucleoside-diphosphate reductase, alpha subunit	100	100	100	98
BMA2511	ampD protein	100	100	100	92
BMA2512	conserved hypothetical protein	100	100	100	94
BMA2513	cytochrome C assembly family protein	100	99	100	96
BMA2514	signal recognition particle protein	100	100	100	99
BMA2515	hypoxanthine-guanine phosphoribosyltransferase, putative	100	100	100	97
BMA2516	membrane protein, MarC family	100	99	100	98
BMA2517	prolyl-tRNA synthetase	100	99	100	98
BMA2518	pyrophosphatase, MutT/nudix family	100	100	100	95
BMA2519	lipoprotein, putative	100	100	100	98
BMA2520	glutamate 5-kinase	100	100	100	96
BMA2521	GTP-binding protein, GTP1/OBG family	100	100	100	97
BMA2522	ribosomal protein L27	100	100	100	100
BMA2523	ribosomal protein L21	100	100	100	100
BMA2524	octaprenyl-diphosphate synthase	100	99	100	97
BMA2530	CBS domain protein	100	99	100	96
BMA2531	type II/IV secretion system protein	100	100	99	92
BMA2532	type IV pilus biogenesis protein PilC	100	99	100	88
BMA2533	type IV pilus prepilin peptidase PilD	100	100	100	96
BMA2534	dephospho-CoA kinase	100	100	100	88

BMA2535	conserved hypothetical protein	100	99	100	98
BMA2536	conserved hypothetical protein	100	100	100	94
BMA2537	pyrophosphatase, NUDIX family	100	99	100	91
BMA2538	conserved hypothetical protein	100	100	100	100
BMA2539	glutamate N-acetyltransferase/amino-acid acetyltransferase	100	99	100	97
BMA2540	preprotein translocase, SecA subunit	100	100	100	98
BMA2542	conserved hypothetical protein	100	100	100	96
BMA2543	UDP-3-0-acyl N-acetylglucosamine deacetylase	100	99	100	98
BMA2545	cell division protein FtsZ	100	100	100	100
BMA2547	cell division protein FtsA	100	99	100	100
BMA2548	cell division protein FtsQ	100	99	100	98
BMA2549	D-alanine--D-alanine ligase B	100	100	100	98
BMA2550	UDP-N-acetylmuramate--alanine ligase	100	99	100	98
BMA2551	UDP-N-acetylglucosamine--N-acetylmuramyl-(pentapeptide) pyrophosphoryl-undecaprenol N-acet	100	100	100	97
BMA2552	cell division protein FtsW	100	100	100	97
BMA2553	UDP-N-acetylmuramoylalanine--D-glutamate ligase	100	99	100	96
BMA2554	phospho-N-acetylmuramoyl-pentapeptide-transferase	100	100	100	100
BMA2555	UDP-N-acetylmuramoyl-tripeptide--D-alanyl-D-alanine ligase	100	100	100	93
BMA2556	UDP-N-acetylmuramoylalanyl-D-glutamate--2,6-diaminopimelate ligase	100	99	100	95
BMA2557	penicillin-binding protein	100	99	100	97
BMA2558	cell division protein FtsL, putative	100	98	100	98
BMA2559	S-adenosyl-methyltransferase MraW	100	99	100	97
BMA2560	mraZ protein	100	99	100	99
BMA2561	conserved hypothetical protein	100	99	100	95
BMA2562	outer membrane porin, putative	100	100	100	98
BMA2571	lipase/acylhydrolase, putative	100	98	100	88
BMA2573	phenylacetic acid degradation protein PaaA	100	99	100	97
BMA2574	phenylacetic acid degradation protein PaaB	100	100	100	98
BMA2575	phenylacetic acid degradation protein PaaC	100	99	100	94
BMA2577	phenylacetic acid degradation NADH oxidoreductase PaaE	100	99	100	97
BMA2578	conserved hypothetical protein	100	99	100	94
BMA2579	transcriptional regulator, TetR family	100	99	100	98
BMA2581	leucine-responsive regulatory protein, putative	100	100	100	98
BMA2582	4-hydroxyphenylpyruvate dioxygenase	100	100	100	98
BMA2582.1	hypothetical protein	100	100	100	87
BMA2584	oxidoreductase, putative	100	99	100	95
BMA2586	NADP-dependent malic enzyme	100	100	100	98
BMA2587	orotate phosphoribosyltransferase	100	100	100	99
BMA2588	phosphatidylethanolamine-binding protein, putative	100	99	100	90
BMA2589	flavodoxin domain protein	100	100	99	97
BMA2590	N-acetyl-gamma-glutamyl-phosphate reductase	100	99	100	97
BMA2591	hypothetical protein	100	99	100	94
BMA2593	outer membrane protein, OmpW family	100	100	100	97
BMA2594	ISBma1, transposase, truncation	96	98	96	98
BMA2597	cytochrome c assembly family protein	100	99	100	95
BMA2598	cytochrome c assembly family protein	100	99	100	96
BMA2599	cytochrome c family protein	100	100	100	96
BMA2600	conserved hypothetical protein	100	100	100	98
BMA2601	porphobilinogen synthase	100	99	100	96

BMA2602	thiol:disulfide interchange protein DsbD, putative	100	99	100	93
BMA2603	periplasmic divalent cation tolerance protein	100	100	100	96
BMA2604	ribosomal protein L17	100	100	100	100
BMA2606	dna-directed rna polymerase alpha chain	100	100	100	99
BMA2607	ribosomal protein S4	100	99	100	97
BMA2608	ribosomal protein S11	100	100	100	100
BMA2609	ribosomal protein S13	100	100	100	99
BMA2610	ribosomal protein L36	100	100	100	100
BMA2611	translation initiation factor IF-1	100	98	100	98
BMA2612	preprotein translocase, SecY subunit	100	99	100	99
BMA2613	ribosomal protein L15	100	100	100	98
BMA2614	ribosomal protein L30	100	100	100	100
BMA2615	ribosomal protein S5	100	100	100	99
BMA2616	ribosomal protein L18	100	100	100	100
BMA2617	ribosomal protein L6	100	100	100	98
BMA2618	ribosomal protein S8	100	100	100	100
BMA2619	ribosomal protein S14	100	99	100	99
BMA2620	ribosomal protein L5	100	99	100	98
BMA2621	ribosomal protein L24	100	100	100	100
BMA2622	ribosomal protein L14	100	100	100	100
BMA2623	ribosomal protein S17	100	100	100	98
BMA2624	ribosomal protein L29	100	100	100	100
BMA2625	ribosomal protein L16	100	100	100	100
BMA2626	ribosomal protein S3	100	100	100	99
BMA2627	ribosomal protein L22	100	100	100	100
BMA2628	ribosomal protein S19	100	100	100	100
BMA2629	ribosomal protein L2	100	100	100	98
BMA2630	ribosomal protein L23	100	100	100	100
BMA2631	ribosomal protein L4	100	100	100	100
BMA2632	ribosomal protein L3	100	100	100	99
BMA2633	ribosomal protein S10	100	100	100	100
BMA2634	translation elongation factor Tu	100	100	100	100
BMA2635	translation elongation factor G	100	99	100	99
BMA2636	ribosomal protein S7	100	100	100	100
BMA2637	ribosomal protein S12	100	99	100	99
BMA2638	ATP-dependent DNA helicase RecQ	100	100	100	98
BMA2639	ISBma1, transposase	100	99	100	98
BMA2640	DNA-directed RNA polymerase, beta subunit	100	99	100	98
BMA2641	DNA-directed RNA polymerase, beta subunit	100	100	100	99
BMA2642	ribosomal protein L7/L12	100	100	100	97
BMA2643	ribosomal protein L10	100	99	100	98
BMA2644	ribosomal protein L1	100	100	100	100
BMA2645	ribosomal protein L11	100	100	100	100
BMA2646	transcription antitermination protein NusG	100	99	100	99
BMA2647	preprotein translocase, SecE subunit	100	99	100	99
BMA2649	translation elongation factor Tu	100	100	100	100
BMA2658	transcriptional regulator, PadR family	100	99	100	95
BMA2659	GMC oxidoreductase	100	98	100	92
BMA2660	oxidoreductase, short-chain dehydrogenase/reductase family	100	98	100	97

BMA2661	conserved hypothetical protein	100	100	100	89
BMA2662	conserved hypothetical protein	100	98	100	93
BMA2663	phenylacetaldehyde dehydrogenase	100	99	100	94
BMA2665	branched-chain amino acid ABC transporter, periplasmic amino acid-binding protein, putative	100	100	100	96
BMA2666	branched-chain amino acid ABC transporter, permease protein, putative	100	100	100	95
BMA2667	branched-chain amino acid ABC transporter, permease protein, putative	100	100	100	96
BMA2668	branched-chain amino acid ABC transporter, ATP-binding protein	100	99	100	97
BMA2669	branched-chain amino acid ABC transporter, ATP-binding protein, putative	100	99	100	93
BMA2670	transcriptional regulator, AraC family	100	100	99	96
BMA2671	outer membrane porin, putative	100	99	100	97
BMA2673	type III restriction-modification system, res subunit	100	99	100	93
BMA2674	type III DNA modification methyltransferase	100	99	100	95
BMA2675	outer membrane porin, putative	100	99	100	98
BMA2676	DNA-binding response regulator	100	100	100	98
BMA2677	sensor histidine kinase	100	99	100	94
BMA2678	ABC transporter, permease protein	100	100	100	97
BMA2679	ABC transporter, ATP-binding protein	100	100	100	98
BMA2680	conserved hypothetical protein	100	99	100	96
BMA2683	transcriptional regulator, MerR family	100	99	98	91
BMA2684	flagellar biosynthetic protein FliR	100	99	100	96
BMA2685	flagellar biosynthetic protein FliQ	100	100	100	97
BMA2686	flagellar biosynthetic protein FliP, interruption-C	100	98	100	97
BMA2691	phage portal protein, PBSX family	100	98	100	98
BMA2695	stringent starvation protein A	100	100	95	100
BMA2696	ubiquinol-cytochrome c reductase, cytochrome c1	100	99	100	96
BMA2697	ubiquinol-cytochrome c reductase, cytochrome b	100	100	100	99
BMA2698	ubiquinol-cytochrome c reductase, iron-sulfur subunit	100	100	100	95
BMA2699	conserved hypothetical protein TIGR00486	100	100	100	97
BMA2700	serine protease	100	99	100	97
BMA2701	Sec-independent protein translocase TatC	100	99	100	97
BMA2702	Sec-independent protein translocase protein TatB	100	98	99	90
BMA2703	twin-arginine translocation protein, TatA/E family	100	100	100	94
BMA2704	HIT family protein	100	100	100	96
BMA2705	membrane protein, putative	100	99	100	97
BMA2706	phosphoribosyl-ATP pyrophosphohydrolase	100	100	100	97
BMA2707	phosphoribosyl-AMP cyclohydrolase	100	100	100	96
BMA2708	imidazoleglycerol phosphate synthase, cyclase subunit	100	99	100	97
BMA2709	phosphoribosylformimino-5-aminoimidazole carboxamide ribotide isomerase	100	100	100	99
BMA2710	imidazole glycerol phosphate synthase, glutamine amidotransferase subunit	100	100	100	98
BMA2711	membrane protein, MarC family	100	100	100	100
BMA2712	imidazoleglycerol-phosphate dehydratase	100	100	100	98
BMA2713	histidinol-phosphate aminotransferase	100	100	100	96
BMA2714	histidinol dehydrogenase	100	99	100	97
BMA2715	ATP phosphoribosyltransferase	100	100	100	98
BMA2716	UDP-N-acetylglucosamine 1-carboxyvinyltransferase	100	99	100	96
BMA2717	BolA/YrbA family protein	100	100	100	100
BMA2718	ABC transporter, permease protein	100	100	100	98
BMA2719	ABC transporter, ATP-binding protein	100	99	100	97
BMA2721	conserved hypothetical protein	100	98	100	92

BMA2722	conserved hypothetical protein	100	100	100	97
BMA2723	lipoprotein VacJ	100	99	100	92
BMA2724	ABC transporter, periplasmic substrate-binding protein	100	100	95	95
BMA2725	ABC transporter, permease protein	100	100	100	100
BMA2726	ABC transporter, ATP-binding protein	100	99	100	98
BMA2727	thiamin-phosphate pyrophosphorylase	100	99	100	93
BMA2728	thiamin biosynthesis ThiG	100	100	100	97
BMA2729	thiamine biosynthesis protein ThiS, putative	100	100	100	95
BMA2730	glycine oxidase ThiO	100	99	100	94
BMA2731	ABC transporter, ATP-binding protein	100	100	100	97
BMA2732	conserved hypothetical protein	100	100	99	88
BMA2733	amino acid carrier protein	100	100	100	97
BMA2735	glutamate synthase, small subunit	100	99	100	96
BMA2736	glutamate synthase, large subunit	100	100	100	99
BMA2737	conserved hypothetical protein	100	100	100	99
BMA2738	outer membrane protein, OmpW family	100	100	100	95
BMA2740	glycerophosphoryl diester phosphodiesterase	100	100	100	92
BMA2741	glycerol-3-phosphate ABC transporter, ATP-binding protein	100	99	100	96
BMA2742	glycerol-3-phosphate ABC transporter, permease protein	100	99	100	98
BMA2743	glycerol-3-phosphate ABC transporter, permease protein	100	99	100	97
BMA2744	glycerol-3-phosphate ABC transporter, periplasmic glycerol-3-phosphate-binding protein	100	99	100	98
BMA2745	deoxyguanosinetriphosphate triphosphohydrolase, putative	100	99	100	96
BMA2746	3-dehydroquinate synthase	100	100	100	96
BMA2747	shikimate kinase	100	99	100	98
BMA2751	hypothetical protein	100	98	100	87
BMA2752	penicillin-binding protein, 1A family	100	100	100	97
BMA2754	conserved hypothetical protein	100	100	100	98
BMA2755	lipoprotein, putative	100	98	100	95
BMA2756	diaminopimelate decarboxylase	100	100	100	94
BMA2757	membrane protein, putative	100	99	98	90
BMA2758	conserved hypothetical protein	100	99	100	94
BMA2761	flagellar biosynthetic protein FliP, interruption-N	100	100	100	98
BMA2762	flagellar protein FliO	100	99	100	91
BMA2763	flagellar motor switch protein FliN	100	100	100	98
BMA2764	flagellar motor switch protein FliM	100	99	100	98
BMA2765	flagellar protein FliL	100	100	100	93
BMA2767	membrane protein, putative	100	100	100	99
BMA2768	LrgA family protein	100	99	99	96
BMA2769	transcriptional regulator, LysR family	100	100	100	96
BMA2770	drug resistance transporter, EmrB/QacA family	100	99	100	95
BMA2771	transcriptional regulator, MarR family	100	99	100	92
BMA2773	RND efflux system, outer membrane lipoprotein, NodT family	100	97	99	91
BMA2774	general secretory pathway protein N	100	99	100	92
BMA2775	general secretion pathway protein M	100	99	100	98
BMA2776	general secretory pathway protein L	100	99	100	88
BMA2777	general secretory pathway protein K	100	99	100	95
BMA2778	general secretory pathway protein J	100	99	100	94
BMA2779	general secretory pathway protein I	100	99	100	93
BMA2780	general secretion pathway protein H	100	98	100	88

BMA2781	general secretion pathway protein G	100	100	100	100
BMA2783	general secretion pathway protein C	100	100	100	96
BMA2784	general secretion pathway protein F	100	100	100	99
BMA2785	general secretion pathway protein E	100	99	100	98
BMA2786	general secretion pathway protein D	100	99	100	91
BMA2787	ipgF protein, putative	100	100	100	91
BMA2789	cobalamin synthesis protein/P47K family protein	100	99	100	92
BMA2791	cold-shock domain family protein	100	100	100	100
BMA2796	amino acid permease	100	99	100	96
BMA2799	transporter, putative	100	99	100	95
BMA2800	phosphoheptose isomerase	100	98	100	94
BMA2801	conserved hypothetical protein TIGR00252	100	98	99	87
BMA2802	tetrapyrrole methylase family protein	100	99	100	93
BMA2804	chaperone protein, putative	100	100	100	96
BMA2805	nitroreductase family protein	100	100	100	97
BMA2806	transporter, putative	100	99	100	98
BMA2807	transcriptional regulator, TetR family	100	97	99	91
BMA2809	conserved hypothetical protein	100	99	96	86
BMA2810	conserved domain protein	100	99	100	91
BMA2811	conserved hypothetical protein	100	99	100	94
BMA2815	conserved hypothetical protein	98	99	97	89
BMA2818	peptidase, M1 family	100	100	100	96
BMA2821	toxin secretion ABC transporter, ATP-binding protein, putative	100	99	100	96
BMA2822	type I secretion outer membrane protein, TolC family	100	99	100	98
BMA2824	conserved hypothetical protein	100	99	100	98
BMA2826	conserved hypothetical protein	100	99	100	97
BMA2827	conserved hypothetical protein	100	100	100	98
BMA2828	OmpA domain protein	100	98	100	90
BMA2829	conserved hypothetical protein	100	99	100	94
BMA2830	ClpA/B type protease	100	98	100	94
BMA2831	conserved hypothetical protein	100	100	100	96
BMA2832	conserved hypothetical protein	100	99	100	96
BMA2833	conserved hypothetical protein	100	100	100	98
BMA2836	oxidoreductase, 2-nitropropane dioxygenase family	100	99	100	92
BMA2838	carboxymethylenebutenolidase	100	100	100	96
BMA2839	6-aminohexanoate-cyclic-dimer hydrolase	100	99	100	93
BMA2840	5,10-methylenetetrahydrofolate reductase	100	100	100	99
BMA2841	membrane protein, putative	100	100	100	99
BMA2842	adenosylhomocysteinase	100	100	100	99
BMA2843	RNA polymerase sigma factor for flagellar operon FliA	100	100	100	98
BMA2844	flagellar biosynthesis protein FlhG, putative	100	99	100	95
BMA2845	flagellar biosynthetic protein FlhF, putative	100	99	100	98
BMA2846	flagellar biosynthesis protein FlhA	100	100	100	99
BMA2847	flagellar biosynthetic protein FlhB	100	100	100	96
BMA2848	conserved hypothetical protein	100	99	99	95
BMA2850	conserved hypothetical protein	100	99	100	93
BMA2851	chemotaxis protein CheZ	100	99	100	98
BMA2852	chemotaxis protein CheY	100	100	100	100
BMA2854	protein-glutamate methylesterase CheB	100	99	100	98

BMA2855	chemotaxis protein CheD	100	99	100	95
BMA2857	methyl-accepting chemotaxis protein, putative	100	99	100	89
BMA2858	chemotaxis protein CheW	100	99	100	98
BMA2860	chemotaxis response regulator	100	100	100	92
BMA2862	chemotaxis MotA protein	100	99	100	98
BMA2863	flagellar transcriptional activator FlhC	100	100	100	98
BMA2864	flagellar transcriptional activator FlhD	100	100	100	98
BMA2866	glycosyl transferase, group 1 family protein	100	98	100	97
BMA2867	H-NS histone family protein	100	100	100	95
BMA2868	aquaporin Z	100	99	100	98
BMA2869	Cof-like hydrolase	100	99	100	92
BMA2870	conserved hypothetical protein	100	100	100	93
BMA2871	DNA-3-methyladenine glycosidase I	100	99	100	91
BMA2872	ribosomal protein S21	100	100	100	100
BMA2873	flagellin	100	100	100	90
BMA2874	flagellar hook-associated protein 2	100	99	100	94
BMA2875	hypothetical protein	100	99	99	93
BMA2886	LysE family protein	100	99	100	95
BMA2888	acid phosphatase AcpA	100	99	100	96
BMA2889	lipoprotein, putative	100	100	100	95
BMA2890	lipoprotein, putative	100	100	100	93
BMA2891	curli production assembly/transport component CsgG, putative	100	99	100	98
BMA2893	hydrolase, alpha/beta fold family	100	100	99	92
BMA2894	methyl-accepting chemotaxis protein, putative	100	99	99	97
BMA2895	oxidoreductase, FAD-binding, putative	100	98	100	88
BMA2896	chitin binding protein, putative	100	100	100	95
BMA2901	amino acid ABC transporter, periplasmic amino acid-binding protein, putative	100	99	100	98
BMA2902	amino acid ABC transporter, permease protein, putative	100	99	100	93
BMA2903	conserved hypothetical protein	100	99	100	91
BMA2905	H-NS histone family protein	100	100	100	100
BMA2906	cation efflux family protein	100	99	99	96
BMA2907	transcriptional regulator, AsnC family	100	99	100	99
BMA2908	conserved hypothetical protein	100	100	100	95
BMA2909	metallo-beta-lactamase family protein	100	99	100	95
BMA2911	rare lipoprotein A family protein	100	100	100	96
BMA2913	DNA-binding protein HU, form B	100	100	100	100
BMA2914	oxidoreductase, FAD-binding family protein	100	99	100	91
BMA2916	sodium/hydrogen exchanger family protein	100	100	100	96
BMA2917	conserved hypothetical protein	100	100	100	99
BMA2918	transcriptional regulator, MarR family	100	98	100	96
BMA2919	glutamine amidotransferase class-I domain protein, putative	100	100	100	94
BMA2921	SET domain protein	100	100	100	97
BMA2923	sensor histidine kinase	100	99	100	95
BMA2924	DNA-binding response regulator	100	100	100	100
BMA2926	pterin-4-alpha-carbinolamine dehydratase, putative	100	100	100	98
BMA2927	phenylalanine-4-hydroxylase	100	99	95	89
BMA2928	transcriptional regulator, AsnC family	100	100	99	96
BMA2929	adenylate cyclase, putative	100	100	100	97
BMA2930	transcriptional regulator, LysR family	100	99	100	97

BMA2931	methylmalonate-semialdehyde dehydrogenase	100	100	100	97
BMA2933	oxidoreductase, GMC family protein	100	99	100	95
BMA2934	branched-chain amino acid ABC transporter, ATP-binding protein	100	100	100	98
BMA2935	branched-chain amino acid ABC transporter, ATP-binding protein	100	99	100	96
BMA2936	branched-chain amino acid ABC transporter, periplasmic branched-chain amino acid-binding prote	100	99	100	97
BMA2937	branched-chain amino acid ABC transporter, permease protein	100	99	100	97
BMA2938	branched-chain amino acid ABC transporter, permease protein	100	100	100	97
BMA2940	branched-chain amino acid ABC transporter, periplasmic amino acid-binding protein	100	100	100	96
BMA2941	branched-chain amino acid ABC transporter, permease protein	100	100	100	97
BMA2942	branched-chain amino acid ABC tranpsorter, permease/ATP-binding protein	100	99	100	94
BMA2943	branched-chain amino acid ABC transporter, ATP-binding protein	100	100	100	93
BMA2944	glucose-inhibited division protein A	100	99	100	96
BMA2945	methyltransferase GidB	100	99	100	95
BMA2946	sporulation initiation inhibitor protein Soj	100	100	100	99
BMA2947	stage 0 sporulation protein J, putative	100	100	100	98
BMA2948	transporter, putative	100	99	99	93
BMA2950	ATP synthase protein I, putative	100	99	100	97
BMA2951	ATP synthase F0, A subunit	100	99	100	97
BMA2952	ATP synthase F0, C subunit	100	100	100	100
BMA2953	ATP synthase F0, B subunit	100	99	100	99
BMA2954	ATP synthase F1, delta subunit	100	99	100	98
BMA2955	ATP synthase F1, alpha subunit	100	99	100	99
BMA2956	ATP synthase F1, gamma subunit	100	100	100	98
BMA2957	ATP synthase F1, beta subunit	100	100	100	100
BMA2958	ATP synthase F1, epsilon subunit	100	100	100	97
BMA2959	AMP-binding enzyme domain protein	100	99	100	94
BMA2961	cyclohexadienyl dehydratase	100	99	100	92
BMA2962	uroporphyrinogen decarboxylase	100	99	99	98
BMA2963	primosomal protein n`	100	99	100	95
BMA2965	proline dehydrogenase/delta-1-pyrroline-5-carboxylate dehydrogenase	100	99	100	98
BMA2966	amino acid ABC transporter, periplasmic amino acid-binding protein, putative	100	99	100	97
BMA2967	feruloyl-CoA synthetase	100	100	100	93
BMA2968	acyl-CoA dehydrogenase domain protein	100	100	100	95
BMA2971	aromatic aminotransferase, putative	100	100	100	93
BMA2972	lysine-arginine-ornithine-binding periplasmic protein	100	99	100	90
BMA2974	transcriptional regulator, PadR family	100	100	98	92
BMA2975	transcriptional regulator, merR family	100	100	100	97
BMA2976	cadmium-translocating P-type ATPase	100	99	100	93
BMA2978	membrane protein, putative	100	99	100	97
BMA2981	ethanolamine permease, putative	100	99	100	98
BMA2983	ethanolamine ammonia-lyase heavy chain	100	99	100	96
BMA2984	ethanolamine ammonia-lyase light chain	100	99	98	95
BMA2986	aldehyde dehydrogenase family protein	100	100	100	98
BMA2987	transcriptional regulator, araC family	100	100	100	97
BMA2988	hypothetical protein	100	99	100	89
BMA2989	acetolactate synthase, large subunit, putative	100	99	100	96
BMA2991	L-serine ammonia-lyase	100	99	100	96
BMA2992	hypothetical protein	100	100	100	97
BMA2993	glycine dehydrogenase	100	100	100	96

BMA2994	glycine cleavage system H protein	100	98	100	99
BMA2994.1	glycine cleavage system T protein	100	100	100	95
BMA2997	lipoprotein, putative	100	100	100	98
BMA2998	oxidoreductase, Gfo/Idh/MocA family	100	99	100	92
BMA3002	ATP-dependent DNA helicase Rep	100	99	100	98
BMA3003	cytochrome c family protein	100	99	100	96
BMA3010	alpha,alpha-trehalose-phosphate synthase	100	100	100	98
BMA3011	conserved hypothetical protein	100	99	99	88
BMA3013	drug resistance transporter, EmrB/QacA subfamily, putative	100	99	100	92
BMA3014	conserved hypothetical protein TIGR00481	100	100	98	94
BMA3016	hypothetical protein	100	99	100	90
BMA3020	ecotin	100	98	100	93
BMA3021	D-alanyl-D-alanine carboxypeptidase family protein	100	99	100	87
BMA3021.1	hypothetical protein	100	100	100	90
BMA3029	conserved hypothetical protein	100	99	100	89
BMA3030	conserved hypothetical protein	100	99	100	86
BMA3031	acyl transferase domain protein	100	98	100	89
BMA3034	malonate decarboxylase, gamma subunit	100	99	100	93
BMA3035	malonate decarboxylase, beta subunit	100	100	100	93
BMA3036	malonate decarboxylase, delta subunit	100	99	100	95
BMA3037	malonate decarboxylase alpha-subunit	100	99	100	96
BMA3038	malonate transporter, M subunit	100	99	100	96
BMA3039	malonate transporter, L subunit	100	98	100	91
BMA3040	transcriptional regulator, LysR family	100	100	100	96
BMA3041	3-hydroxyacyl-CoA dehydrogenase family protein	100	100	100	93
BMA3042	phosphoenolpyruvate carboxykinase	100	100	100	96
BMA3047	heat shock protein, Hsp20 family	100	100	100	93
BMA3048	heat shock protein, Hsp20 family	100	98	99	90
BMA3055	oxidoreductase, short-chain dehydrogenase/reductase family	100	99	100	91
BMA3056	transcriptional regulator, LysR family, authentic point mutation	100	99	100	94
BMA3062	transcriptional regulator, putative	100	99	100	88
BMA3063	membrane protein, putative	100	99	100	89
BMA3064	conserved hypothetical protein	100	100	100	93
BMA3066	GGDEF domain protein	100	99	100	95
BMA3070	transcriptional regulator, LacI family	100	99	100	95
BMA3071	ABC transporter, periplasmic substrate-binding protein	100	99	100	93
BMA3072	ABC transporter, permease protein	100	100	100	97
BMA3072.1	hypothetical protein	100	100	100	97
BMA3075	ABC transporter, permease protein	100	98	100	96
BMA3076	ABC transporter, ATP-binding protein	100	100	100	95
BMA3082	major facilitator family transporter	100	100	100	92
BMA3083	membrane protein, putative	100	100	100	96
BMA3085	oxidoreductase, molybdopterin-binding	100	99	100	91
BMA3090	thiamine biosynthesis protein ThiC	100	99	100	97
BMA3094	major facilitator family transporter	100	100	100	98
BMA3095	excinuclease ABC, A subunit	100	99	100	98
BMA3096	membrane protein, putative	100	99	100	97
BMA3097	formyltetrahydrofolate deformylase	100	99	100	95
BMA3098	thiamin pyrophosphokinase-related protein	100	99	99	95

BMA3099	LysE family protein	100	95	100	94
BMA3101	potassium efflux system protein	100	99	100	97
BMA3102	carbohydrate isomerase, KpsF/GutQ family	100	100	100	97
BMA3103	phosphatase, YrbI family	100	100	100	94
BMA3104	conserved hypothetical protein	100	99	100	98
BMA3105	conserved hypothetical protein	100	99	100	96
BMA3107	ABC transporter, ATP-binding protein	100	99	100	97
BMA3108	RNA polymerase sigma-54 factor	100	100	100	98
BMA3109	ribosomal subunit interface protein	100	100	100	97
BMA3110	PTS system, nitrogen regulatory IIA protein	100	100	100	99
BMA3111	HPr(Ser) kinase/phosphatase, putative	100	100	100	100
BMA3112	conserved hypothetical protein	100	99	100	96
BMA3113	ATP-dependent protease La domain protein	100	100	100	96
BMA3114	A/G-specific adenine glycosylase	100	99	100	93
BMA3115	formamidopyrimidine-DNA glycosylase	100	98	100	96
BMA3116	TPR domain protein	100	100	100	96
BMA3118	4-diphosphocytidyl-2C-methyl-D-erythritol kinase	100	99	99	92
BMA3120	ribose-phosphate pyrophosphokinase	100	100	100	99
BMA3121	ribosomal protein L25	100	100	100	96
BMA3122	peptidyl-tRNA hydrolase	100	99	100	94
BMA3123	histidinol-phosphate aminotransferase	100	99	100	94
BMA3124	ferredoxin	100	100	100	97
BMA3125	pantetheine-phosphate adenylyltransferase	100	100	100	100
BMA3126	methyltransferase, putative	100	99	100	95
BMA3127	signal recognition particle-docking protein FtsY	100	99	100	96
BMA3128	maleylacetoacetate isomerase	100	99	100	95
BMA3130	nitrite reductase [NAD(P)H], large subunit	100	99	100	96
BMA3131	nitrite reductase [NAD(P)H], small subunit	100	97	100	94
BMA3132	molybdopterin oxidoreductase family protein	100	99	100	95
BMA3133	glycosyl transferase family protein	100	99	100	90
BMA3135	2-isopropylmalate synthase	100	100	100	96
BMA3135.1	hypothetical protein	100	100	100	97
BMA3136	conserved hypothetical protein	100	99	100	95
BMA3138	RNA polymerase sigma-32 factor	100	99	100	97
BMA3140	ABC transporter, periplasmic substrate-binding protein, putative	100	99	100	97
BMA3141	ABC transporter, permease protein	100	100	100	97
BMA3142	ABC transporter, ATP-binding protein	100	100	100	98
BMA3143	conserved hypothetical protein	100	99	100	95
BMA3144	conserved hypothetical protein	100	99	100	93
BMA3145	DNA polymerase III, alpha subunit, form 2	100	99	100	95
BMA3168	transcriptional regulator, GntR family	100	99	100	97
BMA3168.1	N-acetylglucosamine-6-phosphate deacetylase	100	100	100	97
BMA3170	SIS domain protein	100	100	100	95
BMA3171	PTS system, glucose-specific EIIA/HPr/phosphoenolpyruvate-protein phosphotransferase compon	100	99	100	92
BMA3172	PTS system, N-acetylglucosamine-specific IIABC component	100	99	100	96
BMA3173	beta-N-acetylhexosaminidase, putative	100	99	100	95
BMA3176	cyd operon protein YbgT	100	100	100	96
BMA3177	cytochrome d ubiquinol oxidase, subunit II	100	99	100	96
BMA3178	cytochrome d ubiquinol oxidase, subunit I	100	100	100	97

BMA3179	hypothetical protein	100	100	100	93
BMA3181	ISBma1, transposase	100	98	100	98
BMA3183	D-methionine ABC transporter, periplasmic D-methionine-binding protein	100	100	100	97
BMA3184	methyl-accepting chemotaxis protein	100	99	100	97
BMA3186	conserved hypothetical protein	100	100	99	98
BMA3187	SCO1/SenC family protein	100	100	100	97
BMA3188	protoheme IX farnesyltransferase	100	99	100	99
BMA3189	cytochrome c assembly family protein	100	99	100	98
BMA3190	conserved hypothetical protein	100	99	100	92
BMA3191	conserved hypothetical protein	100	100	100	96
BMA3192	conserved hypothetical protein	100	100	100	100
BMA3193	cytochrome c oxidase, subunit III	100	100	100	98
BMA3194	conserved hypothetical protein	100	98	100	95
BMA3195	cytochrome c oxidase assembly protein ctaG, putative	100	100	100	94
BMA3196	cytochrome c oxidase, subunit I	100	100	100	99
BMA3197	cytochrome c oxidase, subunit II	100	100	100	96
BMA3198	conserved hypothetical protein	100	100	100	98
BMA3199	conserved hypothetical protein	100	99	100	95
BMA3200	ComF family protein	100	98	100	92
BMA3201	RNA methyltransferase, TrmH family, group 2	100	99	100	96
BMA3203	conserved hypothetical protein	100	100	99	91
BMA3204	glycerol-3-phosphate dehydrogenase (NAD(P)+)	100	100	100	96
BMA3205	protein-export protein SecB	100	100	100	96
BMA3206	glutaredoxin 3	100	98	100	97
BMA3207	rhodanese-like domain protein	100	98	100	97
BMA3208	phosphoglycerate mutase	100	100	100	95
BMA3209	carboxy-terminal protease	100	99	100	97
BMA3210	HesA/MoeB/ThiF family protein	100	100	100	95
BMA3211	phosphoenolpyruvate-protein phosphotransferase	100	99	100	97
BMA3212	phosphocarrier protein HPr	100	100	100	96
BMA3213	PTS system, fructose-specific IIA component	100	100	100	98
BMA3214	glutathione synthetase	100	100	100	97
BMA3215	conserved hypothetical protein	100	99	100	98
BMA3217	ammonium transporter	100	97	99	95
BMA3218	regulatory protein, P-II family	100	100	100	100
BMA3219	conserved hypothetical protein	100	100	100	97
BMA3220	Mg chelatase, subunit D/I family protein	100	99	100	95
BMA3223	thioesterase domain protein	100	99	100	91
BMA3225	conserved hypothetical protein	100	100	100	97
BMA3226	C4-dicarboxylate transport transcriptional regulatory protein	100	100	100	97
BMA3228	C4-dicarboxylate transport protein	100	99	100	97
BMA3229	sodium:solute symporter family protein	100	99	100	96
BMA3230	conserved hypothetical protein	100	100	100	95
BMA3231	CAIB/BAIF family protein	100	99	100	96
BMA3232	acyl-CoA dehydrogenase domain protein	100	99	100	91
BMA3233	acyl-CoA dehydrogenase domain protein	100	99	100	95
BMA3234	fatty oxidation complex, alpha subunit, putative	100	99	100	95
BMA3235	transcriptional regulator, LysR family	100	99	98	98
BMA3237	gamma-glutamyltransferase	100	99	100	91

BMA3238	conserved domain protein	100	98	100	88
BMA3240	alcohol dehydrogenase, zinc-containing	100	99	100	94
BMA3241	ISBma1, transposase	100	99	100	99
BMA3242	transcriptional regulator, AraC family	100	99	100	95
BMA3243	conserved hypothetical protein	100	98	100	93
BMA3244	AmpG-related permease	100	99	100	98
BMA3245	methionine biosynthesis protein MetW	100	99	100	98
BMA3246	homoserine O-acetyltransferase	100	100	100	99
BMA3247	transcriptional regulator, TetR family	100	99	100	91
BMA3248	HAD-superfamily hydrolase	100	99	100	95
BMA3249	acetylglutamate kinase	100	100	100	99
BMA3251	sensor histidine kinase	100	100	100	99
BMA3252	response regulator	100	100	100	99
BMA3253	heat shock protein HslVU, ATPase subunit HslU	100	99	100	98
BMA3254	protease HslVU, subunit HslV	100	100	100	97
BMA3255	DnaK suppressor protein	100	100	100	100
BMA3256	cobalamin synthesis protein/P47K family protein	100	100	100	97
BMA3257	conserved hypothetical protein	100	99	100	94
BMA3258	integrase/recombinase XerC	100	99	98	94
BMA3259	conserved hypothetical protein	100	99	98	95
BMA3260	diaminopimelate epimerase	100	99	100	95
BMA3261	lipid A biosynthesis lauroyl acyltransferase, putative	100	99	100	96
BMA3262	S-adenosylmethionine synthetase	100	99	100	99
BMA3263	hypothetical protein	100	100	100	96
BMA3264	conserved hypothetical protein	100	99	100	90
BMA3266	oxidoreductase, aldo/keto reductase family	100	100	100	96
BMA3267	hypothetical protein	100	100	100	100
BMA3268	membrane protein, putative	100	99	100	90
BMA3269	major facilitator family transporter	100	99	96	92
BMA3269.1	hypothetical protein	100	100	100	100
BMA3271	conserved hypothetical protein	100	99	100	95
BMA3273	coniferyl aldehyde dehydrogenase	100	100	100	96
BMA3275	oxidoreductase, GMC family	100	99	100	95
BMA3277	flagellar FliJ protein	100	100	100	96
BMA3278	flagellum-specific ATP synthase FliI	100	100	100	93
BMA3279	flagellar assembly protein FliH	100	99	100	96
BMA3280	flagellar motor switch protein FliG	100	100	100	97
BMA3281	flagellar M-ring protein FliF	100	99	100	97
BMA3282	flagellar hook-basal body complex protein FliE, putative	100	99	100	96
BMA3283	flagellar protein FliS	100	100	100	93
BMA3284	conserved hypothetical protein	100	100	100	95
BMA3286	flagellar biosynthetic protein FlhB domain protein	100	98	100	93
BMA3287	conserved hypothetical protein	100	100	100	93
BMA3288	lipoprotein, putative	100	98	100	94
BMA3289	amino acid permease	100	99	100	98
BMA3292	ferredoxin--NADP reductase	100	100	100	98
BMA3294	conserved hypothetical protein	100	100	99	95
BMA3296	ATP-dependent protease domain protein	100	100	100	97
BMA3297	oxidoreductase, FAD-binding family protein	100	99	100	94

BMA3298	high potential iron-sulfur protein, putative	100	100	100	96
BMA3299	major facilitator family transporter	100	99	100	95
BMA3301	dipeptide ABC transporter, periplasmic didpeptide-binding protein	100	99	100	97
BMA3302	dipeptide ABC transporter, permease protein	100	99	100	97
BMA3304	peptide ABC transporter, ATP-binding protein	100	99	100	96
BMA3305	dipeptide ABC transporter, ATP-binding protein	100	99	100	96
BMA3306	GumN family protein	100	99	100	97
BMA3307	membrane protein, putative	100	100	100	95
BMA3308	membrane protein, putative	100	100	100	93
BMA3309	LamB/YcsF family protein	100	99	100	96
BMA3310	allophanate hydrolase, subunit 2	96	100	98	91
BMA3311	conserved hypothetical protein TIGR00370	100	100	100	97
BMA3312	conserved hypothetical protein	100	100	100	98
BMA3313	5-formyltetrahydrofolate cyclo-ligase family protein	100	98	100	93
BMA3314	lytic murein transglycosylase, putative	100	99	100	96
BMA3315	NADH-ubiquinone oxidoreductase, putative	100	100	100	91
BMA3316	glutathione S-transferase, N-terminal domain protein	100	100	100	95
BMA3317	tRNA nucleotidyltransferase	100	99	100	95
BMA3321	flagella synthesis protein FlgN, putative	99	100	100	94
BMA3322	negative regulator of flagellin synthesis FlgM, putative	100	99	99	93
BMA3323	flagella basal body P-ring formation protein FlgA	100	98	100	89
BMA3325	flagellar basal-body rod protein FlgB	100	99	100	95
BMA3326	flagellar basal-body rod protein FlgC	100	100	100	97
BMA3327	basal-body rod modification protein FlgD	100	97	100	91
BMA3328	flagellar hook protein FlgE	100	100	100	86
BMA3329	flagellar basal-body rod protein FlgF	100	100	100	99
BMA3330	flagellar basal-body rod protein FlgG	100	99	100	98
BMA3331	flagellar L-ring protein FlgH	100	100	100	98
BMA3332	flagellar P-ring protein	100	99	100	97
BMA3333	flagellar protein FlgJ	100	100	100	94
BMA3334	conserved hypothetical protein	100	100	100	95
BMA3335	flagellar hook-associated protein 1	100	100	100	93
BMA3336	flagellar hook-associated protein 3	100	100	100	95
BMA3337	xanthine/uracil permease family protein	100	100	100	96
BMA3340	chromate transporter, putative, authentic frameshift	100	96	100	89
BMA3341	hypothetical protein	100	100	100	93
BMA3342	alkylphosphonate utilization operon protein PhnA, putative	100	100	100	99
BMA3343	membrane protein, putative	100	98	100	93
BMA3345	outer membrane porin, putative	100	99	100	96
BMA3346	hypothetical protein	100	99	100	92
BMA3347	lipoprotein, putative	100	99	100	88
BMA3348.1	hypothetical protein	100	99	100	90
BMA3350	glycosyl transferase, group 2 family protein	98	99	98	93
BMA3354	carbohydrate porin, OprB family	100	100	100	89
BMA3356.1	hypothetical protein	100	100	98	88
BMA3358	manganese/iron transporter, NRAMP family	100	98	100	88
BMA3359.1	hypothetical protein	100	98	100	94
BMA3362	glutathione-disulfide reductase	100	100	100	94
BMA3363	argininosuccinate synthase	100	100	100	97

BMA3365	conserved hypothetical protein	100	100	100	86
BMA3368	copper-translocating P-type ATPase	100	99	100	90
BMA3370	LemA family protein	100	99	100	90
BMA3371	lipoprotein, putative	100	99	100	89
BMA3372	conserved hypothetical protein	100	98	100	86
BMA3379	glucosamine--fructose-6-phosphate aminotransferase, isomerizing	100	100	100	96
BMA3380	UDP-N-acetylglucosamine pyrophosphorylase	100	99	100	96
BMA3381	conserved hypothetical protein	100	100	100	98
BMA3382	dihydroneopterin aldolase	100	100	100	99
BMA3384	conserved hypothetical protein	100	99	100	92
BMA3385	membrane protein, putative	100	100	100	95
BMA3386	fructokinase, putative	100	100	100	95
BMA3387	conserved hypothetical protein	100	97	100	88
BMA3388	transcriptional regulator, LacI family	100	100	100	98
BMA3389	methyl-accepting chemotaxis protein, putative	100	100	100	95
BMA3390	sodium/bile acid symporter family protein	100	100	100	94
BMA3395	tRNA modification GTPase TrmE	100	100	100	95
BMA3396	hypothetical protein	100	99	100	98
BMA3397	inner membrane protein, 60 kDa	100	99	100	97
BMA3398	conserved hypothetical protein	100	99	100	96
BMA3399	ribonuclease P protein component	100	98	100	97
BMA3400	ribosomal protein L34	100	100	100	100
BMAA0001	hypothetical protein	100	100	100	91
BMAA0002	phage integrase family protein	100	99	100	95
BMAA0003	hypothetical protein	96	99	100	88
BMAA0004	DNA-binding protein	100	100	100	96
BMAA0005	2-amino-3-ketobutyrate coenzyme A ligase	100	99	100	96
BMAA0006	threonine 3-dehydrogenase	100	100	100	99
BMAA0007	YceI like familyprotein	100	99	100	91
BMAA0008	transcriptional regulator, TetR family	100	100	100	90
BMAA0009	conserved hypothetical protein	100	100	100	96
BMAA0010	succinylglutamate desuccinylase / aspartoacylase family protein	100	99	100	88
BMAA0011	tartrate dehydrogenase	100	99	100	95
BMAA0012	transcriptional regulator, LysR family	100	99	100	95
BMAA0013	membrane protein, putative	100	99	100	87
BMAA0014	glutathione S-transferase	100	100	100	90
BMAA0015	hypothetical protein	100	100	100	94
BMAA0016	patatin-like phospholipase	100	99	100	98
BMAA0017	D-beta-hydroxybutyrate dehydrogenase	100	100	100	97
BMAA0018	acetoacetate decarboxylase	100	100	100	99
BMAA0019	PAP2 family protein	100	99	100	92
BMAA0021	siderophore-interacting protein	100	99	100	92
BMAA0022	transcriptional regulator, PadR family	100	99	100	92
BMAA0024	hypothetical protein	99	100	99	98
BMAA0025	conserved hypothetical protein	100	100	100	91
BMAA0027	radical SAM domain/B12 binding domain protein	100	99	100	88
BMAA0029	LysE family protein	100	99	100	97
BMAA0030	transcriptional regulator, LysR family	100	99	100	95
BMAA0031	transcriptional regulator, Crp/Fnr family	100	99	100	98

BMAA0032	universal stress protein family	100	100	100	98
BMAA0035	aldehyde dehydrogenase (NADP) family protein	100	99	100	92
BMAA0039	conserved hypothetical protein	100	100	100	86
BMAA0040	hypothetical protein	100	100	100	97
BMAA0041	serine O-acetyltransferase, putative	100	99	100	93
BMAA0042	oxidoreductase, short chain dehydrogenase/reductase family	100	100	100	97
BMAA0043	GTP cyclohydrolase I	100	99	100	90
BMAA0044	transcriptional regulator, LysR family	100	100	100	95
BMAA0045	4-hydroxybenzoate hydroxylase	100	99	100	92
BMAA0046	3-oxoadipate CoA-transferase, alpha subunit	100	100	100	97
BMAA0047	3-oxoadipate CoA-succinyl transferase beta subunit	100	100	100	94
BMAA0048	3-carboxy-cis,cis-muconate cycloisomerase	100	99	100	93
BMAA0049	3-oxoadipate enol-lactone hydrolase/4-carboxymuconolactone decarboxylase, authentic point muta	100	99	100	92
BMAA0050	4-hydroxybenzoate transporter	100	99	100	94
BMAA0051	hypothetical protein	100	100	100	97
BMAA0052	LysE family protein	100	100	100	94
BMAA0055	chromate transport protein, authentic point mutation	100	98	100	93
BMAA0056	chromate resistance protein ChrB	100	99	100	92
BMAA0062	excinuclease ABC, subunit A, form 2	100	99	100	94
BMAA0076	conserved domain protein	100	100	99	88
BMAA0079	phospholipase C	100	99	100	95
BMAA0085	exopolysaccharide tyrosine-protein kinase, putative	100	99	100	93
BMAA0086	conserved domain protein	100	100	100	88
BMAA0087	membrane protein, putative	100	100	100	92
BMAA0089	membrane protein, putative	100	100	100	95
BMAA0093	serine protease, subtilase family	100	99	100	95
BMAA0096	hypothetical protein	100	100	100	94
BMAA0097	GGDEF domain protein	100	99	100	88
BMAA0099	hydrolase, alpha/beta fold family	100	100	100	96
BMAA0100	conserved hypothetical protein	100	99	100	94
BMAA0101	oxidoreductase, short chain dehydrogenase/reductase family	100	99	100	95
BMAA0102	transcriptional regulator, TetR family	100	99	100	89
BMAA0104	transcriptional regulator, LysR family	100	100	100	95
BMAA0107	glutathione S-transferase	100	99	100	92
BMAA0109	nucleoside transporter	100	99	100	97
BMAA0110	deoxyribose-phosphate aldolase	100	99	100	94
BMAA0114	thymidine phosphorylase	100	100	100	91
BMAA0115	cytidine deaminase	100	100	100	95
BMAA0116	osmotically inducible protein Y domain protein	100	99	100	93
BMAA0117	6-phosphofructokinase	100	99	100	89
BMAA0120	acetate kinase	100	99	100	92
BMAA0121	phosphate acetyl/butyryltransferase family protein	100	99	100	94
BMAA0122	poly-beta-hydroxybutyrate polymerase	100	99	100	90
BMAA0123	ATP synthase F1, beta subunit	100	98	100	90
BMAA0124	ATP synthase F1, epsilon subunit	100	100	100	95
BMAA0126	hypothetical protein	100	100	100	91
BMAA0127	ATP synthase F0, A subunit	100	100	100	95
BMAA0128	ATP synthase F0, C subunit	100	100	100	100
BMAA0129	ATP synthase F0, B subunit	100	100	100	92

BMAA0131	ATP synthase F1, gamma subunit, putative	100	99	100	92
BMAA0132	alcohol dehydrogenase, zinc-containing	100	99	100	95
BMAA0134	conserved hypothetical protein	100	98	100	92
BMAA0138	ABC transporter, permease	100	98	100	96
BMAA0141	RND efflux system, outer membrane protein	100	100	100	92
BMAA0144	universal stress protein family	100	100	100	98
BMAA0145	hypothetical protein	100	97	100	86
BMAA0146	ABC transporter, permease protein	100	99	100	95
BMAA0147	ABC transporter, ATP-binding protein	100	99	100	95
BMAA0148	membrane protein	100	99	100	87
BMAA0150	polysaccharide deacetylase domain protein	100	99	100	86
BMAA0152	methyl-accepting chemotaxis protein	100	99	99	91
BMAA0154	sulfotransferase domain protein	100	97	100	89
BMAA0155	osmotically inducible protein Y domain protein	100	100	100	95
BMAA0157	hypothetical protein	100	100	99	90
BMAA0158	decarboxylase family protein	100	97	100	90
BMAA0160	hypothetical protein	100	99	100	88
BMAA0163	alcohol dehydrogenase, zinc-containing	100	100	100	96
BMAA0164	transcriptional regulator, Crp/Fnr family	100	100	100	97
BMAA0166	acetoacetyl-CoA reductase	100	99	100	96
BMAA0168	metallo-beta-lactamase family protein	100	99	100	93
BMAA0169	conserved hypothetical protein	100	99	100	96
BMAA0172	lysine-specific permease	100	100	100	98
BMAA0176	ISBma1, transposase, interruption-N	100	99	100	98
BMAA0180	isochorismatase family protein	100	98	100	93
BMAA0181	transcriptional regulator, MarR family	100	100	100	87
BMAA0183	carboxylesterase family protein	100	98	99	88
BMAA0184	oxidoreductase, short chain dehydrogenase/reductase family	100	99	100	93
BMAA0185	benzoate 1,2-dioxygenase, ferredoxin reductase component	100	99	100	95
BMAA0186	benzoate 1,2-dioxygenase beta subunit	100	100	100	93
BMAA0187	benzoate 1,2-dioxygenase, alpha subunit	100	99	100	95
BMAA0188	transcriptional regulator CatR	100	99	100	96
BMAA0191	transcriptional regulator, AraC family	100	100	100	94
BMAA0192	2,4-dienoyl-CoA reductase	100	99	100	97
BMAA0194	ubiquinol oxidase, subunit II	100	98	99	90
BMAA0195	ubiquinol oxidase, subunit I	100	99	100	96
BMAA0196	ubiquinol oxidase, subunit III	100	99	95	87
BMAA0197	ubiquinol oxidase, subunit IV	100	100	100	99
BMAA0198	muconolactone delta-isomerase	100	100	100	95
BMAA0199	catechol 1,2-dioxygenase	100	99	100	94
BMAA0200	muconate cycloisomerase	100	99	100	96
BMAA0201	transcriptional regulator CatR	100	99	100	95
BMAA0202	transcriptional regulator, AraC family	100	100	100	94
BMAA0203	ortho-halobenzoate 1,2-dioxygenase alpha-ISP protein OhbB	100	99	100	95
BMAA0204	ortho-halobenzoate 1,2-dioxygenase beta-ISP protein OhbA	100	100	99	94
BMAA0205	iron-sulfur cluster-binding protein, rieske family	100	100	99	91
BMAA0206	ferredoxin reductase	100	98	100	88
BMAA0207	HIT family protein	100	100	100	97
BMAA0208	phospholipase D, putative	100	99	100	90

BMAA0211	Na+/H+ antiporter	100	100	100	96
BMAA0212	acetolactate synthase, putative	100	99	100	98
BMAA0213	aldehyde dehydrogenase	100	99	100	95
BMAA0216	sensor histidine kinase/response regulator	100	99	100	97
BMAA0217	methyl-accepting chemotaxis protein, putative	100	100	100	95
BMAA0218	cheW domain protein	100	100	100	92
BMAA0220	chemotaxis protein cheW	100	99	100	90
BMAA0223	haloacid dehalogenase, type II	100	99	100	94
BMAA0225	voltage-gated chloride channel	100	99	100	93
BMAA0226	CAAX amino terminal protease family protein	100	99	99	87
BMAA0228	conserved hypothetical protein	100	98	100	89
BMAA0230	tryptophanyl-tRNA synthetase	100	99	100	95
BMAA0231	transcriptional regulator, AraC family	100	99	100	88
BMAA0232	DJ-1/PfpI family protein	100	99	100	94
BMAA0233	ABC transporter, permease protein/ATP-binding protein	100	99	100	93
BMAA0239	conserved domain protein	100	99	100	87
BMAA0240	conserved hypothetical protein	100	100	100	96
BMAA0241	transcriptional regulator, LysR family	100	99	100	93
BMAA0242	acyl-CoA dehydrogenase domain protein	100	99	100	96
BMAA0243	phosphotransferase enzyme family protein	100	99	100	94
BMAA0244	phosphoglycerate mutase family protein	100	99	100	96
BMAA0245	7-alpha-hydroxysteroid dehydrogenase	100	99	100	95
BMAA0246	2,5-dichloro-2,5-cyclohexadiene-1,4-diol dehydrogenase	100	100	99	94
BMAA0247	oxidoreductase, zinc-binding dehydrogenase family protein	100	99	100	97
BMAA0250	major facilitator family transporter	100	100	100	92
BMAA0251	TonB-dependent receptor	100	99	100	93
BMAA0252	hypothetical protein	100	100	98	94
BMAA0253	membrane protein, putative	100	100	100	98
BMAA0256	conserved hypothetical protein	100	100	100	94
BMAA0259	extracellular nuclease, putative	100	100	100	93
BMAA0260	conserved hypothetical protein	100	99	100	96
BMAA0261	conserved hypothetical protein	100	98	100	91
BMAA0263	RNA polymerase sigma-70 factor, ECF subfamily	100	99	100	97
BMAA0266	N-acetylmuramoyl-L-alanine amidase domain protein	100	99	100	92
BMAA0268	rubrerythrin	100	100	100	100
BMAA0269	conserved hypothetical protein	100	99	100	93
BMAA0270	conserved hypothetical protein	100	99	100	90
BMAA0271	conserved domain protein	100	99	100	93
BMAA0272	mannose-1-phosphate guanylyltransferase/mannose-6-phosphate isomerase, authentic point muta	100	98	100	93
BMAA0274	membrane protein, putative	100	99	99	94
BMAA0277	hypothetical protein	98	99	98	87
BMAA0279	transcriptional regulator, AraC family	100	98	100	90
BMAA0280	membrane protein, putative	100	100	100	93
BMAA0283	FMN-dependent dehydrogenase	100	100	100	88
BMAA0284	ebsC protein, putative	100	100	99	98
BMAA0286	alpha amylase family protein	100	100	100	91
BMAA0287	thiamine-phosphate pyrophosphorylase ThiE, putative	100	99	100	89
BMAA0288	CBS domain protein	100	99	100	95
BMAA0289	sensor histidine kinase	100	98	99	86

BMAA0290	membrane protein, putative	100	100	100	94
BMAA0291	transcriptional regulator, LysR family	100	99	100	95
BMAA0292	tautomerase enzyme family protein	100	100	100	94
BMAA0294	transcriptional regulator ModE	100	98	100	94
BMAA0297	molybdenum ABC transporter, ATP-binding protein	100	99	100	95
BMAA0298	molybdenum ABC transporter, permease protein	100	99	100	95
BMAA0299	molybdenum ABC transporter, periplasmic molybdate-binding protein	100	99	100	96
BMAA0300	PAP2 family protein	100	99	100	91
BMAA0301	polyhydroxybutyrate depolymerase domain protein	100	100	100	90
BMAA0302	hypothetical protein	100	100	100	100
BMAA0303	organic hydroperoxide resistance protein	100	99	100	95
BMAA0304	transcriptional regulator, MarR family	100	99	100	96
BMAA0306	membrane protein, putative	100	99	100	95
BMAA0307	homoserine kinase	100	99	100	96
BMAA0308	conserved hypothetical protein	100	100	100	95
BMAA0309	AMP nucleosidase	100	100	100	97
BMAA0311	portal protein, PBSX family, truncation	100	98	95	94
BMAA0318	decarboxylase family protein	100	100	100	99
BMAA0319	DNA polymerase I	100	99	100	95
BMAA0320	NADH dehydrogenase	100	100	100	93
BMAA0321	conserved hypothetical protein	100	99	100	91
BMAA0324	carboxymethylenebutenolidase	100	99	100	92
BMAA0325	rhodanese domain protein	100	100	100	94
BMAA0327	iron-sulfur cluster-binding protein, rieske family	100	100	100	97
BMAA0328	exonuclease VII, small subunit	100	100	100	97
BMAA0329	geranyltranstransferase	100	99	100	97
BMAA0330	1-deoxy-D-xylulose-5-phosphate synthase	100	100	100	98
BMAA0331	conserved hypothetical protein TIGR00294	100	100	100	98
BMAA0334	O-sialoglycoprotein endopeptidase	100	99	100	97
BMAA0335	conserved hypothetical protein TIGR00275	100	100	100	97
BMAA0336	ribosomal protein S21	100	100	100	98
BMAA0337	GatB/Yqey family protein	100	100	100	95
BMAA0338	DNA primase	100	99	100	97
BMAA0339	RNA polymerase sigma factor RpoD	100	100	100	99
BMAA0343	conserved hypothetical protein	100	100	100	95
BMAA0345	transcriptional regulator, LysR family	100	98	100	95
BMAA0348	amino acid/peptide transporter	100	99	100	97
BMAA0349	hypothetical protein	100	98	100	91
BMAA0350	DNA-binding protein	100	99	100	98
BMAA0351	oligopeptide ABC transporter, periplasmic oligopeptide-binding protein	100	99	100	95
BMAA0352	oligopeptide ABC transporter, permease protein	100	99	100	98
BMAA0353	oligopeptide ABC transporter, permease protein	100	99	100	96
BMAA0354	oligopeptide ABC transporter, ATP-binding protein	100	99	100	95
BMAA0356	outer membrane porin, OprD family	100	100	100	96
BMAA0358	dedA family protein	100	100	100	94
BMAA0360	hypothetical protein	100	99	99	91
BMAA0362	membrane protein, putative	100	99	100	98
BMAA0363	sigma-54 dependent DNA-binding transcriptional regulator	100	99	100	92
BMAA0365	H-NS histone family protein	100	98	100	96

BMAA0367	acetyltransferase, GNAT family	100	99	100	94
BMAA0368	hypothetical protein	100	100	100	97
BMAA0369	indole-3-acetamide hydrolase-related protein	100	99	100	92
BMAA0370	transcriptional regulator, IclR family	100	100	100	91
BMAA0373	fumarylacetoacetate hydrolase family protein	100	99	100	96
BMAA0376	major facilitator family transporter	100	100	100	91
BMAA0385	transcriptional regulator, LysR family	100	99	99	90
BMAA0387	transcriptional regulator, LysR family	100	99	100	90
BMAA0388	oxidoreductase, short chain dehydrogenase/reductase family	100	99	100	93
BMAA0392	conserved hypothetical protein	100	99	100	88
BMAA0394	lipoprotein, putative	100	100	100	87
BMAA0396	lipoprotein, putative	100	100	100	94
BMAA0397	conserved hypothetical protein	100	99	100	96
BMAA0398	ompA family protein	100	100	100	96
BMAA0399	lipoprotein, putative	100	99	100	95
BMAA0402	conserved hypothetical protein	100	99	100	90
BMAA0403	conserved hypothetical protein	100	99	100	91
BMAA0404	conserved hypothetical protein	100	100	100	98
BMAA0405	conserved hypothetical protein	100	100	100	98
BMAA0406	conserved hypothetical protein	96	100	99	93
BMAA0407	conserved hypothetical protein	100	99	100	95
BMAA0409	ATP-dependent Clp protease, ATP-binding subunit ClpB, putative	100	99	100	95
BMAA0410	Rhs element Vgr protein	100	99	100	97
BMAA0416	hypothetical protein	100	99	100	92
BMAA0417	cytosine/purines/uracil/thiamine/allantoin permease family protein	100	99	100	97
BMAA0418	transcriptional regulator, Sir2 family	100	98	100	89
BMAA0419	conserved hypothetical protein	100	99	100	93
BMAA0420	6-phosphogluconate dehydrogenase, decarboxylating	100	100	100	97
BMAA0421	hypothetical protein	100	99	100	91
BMAA0422	conserved hypothetical protein	100	100	100	94
BMAA0423	lipoprotein NlpD, putative	100	100	100	92
BMAA0424	membrane protein, putative	100	100	100	95
BMAA0425	cytochrome b561, putative	100	100	100	94
BMAA0427	TonB-dependent copper receptor	100	98	100	92
BMAA0429	lipase chaperone	100	99	100	88
BMAA0430	transcriptional regulator, GntR family	100	99	100	96
BMAA0431	spermidine/putrescine ABC transporter, ATP-binding protein	100	99	100	94
BMAA0432	spermidine/putrescine ABC transporter, periplasmic spermidine/putrescine-binding protein	100	99	100	95
BMAA0433	spermidine/putrescine ABC transporter, permease protein	100	100	100	97
BMAA0438	conserved hypothetical protein, truncation	100	100	100	96
BMAA0439	conserved hypothetical protein	100	100	100	99
BMAA0440	conserved hypothetical protein	100	99	100	98
BMAA0441	conserved hypothetical protein	100	99	99	96
BMAA0442	conserved hypothetical protein	100	100	100	96
BMAA0443	conserved hypothetical protein	100	100	100	93
BMAA0444	ATP-dependent Clp protease, ATP-binding subunit ClpB, putative, authentic point mutation	100	99	100	92
BMAA0445	Rhs element Vgr protein	100	97	100	94
BMAA0446	Rhs element Vgr protein	100	100	100	95
BMAA0447	conserved hypothetical protein	100	100	100	97

BMAA0448	pentapeptide repeat family protein	100	99	100	91
BMAA0449	conserved hypothetical protein	100	99	100	97
BMAA0450	conserved hypothetical protein	100	100	100	99
BMAA0451	lipoprotein, putative	100	98	100	90
BMAA0452	conserved hypothetical protein, authentic point mutation	100	99	100	98
BMAA0453	OmpA domain protein	100	99	99	92
BMAA0454	conserved hypothetical protein	100	99	100	97
BMAA0455	conserved hypothetical protein	100	100	100	97
BMAA0457	hypothetical protein	100	98	100	92
BMAA0459	rhamnosyltransferase I, subunit A	100	99	100	96
BMAA0460	rhamnosyltransferase I, subunit B	100	99	100	89
BMAA0461	multidrug resistance protein	100	99	98	95
BMAA0462	rhamnosyltransferase II	100	99	98	89
BMAA0463	RND efflux system, outer membrane lipoprotein, NodT family	100	99	98	91
BMAA0464	multidrug resistance protein, putative	100	99	99	91
BMAA0466	levansucrase	100	100	100	94
BMAA0467	raffinose repressor, putative	100	99	100	92
BMAA0468	glutathione-independent formaldehyde dehydrogenase	100	99	100	96
BMAA0469	transcriptional regulator, AraC family	100	100	96	98
BMAA0471	serine hydroxymethyltransferase 2	100	99	100	97
BMAA0472	renal dipeptidase family protein	100	100	100	96
BMAA0473	conserved hypothetical protein	100	100	100	94
BMAA0474	oxidoreductase, FAD/FMN-binding	100	99	100	97
BMAA0475	iron-sulfur cluster binding protein	100	100	100	94
BMAA0476	electron transfer flavoprotein, subunit alpha, putative	100	99	100	89
BMAA0477	conserved hypothetical protein	100	99	100	92
BMAA0478	iron-sulfur cluster-binding protein, rieske family	100	99	100	98
BMAA0479	ferredoxin, 2Fe-2S, putative	100	99	100	93
BMAA0480	ABC transporter, periplasmic glycine/betaine-binding protein	100	100	99	94
BMAA0481	amino acid transporter, putative	100	99	100	96
BMAA0482	formyltetrahydrofolate deformylase	100	100	100	97
BMAA0483	transcriptional regulator, LysR family	100	99	100	96
BMAA0484	choline sulfatase	100	100	100	95
BMAA0486	outer membrane porin, putative	100	100	100	98
BMAA0487	leucine aminopeptidase, putative	100	99	100	92
BMAA0488	metallopeptidase domain protein	100	99	100	93
BMAA0489	transcriptional regulator, AraC family	100	99	99	94
BMAA0490	ABC transporter, periplasmic glycine/betaine-binding protein	100	99	100	96
BMAA0491	conserved hypothetical protein	100	99	100	95
BMAA0492	3-hydroxyacyl-CoA dehydrogenase family protein	100	99	99	95
BMAA0493	conserved hypothetical protein	100	100	100	95
BMAA0494	lipase, putative	100	99	100	86
BMAA0495	hypothetical protein	100	100	100	95
BMAA0499	amino acid permease	100	99	100	97
BMAA0500	class III extradiol-type catecholic dioxygenase family protein	100	99	100	93
BMAA0501	3-octaprenyl-4-hydroxybenzoate carboxy-lyase	100	99	100	94
BMAA0502	glutaredoxin-related protein	100	100	100	99
BMAA0503	hemK protein	100	99	100	92
BMAA0504	peptide chain release factor 1	100	99	100	99

BMAA0505	glutamyl-tRNA reductase	100	100	100	99
BMAA0507	peptidase, M24 family protein	100	99	100	97
BMAA0508	isochorismatase family protein	100	99	100	92
BMAA0509	HAD-superfamily hydrolase	100	100	100	90
BMAA0510	ABC transporter, ATP-binding protein	100	100	100	96
BMAA0515	GABA permease	100	99	100	92
BMAA0519	GTP-binding protein YchF	100	100	100	98
BMAA0521	hypothetical protein	100	100	100	97
BMAA0524	monooxygenase family protein	100	99	100	97
BMAA0525	thiol:disulfide interchange protein DsbC, putative	100	100	100	98
BMAA0526	PDZ domain protein	100	99	100	94
BMAA0527	acyl carrier protein phosphodiesterase	100	100	100	93
BMAA0528	uracil-DNA glycosylase	100	98	100	91
BMAA0529	adenylate cyclase, putative	100	98	100	90
BMAA0530	indole-3-glycerol phosphate synthase	100	98	100	93
BMAA0531	anthranilate phosphoribosyltransferase	100	100	100	97
BMAA0532	anthranilate synthase component II	100	100	100	98
BMAA0533	anthranilate synthase component I	100	100	100	97
BMAA0534	phosphoglycolate phosphatase	100	99	100	93
BMAA0535	ribulose-phosphate 3-epimerase	100	99	100	97
BMAA0536	apaG protein	100	100	100	98
BMAA0538	membrane-bound lytic murein transglycosylase A, putative	100	100	100	96
BMAA0539	phenylacetate-coenzyme A ligase	100	99	100	97
BMAA0541	enoyl-CoA hydratase/isomerase family protein	100	100	100	93
BMAA0543	aldehyde dehydrogenase family protein	100	99	100	94
BMAA0544	enoyl-CoA hydratase/isomerase family protein	100	100	100	97
BMAA0557	arsenical resistance protein, putative	100	99	100	90
BMAA0558	arsenate reductase	100	100	100	89
BMAA0560	arsenical resistance transcriptional regulator	100	100	100	91
BMAA0561	hypothetical protein	100	100	100	91
BMAA0564	glycine betaine/L-proline ABC transporter, ATP-binding protein	100	100	100	98
BMAA0565	glycine betaine/L-proline ABC transporter, permease protein, putative	100	100	100	97
BMAA0566	transcriptional regulator, AraC family	100	99	100	96
BMAA0567	glycine betaine/L-proline ABC transporter, periplasmic glycine betaine/L-proline-binding protein	100	100	100	98
BMAA0571	conserved hypothetical protein	100	100	100	93
BMAA0572	conserved hypothetical protein	100	100	100	91
BMAA0573	hydroxypyruvate isomerase	100	99	100	96
BMAA0574	major facilitator family transporter	100	99	100	94
BMAA0575	class II aldolase/adducin domain protein	100	100	100	96
BMAA0576	conserved hypothetical protein	100	98	100	92
BMAA0577	2-hydroxy-3-oxopropionate reductase	100	99	100	95
BMAA0578	transcriptional regulator, GntR family	100	100	100	96
BMAA0579	HAD-superfamily hydrolase	100	100	100	91
BMAA0589	conserved hypothetical protein	100	100	100	96
BMAA0590	transcriptional regulator, LuxR family	100	100	100	96
BMAA0591	amino acid permease	100	99	100	96
BMAA0592	D-aminopeptidase, putative	100	98	100	96
BMAA0593	lipoprotein, putative	100	98	98	90
BMAA0595	fusaric acid resistance domain protein	100	99	100	95

BMAA0596	transcriptional regulator, LysR family	100	99	100	99
BMAA0599	outer membrane porin, putative	100	99	100	97
BMAA0601	cytosine permease	100	99	100	96
BMAA0602	DNA-binding protein	100	99	100	95
BMAA0603	cytosine deaminase	100	100	100	94
BMAA0612	twin-arginine translocation pathway signal sequence domain protein	100	99	100	90
BMAA0615	DNA-binding response regulator, LuxR family	100	99	100	90
BMAA0622	senescence marker protein-30 family protein	100	99	100	87
BMAA0624	conserved hypothetical protein	100	100	100	95
BMAA0636	transcriptional regulator, IclR family	100	100	100	95
BMAA0637	sugar ABC transporter, periplasmic sugar-binding protein	100	99	100	95
BMAA0638	sugar ABC transporter, ATP-binding protein	100	99	100	91
BMAA0639	sugar ABC transporter, permease protein	100	99	100	95
BMAA0641	hydrolase	100	100	100	89
BMAA0642	oxidoreductase, short chain dehydrogenase/reductase family	100	99	100	93
BMAA0643	fumarylacetoacetate hydrolase family protein	100	100	100	93
BMAA0644	oxidoreductase, short-chain dehydrogenase/reductase family	100	100	99	89
BMAA0645	conserved hypothetical protein	100	99	100	92
BMAA0658	transcriptional regulator, TetR family	100	99	100	91
BMAA0659	conserved hypothetical protein	100	100	100	93
BMAA0660	amino acid ABC transporter, periplasmic amino acid-binding protein, putative	100	99	100	96
BMAA0662	hemolysin III	100	99	100	97
BMAA0665	leucine-responsive regulatory protein, putative	100	100	100	99
BMAA0666	aromatic amino acid transport protein	100	99	100	95
BMAA0667	aromatic-amino-acid aminotransferase	100	100	100	97
BMAA0668	conserved hypothetical protein	100	100	100	93
BMAA0672	oxidoreductase, 2OG-Fe(II) oxygenase family	100	99	100	89
BMAA0673	hypothetical protein	100	100	100	87
BMAA0677	sensor histidine kinase	99	97	95	87
BMAA0678	response regulator	100	100	100	93
BMAA0679	CheC family protein	100	100	100	98
BMAA0681	conserved hypothetical protein	100	99	100	91
BMAA0694	microbial collagenase, putative	100	99	100	92
BMAA0696	monooxygenase family protein	100	99	100	86
BMAA0698	DNA-binding protein	100	99	100	86
BMAA0699	hypothetical protein	100	98	100	96
BMAA0701	conserved hypothetical protein	100	100	100	98
BMAA0702	hypothetical protein	100	100	100	97
BMAA0705	universal stress protein family	100	98	100	91
BMAA0706	conserved hypothetical protein	100	100	100	93
BMAA0707	universal stress protein family	100	100	100	95
BMAA0708	conserved hypothetical protein	100	100	100	96
BMAA0709	alcohol dehydrogenase, zinc-containing	100	100	100	94
BMAA0711	dedA family protein	100	99	100	95
BMAA0712	major facilitator superfamily protein	100	100	99	96
BMAA0714	oxidoreductase, short-chain dehydrogenase/reductase family	100	99	100	92
BMAA0717	amino acid permease	100	99	100	96
BMAA0719	DNA-binding protein	100	98	100	98
BMAA0720	H-NS histone family protein	100	100	100	91

BMAA0723	conserved hypothetical protein	100	99	100	93
BMAA0731	hypothetical protein	100	99	100	87
BMAA0732	conserved hypothetical protein	100	99	100	92
BMAA0734	conserved domain protein	100	100	100	90
BMAA0737	Rhs element Vgr protein	100	99	99	91
BMAA0739	conserved hypothetical protein	100	99	100	90
BMAA0740	conserved hypothetical protein	100	99	100	89
BMAA0743	conserved hypothetical protein	100	100	100	92
BMAA0744	conserved hypothetical protein	100	99	100	90
BMAA0745	sensor histidine kinase	100	99	100	86
BMAA0746	DNA-binding response regulator	100	99	99	93
BMAA0750	conserved hypothetical protein	100	97	100	88
BMAA0753	SCO1/SenC family protein	100	99	100	87
BMAA0754	conserved hypothetical protein	100	98	100	93
BMAA0755	outer membrane nitrite reductase, putative	100	99	100	93
BMAA0756	conserved hypothetical protein	100	100	100	92
BMAA0757	conserved hypothetical protein	100	100	100	98
BMAA0758	conserved hypothetical protein	100	99	100	94
BMAA0760	NH3-dependent NAD+ synthetase	100	99	100	92
BMAA0762	membrane protein, putative	100	99	100	91
BMAA0763	hypothetical protein	100	100	100	90
BMAA0770	transcriptional regulator, DeoR family	100	99	100	96
BMAA0772	hypothetical protein	100	99	100	92
BMAA0773	transcriptional regulator, TetR family, putative	100	100	100	95
BMAA0774	oxidoreductase, FAD-binding	100	99	99	92
BMAA0776	transcriptional regulator, LysR family	100	100	100	97
BMAA0777	conserved hypothetical protein	100	99	100	95
BMAA0779	piperideine-6-carboxylate dehydrogenase	100	99	100	97
BMAA0782	conserved hypothetical protein	100	100	100	96
BMAA0783	conserved hypothetical protein	100	100	100	86
BMAA0784	DNA-binding response regulator	100	100	100	92
BMAA0785	sensor histidine kinase	100	99	100	90
BMAA0787	acetyltransferase, GNAT family	100	100	96	93
BMAA0788	major facilitator family transporter	100	100	100	96
BMAA0790	conserved hypothetical protein	100	100	100	94
BMAA0801	transcriptional regulator, TetR family	100	100	100	93
BMAA0802	isovaleryl-CoA dehydrogenase	100	99	100	96
BMAA0803	carboxyl transferase domain protein	100	99	100	97
BMAA0804	enoyl-CoA hydratase/isomerase family protein	100	100	100	96
BMAA0805	acetyl-CoA carboxylase, biotin carboxylase, putative	100	99	100	95
BMAA0806	hypothetical protein	100	100	100	97
BMAA0808	hypothetical protein	100	99	100	97
BMAA0809	hypothetical protein	100	99	100	94
BMAA0814	glutaminase A	100	100	100	92
BMAA0815	DNA-binding response regulator	100	100	100	96
BMAA0816	sensor histidine kinase	100	100	99	91
BMAA0818	outer membrane protein, OMP85 family, putative	100	99	100	87
BMAA0819	rod shape-determining protein MreB, putative	100	100	100	96
BMAA0822	conserved hypothetical protein	100	99	100	96

BMAA0826	glyoxalase family protein	100	99	100	92
BMAA0827	glutathione S-transferase, putative	100	99	100	91
BMAA0828	conserved hypothetical protein	100	99	100	94
BMAA0829	sensor histidine kinase	100	100	100	94
BMAA0830	DNA-binding response regulator	100	100	100	97
BMAA0831	conserved hypothetical protein	100	100	100	97
BMAA0833	immunity protein, putative	100	96	100	87
BMAA0834	rhodanese-like domain protein	100	98	100	92
BMAA0835	transcriptional regulator, AraC family	100	99	100	95
BMAA0836	peptidase, M20/M25/M40 family	100	99	99	96
BMAA0839	membrane protein, putative	100	99	100	93
BMAA0847	ISBma3, transposase, truncation	100	95	100	89
BMAA0848	4-hydroxyphenylpyruvate dioxygenase, putative	100	99	100	88
BMAA0849	3-dehydroquinate dehydratase, type II	100	99	100	92
BMAA0850	shikimate 5-dehydrogenase, putative	100	98	100	91
BMAA0851	D-galactonate transporter, putative	100	100	100	95
BMAA0852	aminotransferase, class V	100	99	100	94
BMAA0855	ABC transporter, periplasmic substrate-binding protein	100	99	100	97
BMAA0856	ABC transporter, ATP-binding protein	100	99	100	97
BMAA0857	ABC transporter, permease protein	100	100	100	93
BMAA0858	ABC transporter, permease protein	100	98	100	94
BMAA0859	phosphonoacetate hydrolase	100	100	100	93
BMAA0860	glyceraldehyde-3-phosphate dehydrogenase, NADP-dependent, putative	100	100	100	97
BMAA0861	conserved hypothetical protein	100	100	97	93
BMAA0874	conserved hypothetical protein	100	99	97	94
BMAA0875	oxidoreductase, aldo/keto reductase family	100	99	100	94
BMAA0876	D-beta-hydroxybutyrate dehydrogenase	100	100	97	97
BMAA0879	aromatic-amino-acid aminotransferase	100	100	100	99
BMAA0880	excinuclease ABC, B subunit	100	100	100	98
BMAA0881	antigen, putative	100	100	100	97
BMAA0882	conserved hypothetical protein	100	100	100	96
BMAA0883	iron permease, FTR1 family	100	100	100	97
BMAA0884	membrane protein, putative	100	99	100	94
BMAA0885	glcG protein	100	100	100	94
BMAA0892	sarcosine oxidase, alpha subunit, truncation	100	99	100	97
BMAA0894	dihydroneopterin aldolase, putative	100	98	98	92
BMAA0896	ribosomal protein S21	100	98	100	95
BMAA0899	cold-shock domain family protein	100	100	100	97
BMAA0907	conserved hypothetical protein	100	100	100	92
BMAA0908	sensory box histidine kinase	100	99	100	88
BMAA0909	response regulator	100	100	99	95
BMAA0910	sensory box histidine kinase/response regulator	100	99	100	95
BMAA0912	hypothetical protein	100	99	100	94
BMAA0914	choline dehydrogenase	100	99	100	97
BMAA0915	betaine aldehyde dehydrogenase	100	99	100	97
BMAA0916	regulatory protein betI	100	99	99	92
BMAA0919	rhamnosyltransferase 1, subunit A	100	99	100	96
BMAA0921	rhamnosyltransferase I, subunit B	100	99	100	89
BMAA0922	drug resistance transporter, EmrB/QacA family	100	99	98	95

BMAA0923	rhamnosyltransferase family protein	100	99	98	89
BMAA0924	RND efflux system, outer membrane lipoprotein, NodT family	100	99	98	91
BMAA0925	multidrug resistance protein	100	99	100	89
BMAA0927	prolyl oligopeptidase family protein	100	99	97	90
BMAA0934	cytochrome b561 family protein	100	98	100	93
BMAA0937	transcriptional regulator, ArsR family	100	100	100	92
BMAA0938	YeeE/YedE family protein	100	97	100	88
BMAA0939	membrane protein, putative	100	100	100	94
BMAA0940	acetyltransferase, GNAT family	100	98	100	89
BMAA0942	conserved hypothetical protein	100	100	100	90
BMAA0943	conserved hypothetical protein	100	98	100	86
BMAA0944	conserved hypothetical protein	100	100	100	93
BMAA0946	Ser/Thr protein phosphatase family protein	100	98	100	94
BMAA0947	hydrolase	100	98	100	88
BMAA0949	transcriptional regulator, GntR family	100	100	100	97
BMAA0951	leucine-responsive regulatory protein, putative	100	100	100	95
BMAA0952	1-aminocyclopropane-1-carboxylate deaminase	100	100	100	97
BMAA0966	membrane protein, putative	100	99	100	89
BMAA0968	phosphoesterase family protein	100	99	99	87
BMAA0970	esterase, putative	100	100	100	90
BMAA0971	choline/carnitine/betaine transporter	100	99	100	96
BMAA0972	SIS domain protein	100	99	99	94
BMAA0973	asparaginase family protein	100	97	100	91
BMAA0974	dipeptide ABC transporter, ATP-binding protein, putative	100	98	100	90
BMAA0975	dipeptide ABC transporter periplasmic dipeptide-binding protein, putative	100	100	100	95
BMAA0976	dipeptide ABC transporter, permease protein, putative	100	99	100	97
BMAA0977	dipeptide ABC transporter, permease protein, putative	100	99	100	96
BMAA0979	dipeptide transport protein, putative	100	100	100	93
BMAA0980	protocatechuate 3,4-dioxygenase, alpha subunit	100	99	100	92
BMAA0981	protocatechuate 3,4-dioxygenase, beta subunit	100	100	100	97
BMAA0982	transcriptional regulator, LysR family	100	99	99	91
BMAA0984	GGDEF domain protein	100	99	100	91
BMAA0986	O-methyltransferase family protein	100	99	100	90
BMAA0987	phospho-2-dehydro-3-deoxyheptonate aldolase	100	100	100	95
BMAA0988	DGPF domain protein	100	100	96	90
BMAA0990	amino acid permease	100	99	100	92
BMAA0995	hypothetical protein	100	99	100	94
BMAA0996	gamma-glutamyltranspeptidase	100	99	100	95
BMAA0997	outer membrane protein TolC, putative	100	99	100	97
BMAA0998	hypothetical protein	100	100	100	98
BMAA1000	ABC transporter, permease/ATP-binding protein	100	99	100	98
BMAA1001	hypothetical protein	100	100	100	96
BMAA1002	secretion protein, putative	100	99	100	98
BMAA1003	conserved hypothetical protein	100	99	100	94
BMAA1006	threonine dehydratase, biosynthetic, truncation	100	98	100	91
BMAA1010	conserved hypothetical protein	100	100	100	93
BMAA1027	sensor histidine kinase IrlS	100	99	100	90
BMAA1028	DNA-binding response regulator IrlR	100	100	100	97
BMAA1029	heavy metal efflux pump CzcA	100	99	100	93

BMAA1030	heavy metal resistance protein CzcB	100	99	100	88
BMAA1036	conserved hypothetical protein	100	99	100	91
BMAA1037	YbaK / prolyl-tRNA synthetases-associated domain family protein	100	100	100	97
BMAA1039	serine protease, rhomboid family	100	99	100	87
BMAA1040	ATP-dependent metalloprotease, FtsH family	100	99	100	91
BMAA1042	conserved hypothetical protein	100	99	100	95
BMAA1043	conserved hypothetical protein	100	99	100	96
BMAA1044	efflux transporter, RND family, MFP subunit	100	99	100	96
BMAA1045	hydrophobe/amphiphile efflux family protein	100	99	100	98
BMAA1046	RND efflux system, outer membrane lipoprotein, NodT family	100	99	98	95
BMAA1048	GTP cyclohydrolase II	100	100	100	99
BMAA1049	WD domain protein	100	99	100	98
BMAA1050	conserved hypothetical protein	97	99	97	96
BMAA1056	penicillin-binding protein	100	99	100	96
BMAA1057	periplasmic sugar-binding protein, putative	100	100	100	97
BMAA1058	oxidoreductase, short chain dehydrogenase/reductase family	100	100	100	95
BMAA1059	potassium efflux system protein, putative	100	98	100	91
BMAA1062	conserved hypothetical protein	100	99	100	96
BMAA1063	conserved domain protein	100	99	100	91
BMAA1064	hypothetical protein	100	98	100	89
BMAA1069	transcriptional regulator, DeoR family	100	100	100	88
BMAA1070	transporter, putative	100	100	100	87
BMAA1073	sugar ABC transporter, periplasmic sugar-binding protein, putative	100	98	100	93
BMAA1074	sugar ABC transporter, permease protein, putative	100	100	100	94
BMAA1075	sugar ABC transporter, permease protein, putative	100	99	100	95
BMAA1076	oxidoreductase, zinc-binding dehydrogenase family	100	99	100	96
BMAA1077	sugar ABC transporter, ATP-binding protein, putative	100	98	98	90
BMAA1078	transcriptional regulator, AraC family	100	100	100	90
BMAA1079	xylulokinase	100	97	100	95
BMAA1084	nitrate reductase/sulfite reductase flavoprotein alpha-component, putative	100	99	100	91
BMAA1085	nitrite reductase [NAD(P)H], small subunit	100	100	100	92
BMAA1086	nitrite reductase [NAD(P)H], large subunit	100	99	100	93
BMAA1087	nitrate transporter, putative	100	99	100	96
BMAA1089	uroporphyrin-III C-methyltransferase	100	100	97	87
BMAA1090	response regulator NasT	100	100	100	93
BMAA1091	regulatory protein NasS, putative	100	95	100	92
BMAA1096	lipoprotein, putative	100	99	100	91
BMAA1098	acetylpolyamine aminohydrolase, putative	100	99	100	92
BMAA1099	N-carbamyl-L-amino acid amidohydrolase	100	99	100	86
BMAA1100	major facilitator family transporter	100	99	100	96
BMAA1101	transcriptional regulator, LysR family, frameshift	100	99	100	98
BMAA1104	oxidoreductase, short chain dehydrogenase/reductase family	100	100	100	92
BMAA1112	conserved hypothetical protein	100	99	100	92
BMAA1115	ompA family protein	100	100	100	96
BMAA1122	porin OpcP1, putative	100	99	100	99
BMAA1123	ISBma1, transposase	100	99	100	98
BMAA1125	MOSC domain protein	100	100	99	93
BMAA1126	sensor histidine kinase	100	99	100	90
BMAA1127	DNA-binding response regulator TctD	100	99	100	98

BMAA1128	ABC transporter, periplasmic substrate-binding protein	100	100	100	95
BMAA1130	ABC transporter, ATP-binding protein	100	100	100	96
BMAA1131	porin	100	99	100	94
BMAA1135	2-oxo-hepta-3-ene-1,7-dioate hydratase	100	100	100	97
BMAA1136	5-carboxymethyl-2-hydroxymuconate delta isomerase	100	100	100	96
BMAA1137	3,4-dihydroxyphenylacetate 2,3-dioxygenase	100	100	100	98
BMAA1138	5-carboxy-2-hydroxymuconate semialdehyde dehydrogenase	100	99	100	96
BMAA1139	5-carboxymethyl-2-oxo-hex-3-ene-1,7-dioate decarboxylase, putative	100	100	100	97
BMAA1140	2-hydroxyhepta-2,4-diene-1,7-dioate isomerase, putative	100	99	100	93
BMAA1141	homoprotocatechuate degradative operon repressor	100	99	100	97
BMAA1142	conserved hypothetical protein	100	99	100	91
BMAA1143	conserved hypothetical protein	100	99	100	86
BMAA1144	DNA-binding response regulator	100	98	100	95
BMAA1145	sensor histidine kinase	100	99	100	92
BMAA1146	monoxygenase	100	100	99	95
BMAA1148	acyl-CoA dehydrogenase domain protein	100	99	100	93
BMAA1150	hypothetical protein	100	100	100	92
BMAA1153	transcriptional regulator, AraC family	100	100	100	93
BMAA1154	isoquinoline 1-oxidoreductase, alpha subunit	100	99	100	98
BMAA1154.1	isoquinoline 1-oxidoreductase, beta subunit	100	99	100	93
BMAA1158	asparagine synthase (glutamine-hydrolyzing)	100	99	100	96
BMAA1159	conserved hypothetical protein	100	100	100	94
BMAA1161	transcriptional regulator, LuxR family	100	100	100	94
BMAA1166	trehalase	100	99	100	94
BMAA1168	drug resistance transporter, EmrB/QacA family	100	99	100	97
BMAA1169	conserved hypothetical protein	100	99	99	94
BMAA1170	conserved hypothetical protein	100	100	100	95
BMAA1180	TonB-dependent siderophore receptor	100	99	100	93
BMAA1181	FecR family protein	97	97	95	86
BMAA1182	RNA polymerase sigma-70 factor, ECF family	100	99	100	94
BMAA1187	RNA polymerase sigma-70 factor, ECF subfamily	100	99	100	91
BMAA1190	fumarylacetoacetate hydrolase family protein	100	100	100	95
BMAA1191	CAAX protease family protein	100	100	100	95
BMAA1193	conserved hypothetical protein TIGR00427	100	96	97	93
BMAA1195	calcium/proton exchanger	100	99	100	96
BMAA1196	transcriptional regulator, LysR family	100	100	100	98
BMAA1197	HPP family protein	100	99	100	93
BMAA1198	conserved hypothetical protein	100	99	100	95
BMAA1199	voltage-gated chloride channel/CBS domain protein	100	99	100	90
BMAA1213	ISBma1, transposase	100	99	100	98
BMAA1232	hypothetical protein	100	97	100	91
BMAA1233	hypothetical protein	100	97	100	91
BMAA1236	conserved hypothetical protein	100	98	100	91
BMAA1237	UDP-N-acetylglucosamine 1-carboxyvinyltransferase, putative	100	100	100	98
BMAA1238	histidine ABC transporter, ATP-binding protein	100	99	100	93
BMAA1239	histidine ABC transporter, permease protein	100	99	100	97
BMAA1240	histidine ABC transporter, permease protein	100	100	100	95
BMAA1241	membrane protein, putative	100	100	100	93
BMAA1242	outer membrane porin, putative	100	99	100	96

BMAA1243	DoxD-like family protein	100	98	98	90
BMAA1244	cytochrome c family protein	100	98	100	90
BMAA1245	conserved hypothetical protein	100	100	100	96
BMAA1246	oxidoreductase, putative	100	99	100	93
BMAA1248	serine metalloprotease MrpA	100	99	100	92
BMAA1250	magnesium-translocating P-type ATPase	100	99	100	94
BMAA1251	conserved hypothetical protein	100	100	100	90
BMAA1253	transcriptional regulator, GntR family	100	99	100	96
BMAA1254	molybdopterin oxidoreductase, alpha subunit	100	99	100	93
BMAA1255	conserved hypothetical protein	100	100	100	94
BMAA1258	manganese/iron transporter, NRAMP family	100	99	100	93
BMAA1259	cupin family protein	100	99	100	96
BMAA1261	bacterial extracellular solute-binding protein, family 5	100	99	100	94
BMAA1262	hypothetical protein	100	100	100	88
BMAA1263	serine protease, subtilase family	100	99	100	91
BMAA1265	hypothetical protein	100	99	99	86
BMAA1267	hypothetical protein	100	100	100	87
BMAA1268	conserved hypothetical protein	100	98	100	97
BMAA1269	Rhs element Vgr protein	100	100	100	97
BMAA1272	conserved hypothetical protein	100	100	100	93
BMAA1273	D-alanyl-D-alanine dipeptidase	100	98	100	88
BMAA1274	ABC transporter, periplasmic substrate-binding protein	100	100	100	93
BMAA1275	ABC transporter, permease protein	100	99	100	95
BMAA1276	ABC transporter, permease protein	100	99	100	94
BMAA1277	ABC transporter, ATP-binding protein	100	98	100	91
BMAA1278	ABC transporter, ATP-binding protein	100	99	100	92
BMAA1281	transcriptional regulator, LysR family	100	99	100	89
BMAA1283	class A beta-lactamase	100	100	100	89
BMAA1285	transcriptional regulator, LysR family	100	100	100	98
BMAA1286	outer membrane porin, putative	100	100	100	97
BMAA1288	opgC protein, putative	100	99	100	92
BMAA1289	esterase, putative	100	98	100	94
BMAA1290	ornithine cyclodeaminase/mu-crystallin family protein	100	99	100	86
BMAA1291	4-carboxymuconolactone decarboxylase	100	98	100	92
BMAA1292	hydrolase, alpha/beta fold family	100	100	98	91
BMAA1293	TrkA domain protein	100	99	100	98
BMAA1294	conserved hypothetical protein	100	99	100	94
BMAA1295	conserved hypothetical protein	100	99	100	97
BMAA1297	hypothetical protein	100	97	100	97
BMAA1299	drug resistance transporter, EmrB/QacA family	100	98	100	97
BMAA1302	transcriptional regulator, MerR family	100	100	100	98
BMAA1303	aliphatic compound ABC transporter, periplasmic substrate-binding protein	100	99	100	88
BMAA1305	membrane protein, putative	100	100	100	91
BMAA1311	conserved hypothetical protein	100	98	100	90
BMAA1313	N-hydroxyarylamine O-acetyltransferase	100	99	99	91
BMAA1317	trans-sulfuration enzyme family protein	100	100	100	96
BMAA1319	transcriptional regulator, AsnC family	100	100	99	98
BMAA1320	amidase family protein	100	99	100	91
BMAA1322	UspA family protein	100	100	100	95

BMAA1323	outer membrane lipoprotein, OmpA/SmpA/OmlA family	100	100	100	91
BMAA1324	haemagluttinin family protein	100	99	100	93
BMAA1325	major facilitator family transporter	100	99	100	94
BMAA1326	pyridine nucleotide-disulfide oxidoreductase, class I	100	99	100	90
BMAA1327	glyoxalase family protein	100	99	100	93
BMAA1328	aldehyde dehydrogenase family protein	100	99	100	96
BMAA1329	conserved hypothetical protein	100	99	100	94
BMAA1330	acetolactate synthase, large subunit, putative	100	98	100	96
BMAA1331	oxidoreductase, short chain dehydrogenase/reductase family	100	99	100	94
BMAA1332	transcriptional regulator, IclR family	100	100	100	96
BMAA1333	hydrolase, alpha/beta fold family	100	99	99	88
BMAA1334	conserved hypothetical protein	100	99	100	98
BMAA1335	oxidoreductase, short chain dehydrogenase/reductase family	100	99	100	93
BMAA1336	iron-sulfur cluster-binding protein, rieske family	100	99	100	97
BMAA1337	conserved hypothetical protein	100	98	99	95
BMAA1338	naphthalene 1,2-dioxygenase system ferredoxin component	100	100	100	99
BMAA1339	outer membrane porin OpcP, putative	100	99	100	94
BMAA1340	transcriptional regulator, IclR family	100	99	100	94
BMAA1341	oxidoreductase, 2OG-Fe(II) oxygenase family	100	98	100	91
BMAA1342	nitroreductase family protein	100	100	100	96
BMAA1343	Ser/Thr protein phosphatase family protein	100	99	100	92
BMAA1344	MgtC family protein	100	100	100	99
BMAA1345	N-acyl homoserine lactone transcriptional regulator	100	100	100	98
BMAA1346	hypothetical protein	100	98	100	91
BMAA1347	autoinducer synthetase family protein	100	99	100	96
BMAA1348	conserved domain protein	100	99	100	90
BMAA1349	GTP cyclohydrolase II	100	99	100	98
BMAA1350	CBS domain protein	100	100	99	98
BMAA1351	hypothetical protein	100	100	100	90
BMAA1352	nucleoside diphosphate kinase regulator, putative	100	100	100	91
BMAA1353	outer membrane porin OpcP	100	98	100	99
BMAA1355	transcriptional regulator, ArsR family	100	100	100	97
BMAA1356	N-ethylmaleimide reductase	100	100	100	93
BMAA1360	outer membrane porin, putative	100	99	100	88
BMAA1361	transcriptional regulator, araC family	100	100	100	95
BMAA1363	amino acid permease	100	99	100	94
BMAA1364	adenosylmethionine-8-amino-7-oxononanoate aminotransferase, putative	100	99	100	98
BMAA1365	phenylacetaldehyde dehydrogenase	100	99	100	95
BMAA1366	transcriptional regulator, LuxR family	100	99	100	92
BMAA1367	oxidoreductase, short chain dehydrogenase/reductase family	100	100	100	92
BMAA1368	conserved hypothetical protein	100	99	100	88
BMAA1370	oxidoreductase, aldo/keto reductase family	100	99	100	94
BMAA1372	conserved hypothetical protein	100	100	100	88
BMAA1373	transcriptional regulator, LysR family	100	98	100	94
BMAA1374	flagellar hook-associated protein 2	100	99	100	93
BMAA1376	DNA repair helicase, truncation	100	98	100	93
BMAA1379	methylmalonate-semialdehyde dehydrogenase	100	99	100	91
BMAA1380	omega-amino acid--pyruvate aminotransferase	100	99	100	96
BMAA1381	transcriptional regulator, LysR family	100	100	100	97

BMAA1384	hypothetical protein	100	98	100	93
BMAA1385	2,4'-dihydroxyacetophenone dioxygenase	100	100	100	95
BMAA1394	AMP-binding domain protein	100	99	100	96
BMAA1397	conserved hypothetical protein	100	100	100	91
BMAA1398	transcriptional regulator, GntR family	100	99	100	93
BMAA1399	D-beta-hydroxybutyrate dehydrogenase	100	99	100	94
BMAA1400	osmotically-inducible lipoprotein OsmE	100	99	100	89
BMAA1401	membrane protein, putative	100	99	100	91
BMAA1403	enoyl-(acyl-carrier-protein) reductase	100	100	100	97
BMAA1405	xenobiotic reductase A	100	99	100	95
BMAA1406	DJ-1/PfpI family protein	100	100	100	96
BMAA1408	transcriptional regulator, TetR family	100	100	100	93
BMAA1414	hypothetical protein	100	97	100	94
BMAA1415	transcriptional regulator, AraC family	100	99	98	87
BMAA1416	oxidoreductase, putative	100	99	100	92
BMAA1417	2Fe-2S iron-sulfur cluster binding domain	100	97	100	90
BMAA1418	oxidoreductase, FAD-binding family protein	100	98	99	91
BMAA1419	proline racemase, putative	100	99	100	92
BMAA1420	dihydrodipicolinate synthetase family protein	100	99	100	97
BMAA1422	aldehyde dehydrogenase family protein	100	99	100	92
BMAA1423	malate/L-lactate dehydrogenase family protein	100	99	100	93
BMAA1424	major facilitator family transporter	100	99	100	97
BMAA1426	DoxD-like family protein	100	100	100	91
BMAA1428	lactate permease family protein	100	99	100	97
BMAA1429	conserved hypothetical protein	100	99	100	88
BMAA1430	iron-sulfur cluster binding protein	100	99	100	96
BMAA1431	cysteine-rich domain protein	100	99	100	95
BMAA1432	transcriptional regulator, GntR family	100	100	100	96
BMAA1435	hypothetical protein	100	100	100	92
BMAA1436	monoxygenase	100	97	100	92
BMAA1440	monoxygenase	100	99	100	90
BMAA1443	autoinducer-binding transcriptional regulator, LuxR family	100	100	100	97
BMAA1447	conserved domain protein	100	100	100	97
BMAA1449	syringomycin synthesis regulator SyrP, putative	100	99	100	95
BMAA1450	adenosylmethionine-8-amino-7-oxononanoate aminotransferase, putative	100	99	100	95
BMAA1451	aldehyde dehydrogenase family protein	100	98	100	95
BMAA1455	diaminopimelate decarboxylase, putative	100	99	100	92
BMAA1456	long-chain-fatty-acid--CoA ligase, putative	100	99	100	89
BMAA1457	lipoprotein, putative	100	100	100	95
BMAA1458	malonyl CoA-acyl carrier protein transacylase	100	100	99	86
BMAA1459	AMP-binding domain protein	100	99	100	89
BMAA1460	auxin efflux carrier, putative	100	99	100	93
BMAA1462	transcriptional regulator, GntR family	100	99	100	99
BMAA1463	sodium:dicarboxylate symporter family protein	100	99	100	97
BMAA1464	RND efflux system, outer membrane lipoprotein, NodT family	100	99	100	97
BMAA1465	hydrophobe/amphiphile efflux family protein	100	100	100	99
BMAA1466	efflux transporter, RND family, MFP subunit	100	100	100	96
BMAA1467	carboxylesterase, putative	100	99	100	93
BMAA1468	transcriptional regulator, LysR family	100	100	100	97

BMAA1471	lipoprotein, putative	100	100	100	94
BMAA1473	selenide, water dikinase	100	99	100	94
BMAA1474	membrane protein, putative	100	99	100	89
BMAA1480	4-aminobutyrate aminotransferase	100	99	100	97
BMAA1481	succinate-semialdehyde dehydrogenase	100	99	100	98
BMAA1482	hypothetical protein	100	99	100	98
BMAA1484	conserved hypothetical protein, authentic frameshift	100	97	100	91
BMAA1487	methyl-accepting chemotaxis protein	100	99	100	94
BMAA1491	transcriptional regulator, IclR family	100	99	100	96
BMAA1492	transcriptional regulator, TetR family	100	99	98	97
BMAA1496	amino acid ABC transporter, periplasmic amino acid-binding protein	100	99	100	97
BMAA1499	conserved hypothetical protein	100	100	100	96
BMAA1501	D-methionine-binding lipoprotein metQ	100	100	100	91
BMAA1502	porin, interruption-N	100	98	100	96
BMAA1505	porin, interruption-C	100	99	100	96
BMAA1508	transcriptional regulator, LysR family	100	99	100	94
BMAA1509	pirin family protein	100	100	100	91
BMAA1510	conserved hypothetical protein	100	99	100	92
BMAA1511	conserved hypothetical protein	100	97	100	89
BMAA1517	transcriptional regulator, araC family	100	99	95	89
BMAA1518	hypothetical protein	100	100	100	91
BMAA1518.1	DNA-binding response regulator	100	100	100	95
BMAA1520	type III secretion chaperone BicP	100	100	100	93
BMAA1521	BopA protein	100	99	100	90
BMAA1523	type III secretion target BopE	100	99	100	95
BMAA1524	BapC protein	100	100	100	90
BMAA1525	BapB protein	100	98	100	93
BMAA1528	BprD protein	100	100	100	91
BMAA1529	DNA-binding protein BprA	100	100	100	91
BMAA1530	type III secretion target BipC	100	100	100	86
BMAA1531	BipB protein	100	100	100	90
BMAA1532	type III secretion chaperone BicA	100	100	100	94
BMAA1534	type III secretion system protein BsaY	100	99	99	92
BMAA1535	type III secretion system protein BsaX	100	100	100	100
BMAA1536	type III secretion system protein BsaW	100	100	100	95
BMAA1537	type III secretion system protein BsaV	100	97	100	86
BMAA1539	BsaT protein	100	99	99	86
BMAA1540	type III secretion system protein BsaS	100	99	100	94
BMAA1541	type III secretion system protein BsaR	100	100	100	97
BMAA1542	type III secretion system protein BsaQ	100	100	100	97
BMAA1543	type III secretion system protein BsaP	100	98	100	93
BMAA1544	type III secretion system protein BsaO	100	98	100	94
BMAA1545	type III secretion system transcriptional regulator BsaN	100	99	100	97
BMAA1547	type III secretion system protein BsaM	100	99	100	92
BMAA1548	type III secretion system protein BsaL	100	98	100	97
BMAA1549	type III secretion system protein BsaK	100	99	100	90
BMAA1550	type III secretion system BasJ	100	99	100	93
BMAA1551	oxygen-regulated invasion protein OrgA	100	99	100	92
BMAA1557	hypothetical protein	100	99	100	94

BMAA1559	thermolysin metallopeptidase	100	99	100	95
BMAA1563	metabolite:proton symporter family protein	100	99	100	95
BMAA1564	transcriptional regulator, LysR family	100	100	100	95
BMAA1565	dehydrogenase, FMN-dependent family	100	100	100	92
BMAA1566	serine-type carboxypeptidase family protein	100	100	100	94
BMAA1569	LysE family protein	100	97	100	90
BMAA1570	transcriptional regulator, AsnC family	100	100	100	94
BMAA1572	phosphate transporter family protein	100	99	100	94
BMAA1573	conserved hypothetical protein	100	99	100	89
BMAA1576	transcriptional regulator, LuxR family	100	100	100	96
BMAA1577	N-acyl homoserine lactone synthase	100	99	100	91
BMAA1578	oxidoreductase, FAD/FMN-binding	100	99	100	94
BMAA1580	taurine ABC transporter, permease protein	100	99	100	92
BMAA1581	taurine ABC transporter, ATP-binding protein	100	98	100	96
BMAA1582	taurine ABC transporter, periplasmic taurine-binding protein	100	99	100	92
BMAA1583	taurine dioxygenase-related protein	98	97	96	91
BMAA1584	conserved hypothetical protein	100	99	100	93
BMAA1585	glycosyl transferase, group 2 family protein	100	99	100	92
BMAA1588	cellulose synthase operon protein C	100	98	100	88
BMAA1590	cellulose synthase, cyclic di-GMP binding subunit	100	99	99	89
BMAA1596	transcriptional regulator, LysR family	100	100	100	90
BMAA1597	agmatinase	100	100	100	95
BMAA1600	conserved hypothetical protein	100	99	100	92
BMAA1602	type II/III secretion system protein	100	99	100	93
BMAA1604	PilM, putative	100	100	100	86
BMAA1606	type IV pilus biogenesis protein	100	100	100	95
BMAA1607	type II/IV secretion system protein	100	99	100	94
BMAA1608	hypothetical protein	100	100	100	93
BMAA1609	PilO, putative	100	100	100	92
BMAA1610	type IV pilus biogenesis protein PilN, putative	100	99	97	90
BMAA1614	DNA-binding response regulator	100	100	100	96
BMAA1615	sensor histidine kinase	100	99	100	90
BMAA1621	regulatory protein HrpB	100	99	100	90
BMAA1622	conserved hypothetical protein	100	99	100	86
BMAA1624	conserved hypothetical protein	100	100	100	91
BMAA1627	type III secretion inner membrane protein SctS	100	100	100	95
BMAA1628	type III secretion inner membrane protein SctR	100	100	100	97
BMAA1630	type III secretion inner membrane protein SctV	100	99	100	96
BMAA1631	type III secretion inner membrane protein	100	99	100	89
BMAA1636	type III secretion inner membrane protein SctL	100	100	100	94
BMAA1637	type III secretion cytoplasmic ATPase SctN	100	99	100	98
BMAA1657	transcriptional regulator, LysR family	100	100	100	93
BMAA1658	drug resistance transporter, EmrB/QacA family	100	98	100	88
BMAA1660	sensory box sensor histidine kinase	100	99	100	94
BMAA1661	conserved hypothetical protein	100	99	99	95
BMAA1662	response regulator	100	99	99	86
BMAA1664	lectin repeat domain protein	100	99	100	91
BMAA1666	conserved domain protein	100	99	100	87
BMAA1667	patatin-like phospholipase	100	99	100	88

BMAA1669	cytochrome P450	100	99	100	92
BMAA1678	selenocysteine-specific translation elongation factor	100	99	100	92
BMAA1678.1	L-seryl-tRNA selenium transferase	100	99	100	91
BMAA1680	formate dehydrogenase accessory protein	100	99	100	94
BMAA1681	formate dehydrogenase, gamma subunit	100	99	100	97
BMAA1682	formate dehydrogenase, beta subunit	100	99	100	98
BMAA1683	formate dehydrogenase, alpha subunit, selenocysteine-containing, authentic point mutation	100	99	100	96
BMAA1685	conserved hypothetical protein	100	100	100	88
BMAA1696	conserved hypothetical protein	100	100	100	93
BMAA1697	ribonuclease BN, putative	100	99	100	94
BMAA1700	DNA-binding protein BpH2	100	100	100	98
BMAA1701	acyltransferase family protein	100	99	100	88
BMAA1705	glycosyl transferase, group 1 family protein	100	99	100	95
BMAA1706	polysaccharide biosynthesis family protein	100	99	100	96
BMAA1708	acyltransferase family protein	100	100	100	96
BMAA1709	GDP-mannose 4,6-dehydratase	100	99	100	95
BMAA1711	conserved hypothetical protein	100	99	100	94
BMAA1713	O-succinylhomoserine sulfhydrylase	100	99	100	97
BMAA1714	amidophosphoribosyltransferase	100	99	100	98
BMAA1715	CvpA family protein	100	100	100	96
BMAA1717	folylpolyglutamate synthase/dihydrofolate synthase	100	99	100	95
BMAA1718	acetyl-CoA carboxylase, carboxyl transferase, beta subunit	100	100	100	98
BMAA1719	tryptophan synthase, alpha subunit	100	99	100	93
BMAA1720	N-6 adenine-specific DNA methylase	100	100	100	95
BMAA1721	tryptophan synthase, beta subunit	100	99	99	96
BMAA1722	N-(5'phosphoribosyl)anthranilate isomerase	100	99	100	89
BMAA1723	tRNA pseudouridine synthase A	100	100	100	97
BMAA1725	aspartate-semialdehyde dehydrogenase	100	100	100	98
BMAA1726	3-isopropylmalate dehydrogenase	100	100	100	98
BMAA1727	3-isopropylmalate dehydratase, small subunit	100	100	100	97
BMAA1728	entericidin	100	97	100	91
BMAA1729	3-isopropylmalate dehydratase, large subunit	100	99	100	98
BMAA1736	Bkd operon transcriptional regulator	100	100	100	96
BMAA1737	2-oxo-acid dehydrogenase E1 component	100	99	100	94
BMAA1738	dioxygenase, putative	100	99	99	92
BMAA1739	conserved domain protein	100	100	98	86
BMAA1743	transcriptional regulator, AraC family	100	99	100	90
BMAA1744	citrate synthase I	100	100	100	99
BMAA1745	conserved hypothetical protein	100	98	100	98
BMAA1746	succinate dehydrogenase, iron-sulfur protein	100	100	100	99
BMAA1747	succinate dehydrogenase, flavoprotein subunit	100	99	100	99
BMAA1748	succinate dehydrogenase, hydrophobic membrane anchor protein	100	100	100	98
BMAA1749	succinate dehydrogenase, cytochrome b556 subunit	100	100	100	98
BMAA1750	transcriptional regulator, GntR family	100	100	100	97
BMAA1751	malate dehydrogenase	100	100	100	99
BMAA1752	lyase, putative	100	99	100	94
BMAA1753	conserved hypothetical protein	100	99	100	95
BMAA1754	2-methylcitrate dehydratase	100	99	100	95
BMAA1755	aconitate hydratase 1	100	100	100	98

BMAA1761	conserved hypothetical protein	100	99	100	86
BMAA1763	coenzyme A transferase	100	100	100	99
BMAA1764	CDP-alcohol phosphatidyltransferase family protein	100	100	100	94
BMAA1766	membrane protein, putative	100	100	100	95
BMAA1767	conserved hypothetical protein	100	99	100	98
BMAA1768	phosphoenolpyruvate phosphomutase	100	99	100	96
BMAA1769	3-phosphonopyruvate decarboxylase	100	99	100	95
BMAA1770	2-aminoethylphosphonate-pyruvate transaminase	100	99	100	96
BMAA1773	membrane protein, putative	100	100	100	92
BMAA1775	transcriptional regulator, GntR family	100	99	100	86
BMAA1776	transglutaminase domain protein	100	98	100	91
BMAA1777	conserved hypothetical protein	100	99	100	96
BMAA1778	transglutaminase domain protein	100	99	100	93
BMAA1779	conserved hypothetical protein	100	99	100	88
BMAA1780	conserved hypothetical protein	100	99	100	97
BMAA1781	conserved hypothetical protein	100	100	100	99
BMAA1785	chitin binding domain protein	100	100	100	95
BMAA1786	alkyl hydroperoxide reductase, subunit c	100	99	100	98
BMAA1792	sodium:solute symporter family protein	100	99	100	96
BMAA1793	conserved hypothetical protein	100	99	100	89
BMAA1794	acetyl-coenzyme A synthetase	100	100	100	98
BMAA1796	conserved hypothetical protein TIGR00645	100	99	100	98
BMAA1797	hydro-lyase, Fe-S type, tartrate/fumarate family	100	99	100	99
BMAA1798	bacterioferritin	100	100	100	99
BMAA1799	glutamate racemase	100	98	99	88
BMAA1800	bacterioferritin-associated ferredoxin, putative	100	100	100	96
BMAA1801	TonB protein, putative	100	97	100	89
BMAA1802	MotA/TolQ/ExbB proton channel family protein	100	99	100	97
BMAA1803	biopolymer transport protein, ExbD/TolR family	100	100	100	97
BMAA1806	hypothetical protein	100	99	100	93
BMAA1826	TonB-dependent heme/hemoglobin receptor family protein	100	99	100	93
BMAA1827	hemin transport protein HmuS	100	99	100	89
BMAA1828	hemin ABC transporter, periplasmic hemin-binding protein	100	99	100	93
BMAA1829	hemin ABC transporter, permease protein	100	99	100	88
BMAA1830	hemin ABC transporter, ATP-binding protein	100	100	100	88
BMAA1831	membrane protein, putative	100	100	100	94
BMAA1832	penicillin-binding protein, 1A family	100	99	100	91
BMAA1833	chromosome initiation inhibitor IciA, putative	100	99	100	94
BMAA1834	L-allo-threonine aldolase	100	99	100	96
BMAA1835	ubiquinol oxidase family protein	100	100	100	97
BMAA1836	cyanide-insensitive terminal oxidase	100	99	100	97
BMAA1836.1	hypothetical protein	98	100	100	95
BMAA1840	squalene/phytoene synthase family protein	100	98	100	94
BMAA1846	hypothetical protein	100	100	98	93
BMAA1847	lipoprotein, putative	100	99	100	92
BMAA1853	acetyltransferase, GNAT family	100	100	100	93
BMAA1856	MgtC family protein	100	99	100	95
BMAA1857	conserved hypothetical protein	100	99	99	93
BMAA1858	transcriptional regulator, LysR family	100	98	100	91

BMAA1860	methyl-accepting chemotaxis protein	100	99	100	96
BMAA1862	radical SAM domain protein	100	100	100	93
BMAA1867	conserved hypothetical protein	100	100	100	96
BMAA1868	aconitate hydratase	100	99	100	97
BMAA1869	2-methylcitrate synthase/citrate synthase II	100	100	100	98
BMAA1870	methylisocitrate lyase	100	100	100	98
BMAA1871	propionate catabolism operon regulatory protein	100	99	100	94
BMAA1872	methyl-accepting chemotaxis protein	100	99	100	89
BMAA1876	proline iminopeptidase	100	99	100	93
BMAA1889	glyoxalase family protein	100	99	100	86
BMAA1890	O-acetylhomoserine/O-acetylserine sulfhydrylase	100	100	100	95
BMAA1918	transcriptional regulator, LysR family	100	99	100	95
BMAA1919	class III extradiol-type catecholic dioxygenase, putative	100	99	97	89
BMAA1920	quinone oxidoreductase, putative	100	100	100	93
BMAA1921	asparagine synthase (glutamine-hydrolyzing)	100	99	100	95
BMAA1922	pyoverdin chromophore biosynthetic protein PvcC	100	99	100	95
BMAA1923	pyoverdine chromophore biosynthetic protein PvcB	100	97	100	88
BMAA1924	pyoverdine chromophore biosynthetic protein PvcA	100	99	100	88
BMAA1926	alpha-1,2-mannosidase family protein	100	99	100	95
BMAA1927	conserved domain protein	100	99	100	95
BMAA1929	conserved hypothetical protein	100	97	99	90
BMAA1932	transcriptional regulator, LysR family	100	99	100	92
BMAA1933	D-amino acid dehydrogenase, small subunit, putative	100	100	100	92
BMAA1934	glutamate/aspartate ABC transporter, periplasmic glutamate/aspartate-binding protein, putative	100	100	100	95
BMAA1937	fatty acid desaturase domain protein	100	99	100	87
BMAA1939	conserved hypothetical protein	100	100	100	89
BMAA1940	transcriptional regulator, LysR family	100	99	100	97
BMAA1941	hippurate hydolase	100	99	100	96
BMAA1944	ATP-dependent RNA helicase DbpA	100	99	100	95
BMAA1950	transcriptional regulator, IclR family	100	100	100	97
BMAA1951	citrate lyase, beta subunit, putative	100	100	100	94
BMAA1952	outer membrane porin, putative	100	99	100	90
BMAA1953	MmgE/PrpD family protein	100	98	100	91
BMAA1953.1	OpgC protein, putative	100	99	99	88
BMAA1956	rarD protein	100	99	100	91
BMAA1957	acylphosphatase	100	100	100	94
BMAA1958	dihydroflavonol-4-reductase family protein	100	99	100	95
BMAA1960	glycosyl transferase, group 2 family protein	100	98	100	96
BMAA1961	hypothetical protein	100	100	100	87
BMAA1962	hydroxymethylbutenyl pyrophosphate reductase	100	100	100	98
BMAA1963	radical SAM domain protein	100	99	100	96
BMAA1964	conserved hypothetical protein	100	100	100	88
BMAA1966	glutamate-1-semialdehyde 2,1-aminomutase, putative	100	99	100	92
BMAA2000	2-hydroxy-3-oxopropionate reductase	100	99	100	94
BMAA2001	monooxygenase, putative	100	100	100	95
BMAA2003	4'-phosphopantetheinyl transferase family protein	100	100	99	92
BMAA2004	membrane protein, putative	100	100	100	93
BMAA2006	flavin reductase domain protein	100	97	100	87
BMAA2009	hypothetical protein	100	99	100	95

BMAA2010	2-oxoisovalerate dehydrogenase, E3 component, dihydrolipoamide dehydrogenase	100	99	100	94
BMAA2012	2-oxoisovalerate dehydrogenase, E1 component, beta subunit	100	100	100	97
BMAA2013	2-oxoisovalerate dehydrogenase, E1 component, alpha subunit	100	99	100	97
BMAA2017	conserved hypothetical protein	100	100	99	96
BMAA2018	conserved hypothetical protein	100	100	100	89
BMAA2019	hypothetical protein	100	97	97	88
BMAA2020	transcriptional regulator, LysR family	100	100	100	96
BMAA2022	cholesterol oxidase, putative	100	99	100	95
BMAA2023	pyridine nucleotide-disulphide oxidoreductase family protein	100	99	100	90
BMAA2024	C4-type zinc finger protein, DksA/TraR family	100	100	100	96
BMAA2026	intracellular polyhydroxyalkanoate depolymerase	100	99	100	88
BMAA2027	conserved hypothetical protein	100	100	100	90
BMAA2028	conserved hypothetical protein	100	99	100	88
BMAA2030	conserved hypothetical protein, authentic point mutation	100	98	100	93
BMAA2031	sulfate permease family protein	100	99	100	90
BMAA2033	CBS domain protein	100	98	100	90
BMAA2034	conserved hypothetical protein	100	99	100	92
BMAA2035	stress response protein	100	99	99	93
BMAA2055	penicillin-binding protein, 1A family	100	99	100	92
BMAA2061	amidase family protein	100	99	100	88
BMAA2070	sensor histidine kinase	100	99	100	94
BMAA2071	DNA-binding response regulator, LuxR family	100	100	100	95
BMAA2072	ISBma1, transposase	100	99	100	98
BMAA2076	conserved hypothetical protein	100	100	100	97
BMAA2077	conserved hypothetical protein	100	99	100	96
BMAA2078	sensory box/GGDEF/EAL domain protein	100	99	100	97
BMAA2079	hypothetical protein	100	100	100	91
BMAA2080	lipase	100	99	100	89
BMAA2081	4'-phosphopantetheinyl transferase family protein	100	100	99	90
BMAA2082	hypothetical protein	100	99	100	97
BMAA2083	hypothetical protein	100	100	100	94
BMAA2084	conserved hypothetical protein	100	99	100	95
BMAA2085	ABC transporter, permease protein, putative	100	99	100	91
BMAA2086	ABC transporter, ATP-binding protein	100	99	100	96
BMAA2087	monooxygenase, flavin-binding family	100	99	100	94
BMAA2088	cytochrome P450-related protein	100	99	100	92
BMAA2090	acyl transferase domain protein	100	98	100	88
BMAA2092	lipoprotein, putative	100	97	100	87
BMAA2093	conserved hypothetical protein	100	99	100	93
BMAA2094	aminotransferase, class III	100	99	100	95
BMAA2095	conserved hypothetical protein	100	100	100	91
BMAA2096	glutamine ABC transporter, ATP-binding protein	100	100	100	97
BMAA2097	glutamine ABC transporter, permease protein	100	99	100	95
BMAA2098	glutamine ABC transporter, periplasmic glutamine-binding protein	100	99	100	93
BMAA2099	conserved hypothetical protein	100	98	100	92
BMAA2100	squalene-hopene cyclase	100	99	100	96
BMAA2102	phytoene synthase, putative	100	99	100	95
BMAA2105	GGDEF domain protein	100	100	100	97
BMAA2106	conserved hypothetical protein	100	100	100	99

BMAA2107	response regulator	100	99	96	86
BMAA2109	sensor histidine kinase/response regulator	100	99	100	95
BMAA2110	NADPH-dependent FMN reductase domain protein	100	98	100	91
BMAA2111	sigma-70 factor, putative	100	99	100	97
BMAA2113	arsenate reductase	100	99	100	89
BMAA2114	ParA family protein	100	99	100	98
BMAA2115	parB family protein	100	100	100	95
BMAA2117	plasmid replication protein, putative	100	100	100	98

Segment 2- Conserved in Bm and Bp but not as well or absent in Bt

Gene	Description	to Bp % length	% identity
BMA0004	IS407A, transposase OrfA	100	98
BMA0005	IS407A, transposase OrfB	100	99
BMA0012	hypothetical protein	100	100
BMA0013	hypothetical protein	100	100
BMA0014	hypothetical protein	100	99
BMA0015	hypothetical protein	100	99
BMA0021	hypothetical protein	100	100
BMA0022	hypothetical protein	100	100
BMA0025	hypothetical protein	100	98
BMA0026	EAL/GGDEF domain protein	100	99
BMA0031	hypothetical protein	100	98
BMA0033	PAP2 family protein	100	98
BMA0034	hypothetical protein	100	100
BMA0035	hypothetical protein	100	99
BMA0036	hypothetical protein	100	98
BMA0038	conserved hypothetical protein	100	99
BMA0049	hypothetical protein	100	99
BMA0050	hypothetical protein	100	98
BMA0051	ISBma2, transposase	100	100
BMA0053.1	hypothetical protein	100	99
BMA0059	hypothetical protein	100	95
BMA0071	conserved hypothetical protein	100	99
BMA0078	transcriptional regulator, IclR family	100	100
BMA0079	conserved hypothetical protein	100	98
BMA0080	conserved hypothetical protein	100	99
BMA0082	ISBma2, transposase	100	100
BMA0105	conserved hypothetical protein	100	97
BMA0110	IS407A, transposase OrfB	100	99
BMA0111	IS407A, transposase OrfA	100	98
BMA0116	DNA-3-methyladenine glycosylase II	100	99
BMA0120	hypothetical protein	100	97
BMA0122	transcriptional regulator, LysR family	100	99
BMA0123	ABC transporter, periplasmic substrate-binding protein	100	98
BMA0124	histone deacetylase family protein	100	99
BMA0125	hydrolase, carbon-nitrogen family	100	99
BMA0129	hypothetical protein	100	98

BMA0130	conserved hypothetical protein	100	100
BMA0131	IS407A, transposase OrfB	100	99
BMA0132	IS407A, transposase OrfA	100	97
BMA0151	hypothetical protein	100	98
BMA0168	ISBma2, transposase	100	99
BMA0169	hypothetical protein	100	98
BMA0182	hypothetical protein	100	99
BMA0190	thiopurine S-methyltransferase family protein	100	98
BMA0205	hypothetical protein	100	98
BMA0214	hypothetical protein	100	97
BMA0224	hypothetical protein	100	98
BMA0226.1	hypothetical protein	100	100
BMA0232	membrane protein, putative	100	99
BMA0234	asparagine synthase (glutamine-hydrolyzing)	100	99
BMA0235	3-phosphoshikimate-1-carboxyvinyltransferase, putative	100	99
BMA0236	3-phosphoshikimate-1-carboxyvinyltransferase, putative	100	99
BMA0237	conserved hypothetical protein	100	100
BMA0238	hypothetical protein	100	99
BMA0251	hypothetical protein	100	95
BMA0256	conserved hypothetical protein	100	100
BMA0257	conserved domain protein	100	96
BMA0258	hypothetical protein	100	98
BMA0265	IS407A, transposase OrfA	100	98
BMA0266	IS407A, transposase OrfB	100	99
BMA0273	regulatory protein RecX, putative	100	99
BMA0274	conserved hypothetical protein	100	99
BMA0280	hypothetical protein	100	100
BMA0284	IS407A, transposase OrfB	100	99
BMA0285	IS407A, transposase OrfA	100	98
BMA0297	hypothetical protein	100	100
BMA0306	hypothetical protein	96	100
BMA0321	IS407A, transposase OrfB	100	99
BMA0322	IS407A, transposase OrfA	100	98
BMA0339	transcriptional regulator, LysR family	100	100
BMA0340	esterase, putative	100	99
BMA0341	major facilitator family transporter	100	99
BMA0349	hypothetical protein	100	97
BMA0357	conserved domain protein	100	98
BMA0376	conserved hypothetical protein	100	99
BMA0381	ISBma2, transposase	100	100
BMA0384	hypothetical protein	100	98
BMA0385	hypothetical protein	100	99
BMA0388	ISBma2, transposase	100	100
BMA0399	acetyltransferase, GNAT family	100	100
BMA0405	membrane protein, putative	100	99
BMA0417	murein transglycosylase domain protein	100	99
BMA0425	conserved hypothetical protein	96	100
BMA0426	ISBma2, transposase	100	100
BMA0441	IS407A, transposase OrfB	100	99

BMA0442	IS407A, transposase OrfA	100	98
BMA0456	hypothetical protein	100	98
BMA0459	hypothetical protein	100	97
BMA0470.1	hypothetical protein	100	98
BMA0470.2	hypothetical protein	100	99
BMA0470.3	hypothetical protein	100	98
BMA0475	conserved hypothetical protein	100	96
BMA0483	IS407A, transposase OrfB	100	99
BMA0484	IS407A, transposase OrfA	100	98
BMA0485	pseudouridine synthase family protein	100	100
BMA0488	ISBma2, transposase	100	100
BMA0491	hypothetical protein	99	95
BMA0495	IS407A, transposase OrfB	100	99
BMA0496	IS407A, transposase OrfA	100	98
BMA0500	IS407A, transposase OrfB	100	99
BMA0501	IS407A, transposase OrfA	100	98
BMA0504	hypothetical protein	100	100
BMA0505	hypothetical protein	100	95
BMA0520	ribonuclease E	100	99
BMA0535	lipoprotein, putative	100	99
BMA0543	ribonuclease III/hypothetical protein, fusion	100	99
BMA0573	IS407A, transposase OrfB	100	99
BMA0574	IS407A, transposase OrfA	100	98
BMA0579	conserved domain protein, degenerate	100	98
BMA0585	IS407A, transposase OrfA	100	98
BMA0586	IS407A, transposase OrfB	100	99
BMA0597	glutathione S-transferase, putative	100	99
BMA0611	sufD domain protein	100	99
BMA0628	conserved hypothetical protein	100	98
BMA0633.1	hypothetical protein	100	99
BMA0643.1	hypothetical protein	100	98
BMA0648	conserved domain protein	100	100
BMA0658	IS407A, transposase OrfB	100	99
BMA0659	IS407A, transposase OrfA	100	98
BMA0661	conserved domain protein	100	96
BMA0674	hypothetical protein	100	99
BMA0676	ISBma2, transposase	100	99
BMA0690	phosphoglycerate mutase family protein	100	100
BMA0692	hypothetical protein	100	100
BMA0706.1	hypothetical protein	100	96
BMA0720	IS407A, transposase OrfA	100	98
BMA0721	IS407A, transposase OrfB	100	99
BMA0724	lipoprotein, putative	100	99
BMA0728	ISBma2, transposase	100	100
BMA0740	conserved hypothetical protein	100	99
BMA0757	IS407A, transposase OrfB	100	99
BMA0758	IS407A, transposase OrfA	100	98
BMA0767	major facilitator family tranporter	100	99
BMA0790	PAP2 family protein	100	98

BMA0793	gp33, truncation, frameshift	100	97
BMA0794	conserved hypothetical protein	98	95
BMA0805	IS407A, transposase OrfA	100	98
BMA0806	IS407A, transposase OrfB	100	99
BMA0810	transposase ORF B, degenerate	100	95
BMA0812	transcriptional regulator, GntR family	100	99
BMA0813	conserved hypothetical protein	99	99
BMA0815	conserved hypothetical protein	100	96
BMA0816	maltooligosyl trehalose synthase, putative	100	99
BMA0817	4-alpha-glucanotransferase	100	99
BMA0819	glycogen operon protein GlgX, putative	100	99
BMA0821	alpha amylase family protein	100	99
BMA0822	alpha amylase family protein	100	99
BMA0822.1	hypothetical protein	100	95
BMA0831	hypothetical protein	100	99
BMA0832	transcriptional regulator, LysR family	100	98
BMA0833	DNA-binding response regulator	100	100
BMA0835	hypothetical protein	100	97
BMA0836	prophage protein, putative	100	97
BMA0838	DNA-binding response regulator	100	99
BMA0840	haemagluttinin family protein	100	99
BMA0841	ompA family protein	100	99
BMA0842	TPR Domain domain protein	100	99
BMA0843	H-NS histone family protein	100	99
BMA0845	hypothetical protein	100	99
BMA0846	hypothetical protein	100	98
BMA0847	galactose oxidase-related protein	100	99
BMA0850.2	lipoprotein, putative	100	99
BMA0853	conserved hypothetical protein	100	98
BMA0857	IS407A, transposase OrfB	100	99
BMA0858	IS407A, transposase OrfA	100	98
BMA0859	hypothetical protein	100	98
BMA0872	conserved hypothetical protein	100	99
BMA0880	putative trans-aconitate methyltransferase fragment	100	98
BMA0882	drug resistance transporter, EmrB/QacA family	100	99
BMA0883	multidrug resistance protein, putative	100	99
BMA0884	hypothetical protein	100	99
BMA0898	IS407A, transposase OrfA	100	98
BMA0899	IS407A, transposase OrfB	100	99
BMA0902	hypothetical protein	100	99
BMA0908	branched-chain amino acid ABC transporter, permease/ATP binding protein, putative	100	97
BMA0917	SIS domain protein	100	99
BMA0931	methyl-accepting chemotaxis protein, putative	100	99
BMA0938	Ser/Thr protein phosphatase family protein	100	97
BMA0940	conserved hypothetical protein	100	100
BMA0948	IS407A, transposase OrfB	100	99
BMA0949	IS407A, transposase OrfA	100	98
BMA0950	transcriptional regulator, MerR family	100	100
BMA0952	RND efflux system, outer membrane lipoprotein, NodT family	100	99

BMA0959	hypothetical protein	100	98
BMA0971.1	hypothetical protein	100	96
BMA0975	hypothetical protein	100	97
BMA0983	lipoprotein, putative	100	99
BMA0984	hypothetical protein	100	99
BMA0985	hypothetical protein	100	98
BMA0989	hypothetical protein	100	98
BMA0999	methyl-accepting chemotaxis protein, putative	100	99
BMA1002	conserved domain protein	100	98
BMA1007	hypothetical protein	100	97
BMA1011	hypothetical protein	100	97
BMA1012	polyhydroxyalkanoate depolymerase domain protein	100	99
BMA1015	conserved hypothetical protein	100	100
BMA1022	fimbrial chaperone protein	100	99
BMA1023	outer membrane usher protein	100	99
BMA1024	type-1 fimbrial protein, A subunit	100	99
BMA1025	hypothetical protein	100	97
BMA1027.1	hypothetical protein	99	98
BMA1034	DNA-binding response regulator	100	100
BMA1035	IS407A, transposase OrfB, truncation	100	99
BMA1036	ISBma2, transposase, truncation	100	100
BMA1038	penicillin amidase, putative	100	99
BMA1039	carbamoyltransferase family, truncation	100	99
BMA1040	IS407A, transposase OrfA	100	98
BMA1041	IS407A, transposase OrfB	100	99
BMA1042	hypothetical protein	100	97
BMA1066	segregation and condensation protein B	100	96
BMA1077	IS407A, transposase OrfB	100	99
BMA1078	IS407A, transposase OrfA	100	98
BMA1079	ISBma2, transposase, truncation	100	99
BMA1082	IS407A, transposase OrfA	100	98
BMA1083	IS407A, transposase OrfB	100	99
BMA1102	conserved hypothetical protein	100	98
BMA1113	conserved hypothetical protein	100	99
BMA1118	IS407A, transposase OrfA	100	98
BMA1119	IS407A, transposase OrfB	100	99
BMA1120	histidinol-phosphate aminotransferase, putative, authentic point mutation	96	99
BMA1121	jmjC domain protein	100	99
BMA1123	peptide synthetase, putative	100	97
BMA1126	transcriptional regulator, AraC family	100	98
BMA1128.1	hypothetical protein	100	100
BMA1132	hypothetical protein	100	99
BMA1135	drug resistance transporter, EmrB/QacA family	100	99
BMA1141.1	hypothetical protein	100	98
BMA1141.2	hypothetical protein	100	100
BMA1152	transcriptional regulator, MarR family	99	98
BMA1153	amino acid ABC transporter, periplasmic amino acid-binding protein, putative	100	99
BMA1155	major facilitator family transporter	100	99
BMA1156	fenI protein	99	99

BMA1169	magnesium chelatase, putative, authentic frameshift	100	95
BMA1176	cobyrinic acid a,c-diamide synthase	100	98
BMA1180	non-ribosomal peptide synthetase, putative	100	99
BMA1181	non-ribosomal peptide synthetase, putative	100	99
BMA1201	conserved domain protein	100	99
BMA1202	hypothetical protein	100	99
BMA1203	IS407A, transposase OrfB	100	99
BMA1204	IS407A, transposase OrfA	100	98
BMA1214	conserved hypothetical protein	100	97
BMA1216	IS407A, transposase OrfB	100	99
BMA1217	IS407A, transposase OrfA	100	98
BMA1231	IS407A, transposase OrfA	100	98
BMA1232	IS407A, transposase OrfB	100	99
BMA1242.1	hypothetical protein	100	100
BMA1242.2	hypothetical protein	100	98
BMA1256	IS407A, transposase OrfA	100	98
BMA1257	IS407A, transposase OrfB	100	99
BMA1265	ISBma2, transposase	100	99
BMA1268	phosphoesterase family protein	100	100
BMA1278	hypothetical protein	100	96
BMA1279	hypothetical protein	100	97
BMA1284	conserved hypothetical protein	100	96
BMA1286	membrane protein, putative	100	99
BMA1295	IS407A, transposase OrfA	100	98
BMA1296	IS407A, transposase OrfB, authentic point mutation	100	99
BMA1315	conserved hypothetical protein	100	98
BMA1352	hypothetical protein	100	96
BMA1361	DNA polymerase III, subunit gamma, putative	100	99
BMA1375	conserved domain protein	100	99
BMA1397	lipoprotein, NLP/P60 family	100	98
BMA1406	RNA polymerase sigma-70 factor, ECF subfamily	100	98
BMA1409	IS407A, transposase OrfB	100	99
BMA1410	IS407A, transposase OrfA	100	98
BMA1445	acetyltransferase, GNAT family	100	98
BMA1478	IS407A, transposase OrfB	100	99
BMA1479	IS407A, transposase OrfA	100	97
BMA1480	conserved hypothetical protein, degenerate	100	95
BMA1496	conserved hypothetical protein	100	99
BMA1500	ISBma2, transposase	100	100
BMA1503	ISBma2, transposase	100	100
BMA1518	conserved hypothetical protein	100	100
BMA1519	conserved hypothetical protein, authentic point mutation	100	99
BMA1519.1	hypothetical protein	100	98
BMA1521	hypothetical protein	100	95
BMA1523	conserved hypothetical protein	100	97
BMA1573	hypothetical protein	100	99
BMA1585	ISBma2, transposase	100	99
BMA1592	conserved hypothetical protein	100	100
BMA1609.1	hypothetical protein	100	97

BMA1614	ferredoxin--NADP reductase	100	100
BMA1615	molybdenum cofactor biosynthesis protein A, putative	100	98
BMA1616	ferredoxin	100	100
BMA1639	methyltransferase, putative/adenylsulfate kinase	100	98
BMA1665	hypothetical protein	100	98
BMA1697	conserved hypothetical protein	100	99
BMA1715	ISBma2, transposase	100	100
BMA1718	ISBma2, transposase	100	100
BMA1725	hypothetical protein	100	95
BMA1739	ISBma2, transposase	100	100
BMA1742	ISBma2, transposase	100	100
BMA1754	CoA transferase, CAIB/BAIF family	100	97
BMA1758	ISBma2, transposase, truncation	100	99
BMA1759	IS407A, transposase OrfB	100	99
BMA1760	IS407A, transposase OrfA	100	97
BMA1768	exodeoxyribonuclease V, alpha subunit	100	98
BMA1788	conserved hypothetical protein, degenerate	100	96
BMA1795	DNA-binding response regulator	100	99
BMA1800	conserved domain protein	100	100
BMA1802	glutathione S-transferase N-terminal domain protein	100	99
BMA1808	lipoprotein, putative	100	99
BMA1818	NADH dehydrogenase I, L subunit	100	99
BMA1841	IS407A, transposase OrfB	100	99
BMA1842	IS407A, transposase OrfA	100	98
BMA1894	phospholipase C accessory protein, putative	100	98
BMA1894.2	hypothetical protein	100	99
BMA1898	conserved hypothetical protein, authentic frameshift	100	97
BMA1903	hypothetical protein	100	96
BMA1904	conserved domain protein	100	99
BMA1906	conserved domain protein	100	95
BMA1918	hypothetical protein	100	98
BMA1920	conserved hypothetical protein	100	99
BMA1922.1	hypothetical protein	100	97
BMA1932	membrane protein, putative	100	99
BMA1936	ATPase, AAA familyprotein	100	99
BMA1944	conserved hypothetical protein	100	98
BMA1953	IS407A, transposase OrfA	100	98
BMA1954	IS407A, transposase OrfB	100	99
BMA1961.1	hypothetical protein	100	97
BMA1962	transcriptional regulator, LysR family	100	98
BMA1974	IS407A, transposase OrfA	100	98
BMA1975	IS407A, transposase OrfB	100	99
BMA1977	glycosyl transferase family protein	100	100
BMA1978	NAD-dependent epimerase/dehydratase family protein	100	99
BMA1979	glycosyl transferase, putative	100	98
BMA2030	conserved hypothetical protein	100	99
BMA2070	type IV pilus biogenesis protein, putative	100	100
BMA2070.1	hypothetical protein	100	100
BMA2071.1	hypothetical protein	100	100

BMA2073	conserved hypothetical protein	100	98
BMA2086	IS407A, transposase OrfA	100	98
BMA2087	IS407A, transposase OrfB	100	99
BMA2103	hypothetical protein	100	95
BMA2103.1	hypothetical protein	100	96
BMA2103.2	hypothetical protein	100	96
BMA2124	Smr domain protein	100	100
BMA2137	D-cysteine desulfhydrase	100	99
BMA2137.1	hypothetical protein	100	98
BMA2139.1	hypothetical protein	100	99
BMA2157	conserved hypothetical protein	100	98
BMA2164	conserved hypothetical protein	100	99
BMA2165	transcriptional regulator, Sir2 family	100	97
BMA2172	hypothetical protein	100	99
BMA2198	IS407A, transposase OrfA	100	98
BMA2199	IS407A, transposase OrfB	100	99
BMA2201	hypothetical protein	100	100
BMA2201.1	hypothetical protein	100	98
BMA2202	translation initiation factor IF-1	100	100
BMA2203	hydrolase, alpha/beta fold family	100	100
BMA2204	membrane protein, putative	100	99
BMA2206	conserved hypothetical protein	100	100
BMA2214	transcriptional regulator, LysR family	100	99
BMA2216	major facilitator family transporter	100	99
BMA2217	N-carbamyl-L-amino acid amidohydrolase	100	100
BMA2224	IS407A, transposase OrfA	100	100
BMA2225	IS407A, transposase OrfB	100	99
BMA2225.1	hypothetical protein	100	100
BMA2227	ISBma2, transposase	100	100
BMA2247	hypothetical protein	100	97
BMA2249	IS407A, transposase OrfB	100	99
BMA2250	IS407A, transposase OrfA	100	98
BMA2257.1	hypothetical protein	100	98
BMA2258	citrate synthase family protein	100	99
BMA2268	IS407A, transposase OrfB	100	99
BMA2269	IS407A, transposase OrfA	100	98
BMA2270	ISBma2, transposase, truncation	100	100
BMA2278	ISBma2, transposase	100	100
BMA2283	IS407A, transposase OrfB	100	99
BMA2284	IS407A, transposase OrfA	100	98
BMA2288	UDP-3-O-[3-hydroxymyristoyl] N-acetylglucosamine deacetylase	100	99
BMA2289	type I polyketide synthase WcbR	100	99
BMA2290	putative capsular polysaccharide biosynthesis protein WcbQ	100	99
BMA2291	oxidoreductase, short-chain dehydrogenase/reductase family	100	99
BMA2292	capsule polysaccharide biosynthesis/export protein. putative	100	99
BMA2293	D-glycero-D-manno-heptose 1,7-bisphosphate phosphatase	100	99
BMA2294	D-glycero-D-manno-heptose 1-phosphate guanosyltransferase	100	100
BMA2295	phosphoheptose isomerase	100	98
BMA2296	D-glycero-D-manno-heptose 7-phosphate kinase	100	100

BMA2297	GDP-D-mannose dehydratase, putative	100	100
BMA2298	capsular polysaccharide biosynthesis protein, putative	100	100
BMA2299	capsular polysaccharide biosynthesis protein, putative	100	99
BMA2300	glycosyl transferase, group 1 family protein	100	100
BMA2301	capsular polysaccharide biosynthesis protein, putative	100	99
BMA2302	capsular polysaccharide biosynthesis protein, putative	100	99
BMA2303	glycosyl transferase, group 1 family protein	100	99
BMA2304	capsule polysaccharide ABC transporter, ATP-binding protein	100	100
BMA2305	capsular polysaccharide export inner-membrane protein	100	100
BMA2306	capsular polysaccharide export inner-membrane protein	100	100
BMA2307	capsular polysaccharide biosynthesis/export periplasmic protein	100	100
BMA2308	glycosyltransferase, putative	100	98
BMA2309	capsule polysaccharide biosynthesis/export protein, putative	100	99
BMA2310	mannose-1-phosphate guanylyltransferase/mannose-6-phosphate isomerase	100	99
BMA2327	conserved hypothetical protein	100	99
BMA2340	ISBma2, transposase	100	100
BMA2351	lipoprotein, putative	100	98
BMA2362	ISBma2, transposase	100	100
BMA2377	lipoprotein, putative	100	99
BMA2392	hypothetical protein	100	100
BMA2395	transcriptional regulator, putative	99	98
BMA2397	alkylphosphonate utilization operon, ATP-binding protein PhnN	100	99
BMA2398	conserved hypothetical protein	100	96
BMA2399	transcriptional regulator, GntR family	100	99
BMA2400	alkylphosphonate utilization operon protein PhnG	100	100
BMA2401	alkylphosphonate utilization operon protein PhnH	100	100
BMA2402	alkylphosphonate utilization operon protein PhnI	100	98
BMA2403	alkylphosphonate utilization operon protein PhnJ	100	98
BMA2404	phosphonates ABC transporter, ATP-binding protein	100	99
BMA2405	phosphonates ABC transporter, ATP-binding protein	100	99
BMA2406	alkylphosphonate utilization operon protein PhnM	100	100
BMA2407	phosphonates ABC transporter, ATP-binding protein	100	99
BMA2408	phosphonates ABC transporter, periplasmic phosphonates-binding protein, authentic point mutatio	100	99
BMA2409	phosphonates ABC transporter, permease protein	100	99
BMA2418	ISBma2, transposase	100	100
BMA2421	conserved hypothetical protein	99	96
BMA2425	conserved hypothetical protein	100	100
BMA2427	heat shock protein, Hsp20 family	100	98
BMA2428	heat shock protein, Hsp20 family	100	100
BMA2429	hypothetical protein	100	98
BMA2431	chaperonin, 10 kDa	100	100
BMA2432	hypothetical protein	100	98
BMA2433	hypothetical protein	100	100
BMA2454	biopolymer transport protein, ExbD/TolR family	100	95
BMA2470	spermidine synthase, putative	100	100
BMA2473	conserved domain protein	100	99
BMA2504	conserved hypothetical protein	100	97
BMA2527	IS407A, transposase OrfB	100	99
BMA2528	IS407A, transposase OrfB	100	99

BMA2529	IS407A, transposase OrfA	100	97
BMA2544	AhpC/TSA family protein	100	98
BMA2563	ISBma2, transposase	100	100
BMA2564	IS407A, transposase OrfB	100	99
BMA2565	IS407A, transposase OrfA	100	97
BMA2576	phenylacetic acid degradation protein PaaD	100	99
BMA2592	lipoprotein, putative	100	99
BMA2595	IS407A, transposase OrfB	100	99
BMA2596	IS407A, transposase OrfA	100	98
BMA2654	IS407A, transposase OrfA	100	97
BMA2655	IS407A, transposase OrfB	100	99
BMA2672	hypothetical protein	100	97
BMA2681	conserved hypothetical protein	100	98
BMA2682	conserved hypothetical protein	100	100
BMA2687	IS407A, transposase OrfB	100	99
BMA2688	IS407A, transposase OrfA	100	98
BMA2689	conserved hypothetical protein	100	98
BMA2690	conserved hypothetical protein	100	100
BMA2694	stringent starvation protein B	100	98
BMA2720	hypothetical protein	100	98
BMA2748	type II/III secretion system protein	100	99
BMA2749	hypothetical protein	100	99
BMA2750	fimbrial assembly protein PilN, putative	100	99
BMA2753	ISBma2, transposase	100	100
BMA2759	IS407A, transposase OrfB	100	99
BMA2760	IS407A, transposase OrfA	100	98
BMA2772	hypothetical protein	100	95
BMA2790	ISBma2, transposase	100	100
BMA2792	IS407A, transposase OrfA	100	98
BMA2793	IS407A, transposase OrfB	100	99
BMA2798	cytochrome c family protein	100	100
BMA2803	ISBma2, transposase	100	100
BMA2813	acyltransferase family protein	100	99
BMA2818.1	hypothetical protein	100	100
BMA2818.2	hypothetical protein	100	100
BMA2820	secretion protein, putative	100	99
BMA2822.1	hypothetical protein	100	96
BMA2834	IS407A, transposase OrfB	100	99
BMA2835	IS407A, transposase OrfA	100	98
BMA2853	hypothetical protein	100	98
BMA2856	chemotaxis protein methyltransferase CheR	100	99
BMA2876	TPR domain protein	100	99
BMA2877	aminotransferase, DegT/DnrJ/EryC1/StrS family	100	100
BMA2878	3-oxoacyl-(acyl-carrier-protein) synthase III, putative	100	99
BMA2879	hypothetical protein	100	98
BMA2880	3-oxoacyl-(acyl-carrier-protein) synthase III, putative	100	99
BMA2881	oxidoreductase, short chain dehydrogenase/reductase family	100	100
BMA2882	hexapeptide transferase family protein	100	100
BMA2883	iron-sulfur cluster-binding protein, Rieske family	100	100

BMA2884	hypothetical protein	100	97
BMA2885	hypothetical protein	100	96
BMA2892	hypothetical protein	100	96
BMA2898	IS407A, transposase OrfA	100	98
BMA2899	IS407A, transposase OrfB	100	99
BMA2912	ISBma2, transposase	100	100
BMA2925	conserved hypothetical protein	100	100
BMA2939	conserved hypothetical protein	100	99
BMA2960	conserved hypothetical protein, authentic frameshift	100	96
BMA2964	hypothetical protein	100	100
BMA2969	transcriptional regulator, TetR family	100	99
BMA2973	oxidoreductase, FAD-binding, degenerate	100	96
BMA2979	acyltransferase family protein	100	99
BMA2982	ISBma2, transposase	100	100
BMA2985	hypothetical protein	100	96
BMA2990	conserved hypothetical protein	97	95
BMA2999	hypothetical protein	100	100
BMA3000	conserved hypothetical protein	100	99
BMA3001	membrane protein, putative	100	99
BMA3006	IS407A, transposase OrfB	100	99
BMA3007	IS407A, transposase OrfA	100	98
BMA3019	lipoprotein, putative	100	98
BMA3024	conserved hypothetical protein	100	98
BMA3025	IS407A, transposase OrfA	100	98
BMA3026	IS407A, transposase OrfB	100	99
BMA3033	mdcG protein	100	99
BMA3045	conserved hypothetical protein	100	100
BMA3050	transcriptional regulator, GntR family	100	99
BMA3052	nitrite/sulfite reductase family protein	100	99
BMA3053	conserved hypothetical protein	100	100
BMA3057	conserved hypothetical protein	100	100
BMA3058	carotenoid 9,10-9',10' cleavage dioxygenase, putative	100	100
BMA3059	glucosamine--fructose-6-phosphate aminotransferase, isomerizing	100	99
BMA3060	transcriptional regulator, AsnC family	100	97
BMA3061	transcriptional regulator, GntR family	100	99
BMA3067	hypothetical protein	100	98
BMA3068	hypothetical protein	100	99
BMA3077	Ser/Thr protein phosphatase family protein	100	95
BMA3079	conserved hypothetical protein	100	100
BMA3080	membrane protein, putative	100	100
BMA3086	conserved domain protein	100	95
BMA3087	conserved hypothetical protein	100	99
BMA3088	conserved hypothetical protein	100	95
BMA3091	IS407A, transposase OrfB	100	99
BMA3092	IS407A, transposase OrfA	100	98
BMA3134	acetyltransferase, GNAT family	100	98
BMA3139	ISBma2, transposase	100	99
BMA3146	conserved hypothetical protein	100	100
BMA3147	major facilitator family transporter	100	99

BMA3148	fatty acid desaturase family protein	100	99
BMA3149	diaminobutyrate--2-oxoglutarate aminotransferase	100	100
BMA3150	hypothetical protein	100	98
BMA3151	ABC transporter, permease protein, putative	100	99
BMA3152	ABC transporter, ATP-binding protein	100	100
BMA3153	syringomycin synthesis regulator SyrP, putative	100	99
BMA3154	conserved hypothetical protein	100	100
BMA3155	citrate synthase-related protein	100	99
BMA3156	acyl-CoA dehydrogenase domain protein	100	99
BMA3157	hypothetical protein	100	99
BMA3158	AMP-binding domain protein	100	99
BMA3159	pyridoxal-dependent decarboxylase family protein	100	99
BMA3160	hypothetical protein	100	99
BMA3161	conserved hypothetical protein	100	100
BMA3162	hypothetical protein	100	99
BMA3163	hypothetical protein	100	100
BMA3164	hypothetical protein	100	100
BMA3164.1	hypothetical protein	100	98
BMA3166	AMP-binding domain protein	100	99
BMA3167	transcriptional regulator, LysR family	100	100
BMA3173.2	hypothetical protein	100	96
BMA3182	ISBma2, transposase	100	100
BMA3216	ISBma2, transposase	100	99
BMA3222	acetyltransferase, GNAT family	100	98
BMA3224	conserved hypothetical protein	100	98
BMA3236	hypothetical protein	100	99
BMA3239	ISBma2, transposase	100	100
BMA3249.1	hypothetical protein	100	100
BMA3265	hypothetical protein	100	97
BMA3276	flagellar hook-length control protein	100	98
BMA3285	hypothetical protein	100	96
BMA3291	ISBma2, transposase	100	100
BMA3293	threonine efflux protein, putative	100	99
BMA3295	hypothetical protein	100	98
BMA3295.1	hypothetical protein	100	100
BMA3319	RebB protein, putative	100	100
BMA3344	conserved hypothetical protein, degenerate	100	95
BMA3351	IS407A, transposase OrfB	100	99
BMA3352	IS407A, transposase OrfA	100	100
BMA3354.1	hypothetical protein	100	95
BMA3354.2	hypothetical protein	100	98
BMA3361	ISBma2, transposase	100	100
BMA3363.1	hypothetical protein	100	96
BMA3366	heavy-metal-associated domain protein	100	100
BMA3374	outer membrane efflux protein	100	99
BMA3375	efflux transporter, RND family, MFP subunit	100	99
BMA3376	heavy metal efflux pump, CzcA family	100	99
BMA3377	conserved hypothetical protein	100	100
BMA3378	streptavidin,putative	100	100

BMA3383	conserved domain protein	100	98
BMA3385.1	hypothetical protein	99	100
BMA3390.1	hypothetical protein	100	100
BMA3393	IS407A, transposase OrfA	100	98
BMA3394	IS407A, transposase OrfB	100	99
BMAA0020	hypothetical protein	100	98
BMAA0023	cytochrome P450-related protein	100	100
BMAA0026	hypothetical protein	100	99
BMAA0028	hypothetical protein	100	99
BMAA0033	hypothetical protein	100	99
BMAA0036	gluconolactonase, putative	100	100
BMAA0037	hypothetical protein	100	97
BMAA0053	membrane protein, putative	100	100
BMAA0054	conserved domain protein	100	100
BMAA0057	hypothetical protein	100	95
BMAA0059	conserved hypothetical protein	100	98
BMAA0060	conserved hypothetical protein	100	98
BMAA0061	RNA polymerase sigma-70 factor, ECF subfamily	100	99
BMAA0064	conserved hypothetical protein	100	97
BMAA0065	hypothetical protein	98	98
BMAA0066	hypothetical protein	100	97
BMAA0067	hypothetical protein	100	97
BMAA0068	H-NS histone family protein	100	100
BMAA0069	alpha-ketoglutarate permease	100	99
BMAA0071	C4-dicarboxylate transport transcriptional regulatory protein	100	99
BMAA0072	hypothetical protein	100	98
BMAA0073	hypothetical protein	100	97
BMAA0077	transcriptional regulator, AraC family	100	97
BMAA0078	hypothetical protein	100	98
BMAA0080	hypothetical protein	100	98
BMAA0082	IS407A, transposase OrfA	100	97
BMAA0083	IS407A, transposase OrfB	100	99
BMAA0084	ISBma2, transposase, truncation	100	98
BMAA0088	hypothetical protein	100	98
BMAA0090	lipoprotein, putative	100	96
BMAA0091	hypothetical protein	100	98
BMAA0092	hypothetical protein	100	98
BMAA0094	hypothetical protein	100	100
BMAA0095	hypothetical protein	100	98
BMAA0098	hypothetical protein	100	96
BMAA0103	hypothetical protein	98	96
BMAA0105	IS407A, transposase OrfB	100	99
BMAA0106	IS407A, transposase OrfA	100	98
BMAA0108	conserved hypothetical protein	100	95
BMAA0111	hypothetical protein	100	97
BMAA0112	hypothetical protein	100	98
BMAA0113	hypothetical protein	100	99
BMAA0124.1	ATP synthase gene 1, putative	100	97
BMAA0130	ATP synthase F1, alpha subunit	100	98

BMAA0133	hypothetical protein	100	99
BMAA0135	methyl-accepting chemotaxis protein, putative	100	99
BMAA0137	sensory box histidine kinase/response regulator	100	99
BMAA0139	RND efflux system, cytoplasmic membrane extrusion protein	100	98
BMAA0142	transcriptional regulator, TetR family	100	99
BMAA0143	hypothetical protein	100	98
BMAA0153	adenylylsulfate kinase	100	98
BMAA0156	hypothetical protein	100	96
BMAA0159	hypothetical protein	100	98
BMAA0162	hypothetical protein	100	100
BMAA0165	hypothetical protein	100	98
BMAA0170	ISBma2, transposase	100	99
BMAA0171	hypothetical protein	100	100
BMAA0174	IS407A, transposase OrfB	100	99
BMAA0175	IS407A, transposase OrfA	100	97
BMAA0177	BNR/Asp-box repeat protein	100	99
BMAA0179	membrane protein, putative	100	99
BMAA0182	hypothetical protein	100	100
BMAA0189	conserved hypothetical protein	100	99
BMAA0190	transcriptional regulator, LysR family, authentic point mutation	100	99
BMAA0193	hypothetical protein	100	97
BMAA0214	conserved hypothetical protein	100	97
BMAA0215	hypothetical protein	100	99
BMAA0219	CheR methyltransferase, SAM binding/TPR domain protein	100	99
BMAA0221	chemotaxis sensor histidine kinase, putative	100	99
BMAA0234	hypothetical protein	100	98
BMAA0235	dyp-type peroxidase family protein	100	98
BMAA0237	hypothetical protein	100	100
BMAA0238	hypothetical protein	100	98
BMAA0254	hypothetical protein	100	96
BMAA0255	lipoprotein, putative	100	100
BMAA0258	hypothetical protein	100	95
BMAA0262	conserved hypothetical protein	100	97
BMAA0267	hypothetical protein	100	97
BMAA0273	hypothetical protein	100	95
BMAA0275	IS407A, transposase OrfA	100	98
BMAA0276	IS407A, transposase OrfB	100	99
BMAA0278	hypothetical protein	100	97
BMAA0282	conserved hypothetical protein	100	98
BMAA0293	hypothetical protein	100	98
BMAA0296	hypothetical protein	100	98
BMAA0305	hypothetical protein	100	97
BMAA0312	hypothetical protein	100	100
BMAA0313	conserved hypothetical protein, point mutation	100	99
BMAA0314	hypothetical protein	100	98
BMAA0316	conserved hypothetical protein	100	99
BMAA0317	membrane protein, putative	100	98
BMAA0322	hypothetical protein	100	96
BMAA0332	ISBma2, transposase	100	99

BMAA0341	IS407A, transposase OrfA, truncation	100	100
BMAA0342	IS407A, transposase OrfB	100	99
BMAA0344	hypothetical protein	100	100
BMAA0346	lipoprotein, putative	100	98
BMAA0347	hypothetical protein	100	100
BMAA0357	hypothetical protein	100	95
BMAA0359	hypothetical protein	100	97
BMAA0361	hypothetical protein	100	98
BMAA0364	conserved hypothetical protein	100	95
BMAA0366	hypothetical protein	100	98
BMAA0371	hypothetical protein	100	97
BMAA0372	hypothetical protein	100	100
BMAA0374	L-lactate dehydrogenase	100	100
BMAA0375	transcriptional regulator, LysR family	100	99
BMAA0377	hypothetical protein	100	99
BMAA0378	hypothetical protein	100	97
BMAA0379	cysteine desulfurase	100	99
BMAA0380	major facilitator family transporter	100	100
BMAA0381	metallo-beta-lactamase family protein	100	99
BMAA0382	transcriptional regulator, ArsR family	100	99
BMAA0383	hypothetical protein	100	97
BMAA0384	conserved hypothetical protein	100	98
BMAA0389	hypothetical protein	100	97
BMAA0390	hypothetical protein	100	98
BMAA0393	FHA domain protein	100	98
BMAA0400.1	serine/threonine protein kinase	100	98
BMAA0408	conserved hypothetical protein	100	97
BMAA0413	IS407A, transposase OrfB	100	99
BMAA0414	IS407A, transposase OrfA	100	98
BMAA0426	hypothetical protein	100	100
BMAA0436	IS407A, transposase OrfB	100	99
BMAA0437	IS407A, transposase OrfA	100	98
BMAA0456	hypothetical protein	100	98
BMAA0458	hypothetical protein	100	98
BMAA0465	levanase	100	99
BMAA0470	hypothetical protein	100	98
BMAA0497	IS407A, transposase OrfB	100	99
BMAA0498	IS407A, transposase OrfA	100	98
BMAA0506	hypothetical protein	97	96
BMAA0511	hypothetical protein	100	100
BMAA0512	glycerophosphoryl diester phosphodiesterase family protein	100	99
BMAA0513	conserved hypothetical protein, authentic frameshift	100	95
BMAA0514	hypothetical protein	100	97
BMAA0516	transcriptional regulator, putative	100	100
BMAA0517	NADH-quinone oxidoreductase, B subunit	100	100
BMAA0518	hypothetical protein	100	95
BMAA0520	hypothetical protein	100	98
BMAA0522	hypothetical protein	100	100
BMAA0523	lipoprotein, putative	100	97

BMAA0537	hypothetical protein	99	100
BMAA0542	beta-ketoadipyl CoA thiolase	100	99
BMAA0548	hypothetical protein	100	97
BMAA0551	IS407A, transposase OrfA	100	98
BMAA0552	IS407A, transposase OrfB	100	99
BMAA0553	Ser/Thr protein phosphatase family protein	100	99
BMAA0555	IS407A, transposase OrfB	100	99
BMAA0556	IS407A, transposase OrfA	100	98
BMAA0559	conserved hypothetical protein	100	100
BMAA0563	NADPH-dependent FMN reductase family protein	100	98
BMAA0568	conserved hypothetical protein	100	100
BMAA0568.1	hypothetical protein	100	98
BMAA0580	conserved hypothetical protein	100	99
BMAA0581	hypothetical protein	100	97
BMAA0582	conserved domain protein	100	99
BMAA0583	IS407A, transposase OrfA	100	98
BMAA0584	IS407A, transposase OrfB	100	99
BMAA0585	secretory lipase family protein	99	99
BMAA0586	hypothetical protein	100	100
BMAA0587	outer membrane porin, putative	100	100
BMAA0588	hypothetical protein	100	95
BMAA0594	hypothetical protein	100	98
BMAA0598	hypothetical protein	100	98
BMAA0600	glyoxalase family protein	100	99
BMAA0604	membrane protein	100	100
BMAA0605	conserved hypothetical protein	100	99
BMAA0606	oxidoreductase, short-chain dehydrogenase/reductase family	100	99
BMAA0607	transcriptional regulator, GntR family	100	99
BMAA0608	voltage gated chloride channel family protein	100	99
BMAA0609	hypothetical protein	100	99
BMAA0613	hypothetical protein	100	96
BMAA0614	conserved domain protein	100	98
BMAA0616	hypothetical protein	100	98
BMAA0617	hypothetical protein	100	96
BMAA0618	hypothetical protein	100	100
BMAA0619	transcriptional regulator, MarR family	100	100
BMAA0621	arabinose ABC transporter, permease protein, putative, authentic frameshift	100	98
BMAA0625	conserved hypothetical protein	100	100
BMAA0626	hypothetical protein	99	99
BMAA0628	hypothetical protein	100	97
BMAA0629	p-hydroxycinnamoyl CoA hydratase/lyase	100	99
BMAA0630	aldehyde dehydrogenase (NADP) family protein	100	100
BMAA0632	3-hydroxyphenylpropionic acid transporter	100	100
BMAA0633	outer membrane porin, putative	100	98
BMAA0634	chlorogenate esterase	100	99
BMAA0640	D-galactarate dehydratase/altronate dehydratase, putative, authentic frameshift	100	99
BMAA0646	hypothetical protein	100	97
BMAA0647	IS407A, transposase OrfB	100	99
BMAA0648	IS407A, transposase OrfA	100	98

BMAA0651	H-NS histone family protein	100	99
BMAA0652	transcriptional regulator, IclR family	100	99
BMAA0653	hypothetical protein	100	99
BMAA0654	EAL domain protein	100	99
BMAA0655	hypothetical protein	100	100
BMAA0656	hypothetical protein	100	97
BMAA0657	hypothetical protein	100	100
BMAA0661	hypothetical protein	100	96
BMAA0663	hypothetical protein	99	96
BMAA0664	EAL/GGDEF domain protein	100	99
BMAA0669	hypothetical protein	100	98
BMAA0670	hypothetical protein	100	98
BMAA0671	acetyltransferase, GNAT family	100	97
BMAA0673.1	hypothetical protein	100	97
BMAA0673.2	hypothetical protein	100	98
BMAA0676	hypothetical protein	100	96
BMAA0680	penicillin-binding protein	100	97
BMAA0684	hypothetical protein	100	96
BMAA0685	hypothetical protein	100	100
BMAA0687	conserved domain protein	100	99
BMAA0688	hypothetical protein	100	100
BMAA0689	hypothetical protein	100	100
BMAA0690	dioxygenase, TauD/TfdA family	100	99
BMAA0691	conserved domain protein	100	100
BMAA0692	isopenicillin N epimerase, putative	100	100
BMAA0697	hypothetical protein	100	96
BMAA0699.1	hypothetical protein	100	98
BMAA0703	DNA-binding response regulator, LuxR family	100	100
BMAA0704	hypothetical protein	100	100
BMAA0715	conserved hypothetical protein	100	99
BMAA0718	hypothetical protein	100	97
BMAA0721	acyl-CoA dehydrogenase domain protein	100	99
BMAA0722	conserved hypothetical protein	100	99
BMAA0724	IS407A, transposase OrfB	100	99
BMAA0725	IS407A, transposase OrfA	100	98
BMAA0726	transposase, degenerate	100	98
BMAA0727	GTP cyclohydrolase I	100	99
BMAA0728	hypothetical protein	100	97
BMAA0729	hypothetical protein	99	98
BMAA0733	conserved hypothetical protein	100	99
BMAA0735	conserved hypothetical protein	100	98
BMAA0736	pentapeptide repeat family protein, authentic frameshift	100	98
BMAA0738	ATP-dependent Clp protease, ATP-binding subunit ClpB	100	98
BMAA0741	conserved hypothetical protein	100	99
BMAA0742	conserved hypothetical protein	100	99
BMAA0747	conserved hypothetical protein	100	95
BMAA0748	hypothetical protein	100	98
BMAA0751	N-acetylmuramoyl-L-alanine amidase domain protein	100	100
BMAA0752	hypothetical protein	100	97

BMAA0761	hypothetical protein	100	95
BMAA0764	IS1356, transposase, degenerate	100	95
BMAA0765	oxidoreductase, zinc-binding dehydrogenase family	100	100
BMAA0766	starvation sensing protein RspA	100	99
BMAA0767	transcriptional regulator, GntRfamily	100	99
BMAA0768	mannitol dehydrogenase family protein	100	99
BMAA0769	sugar transporter, putative	100	96
BMAA0780	ApbE family protein	100	97
BMAA0786	hypothetical protein	100	98
BMAA0791	hypothetical protein	100	99
BMAA0792	conserved domain protein	100	100
BMAA0793	jmjC domain protein	100	98
BMAA0794	conserved hypothetical protein	100	99
BMAA0795	iron-sulfur cluster-binding protein, rieske family/carboxynorspermidine decarboxylase	100	98
BMAA0796	hypothetical protein	100	97
BMAA0797	hypothetical protein	100	96
BMAA0798	mulitcopper oxidase domain protein	100	99
BMAA0800	conserved domain protein, authentic frameshift	100	95
BMAA0811	IS407A, transposase OrfB	100	99
BMAA0812	IS407A, transposase OrfA	100	98
BMAA0821	HD domain protein	100	99
BMAA0823	hypothetical protein	100	100
BMAA0824	conserved hypothetical protein, authentic frameshift	97	95
BMAA0832	hypothetical protein	100	99
BMAA0837	conserved hypothetical protein	100	99
BMAA0840	hypothetical protein	100	99
BMAA0841	conserved hypothetical protein, degenerate	98	97
BMAA0842	hypothetical protein	100	96
BMAA0845	IS407A, transposase OrfB	100	99
BMAA0846	IS407A, transposase OrfA	100	98
BMAA0853	hypothetical protein	100	96
BMAA0866	hypothetical protein	100	97
BMAA0869	IS407A, transposase OrfB	100	99
BMAA0870	IS407A, transposase OrfA	100	98
BMAA0872	ISBma2, transposase	100	99
BMAA0873	hypothetical protein	100	99
BMAA0878	ISBma2, transposase	100	99
BMAA0886	IS407A, transposase OrfA	100	98
BMAA0887	IS407A, transposase OrfB	100	99
BMAA0890	IS407A, transposase OrfB	100	99
BMAA0891	IS407A, transposase OrfA	100	98
BMAA0897	hypothetical protein	100	99
BMAA0898	hypothetical protein	100	100
BMAA0900	hypothetical protein	100	100
BMAA0900.1	hypothetical protein	100	100
BMAA0904	lipoprotein, putative	100	95
BMAA0905	hypothetical protein	100	99
BMAA0906	glyoxylate reductase	100	100
BMAA0911	major facilitator family transporter	100	99

BMAA0913	hypothetical protein	100	98
BMAA0917	IS407A, transposase OrfA	100	98
BMAA0918	IS407A, transposase OrfB	100	99
BMAA0920	hypothetical protein	100	97
BMAA0929	rhodanese-like domain protein	100	99
BMAA0930	serine O-acetyltransferase, putative	100	99
BMAA0931	DNA-binding protein	100	100
BMAA0932	conserved hypothetical protein	100	100
BMAA0933	cysteine desulfurase, putative	100	99
BMAA0935	hypothetical protein	100	98
BMAA0936	hypothetical protein	100	99
BMAA0945	di-haem cytochrome c peroxidase family protein	100	99
BMAA0948	major facilitator family transporter	100	99
BMAA0953	hypothetical protein	100	99
BMAA0954	transcriptional regulator, AraC family	100	99
BMAA0955	hypothetical protein	100	95
BMAA0957	conserved hypothetical protein	100	100
BMAA0958	conserved hypothetical protein	100	96
BMAA0959	glycolate oxidase, subunit GlcD, putative	100	99
BMAA0961	alanyl-tRNA synthetase-related protein	100	99
BMAA0962	membrane protein, putative	100	99
BMAA0963	isoleucyl-tRNA synthetase	100	99
BMAA0964	hypothetical protein	100	96
BMAA0969	hypothetical protein	100	100
BMAA0983	hypothetical protein	100	99
BMAA0991	IS407A, transposase OrfA	100	98
BMAA0992	IS407A, transposase OrfB	100	99
BMAA0993	ATP-dependent helicase, DEAD/DEAH family	100	99
BMAA0994	hypothetical protein	100	99
BMAA0999	hypothetical protein	100	97
BMAA1004	IS407A, transposase OrfA	100	98
BMAA1005	IS407A, transposase OrfB	100	99
BMAA1007	transcriptional regulator, TetR family	100	100
BMAA1008	protoporphyrinogen oxidase, putative	100	100
BMAA1009	membrane protein	100	98
BMAA1011	hypothetical protein	100	100
BMAA1013	RNA polymerase sigma-70 factor, ECF subfamily	100	100
BMAA1014	hypothetical protein	100	98
BMAA1016	AMP-binding domain protein	100	99
BMAA1017	acyl-CoA dehydrogenase domain protein	100	99
BMAA1018	acyl-CoA dehydrogenase domain protein	100	98
BMAA1019	phosphopantetheine attachment site domain protein	100	98
BMAA1020	conserved hypothetical protein	100	99
BMAA1021	thiotemplate mechanism natural product synthetase	99	98
BMAA1022	polyketide synthase, putative	100	99
BMAA1023	IS407A, transposase OrfB	100	99
BMAA1024	IS407A, transposase OrfA	100	98
BMAA1025	hypothetical protein	100	96
BMAA1026	hypothetical protein	100	97

BMAA1031	heavy metal resistance protein CzcC	100	99
BMAA1032	hypothetical protein	100	100
BMAA1033	hypothetical protein	100	100
BMAA1034	hypothetical protein	100	99
BMAA1041	hypothetical protein	100	98
BMAA1047	hypothetical protein	100	97
BMAA1054	IS407A, transposase OrfB	100	99
BMAA1055	IS407A, transposase OrfA	100	98
BMAA1061	hypothetical protein	100	98
BMAA1065	conserved hypothetical protein, degenerate	100	96
BMAA1066	conserved hypothetical protein	100	100
BMAA1067	transporter, CorA family	100	100
BMAA1068	hypothetical protein	100	100
BMAA1071	conserved hypothetical protein	100	100
BMAA1072	hypothetical protein	100	100
BMAA1080	hypothetical protein	100	96
BMAA1081	D-alanyl-D-alanine carboxypeptidase family protein	100	99
BMAA1082	penicillin-binding protein	100	99
BMAA1088	hypothetical protein	99	99
BMAA1092	GTP cyclohydrolase I	100	98
BMAA1093.1	hypothetical protein	100	100
BMAA1095	hypothetical protein	100	99
BMAA1097	hypothetical protein	100	95
BMAA1105	CoA transferase, CAIB/BAIF family	100	99
BMAA1106	oxidoreductase, zinc-binding dehydrogenase family protein	100	100
BMAA1107	alcohol dehydrogenase, iron-containing	100	100
BMAA1110	hypothetical protein	100	98
BMAA1111	hypothetical protein	100	99
BMAA1113	conserved hypothetical protein	100	98
BMAA1114	conserved hypothetical protein	100	98
BMAA1117	nonribosomal peptide synthetase DhbF	100	99
BMAA1118	mbtH-like protein	100	100
BMAA1119	multidrug resistance protein	100	100
BMAA1120	non-ribosomal peptide synthase, putative	100	99
BMAA1121	ISBma2, transposase	100	99
BMAA1132	oxidoreductase, zinc-binding dehydrogenase family	100	99
BMAA1133	transcriptional regulator, AraC family	100	98
BMAA1147	acyl-CoA dehydrogenase domain protein	100	99
BMAA1149	hypothetical protein	100	98
BMAA1151	conserved hypothetical protein	100	100
BMAA1157	hypothetical protein	100	100
BMAA1160	hypothetical protein	100	97
BMAA1162	hypothetical protein	98	96
BMAA1163	cytochrome c oxidase, subunit III family protein	100	100
BMAA1164	membrane protein, putative	100	98
BMAA1165	hypothetical protein	100	98
BMAA1167	hypothetical protein	100	100
BMAA1172	hypothetical protein	100	100
BMAA1174	IS407A, transposase OrfA	100	98

BMAA1175	IS407A, transposase OrfB	100	99
BMAA1178	major facilitator family transporter, truncation	100	99
BMAA1179	branched-chain amino acid ABC transporter, permease protein, putative	100	99
BMAA1183	hypothetical protein	100	95
BMAA1184	conserved hypothetical protein	100	98
BMAA1185	YceI like family protein	100	98
BMAA1186	conserved hypothetical protein	100	98
BMAA1188	lipoprotein, putative	100	100
BMAA1192	hypothetical protein	100	98
BMAA1200	drug resistance transporter, EmrB/QacA family	100	98
BMAA1201	halogenase PrnC	100	99
BMAA1202	polyketide synthase, putative, degenerate	100	98
BMAA1208	sodium/hydrogen exchanger	100	99
BMAA1209	malonyl CoA-acyl carrier protein transacylase	100	99
BMAA1211	hypothetical protein	100	99
BMAA1212	polyketide biosynthesis protein, interruption-N	96	99
BMAA1214	polyketide biosynthesis protein, interruption-C	100	97
BMAA1215	polyketide biosynthesis enoyl-CoA hydratase	100	100
BMAA1216	polyketide biosynthesis enoyl-CoA hydratase	100	99
BMAA1217	acyl carier protein	100	100
BMAA1218	polyketide beta-ketoacyl synthase, putative	100	99
BMAA1220	iron-sulfur cluster-binding protein, rieske family	100	100
BMAA1221	transcriptional regulator, AraC family	100	100
BMAA1222	conserved hypothetical protein	100	99
BMAA1223	catalase	100	100
BMAA1224	hypothetical protein	100	97
BMAA1226	IS407A, transposase OrfA	100	98
BMAA1227	IS407A, transposase OrfB	100	99
BMAA1230	transporter, putative	100	97
BMAA1231	hypothetical protein	100	97
BMAA1234	hypothetical protein	100	100
BMAA1235	conserved domain protein	100	99
BMAA1248.1	hypothetical protein	100	98
BMAA1252	hypothetical protein	100	99
BMAA1256	conserved hypothetical protein	100	100
BMAA1264	hypothetical protein	100	96
BMAA1266	YD repeat protein	100	99
BMAA1269.1	hypothetical protein	100	98
BMAA1271	hypothetical protein	100	99
BMAA1280	hypothetical protein	100	100
BMAA1282	hypothetical protein	100	98
BMAA1284	lipoprotein NlpD, putative	100	99
BMAA1287	hypothetical protein	100	96
BMAA1296	hypothetical protein	100	98
BMAA1301	phosphinothricin N-acetyltransferase	100	98
BMAA1304	hypothetical protein	100	100
BMAA1307	IS407A, transposase OrfA	100	98
BMAA1308	IS407A, transposase OrfB	100	99
BMAA1309	transposase, degenerate	100	98

BMAA1312	conserved hypothetical protein	100	97
BMAA1314	hypothetical protein	100	99
BMAA1315	hypothetical protein	100	97
BMAA1316	hypothetical protein	100	99
BMAA1318	hypothetical protein	100	95
BMAA1321	hypothetical protein	100	96
BMAA1357	acetyltransferase, GNAT family	100	97
BMAA1358	LysE family protein	100	100
BMAA1359	hypothetical protein	97	95
BMAA1362	hypothetical protein	100	97
BMAA1371	conserved domain protein, authentic frameshift	100	97
BMAA1377	IS407A, transposase OrfA	100	98
BMAA1378	IS407A, transposase OrfB	100	99
BMAA1386	conserved hypothetical protein	100	98
BMAA1387	hypothetical protein	100	99
BMAA1390	conserved hypothetical protein	100	99
BMAA1391	cellulose synthase, putative	100	99
BMAA1392	RND efflux system, outer membrane lipoprotein, NodT family	100	99
BMAA1395	cyclic nucleotide-binding domain protein	100	96
BMAA1396	sensor histidine kinase, authentic frameshift	100	98
BMAA1407	hypothetical protein	100	98
BMAA1409	hypothetical protein	100	99
BMAA1410	membrane protein, putative	100	99
BMAA1411	IS407A, transposase OrfA	100	98
BMAA1412	IS407A, transposase OrfB	100	99
BMAA1425	methylenomycin A resistance protein, putative	100	96
BMAA1433	hypothetical protein	100	100
BMAA1434	hypothetical protein	100	99
BMAA1436.1	hypothetical protein	100	95
BMAA1438	ABC transporter, permease protein	100	100
BMAA1439	ABC transporter, ATP-binding protein	100	99
BMAA1441	D-methionine-binding lipoprotein metQ, putative	100	100
BMAA1442	hypothetical protein	100	98
BMAA1445	hypothetical protein	100	95
BMAA1446	thiotemplate mechanism natural product synthetase	100	99
BMAA1448	hypothetical protein	97	96
BMAA1454	FkbH domain protein	100	99
BMAA1461	acetyltransferase, GNAT family	100	99
BMAA1469	hypothetical protein	100	96
BMAA1470	hypothetical protein	100	98
BMAA1476	conserved hypothetical protein	100	100
BMAA1477	transcriptional regulator, IclR family	100	99
BMAA1478	C4-dicarboxylate anaerobic carrier family protein	100	97
BMAA1485	hypothetical protein	100	100
BMAA1486	O-methyltransferase family protein	100	99
BMAA1488	hypothetical protein	100	99
BMAA1490	beta-ketoadipyl CoA thiolase	100	99
BMAA1493	major facilitator family transporter	100	99
BMAA1494	hippurate hydrolase	100	100

BMAA1495	transcriptional regulator, LysR family	100	100
BMAA1498	O-antigen acetylase, putative	100	99
BMAA1500	hypothetical protein	100	97
BMAA1503	IS407A, transposase OrfB	100	99
BMAA1504	IS407A, transposase OrfA	100	98
BMAA1506	ISBma2, transposase	100	99
BMAA1507	hypothetical protein	97	98
BMAA1512	IS407A, transposase OrfB	100	99
BMAA1513	IS407A, transposase OrfA	100	98
BMAA1514	hypothetical protein	100	99
BMAA1515	hypothetical protein	100	99
BMAA1522	hypothetical protein	100	97
BMAA1533	type III secretion system protein BsaZ	100	99
BMAA1546	hypothetical protein	100	98
BMAA1552	hypothetical protein	100	98
BMAA1553	hypothetical protein	100	99
BMAA1554	hypothetical protein	100	98
BMAA1555	hypothetical protein	100	97
BMAA1556	transcriptional regulator, putative	100	99
BMAA1561	transcriptional regulator, MarR family	100	97
BMAA1562	conserved hypothetical protein	100	98
BMAA1568	serine protease, kumamolysin	100	98
BMAA1574	AMP-binding enzyme domain protein	100	99
BMAA1575	hypothetical protein	100	100
BMAA1579	hypothetical protein	100	98
BMAA1586	cellulose biosynthesis protein, putative	100	99
BMAA1587	conserved domain protein	100	97
BMAA1589	endo-1,4-D-glucanase	100	98
BMAA1593	hipA protein	100	99
BMAA1594	hypothetical protein	96	96
BMAA1595	hypothetical protein	100	100
BMAA1597.1	hypothetical protein	97	99
BMAA1601	hypothetical protein	100	98
BMAA1603	type IV prepilin	100	96
BMAA1605	type IV pilus biogenesis protein, putative	100	99
BMAA1611	pilL domain protein	100	100
BMAA1612	twitching motility protein PilT, putative	100	99
BMAA1613	type II/III secretion system family protein	100	96
BMAA1616	hypothetical protein	100	98
BMAA1617	hrp protein, putative	100	99
BMAA1618	conserved hypothetical protein	100	99
BMAA1619	hypothetical protein	100	97
BMAA1620	hypothetical protein	99	96
BMAA1623	hypothetical protein	100	98
BMAA1626	conserved hypothetical protein	100	98
BMAA1632	conserved hypothetical protein	100	98
BMAA1633	HrpB2-like protein	100	100
BMAA1635	conserved hypothetical protein	100	99
BMAA1639	type III secretion inner membrane protein SctT, putative	100	97

BMAA1640	hypothetical protein	100	100
BMAA1641	conserved hypothetical protein	100	99
BMAA1642	syringomycin biosynthesis enzyme, putative	100	100
BMAA1643	peptide synthetase, putative	100	99
BMAA1644	IS407A, transposase OrfB	100	99
BMAA1645	IS407A, transposase OrfA	100	98
BMAA1646	peptide synthetase, putative	100	99
BMAA1647	diaminobutyrate--pyruvate aminotransferase/membrane protein, putative	100	99
BMAA1648	hypothetical protein	100	97
BMAA1649	hypothetical protein	100	98
BMAA1650	pyruvate oxidase	100	99
BMAA1651	acetyl-CoA hydrolase/transferase family protein	100	99
BMAA1652	MoaC domain protein	100	100
BMAA1653	citrate lyase, beta subunit, putative	100	99
BMAA1654	transcriptional regulator, LysR family	100	99
BMAA1655	hypothetical protein	100	100
BMAA1656	conserved hypothetical protein	100	98
BMAA1659	conserved hypothetical protein	100	97
BMAA1663	hypothetical protein	100	95
BMAA1664.1	hypothetical protein	100	99
BMAA1668	transporter, putative	100	97
BMAA1671	transporter, putative	100	99
BMAA1672	beta-glucosidase, putative, authentic point mutation	100	98
BMAA1673	hypothetical protein	100	99
BMAA1674	amidase, putative, authentic frameshift	100	95
BMAA1675	conserved hypothetical protein	100	100
BMAA1676	hypothetical protein	100	100
BMAA1677	hypothetical protein	100	99
BMAA1686	amino acid ABC transporter, periplasmic amino acid-binding protein, putative	100	99
BMAA1688	amino acid ABC transporter, ATP-binding/permease protein	100	99
BMAA1689	nitrilotriacetate monooxygenase component A	100	99
BMAA1690	luciferase-like monooxygenase	100	100
BMAA1691	amino acid ABC transporter, periplasmic amino acid-binding protein, putative	100	99
BMAA1692	acetyltransferase, GNAT family	100	99
BMAA1693	conserved hypothetical protein	100	99
BMAA1698	outer membrane porin, putative	100	99
BMAA1702	UTP-glucose-1-phosphate uridylyltransferase	100	100
BMAA1703	hypothetical protein	100	98
BMAA1716	sporulation-related repeat protein	100	99
BMAA1724	conserved hypothetical protein	100	97
BMAA1730	hypothetical protein	100	97
BMAA1731	EDTA monooxygenase, putative	100	100
BMAA1732	hypothetical protein	100	99
BMAA1733	hypothetical protein	100	97
BMAA1734	hypothetical protein	100	98
BMAA1735	hypothetical protein	100	100
BMAA1740	IS407A, transposase OrfA	100	97
BMAA1741	IS407A, transposase OrfB	100	99
BMAA1756	hemagglutinin, homlog	100	98

BMAA1757	hemolysin activator protein, HlyB family	100	100
BMAA1758	transposase, truncated	100	96
BMAA1759	IS407A, transposase OrfA	100	98
BMAA1760	IS407A, transposase OrfB	100	99
BMAA1771	DJ-1/PfpI family protein	100	100
BMAA1772	transcriptional regulator, AraC family	100	100
BMAA1774	drug resistance transporter, Bcr/CflA subfamily, putative	100	98
BMAA1782	hypothetical protein	100	99
BMAA1783	nitroreductase family protein	100	99
BMAA1788	IS407A, transposase OrfA	100	98
BMAA1789	IS407A, transposase OrfB	100	99
BMAA1804	ISBma2, transposase	100	99
BMAA1805	hypothetical protein	100	98
BMAA1807	aminoglycoside 6'-N-acetyltransferase Iz	100	99
BMAA1808	dihydroxyacetone kinase family protein	100	99
BMAA1809	dihydroxyacetone kinase family protein	100	99
BMAA1810	oxidoreductase, zinc-binding dehydrogenase family	100	99
BMAA1811	2-deoxy-D-gluconate 3-dehydrogenase	100	99
BMAA1812	sugar ABC transporter, periplasmic sugar-binding protein, putative	100	100
BMAA1814	sugar ABC transporter, ATP-binding protein	100	99
BMAA1815	ribose 5-phosphate isomerase	100	100
BMAA1816	sugar-binding protein, putative	100	99
BMAA1817	outer membrane porin, putative	100	100
BMAA1818	conserved hypothetical protein TIGR00725	100	98
BMAA1819	ABC transporter, permease protein, putative	100	99
BMAA1820	ABC transporter, periplasmic substrate-binding protein	100	100
BMAA1821	ABC transporter, ATP-binding protein	100	99
BMAA1822	ABC transporter, permease protein, putative	100	99
BMAA1823	hypothetical protein	100	100
BMAA1824	transcriptional regulator, LysR family	100	100
BMAA1825	hypothetical protein	100	100
BMAA1841	glyoxalase family protein	100	99
BMAA1842	hypothetical protein	100	100
BMAA1843	type I phosphodiesterase/nucleotide pyrophosphatase family protein	100	99
BMAA1845	conserved hypothetical protein	100	98
BMAA1848	hypothetical protein	100	98
BMAA1850	hypothetical protein	100	96
BMAA1852	conserved domain protein	100	99
BMAA1854	hypothetical protein	97	97
BMAA1863	conserved hypothetical protein	100	98
BMAA1864	conserved hypothetical protein	100	100
BMAA1865	conserved hypothetical protein	100	99
BMAA1873	hypothetical protein	100	98
BMAA1874	lipoprotein, putative	100	99
BMAA1875	hypothetical protein	100	99
BMAA1877	penicillin amidase, putative	100	99
BMAA1878	transcriptional regulator, LysR family	100	100
BMAA1879	hypothetical protein	100	95
BMAA1880	LamB/YcsF family protein	100	99

BMAA1881	biotin-requiring enzyme domain protein	100	98
BMAA1882	acetyl-CoA carboxylase, biotin carboxylase, putative	100	98
BMAA1883	allophanate hydrolase, subunit 1	100	100
BMAA1884	urea amidolyase-related protein	100	99
BMAA1885	membrane protein, putative	100	99
BMAA1886	hypothetical protein	100	97
BMAA1891	hypothetical protein	100	98
BMAA1892	transcriptional regulator, AraC family, truncation	100	98
BMAA1893	NAD(P)H dehydrogenase, quinone family	100	100
BMAA1894	transcriptional regulator, LysR family	100	99
BMAA1895	conserved domain protein	100	98
BMAA1897	conserved hypothetical protein	100	98
BMAA1898	conserved hypothetical protein	100	98
BMAA1899	hypothetical protein	100	99
BMAA1900	pentapeptide repeat family protein	100	99
BMAA1901	Rhs element Vgr protein	100	99
BMAA1902	conserved hypothetical protein	100	96
BMAA1904	conserved hypothetical protein	100	99
BMAA1905	conserved hypothetical protein	100	98
BMAA1906	conserved hypothetical protein	100	99
BMAA1907	hypothetical protein	100	99
BMAA1909	conserved hypothetical protein	100	99
BMAA1910	conserved hypothetical protein	100	100
BMAA1911	conserved hypothetical protein	100	100
BMAA1912	conserved hypothetical protein	100	99
BMAA1913	conserved hypothetical protein	100	99
BMAA1914	OmpA domain protein	100	99
BMAA1917	hypothetical protein	100	99
BMAA1925	hypothetical protein	100	96
BMAA1928	hypothetical protein	100	95
BMAA1930	hypothetical protein	100	100
BMAA1931	oxidoreductase, short-chain dehydrogenase/reductase family	100	99
BMAA1936	hypothetical protein	100	100
BMAA1943	conserved domain protein	100	98
BMAA1946	IS407A, transposase OrfA	100	98
BMAA1947	IS407A, transposase OrfB	100	99
BMAA1955	hypothetical protein	100	95
BMAA1965	hypothetical protein	100	97
BMAA1967	hypothetical protein	100	96
BMAA1968	ISBma2, transposase	100	99
BMAA1969	hypothetical protein	100	99
BMAA1970	conserved hypothetical protein	100	100
BMAA1971	oxidoreductase, short-chain dehydrogenase/reductase family	100	100
BMAA1972	hypothetical protein	100	96
BMAA1973	conserved hypothetical protein	100	99
BMAA1974	conserved hypothetical protein	100	98
BMAA1975	oxidoreductase, short chain dehydrogenase/reductase family	100	98
BMAA1977	glycosyl transferase, group 1 family protein	100	99
BMAA1978	NAD-dependent epimerase/dehydratase family protein	100	100

BMAA1979	sensory box histidine kinase/response regulator	100	99
BMAA1980	conserved hypothetical protein	100	99
BMAA1981	glycosyl transferase, group 2 family protein	100	100
BMAA1982	sigma-54 dependent DNA-binding response regulator	100	100
BMAA1983	hypothetical protein	100	98
BMAA1984	sigma-54 dependent transcriptional regulator, authentic point mutation	100	99
BMAA1986	ADP-heptose--LPS heptosyltransferase II, putative	100	99
BMAA1987	glycosyl transferase, group 2 family protein	100	100
BMAA1988	carbamoyltransferase family protein	100	99
BMAA1989	heptosyltransferase family protein	100	97
BMAA1990	HAD-superfamily hydrolase	100	99
BMAA1991	oxidoreductase, short chain dehydrogenase/reductase family	100	99
BMAA1992	hypothetical protein	100	99
BMAA1995	conserved domain protein	100	99
BMAA1997	phosphatidylserine decarboxylase, putative	100	99
BMAA1998	succinate dehydrogenase, iron-sulfur protein	100	100
BMAA2002	hypothetical protein	100	98
BMAA2005	hypothetical protein	100	98
BMAA2006.1	hypothetical protein	100	98
BMAA2008	hypothetical protein	100	99
BMAA2011	2-oxoisovalerate dehydrogenase, E2 component, dihydrolipamide acetyltransferase	100	99
BMAA2014	hypothetical protein	100	100
BMAA2015	hypothetical protein	100	96
BMAA2016	hypothetical protein	100	98
BMAA2021	hypothetical protein	100	98
BMAA2036	outer membrane porin, putative	100	100
BMAA2037	medium-chain-fatty-acid--CoA ligase	100	99
BMAA2039	transcriptional regulator, LuxR family	100	99
BMAA2040	hypothetical protein	100	98
BMAA2041	hypothetical protein	100	98
BMAA2042	quinone oxidoreductase	100	100
BMAA2043	alpha-ketoglutarate-dependent taurine dioxygenase	100	99
BMAA2044	conserved hypothetical protein	100	99
BMAA2045	major facilitator family transporter	100	99
BMAA2047	molybdopterin oxidoreductase family protein	100	99
BMAA2048	iron-sulfur cluster-binding protein	100	98
BMAA2049	membrane protein, putative	100	100
BMAA2049.2	hypothetical protein	99	97
BMAA2052	polysaccharide deacetylase family protein	99	96
BMAA2054	conserved domain protein	100	98
BMAA2056	lipoprotein, putative	100	99
BMAA2057	hypothetical protein	98	100
BMAA2057.1	hypothetical protein	100	97
BMAA2059	outer membrane porin, putative	100	99
BMAA2060	hypothetical protein	100	100
BMAA2061.1	hypothetical protein	100	99
BMAA2063	acetyltransferase, GNAT family	100	100
BMAA2064	acetyltransferase, GNAT family	100	100
BMAA2065	conserved hypothetical protein	100	99

BMAA2066	TldD/PmbA family protein	100	100
BMAA2067	conserved domain protein	100	97
BMAA2068	hypothetical protein	100	99
BMAA2069	hypothetical protein	100	100
BMAA2073	IS407A, transposase OrfB	100	99
BMAA2074	IS407A, transposase OrfA	100	98
BMAA2075	ISBma2, transposase	100	99
BMAA2100.1	hypothetical protein	100	98
BMAA2104	esterase EstC	100	99
BMAA2108	hypothetical protein	100	97
BMAA2116	ISBma2, transposase	100	99
BMAA2118	hypothetical protein	100	97

Segment 3- Bm-unique

Gene	Description
BMA0007	hypothetical protein
BMA0008	FG-GAP/YD repeat domain protein
BMA0009	hypothetical protein
BMA0016.1	hypothetical protein
BMA0017	hypothetical protein
BMA0090	hypothetical protein
BMA0099	conserved hypothetical protein
BMA0108	conserved hypothetical protein
BMA0112	hypothetical protein
BMA0119	hypothetical protein
BMA0126	hypothetical protein
BMA0127	hypothetical protein
BMA0178	N-acyl-D-amino-acid deacylase family protein
BMA0212	membrane protein, putative, authentic frameshift
BMA0233	conserved hypothetical protein, authentic frameshift
BMA0253	hypothetical protein
BMA0255	hypothetical protein
BMA0262.1	hypothetical protein
BMA0267	conserved domain protein, truncation
BMA0286	molybdenum cofactor biosynthesis protein C, truncation
BMA0287	conserved hypothetical protein
BMA0318	hypothetical protein
BMA0332	tagatose 6-phosphate kinase, putative, authentic frameshift
BMA0361	thioredoxin, authentic frameshift
BMA0364	hypothetical protein
BMA0379	conserved hypothetical protein, authentic frameshift
BMA0386	sigma-54 dependent DNA-binding transcriptional regulator
BMA0409	acyl-CoA dehydrogenase domain protein, authentic frameshift
BMA0419	hypothetical protein
BMA0440	ISBma2, transposase, truncation
BMA0482	hypothetical protein
BMA0497	site-specific recombinase, phage integrase family, truncation
BMA0522	phosphoglycolate phosphatase, putative, degenerate
BMA0569	hypothetical protein

BMA0570	conserved hypothetical protein
BMA0582.1	hypothetical protein
BMA0587	IS407A, transposase OrfB, truncation
BMA0601	ubiquinol oxidase, subunit III, authentic frameshift
BMA0602	ubiquinol oxidase, subunit I, authentic frameshift
BMA0605	conserved hypothetical protein
BMA0612	SUF system FeS assembly ATPase SufC, internal deletion
BMA0613	FeS assembly protein SufB , authentic frameshift
BMA0642	conserved hypothetical protein, degenerate
BMA0702	hypothetical protein
BMA0737	endo/excinuclease domain protein
BMA0738	beta-glucosidase, degenerate
BMA0748	conserved hypothetical protein, authentic frameshift
BMA0756	phage integrase, truncation
BMA0789.1	hypothetical protein
BMA0792	hypothetical protein
BMA0801	conserved hypothetical protein
BMA0818	maltooligosyl trehalose trehalohydrolase, putative
BMA0820	1,4-alpha-glucan branching enzyme, authentic frameshift
BMA0824.1	hypothetical protein
BMA0844	hypothetical protein
BMA0850	conserved hypothetical protein, authentic frameshift
BMA0850.1	hypothetical protein
BMA0876	fimbrial usher family protein, authentic frameshift
BMA0881	conserved hypothetical protein, authentic frameshift
BMA0889	beta-N-acetylglucosaminidase, putative, authentic frameshift
BMA0894	CAIB/BAIF family protein
BMA0897	conserved hypothetical protein, truncation
BMA0901	serine protease, authentic frameshift
BMA0945	acyltransferase, putative, authentic frameshift
BMA0947	ISBma4, transposase, truncation
BMA0951	hypothetical protein
BMA0964	hypothetical protein
BMA0971	nonribosomal peptide synthetase, putative, degenerate
BMA0999.1	hypothetical protein
BMA1009	alcohol dehydrogenase, iron-containing, authentic frameshift
BMA1010	hypothetical protein
BMA1014	hypothetical protein
BMA1021	type-1 fimbrial protein, authentic frameshift
BMA1026	hypothetical protein
BMA1027	outer membrane protein, putative
BMA1037	hypothetical protein
BMA1084	conserved hypothetical protein
BMA1117	transposase, IS3 family, truncation
BMA1122	hypothetical protein
BMA1170	CobN/magnesium chelatase family protein
BMA1174	cbiG protein
BMA1182	hypothetical protein
BMA1184	conserved hypothetical protein

BMA1192	hypothetical protein
BMA1224.1	hypothetical protein
BMA1267	methyl-accepting chemotaxis domain protein
BMA1287	membrane protein, putative
BMA1288	secretion system protein, putaive, authentic frameshift
BMA1297	hypothetical protein
BMA1313	phosphoserine phosphatase
BMA1386	aminotransferase, classes I and II, authentic frameshift
BMA1405	conserved hypothetical protein
BMA1411	ISBma4, transposase, truncation
BMA1414.1	hypothetical protein
BMA1452	hypothetical protein
BMA1455	carboxymuconolactone decarboxylase family protein
BMA1456	conserved hypothetical protein
BMA1470	hypothetical protein
BMA1477	hypothetical protein
BMA1488.1	hypothetical protein
BMA1525	hypothetical protein
BMA1527	conserved hypothetical protein, authentic frameshift
BMA1561	hypothetical protein
BMA1595	hypothetical protein
BMA1634.1	nonribosomal peptide synthetase, authentic frameshift
BMA1649	hypothetical protein
BMA1673	peptidyl-prolyl cis-trans isomerase, FKBP-type, authentic frameshift
BMA1674.1	hypothetical protein
BMA1720	pyruvate dehydrogenase, E2 component, dihydrolipoamide acetyltransferase
BMA1766	EAL domain protein
BMA1781	conserved hypothetical protein, authentic frameshift
BMA1787	transcriptional regulator, putative
BMA1843	2-isopropylmalate synthase, authentic frameshift
BMA1868	membrane protein, putative, authentic frameshift
BMA1871	conserved domain protein
BMA1873	sensor histidine kinase KdpD, authentic frameshift
BMA1894.1	hypothetical protein
BMA1924	cell division protein FtsK, putative
BMA1925	hypothetical protein
BMA2003	hypothetical protein
BMA2007	hypothetical protein
BMA2016	transcriptional regulator, putative
BMA2029	transport-associated domain protein
BMA2034	iron compound ABC transporter, periplasmic iron-compound binding protein, authentic frameshift
BMA2043.1	hypothetical protein
BMA2085	IS110 transposase, degenerate
BMA2114	acetyltransferase, GNAT family
BMA2185	urease accessory protein UreE
BMA2253.1	hypothetical protein
BMA2253.2	hypothetical protein
BMA2253.3	membrane protein, putative
BMA2260	hypothetical protein

BMA2426	hypothetical protein
BMA2459	ureidoglycolate hydrolase, authentic frameshift
BMA2508	hypothetical protein
BMA2541	hypothetical protein
BMA2580	acetyltransferase, GNAT family
BMA2692	terminase, large subunit, truncation
BMA2808	hypothetical protein
BMA2812	hypothetical protein
BMA2849	lipoprotein, putative
BMA2859	chemotaxis protein CheA
BMA2861	chemotaxis MotB protein, putative, authentic frameshift
BMA2899.1	hypothetical protein
BMA2920	oxidoreductase, FAD/FMN-binding
BMA2996	conserved domain protein
BMA3005	site-specific recombinase, phage integrase family
BMA3008	lipoprotein, putative
BMA3009	lipoprotein, putative
BMA3012	hypothetical protein
BMA3027	conserved hypothetical protein, truncation
BMA3028	conserved domain protein
BMA3032	triphosphoribosyl-dephospho-CoA synthase
BMA3043.1	hypothetical protein
BMA3054	conserved hypothetical protein, authentic frameshift
BMA3065	hypothetical protein
BMA3069	hypothetical protein
BMA3074	hypothetical protein
BMA3100	adenine phosphoribosyltransferase, authentic frameshift
BMA3117	lipoprotein, putative
BMA3173.1	hypothetical protein
BMA3227	C4-dicarboxylate transport sensor protein, putative, authentic frameshift
BMA3272	conserved domain protein
BMA3274	acyl-CoA dehydrogenase domain protein, authentic frameshift
BMA3290	DNA-binding response regulator, LuxR family
BMA3300	hypothetical protein
BMA3303	dipeptide ABC transporter, permease protein, authentic frameshift
BMA3324	hypothetical protein
BMA3338	transcriptional regulator, LysR family, authentic frameshift
BMA3373	hypothetical protein
BMA3392	outer membrane porin, truncation
BMAA0034	hypothetical protein
BMAA0038	hypothetical protein
BMAA0057.1	hypothetical protein
BMAA0063	hypothetical protein
BMAA0070	sensor histidine kinase, authentic frameshift
BMAA0074	hypothetical protein
BMAA0075	hypothetical protein
BMAA0081	conserved domain protein
BMAA0118	hypothetical protein
BMAA0119	hypothetical protein

BMAA0136	hypothetical protein
BMAA0140	RND efflux system, membrane fusion protein, authentic frameshift
BMAA0149	outer membrane efflux lipoprotein, NodT family, authentic frameshift
BMAA0151	hypothetical protein
BMAA0161	hypothetical protein
BMAA0167	hypothetical protein
BMAA0173	ISBma1, transposase, interruption-C
BMAA0178	hypothetical protein
BMAA0209	hypothetical protein
BMAA0210	conserved hypothetical protein, authentic frameshift
BMAA0224	hypothetical protein
BMAA0227	hypothetical protein
BMAA0229	hypothetical protein
BMAA0236	hypothetical protein
BMAA0248	hypothetical protein
BMAA0249	hypothetical protein
BMAA0257	hypothetical protein
BMAA0264	hypothetical protein
BMAA0265	hypothetical protein
BMAA0281	hypothetical protein
BMAA0285	conserved hypothetical protein, authentic frameshift
BMAA0295	hypothetical protein
BMAA0310	site-specific recombinase, phage integrase family
BMAA0315	hypothetical protein
BMAA0323	hypothetical protein
BMAA0326	hypothetical protein
BMAA0333	hypothetical protein
BMAA0386	D-serine dehydratase, authentic frameshift
BMAA0391	monooxygenase family protein
BMAA0395	hypothetical protein
BMAA0400	conserved hypothetical protein
BMAA0411	conserved hypothetical protein, authentic frameshift
BMAA0412	YD repeat protein
BMAA0415	conserved hypothetical protein, truncation
BMAA0428	lipase, authentic frameshift
BMAA0434	spermidine/putrescine ABC transporter, permease protein, authentic frameshift
BMAA0485	ABC transporter, periplasmic glycine/betaine-binding protein, putative
BMAA0496	oxidoreductase, short-chain dehydrogenase/reductase family, truncation
BMAA0539.1	hypothetical protein
BMAA0554	di-haem cytochrome c peroxidase family protein, authentic frameshift
BMAA0562	hypothetical protein
BMAA0570	hypothetical protein
BMAA0597	hypothetical protein
BMAA0610	di-haem cytochrome c peroxidase family protein
BMAA0620	LysR substrate-binding domain family protein, degenerate
BMAA0623	hypothetical protein
BMAA0631	feruloyl-CoA synthetase, putative, authentic frameshift
BMAA0635	hypothetical protein
BMAA0649	Hep_Hag family protein/haemagluttinin motif family protein/YadA-like domain protein

BMAA0650	hypothetical protein
BMAA0682	hypothetical protein
BMAA0683	hypothetical protein
BMAA0686	leucyl-tRNA synthetase, authentic frameshift
BMAA0693	hypothetical protein
BMAA0695	hypothetical protein
BMAA0710	hypothetical protein
BMAA0713	hypothetical protein
BMAA0716	hypothetical protein
BMAA0749	hemagglutinin domain protein
BMAA0771	hypothetical protein
BMAA0775	hypothetical protein
BMAA0778	aminotransferase family protein
BMAA0781	sulfite reductase (NADPH) flavoprotein alpha-component, authentic frameshift
BMAA0789	hypothetical protein
BMAA0807	hypothetical protein
BMAA0810	YadA-like C-terminal region protein
BMAA0813	hypothetical protein
BMAA0820	cytochrome c4 family protein
BMAA0825	hypothetical protein
BMAA0838	hypothetical protein
BMAA0843	hypothetical protein
BMAA0844	transposase, degenerate
BMAA0862	hypothetical protein
BMAA0871	hypothetical protein
BMAA0877	hypothetical protein
BMAA0889	conserved domain protein
BMAA0893	sarcosine oxidase, gamma subunit, authentic frameshift
BMAA0895	hypothetical protein
BMAA0902	hypothetical protein
BMAA0902.1	hypothetical protein
BMAA0926	hypothetical protein
BMAA0928	hypothetical protein
BMAA0941	hypothetical protein
BMAA0950	major facilitator family transporter
BMAA0956	conserved domain protein
BMAA0960	hypothetical protein
BMAA0965	hypothetical protein
BMAA0967	hypothetical protein
BMAA0985	hypothetical protein
BMAA0989	conserved hypothetical protein
BMAA1012	hypothetical protein
BMAA1015	hypothetical protein
BMAA1035	hypothetical protein
BMAA1038	amino acid permease
BMAA1051	phage integrase family protein
BMAA1052	hypothetical protein
BMAA1053	conserved hypothetical protein
BMAA1060	hypothetical protein

BMAA1083	hypothetical protein
BMAA1093	hypothetical protein
BMAA1102	hypothetical protein
BMAA1103	transcriptional regulator, LysR family
BMAA1108	hypothetical protein
BMAA1109	hypothetical protein
BMAA1116	conserved hypothetical protein
BMAA1124	hypothetical protein
BMAA1129	ABC transporter, permease protein, authentic frameshift
BMAA1134	2-dehydro-3-deoxyglucarate aldolase, authentic frameshift
BMAA1152	hypothetical protein
BMAA1156	hypothetical protein
BMAA1171	conserved hypothetical protein, authentic frameshift
BMAA1173	conserved domain protein
BMAA1176	hypothetical protein
BMAA1177	hypothetical protein
BMAA1178.1	ribose transport system permease protein RbsC, internal deletion
BMAA1189	transcriptional regulator, AraC family
BMAA1194	hypothetical protein
BMAA1203	polyketide synthase, putative, degenerate
BMAA1204	polyketide synthase, putative
BMAA1205	polyketide synthase, putative
BMAA1206	polyketide synthase, putative, degenerate
BMAA1207	hypothetical protein
BMAA1219	TPR domain protein
BMAA1225	response regulator, authentic frameshift
BMAA1228	histidine kinase
BMAA1229	hypothetical protein
BMAA1246.1	hypothetical protein
BMAA1257	hypothetical protein
BMAA1260	hypothetical protein
BMAA1279	hypothetical protein
BMAA1300	membrane protein, putative
BMAA1306	monooxygenase, truncation
BMAA1310	pyridine nucleotide-disulphide oxidoreductase, class II, truncation
BMAA1354	hypothetical protein
BMAA1369	hypothetical protein
BMAA1375	L-2-Amino-thiazoline-4-carboxylic acid hydrolase, authentic frameshift
BMAA1382	hypothetical protein
BMAA1383	hypothetical protein
BMAA1388	hypothetical protein
BMAA1389	conserved hypothetical protein
BMAA1393	methyl-accepting chemotaxis protein, putative, authentic frameshift
BMAA1402	conserved hypothetical protein, authentic frameshift
BMAA1404	hypothetical protein
BMAA1413	ISBma3, transposase, truncation
BMAA1421	amino acid permease
BMAA1427	hypothetical protein
BMAA1444	hypothetical protein

BMAA1453	ketol-acid reductoisomerase, putative
BMAA1472	hypothetical protein
BMAA1475	porin, degenerate
BMAA1483	hypothetical protein
BMAA1489	hypothetical protein
BMAA1497	hypothetical protein
BMAA1516	ISBma5, transposase, authentic frameshift
BMAA1526	BapA protein
BMAA1538	BsaU protein
BMAA1558	hypothetical protein
BMAA1560	hypothetical protein
BMAA1567	hypothetical protein
BMAA1571	conserved domain protein
BMAA1591	hypothetical protein
BMAA1592	hipB domain protein
BMAA1599	hypothetical protein
BMAA1625	type III secretion inner membrane protein, authentic frameshift
BMAA1629	type III secretion inner membrane protein SctQ
BMAA1634	type III secretion inner membrane protein SctJ, authentic frameshift
BMAA1670	hypothetical protein
BMAA1684	hypothetical protein
BMAA1687	hypothetical protein
BMAA1694	hypothetical protein
BMAA1695	lipoprotein, putative
BMAA1699	hypothetical protein
BMAA1704	acyltransferase, putative, authentic frameshift
BMAA1707	conserved hypothetical protein
BMAA1710	GDP-6-deoxy-D-lyxo-4-hexulose reductase, putative
BMAA1711.1	hypothetical protein
BMAA1742	hypothetical protein
BMAA1762	hypothetical protein
BMAA1765	oxidoreductase, 2OG-Fe(II) oxygenase family
BMAA1784	hypothetical protein
BMAA1787	alkyl hydroperoxide reductase, subunit f, truncation
BMAA1790	conserved hypothetical protein, truncation
BMAA1795	membrane protein, putative, authentic frameshift
BMAA1813	sugar ABC transporter, permease protein
BMAA1838	conserved hypothetical protein
BMAA1839	transcriptional regulator, LysR family, authentic frameshift
BMAA1849	hypothetical protein
BMAA1851	copper-translocating P-type ATPase
BMAA1859	drug resistance transporter family protein, authentic frameshift
BMAA1861	hypothetical protein
BMAA1866	conserved hypothetical protein
BMAA1887	hypothetical protein
BMAA1888	hypothetical protein
BMAA1896	hypothetical protein
BMAA1903	conserved hypothetical protein, authentic frameshift
BMAA1908	ATP-dependent Clp protease, ATP-binding subunit ClpB, authentic frameshift

BMAA1915	conserved hypothetical protein, authentic frameshift
BMAA1916	hypothetical protein
BMAA1935	hypothetical protein
BMAA1938	hypothetical protein
BMAA1942	transporter, putative, authentic frameshift
BMAA1945	hypothetical protein
BMAA1948	conserved domain protein
BMAA1949	CAIB/BAIF family protein, authentic frameshift
BMAA1959	hypothetical protein
BMAA1975.1	hypothetical protein
BMAA1985	transferase, putative, authentic frameshift
BMAA1993	response regulator
BMAA1994	ABC transporter, ATP-binding protein, putative
BMAA1999	hypothetical protein
BMAA2024.1	hypothetical protein
BMAA2028.1	hypothetical protein
BMAA2032	hypothetical protein
BMAA2038	hypothetical protein
BMAA2046	conserved hypothetical protein, authentic frameshift
BMAA2049.1	hypothetical protein
BMAA2053	hypothetical protein
BMAA2089	polyketide synthase, putative
BMAA2103	hypothetical protein
BMAA2112	hypothetical protein

Segment 4- Conserved between Bm and Bt but not as well or absent in Bp

Gene	Description	to Bt % length	% identity
BMA0016	hypothetical protein	99	87
BMA0632	conserved hypothetical protein	100	94
BMA0808	conserved hypothetical protein	99	95
BMA0860	manganese/iron transporter, NRAMP family	100	91
BMA0935	RND efflux system, outer membrane lipoprotein, NodT family protein	98	86
BMA1076	ISBma1, transposase, truncation	99	90
BMA1133	hypothetical protein	100	94
BMA1194	carbohydrate kinase, FGGY family	100	93
BMA1233	ISBma1, transposase, interruption-C	95	98
BMA1280	hfq protein	100	89
BMA2657	conserved hypothetical protein	100	86
BMA3093	single-strand binding protein	100	91
BMAA0435	serine protease, subtilase family, truncation	95	93
BMAA0611	phosphoesterase family protein	100	95
BMAA0799	nitric oxide reductase	100	90
BMAA0854	hypothetical protein	100	86
BMAA1298	methyl-accepting chemotaxis protein	99	91
BMAA2091	membrane protein, putative	98	88

Segment 5- Bp unique

Gene	Description

BPSL0066	putative membrane protein
BPSL0068	putative lipoprotein
BPSL0081	putative phage integrase
BPSL0083	hypothetical protein
BPSL0084	hypothetical protein
BPSL0085	conserved hypothetical protein
BPSL0086	hypothetical protein
BPSL0087	putative DNA-binding protein
BPSL0088	conserved hypothetical protein
BPSL0089	hypothetical protein
BPSL0092	putative lipoprotein
BPSL0095	hypothetical protein
BPSL0105	hypothetical protein
BPSL0129	prophage integrase
BPSL0130	conserved hypothetical phage protein
BPSL0130a	hypothetical phage protein
BPSL0131	hypothetical phage protein
BPSL0132	hypothetical phage protein
BPSL0133	hypothetical phage protein
BPSL0134	putative phage-encoded membrane protein
BPSL0135	conserved hypothetical phage protein
BPSL0136	hypothetical phage protein
BPSL0137	hypothetical phage protein
BPSL0138	putative phage protein
BPSL0139	putative phage DNA-binding protein
BPSL0140	hypothetical phage protein
BPSL0141	putative phage DNA-binding protein
BPSL0142	putative phage-encoded membrane protein
BPSL0143	hypothetical phage protein
BPSL0146	putative phage-encoded membrane protein
BPSL0158	putative phage protein
BPSL0176	putative phage-encoded membrane protein
BPSL0221	conserved hypothetical protein (partial)
BPSL0234	hypothetical protein
BPSL0269	putative flagella basal body P-ring formation protein
BPSL0306	putative membrane protein
BPSL0337	MutT/NUDIX family protein
BPSL0339	putative lipoprotein
BPSL0343	putative hydrolase
BPSL0344	putative exported protein
BPSL0345	hypothetical protein
BPSL0347	putative insertion element protein
BPSL0348	hypothetical protein
BPSL0349	conserved hypothetical protein
BPSL0350	Fis family regulatory protein
BPSL0377	hypothetical protein
BPSL0548	hypothetical protein
BPSL0549	hypothetical protein
BPSL0550	hypothetical protein

BPSL0551	hypothetical protein
BPSL0552	hypothetical protein
BPSL0553	putative DNA-binding protein
BPSL0554	hypothetical phage protein
BPSL0555	putative membrane protein
BPSL0556	hypothetical protein
BPSL0557	hypothetical protein
BPSL0558	putative DNA-binding protein
BPSL0559	hypothetical protein
BPSL0560	hypothetical protein
BPSL0561	putative exported protein
BPSL0562	putative DNA-binding protein
BPSL0563	hypothetical protein
BPSL0564	hypothetical protein
BPSL0565	hypothetical protein
BPSL0566	hypothetical protein
BPSL0567	hypothetical protein
BPSL0568	hypothetical protein
BPSL0569	conserved hypothetical protein
BPSL0570	conserved hypothetical protein
BPSL0571	putative membrane protein
BPSL0572	hypothetical protein
BPSL0573	putative exported protein
BPSL0574	subtilase family protein
BPSL0575	hypothetical protein
BPSL0576	hypothetical protein
BPSL0577	phage integrase family protein
BPSL0582	hypothetical protein
BPSL0583	hypothetical protein
BPSL0584	putative membrane protein
BPSL0585	hypothetical protein
BPSL0586	hypothetical protein
BPSL0586a	hypothetical protein
BPSL0587	phage integrase family protein
BPSL0588	hypothetical protein
BPSL0681	conserved hypothetical protein
BPSL0701	putative exported protein
BPSL0708	putative transport protein (partial)
BPSL0709	putative LysR family transcriptional regulator
BPSL0711	putative membrane protein
BPSL0714	hypothetical protein (pseudogene)
BPSL0718	putative phosphoribosyl transferase protein
BPSL0720	putative alcohol dehydrogenase cytochrome c subunit precursor
BPSL0721	putative membrane protein
BPSL0722	putative cytochrome c oxidase subunit I
BPSL0723	putative cytochrome c oxidase polypeptide II precursor
BPSL0724	putative thiamine pyrophosphate requiring enzyme
BPSL0725	putative mandelate racemase
BPSL0726	putative membrane protein

BPSL0727	hypothetical protein
BPSL0728	putative glucose dehydrogenase
BPSL0729	conserved hypothetical protein
BPSL0730	putative penicillin acylase II
BPSL0731	LysR family transcriptional regulator
BPSL0735	hypothetical protein
BPSL0736	hypothetical protein
BPSL0737	hypothetical protein
BPSL0738	hypothetical protein
BPSL0739	hypothetical protein
BPSL0740	hypothetical protein
BPSL0741	conserved hypothetical protein
BPSL0742	putative membrane protein
BPSL0743	conserved hypothetical protein
BPSL0744	putative phage-related integrase
BPSL0745	hypothetical protein
BPSL0746	hypothetical protein
BPSL0747	hypothetical protein
BPSL0747a	hypothetical protein
BPSL0748	hypothetical protein
BPSL0749	hypothetical protein
BPSL0750	hypothetical protein
BPSL0751	hypothetical protein
BPSL0752	hypothetical protein
BPSL0753	hypothetical protein
BPSL0754	putative integrase/recombinase (partial)
BPSL0756	putative DNA-binding protein
BPSL0757	hypothetical protein
BPSL0758	putative phosphoesterase
BPSL0759	hypothetical protein
BPSL0760	hypothetical protein
BPSL0761	hypothetical protein
BPSL0762	putative RNA 2'-phosphotransferase
BPSL0763	putative helicase SNF2 family protein
BPSL0764	hypothetical protein
BPSL0765	putative helicase family protein
BPSL0766	hypothetical protein
BPSL0767	putative phospholipase protein
BPSL0768	conserved hypothetical protein
BPSL0769	hypothetical protein
BPSL0770	conserved hypothetical protein
BPSL0771	hypothetical protein
BPSL0772	phage integrase family protein
BPSL0828	putative tagatose 6-phosphate kinase protein
BPSL0889	hypothetical protein
BPSL0892a	putative lipoprotein
BPSL0938A	hypothetical protein
BPSL0939	putative DeoR family regulatory protein
BPSL0940	conserved hypothetical protein

BPSL0941	hypothetical protein
BPSL0942	hypothetical protein
BPSL0943	putative insertion element protein
BPSL0944	putative phage integrase/recombinase protein
BPSL0945	conserved hypothetical protein
BPSL0946	conserved hypothetical protein
BPSL0947	putative type I restriction enzyme specificity protein
BPSL0948	putative type I restriction-modification methylase
BPSL0949	hypothetical protein
BPSL0951	insertion element hypothetical protein (partial)
BPSL0952	putative replication protein
BPSL0953	hypothetical protein
BPSL0954	hypothetical protein
BPSL0970	putative isoleucine biosynthesis transcriptional activator
BPSL1022	hypothetical protein
BPSL1023	putative beta-glucosidase (partial)
BPSL1041	hypothetical protein
BPSL1047	hypothetical protein
BPSL1051	putative membrane protein (partial)
BPSL1058	putative transposase (partial)
BPSL1059	conserved hypothetical protein
BPSL1060	hypothetical protein
BPSL1109	putative membrane protein
BPSL1137	hypothetical protein
BPSL1138	hypothetical protein
BPSL1139	hypothetical protein
BPSL1140	putative phage-related protein
BPSL1141	hypothetical protein
BPSL1142	putative phage-related protein
BPSL1143	putative phage terminase
BPSL1144	putative exported protein
BPSL1145	putative phage-related protein
BPSL1146	hypothetical protein
BPSL1147	hypothetical protein
BPSL1148	hypothetical protein
BPSL1149	hypothetical protein
BPSL1150	hypothetical protein
BPSL1151	hypothetical protein
BPSL1152	hypothetical protein
BPSL1153	hypothetical protein
BPSL1153A	hypothetical protein
BPSL1154	hypothetical protein
BPSL1155	conserved hypothetical protein
BPSL1156	hypothetical protein
BPSL1157	putative phage integrase
BPSL1252	putative phosphorous compounds metabolism-related protein
BPSL1255	hypothetical protein
BPSL1274	hypothetical protein
BPSL1290a	hypothetical protein

BPSL1293	putative membrane protein
BPSL1317	putative lipoprotein
BPSL1368	hypothetical protein
BPSL1384	putative phage-related protein (partial)
BPSL1384a	putative phage-related protein (partial)
BPSL1385	hypothetical protein
BPSL1386	hypothetical protein
BPSL1390	hypothetical protein
BPSL1391	putative phage related protein (partial)
BPSL1392	hypothetical protein
BPSL1393	putative exported avidin family protein
BPSL1408	conserved hypothetical protein (partial)
BPSL1409	hypothetical protein
BPSL1457	conserved hypothetical protein
BPSL1565	putative MerR-family transcriptional regulator
BPSL1584	peptide synthetase nrps12
BPSL1612	hypothetical protein
BPSL1619	putative alcohol dehydrogenase (pseudogene)
BPSL1621	hypothetical protein
BPSL1624	hypothetical protein
BPSL1626	putative fimbrial subunit type 1 precursor
BPSL1630	hypothetical protein
BPSL1631	putative outer membrane protein
BPSL1634	putative two-component regulatory system, sensor kinase protein
BPSL1635	conserved hypothetical protein
BPSL1636	putative regulatory protein
BPSL1637	putative lipase
BPSL1639	putative transposase (partial)
BPSL1642	putative GntR-family regulatory protein
BPSL1643	conserved hypothetical protein
BPSL1644	putative hydrolase
BPSL1645	putative oxygenase
BPSL1646	putative monooxygenase
BPSL1647	putative betaine aldehyde dehydrogenase
BPSL1648	conserved hypothetical protein
BPSL1649	putative ABC transport system, substrate-binding protein
BPSL1650	putative ABC transport system, permease protein
BPSL1651	putative ABC transport system, permease protein
BPSL1652	putative ABC transport system, ATP-binding protein
BPSL1653	putative GntR-family regulatory protein
BPSL1654	succinate-semialdehyde dehydrogenase [NADP+]
BPSL1655	putative outer membrane porin protein
BPSL1656	hypothetical protein
BPSL1658a	putative DNA-biding protein, H-NS-like
BPSL1659	putative exported protein
BPSL1660	putative outer membrane protein
BPSL1661	putative hemolysin-related protein
BPSL1662	hypothetical protein
BPSL1663	conserved hypothetical protein

BPSL1664	putative toxin transport-related membrane protein
BPSL1665	putative toxin-related secretion protein
BPSL1666	hypothetical protein
BPSL1667	hypothetical protein
BPSL1668	putative adenylylsulfate kinase
BPSL1669	putative two component system, response regulator
BPSL1670	putative transposase
BPSL1671	putative exported protein
BPSL1672	hypothetical protein
BPSL1673	putative tranposase (partial)
BPSL1674	putative outer membrane porin protein precursor
BPSL1675	putative transposase
BPSL1676	putative exported histidine ammonia-lyase
BPSL1677	putative transport-related, integral membrane protein
BPSL1678	putative urocanate hydratase
BPSL1679	conserved hypothetical protein
BPSL1680	putative LysR-family transcriptional regulatory protein
BPSL1681	putative allantoinase
BPSL1682	putative aminohydrolase
BPSL1683	conserved hypothetical protein (partial)
BPSL1684	putative transposase (partial)
BPSL1685	putative transposase (partial)
BPSL1686	putative RNA polymerase sigma factor
BPSL1687	putative membrane protein
BPSL1688	putative membrane protein
BPSL1689	putative membrane protein
BPSL1690	conserved hypothetical protein
BPSL1691	conserved hypothetical protein
BPSL1692	putative exported protein
BPSL1693	insertion element hypothetical protein (partial)
BPSL1694	putative transposase (partial)
BPSL1695	putative GntR-family transcriptional regulator
BPSL1696	putative recombinase
BPSL1697	putative recombinase (partial)
BPSL1699	hypothetical protein
BPSL1700	conserved hypothetical protein (partial)
BPSL1702	putative invertase
BPSL1703	transposase (partial)
BPSL1704	transposase
BPSL1704a	hypothetical protein
BPSL1705	putative membrane protein
BPSL1706	putative HNS-like protein
BPSL1707	putative exported oxidase
BPSL1708	putative exported protein
BPSL1708A	putative insertion element (partial)
BPSL1709	putative non-ribosomal peptide synthase (partial)
BPSL1711	putative carbamoyl transferase
BPSL1712	putative non-ribosomal antibiotic-related peptide synthase
BPSL1713	putative acetyltransferase

BPSL1714	putative threonine aldolase
BPSL1715	putative bifunctional protein (ligase and argininosuccinate lyase)
BPSL1716	putative cysteine synthase
BPSL1717	hypothetical protein
BPSL1718	putative membrane protein
BPSL1719	putative kinase
BPSL1720	putative argininosuccinate lyase
BPSL1721	putative argininosuccinate synthase
BPSL1722	putative formyl transferase
BPSL1723	hypothetical protein
BPSL1724	putative histidinol-phosphate aminotransferase
BPSL1727	putative non-ribosomal peptide synthase (thioesterase domain)
BPSL1789	putative sugar kinase (pseudogene)
BPSL1796	hypothetical protein
BPSL1799	putative fimbrial chaperone
BPSL1800	putative outer membrane usher protein precursor
BPSL1801	putative type-1 fimbrial protein
BPSL1812	putative membrane protein
BPSL1822	putative membrane protein
BPSL1823	putative ABC transport system, ATP-binding protein
BPSL1824	putative ABC transport system, substrate-binding protein
BPSL1829	putative methyl-accepting chemotaxis protein
BPSL1889	putative lipoprotein
BPSL1922	conserved hypothetical protein
BPSL1934	putative exported protein
BPSL1949	conserved hypothetical protein (partial)
BPSL1972	putative antibiotic resistance membrane protein
BPSL2006	putative signal peptide protease
BPSL2007	putative membrane protein
BPSL2022	putative drug-resistance related outer-membrane protein
BPSL2037	hypothetical protein
BPSL2038	putative exported protein
BPSL2039	putative membrane protein
BPSL2040	hypothetical protein
BPSL2042	hypothetical protein
BPSL2042A	hypothetical protein
BPSL2043	putative lipoprotein
BPSL2044	hypothetical protein
BPSL2048	conserved hypothetical protein
BPSL2054	hypothetical protein
BPSL2055	conserved hypothetical protein
BPSL2059	putative exported protein
BPSL2064	hypothetical protein
BPSL2071	putative membrane protein
BPSL2076	1,4-alpha-glucan branching enzyme
BPSL2078	putative trehalose trehalohydrolase protein
BPSL2081	conserved hypothetical protein
BPSL2087A	hypothetical protein
BPSL2088	hypothetical protein

BPSL2089	putative phage protein (partial)
BPSL2119	putative amino acid racemase
BPSL2131	conserved hypothetical protein (pseudogene)
BPSL2173	hypothetical protein
BPSL2221	hypothetical protein
BPSL2331	hypothetical protein
BPSL2332	hypothetical protein
BPSL2333	conserved hypothetical protein
BPSL2334	conserved hypothetical protein
BPSL2352	conserved hypothetical protein (pseudogene)
BPSL2513	putative membrane protein
BPSL2551	putative exported protein
BPSL2558	hypothetical protein
BPSL2568	hypothetical protein
BPSL2569	putative exported protein
BPSL2570	hypothetical protein
BPSL2571	hypothetical protein
BPSL2572	hypothetical protein
BPSL2573	hypothetical protein
BPSL2574	hypothetical protein
BPSL2575	hypothetical protein
BPSL2576	hypothetical protein
BPSL2577	hypothetical protein
BPSL2578	hypothetical protein
BPSL2579	hypothetical protein
BPSL2580	hypothetical protein
BPSL2581	hypothetical protein
BPSL2582	hypothetical protein
BPSL2583	hypothetical protein
BPSL2584	hypothetical protein
BPSL2585	hypothetical protein
BPSL2586	putative phage integrase
BPSL2699	putative exported protein
BPSL2702	putative membrane protein
BPSL2767	putative flagellar transcriptional activator (partial)
BPSL2768	hypothetical protein
BPSL2773	putative glycosyltransferase protein
BPSL2777	putative O-antigen translocase
BPSL2784	putative membrane protein
BPSL2859	conserved hypothetical protein
BPSL3095	hypothetical protein
BPSL3113	putative integrase (partial)
BPSL3114	hypothetical protein
BPSL3115	putative transcriptional regulator
BPSL3118	putative restriction modification system methylase
BPSL3255	putative membrane protein
BPSL3257	putative plasmid recombinase
BPSL3258	conserved hypothetical protein
BPSL3259	putative plasmid conjugal transfer protein

BPSL3260	conserved hypothetical protein
BPSL3261	hypothetical protein
BPSL3262	putative plasmid conjugal transfer protein
BPSL3263	putative plasmid conjugal transfer protein
BPSL3264	putative plasmid conjugal transfer protein
BPSL3265	putative plasmid conjugal transfer protein
BPSL3266	hypothetical protein
BPSL3267	hypothetical protein
BPSL3268	putative membrane protein
BPSL3269	hypothetical protein
BPSL3270	putative plasmid replication protein
BPSL3331	putative outer membrane protein
BPSL3342	putative bacteriophage protein
BPSL3344	putative bacteriophage integrase
BPSL3346	hypothetical protein
BPSL3347	putative bacteriophage-related lipoprotein
BPSL3348	putative bacteriophage protein
BPSL3349	putative membrane protein
BPSL3350	putative membrane protein
BPSL3351	putative bacteriophage coat protein
BPSL3370	putative membrane protein
BPSL3381	putative oxidoreductase (partial)
BPSS0003	hypothetical protein
BPSS0062	putative C4-dicarboxylate transport sensor kinase
BPSS0068	hypothetical protein
BPSS0069	transposase (pseudogene)
BPSS0070	putative transposase
BPSS0072	IS element hypothetical protein (pseudogene)
BPSS0072A	conserved hypothetical protein (partial)
BPSS0075	putative ABC transport system, membrane protein
BPSS0076	putative ABC transport system, membrane protein
BPSS0077	putative ABC transport system, exported substrate-binding protein
BPSS0085	putative membrane protein
BPSS0086	putative cytochrome C oxidase-related protein
BPSS0087	putative membrane protein
BPSS0088	putative exported protein
BPSS0090	putative exported protein
BPSS0091	putative fimbrial protein
BPSS0095	putative membrane protein
BPSS0115	hypothetical protein
BPSS0118	hypothetical protein
BPSS0120	putative fimbrial usher protein
BPSS0121	putative fimbrial chaperone
BPSS0122	putative exported protein
BPSS0123	putative exported protein
BPSS0134	putative LysR-family transcriptional regulator
BPSS0135	conserved hypothetical protein
BPSS0136	putative membrane protein
BPSS0137	putative exported protein

BPSS0138	putative membrane protein
BPSS0139	putative pyridine nucleotide-disulphide oxidoreductase
BPSS0167	putative membrane protein
BPSS0174	putative chaperone
BPSS0179	conserved hypothetical protein
BPSS0180	conserved hypothetical protein
BPSS0220	putative membrane protein
BPSS0233	LysR-family regulatory protein (partial)
BPSS0256	putative ABC ribose transport system, permease protein
BPSS0283	putative membrane protein
BPSS0285A	putative outer membrane porin precursor (partial)
BPSS0306	putative multifunctional polyketide-peptide syntase
BPSS0326	putative transport-related membrane protein
BPSS0379	hypothetical protein
BPSS0380A	hypothetical protein
BPSS0380B	hypothetical protein
BPSS0384A	hypothetical protein
BPSS0385	hypothetical protein
BPSS0392	IS element hypothetical protein (partial)
BPSS0393	putative bacteriophage-related protein (partial)
BPSS0395	hypothetical protein
BPSS0397	bacteriophage protein Gp48
BPSS0397A	hypothetical protein
BPSS0398	hypothetical protein
BPSS0399	bacteriophage protein Gp46
BPSS0400	putative bacteriophage protein
BPSS0402	bacteriophage/transposase fusion protein
BPSS0404	putative methylamine utilization protein
BPSS0405	glycerophosphoryl diester phosphodiesterase family protein
BPSS0406	hypothetical protein
BPSS0407	insertion element hypothetical protein
BPSS0412	hypothetical protein
BPSS0413	hypothetical protein
BPSS0416A	hypothetical protein
BPSS0425	putative heptosyltransferase (O-antigen related)
BPSS0430	conserved hypothetical protein (pseudogene)
BPSS0433	putative membrane protein
BPSS0434	hypothetical protein
BPSS0435	putative AraC-family transcriptional regulator
BPSS0436	putative AraC-family regulatory protein
BPSS0437	putative transporter protein
BPSS0438	putative LysR-family transcriptional regulator
BPSS0439	conserved hypothetical protein
BPSS0440	putative membrane protein
BPSS0441	conserved hypothetical protein
BPSS0442	hypothetical protein
BPSS0443	conserved hypothetical protein
BPSS0444	non-heme chloroperoxidase
BPSS0445	putative LysR-family transcriptional regulator (partial)

BPSS0447	conserved hypothetical protein
BPSS0448	hypothetical protein
BPSS0452	putative phosphoesterase
BPSS0453	putative exported protein
BPSS0454	putative exported protein
BPSS0455	putative copper resistance-related lipoprotein
BPSS0458	putative copper-resistance exported protein
BPSS0460	putative methyl-accepting chemotaxis protein
BPSS0470	putative glutamine amidotransferase
BPSS0475	hypothetical protein
BPSS0480	hypothetical protein
BPSS0486	putative non-ribosomal peptide synthetase
BPSS0488A	putative transposase (partial)
BPSS0489	putative exported protein
BPSS0490	putative transposase (partial)
BPSS0586	pyochelin synthetase
BPSS0587	pyochelin synthetase
BPSS0594	putative membrane protein
BPSS0599	hypothetical protein
BPSS0600	putative membrane protein
BPSS0602	hypothetical protein
BPSS0605	putative membrane protein
BPSS0606	LysR family regulatory protein
BPSS0607	aldehyde dehydrogenase family protein
BPSS0608	TauD/TfdA family dioxygenase
BPSS0609	thiamine pyrophosphate enzyme family protein
BPSS0610	putative phosphoenolpyruvate phosphomutase
BPSS0629	putative exported protein
BPSS0637	conserved hypothetical protein (pseudogene)
BPSS0652a	hypothetical protein
BPSS0653	transposase (partial)
BPSS0654	putative X-Pro dipeptidyl-peptidase
BPSS0655	conserved hypothetical protein
BPSS0656	hypothetical protein
BPSS0657	transposase (partial)
BPSS0658	putative exported protein
BPSS0659	conserved hypothetical protein
BPSS0660	insertion sequence protein (partial)
BPSS0661	putative insertion element protein (pseudogene)
BPSS0663	putative exported protein
BPSS0664	response regulator protein
BPSS0664a	putative response regulator protein (partial)
BPSS0665	putative alpha-ketoglutarate-dependent taurine dioxygenase
BPSS0666	putative collagenase
BPSS0667	acetyltransferase (GNAT) family protein (partial)
BPSS0686a	hypothetical protein
BPSS0712	conserved hypothetical protein
BPSS0715	putative exported protein
BPSS0716	hypothetical protein

BPSS0721a	putative acetyltransferase (partial)
BPSS0732	hypothetical protein
BPSS0737	putative membrane protein
BPSS0744	insertion element protein (partial)
BPSS0745	hypothetical protein
BPSS0746	putative lipase
BPSS0768	hypothetical protein
BPSS0773	LysR family regulatory protein (partial)
BPSS0781	putative AMP-binding enzyme
BPSS0796	putative surface-exposed protein
BPSS0812	hypothetical protein
BPSS0818	hypothetical protein
BPSS0820	putative tRNA synthetase
BPSS0850	conserved hypothetical protein
BPSS0851	putative nitrilotriacetate monooxygenase component A
BPSS0854	putative exported protein
BPSS0857	hypothetical protein
BPSS0858	N-carbamoyl-L-amino acid hydrolase (partial)
BPSS0874	hypothetical protein
BPSS0920	LysR family regulatory protein
BPSS0921	putative peptidase
BPSS0922	putative transporter protein
BPSS0991	response regulator protein (pseudogene)
BPSS0997	conserved hypothetical protein
BPSS1002	putative hydroxymethylglutaryl-coenzyme A synthase
BPSS1006	putative polyketide synthase
BPSS1007	putative polyketide synthase
BPSS1008	putative polyketide synthase
BPSS1017	putative antibiotic resistance protein
BPSS1028	putative iron uptake regulator protein
BPSS1031	putative transmembrane sugar transport protein
BPSS1032	putative sugar ABC transporter ATP-binding component
BPSS1033	putative ABC periplasmic-binding sugar transport protein
BPSS1035	hypothetical protein
BPSS1037	putative sugar transport protein
BPSS1049	hypothetical bacteriophage protein
BPSS1050	hypothetical bacteriophage protein
BPSS1060	hypothetical bacteriophage protein
BPSS1063	putative bacteriophage terminase, ATPase subunit
BPSS1092	conserved hypothetical protein
BPSS1093	putative hydratase protein
BPSS1097	putative capsule biosynthesis protein
BPSS1098	conserved hypothetical protein
BPSS1101	putative exported protein
BPSS1104	putative phosphomethylpyrimidine kinase
BPSS1108	putative NUDIX/MutT family protein
BPSS1109	putative methyltransferase
BPSS1110	putative gamma-butyrobetaine,2-oxoglutarate dioxygenase
BPSS1111	putative membrane protein

BPSS1112	putative amino acid permease
BPSS1135	putative hydroxyethylthioazole kinase
BPSS1136	putative exported protein
BPSS1137	putative membrane protein
BPSS1141	hypothetical protein
BPSS1143	putative formate hydrogenlyase/hydrogenase/NADH dehydrogenase subunit
BPSS1153	hypothetical protein
BPSS1166	putative transport/efflux protein
BPSS1170	putative non-ribosomal peptide synthase/polyketide synthase
BPSS1174	putative non-ribosomal peptide/polyketide synthase
BPSS1177	hypothetical protein
BPSS1179	hypothetical protein
BPSS1185	putative shikimate transporter (partial)
BPSS1185a	hypothetical protein
BPSS1187	hypothetical protein
BPSS1188	putative non ribosomal peptide/polyketide synthase (pseudogene)
BPSS1194	putative peptide synthase/polyketide synthase
BPSS1195	putative non-ribosomal peptide synthase
BPSS1197	putative non-ribosomal peptide synthase
BPSS1202	conserved hypothetical protein
BPSS1208	putative transposase (partial)
BPSS1209	putative transposase
BPSS1214	putative transcriptional regulator
BPSS1215	putative sugar transporter
BPSS1216	putative thiamine-phosphate pyrophosphorylase
BPSS1226	hypothetical protein
BPSS1255	putative LysR family transcriptional regulator
BPSS1265	conserved hypothetical protein (pseudogene)
BPSS1269	putative peptide synthase/polyketide synthase
BPSS1293	putative lipoprotein
BPSS1314	hypothetical protein
BPSS1319	conserved hypothetical protein
BPSS1371	putative AraC family transcriptional regulator
BPSS1372	conserved hypothetical protein
BPSS1374	putative glutathione-S-transferase
BPSS1375	putative membrane protein
BPSS1376	putative quinol oxidase subunit
BPSS1377	putative ubiquinol cytochrome c cyanide insensitive terminal oxidase
BPSS1378	putative membrane protein
BPSS1379	putative transmembrane transporter protein
BPSS1380	hypothetical protein
BPSS1381	putative transmembrane phospholipase protein
BPSS1382	putative Endonuclease/Exonuclease/phosphatase family protein
BPSS1383	hypothetical protein
BPSS1383a	putative rotamase
BPSS1384	putative membrane protein
BPSS1384a	transposase (partial)
BPSS1385	putative ATP/GTP binding protein
BPSS1386	putative ATP/GTP binding protein

BPSS1387	conserved hypothetical protein
BPSS1388	putative membrane protein
BPSS1389	hypothetical protein
BPSS1390	putative type III secretion system protein
BPSS1391	putative AraC family regulator of pathogenicity genes
BPSS1392	putative type III secretion-associated protein
BPSS1393	hypothetical protein
BPSS1394	putative type III secretion associated protein
BPSS1395	putative type III secretion-associated protein
BPSS1396	hypothetical protein
BPSS1397	putative type III secretion associated protein
BPSS1398	hypothetical protein
BPSS1399	hypothetical protein
BPSS1400	putative type III secretion-associated protein
BPSS1401	putative type III secretion-associated protein
BPSS1402	hypothetical protein
BPSS1403	putative type III secretion-associated protein
BPSS1404	putative type III secretion-associated protein
BPSS1405	putative type III secretion-associated protein
BPSS1406	hypothetical protein
BPSS1407	secretion-associated protein
BPSS1408	hypothetical protein
BPSS1409	hypothetical protein
BPSS1410	conserved hypothetical protein
BPSS1422	putative membrane protein
BPSS1434	putative membrane protein
BPSS1439	putative cell surface protein
BPSS1450	putative nitric oxide reductase (pseudogene)
BPSS1464	putative transmembrane NADPH flavoprotein
BPSS1465	putative thiamine biosynthesis lipoprotein precursor
BPSS1475	putative transmembrane sugar transporter
BPSS1491	conserved hypothetical protein
BPSS1492	hypothetical protein
BPSS1493	hypothetical protein
BPSS1511	putative membrane protein
BPSS1516	hypothetical protein
BPSS1518	putative transposase
BPSS1519	transposase
BPSS1528	hypothetical protein
BPSS1539	hypothetical protein
BPSS1580	putative cellulose biosynthesis protein
BPSS1582a	hypothetical protein
BPSS1583	putative transcription regulator
BPSS1590	hypothetical protein
BPSS1603	putative secretion protein
BPSS1609	conserved hypothetical protein
BPSS1632	probable non-ribosomal peptide synthetase
BPSS1633	probable non-ribosomal peptide synthetase
BPSS1634	probable non-ribosomal peptide synthetase

BPSS1702	hypothetical protein
BPSS1703	putative membrane protein
BPSS1729	putative cytochrome C
BPSS1730	putative membrane protein
BPSS1732	putative membrane protein
BPSS1774A	putative phage-related tail protein (partial)
BPSS1776	putative integrase (partial)
BPSS1824	putative AraC-family transcriptional regulator
BPSS1869	putative dehalogenase
BPSS1882	putative acetyltransferase
BPSS1929	putative outer membrane lipoprotein
BPSS1951	putative ATP synthesis-related protein
BPSS1964	conserved hypothetical protein
BPSS1980	putative membrane protein
BPSS1998	putative lipoprotein
BPSS1999	putative transport related, membrane protein
BPSS2000	putative exported protein
BPSS2001	hypothetical protein
BPSS2002	putative periplasmic amino acid-binding protein
BPSS2004	putative IS element protein (partial)
BPSS2005	putative transposase
BPSS2006	hypothetical protein
BPSS2007	conserved hypothetical protein
BPSS2008	putative transposase (partial)
BPSS2013	putative outer membrane protein
BPSS2019	conserved hypothetical protein
BPSS2020	conserved hypothetical protein
BPSS2023	hypothetical protein
BPSS2024	putative zinc-containing alcohol dehydrogenase
BPSS2025	putative decarboxylase
BPSS2027	putative exported protein
BPSS2046	putative IS element transposase
BPSS2047	putative chrolohydrolase
BPSS2048	putative glutathione S-transferase
BPSS2049	putative IS element transposase
BPSS2050	hypothetical protein (partial)
BPSS2051	putative DNA-binding protein
BPSS2052	putative activator/secretion protein
BPSS2053	putative cell surface protein
BPSS2054	conserved hypothetical protein
BPSS2055	conserved hypothetical protein
BPSS2056	conserved hypothetical protein
BPSS2058	putative ATP-binding inner membrane transport protein
BPSS2059	conserved hypothetical protein
BPSS2060	L-asparaginase
BPSS2061	conserved hypothetical protein
BPSS2061A	putative transposase (partial)
BPSS2062	acetyltransferase (GNAT) family protein
BPSS2063	hypothetical protein

BPSS2064	putative porin protein
BPSS2065	putative fatty aldehyde dehydrogenase
BPSS2066	conserved hypothetical protein
BPSS2067	putative aldose 1-epimerase
BPSS2068	short chain dehydrogenase
BPSS2069	ABC transporter, ATP-binding protein
BPSS2070	branched-chain amino acid transport system permease
BPSS2071	putative exported protein
BPSS2072	mandelate racemase / muconate lactonizing enzyme
BPSS2073	GntR family regulator protein
BPSS2074	senescence marker protein-30 (SMP-30) family protein
BPSS2074a	conserved hypothetical protein (partial)
BPSS2075	conserved hypothetical protein
BPSS2076	transposase IS66 family protein (pseudogene)
BPSS2079	putative DNA-binding protein
BPSS2080	hypothetical protein
BPSS2081	putative alpha-galactosidase
BPSS2082	putative ABC transporter system permease
BPSS2083	putative ABC transporter, permease protein
BPSS2084	putative extracellular solute-binding protein
BPSS2085	putative ABC transport system, ATP-binding protein
BPSS2086	hypothetical protein
BPSS2087	LacI family regulatory protein
BPSS2088	transposase (partial)
BPSS2089	putative exported protein
BPSS2090	putative DNA-binding protein
BPSS2091	hypothetical protein
BPSS2092	hypothetical protein (partial)
BPSS2103	hypothetical protein
BPSS2111	putative FAD-dependent monooxygenase
BPSS2148	putative IS element protein
BPSS2148a	transposase
BPSS2166	hypothetical protein
BPSS2178	conserved hypothetical protein
BPSS2179	putative membrane protein
BPSS2208	hypothetical protein
BPSS2209	hypothetical protein
BPSS2210	putative cation transport related protein
BPSS2211	putative ATP-dependent DNA ligase
BPSS2212	conserved hypothetical protein
BPSS2213	putative membrane protein
BPSS2214	catalase HPII
BPSS2215	hypothetical protein
BPSS2216	putative membrane protein
BPSS2217	conserved hypothetical protein
BPSS2218	RNA polymerase sigma-54 factor
BPSS2219	putative lipoprotein
BPSS2220	conserved hypothetical protein
BPSS2221	conserved hypothetical protein

BPSS2222	hypothetical protein
BPSS2223	putative exported protein
BPSS2224	putative lipoprotein
BPSS2225	hypothetical protein
BPSS2226	hypothetical protein
BPSS2227	hypothetical protein
BPSS2227A	conserved hypothetical protein
BPSS2228	hypothetical protein
BPSS2229	conserved hypothetical protein
BPSS2230	conserved hypothetical protein
BPSS2231	putative response regulator
BPSS2232	putative exported protein
BPSS2233	putative DNA glycosylase
BPSS2234	putative transport related, membrane protein
BPSS2235	putative Zinc-binding dehydrogenase
BPSS2240	hypothetical protein
BPSS2243	putative lipoprotein
BPSS2251	putative LPS biosynthesis related transferase
BPSS2259	putative fusion protein, ATP-binding transmembrane ABC transporter and regulatory protein
BPSS2297	conserved hypothetical protein
BPSS2298	conserved hypothetical protein
BPSS2328	putative multi-domain beta keto-acyl synthase

Segment 6- Conserved in Bp and Bt but not as well or absent in Bm

Gene	Description	to Bt % length	to Bt % identity
BPSL0006	putative exported protein	100	91
BPSL0020	putative membrane protein	100	87
BPSL0030	flagellar biosynthetic protein	100	98
BPSL0057	putative membrane protein	100	91
BPSL0058	fatty acid desaturase	100	95
BPSL0059	hypothetical protein	100	97
BPSL0060	AraC family transcriptional regulator	100	92
BPSL0061	putative acyl-CoA dehydrogenase	100	97
BPSL0062	beta-ketoadipyl CoA thiolase	100	97
BPSL0063	putative fatty oxidation complex alpha subunit	100	96
BPSL0064	alpha-methylacyl-CoA racemase	100	97
BPSL0065	putative lipoprotein	100	91
BPSL0067	hypothetical protein	100	97
BPSL0069	putative transmembrane regulator	99	90
BPSL0070	putative RNA polymerase sigma factor	100	99
BPSL0071	putative catalase	100	95
BPSL0072	putative type-b cytochrome	100	94
BPSL0082	hypothetical protein	100	92
BPSL0093	putative lipoprotein	100	93
BPSL0094	putative lipoprotein	100	96
BPSL0096	spermidine n(1)-acetyltransferase	100	94
BPSL0097	putative exported protein	100	94
BPSL0144	putative phage protein	100	97

BPSL0145	putative phage protein	100	98
BPSL0147	putative phage protein	100	97
BPSL0148	putative phage protein	100	100
BPSL0149	phage major tail tube protein	100	95
BPSL0150	phage major tail sheath protein	100	99
BPSL0151	putative phage tail fiber assembly protein	100	98
BPSL0152	phage-related tail fiber protein	100	99
BPSL0153	putative phage protein	100	98
BPSL0154	phage baseplate assembly protein	100	99
BPSL0155	phage baseplate assembly protein	100	99
BPSL0156	phage baseplate assembly protein	100	99
BPSL0157	phage-encoded modification methylase	98	94
BPSL0159	phage tail completion protein	100	94
BPSL0160	phage tail completion protein	100	97
BPSL0161	putative phage protein	100	94
BPSL0162	putative phage-encoded lipoprotein	100	91
BPSL0163	putative phage-encoded peptidoglycan binding protein	100	97
BPSL0164	putative phage-encoded membrane protein	100	98
BPSL0165	putative phage-encoded membrane protein	100	100
BPSL0166	phage tail protein	100	100
BPSL0167	hypothetical phage protein	100	98
BPSL0168	phage head completion/stabilization protein	100	91
BPSL0169	phage terminase, endonuclease subunit	100	100
BPSL0170	phage major capsid protein precursor	100	97
BPSL0171	putative phage capsid scaffolding protein	100	99
BPSL0172	phage terminase, ATPase subunit	97	98
BPSL0223	putative acyl-CoA dehydrogenase	100	94
BPSL0235	putative export system protein	100	93
BPSL0240	sensor kinase protein (partial)	100	88
BPSL0251	putative dipeptide transport system permease protein	100	97
BPSL0283	LysR family regulatory protein	100	95
BPSL0288	putative membrane protein	100	91
BPSL0293	putative glucosyltransferase	99	93
BPSL0326	putative outer membrane porin protein precursor	100	94
BPSL0327	LysR family regulatory protein	100	98
BPSL0328	putative 2-nitropropane dioxygenase	100	98
BPSL0329	putative hydrolase	100	97
BPSL0330	AsnC family regulatory protein	100	92
BPSL0331	putative membrane protein	100	93
BPSL0332	conserved hypothetical protein	100	95
BPSL0333	putative hydroxymethylglutaryl-CoA lyase	100	94
BPSL0334	conserved hypothetical protein	100	92
BPSL0335	glyoxalase/bleomycin resistance protein/dioxygenase superfamily protein	100	96
BPSL0336	conserved hypothetical protein	99	87
BPSL0338	non-hemolytic phospholipase C precursor	100	95
BPSL0340	AsnC family regulatory protein	100	94
BPSL0341	putative transport protein	100	94
BPSL0342	putative membrane protein	100	92
BPSL0346	dihydrodipicolinate synthetase family protein	99	89

BPSL0352	sulfate-binding protein precursor	100	95
BPSL0353	putative exported protein	100	93
BPSL0354	putative exported protein	100	87
BPSL0355	putative inward rectifier potassium channel protein	100	88
BPSL0356	conserved hypothetical protein	100	95
BPSL0357	sodium/hydrogen exchanger family protein	100	97
BPSL0358	hypothetical protein	100	95
BPSL0368	putative exported protein	100	94
BPSL0426	C4-dicarboxylate transport sensor protein	100	94
BPSL0435	ammonium transporter family protein	100	94
BPSL0524	putative lipoprotein	99	87
BPSL0540	putative adenine phosphoribosyltransferase	100	95
BPSL0541	LysE type translocator	100	98
BPSL0547	single-strand binding protein	100	96
BPSL0578	dienelactone hydrolase family protein	100	88
BPSL0579	hypothetical protein	100	91
BPSL0580	hypothetical protein	100	91
BPSL0581	conserved hypothetical protein	100	99
BPSL0589	conserved hypothetical protein	100	95
BPSL0590	putative membrane protein	100	93
BPSL0594	hypothetical protein	99	95
BPSL0630	D-aminoacylase	100	94
BPSL0662	putative membrane protein	100	94
BPSL0707	conserved hypothetical protein	100	91
BPSL0710	putative ATP/GTP binding protein	100	93
BPSL0712	putative ATP-binding transmembrane ABC transporter	100	87
BPSL0713	putative transmembrane transporter	100	89
BPSL0716	conserved hypothetical protein	100	93
BPSL0717	conserved hypothetical protein	99	90
BPSL0719	putative membrane protein	100	91
BPSL0732	putative two-component regulator histidine sensor kinase	100	90
BPSL0733	putative exported protein	100	92
BPSL0734	putative two-component transcriptional response regulator	100	94
BPSL0774	two-component sensor kinase transcriptional regulatory protein	100	94
BPSL0786	molybdenum cofactor biosynthesis protein c	100	95
BPSL0787	putative exported protein	100	93
BPSL0817	putative permease protein	100	96
BPSL0818	putative sugar kinase protein	100	92
BPSL0856	putative thioredoxin protein	100	95
BPSL0873	conserved hypothetical protein	100	97
BPSL0878	putative tetraacyldisaccharide 4'-kinase	100	98
BPSL0881	hypothetical protein	100	90
BPSL0900	putative amino acid permease	100	96
BPSL1034	conserved hypothetical protein	100	93
BPSL1036	putative two-component system, response regulator	100	98
BPSL1042	putative lipoprotein	100	94
BPSL1043	putative lipoprotein	100	93
BPSL1123	putative LPS core biosynthesis-related protein	100	89
BPSL1174	two-component system, sensor kinase protein KdpD	100	91

BPSL1179	putative membrane protein	100	93
BPSL1201	2-isopropylmalate synthase	100	99
BPSL1247	putative aminopeptidase	99	90
BPSL1248	putative membrane protein	100	95
BPSL1249	putative AraC-family transcriptional regulator	100	95
BPSL1250	D-3-phosphoglycerate dehydrogenase	100	94
BPSL1251	putative transposase	100	88
BPSL1253	putative integral membrane protein/sensor kinase	100	95
BPSL1254	hypothetical protein	100	92
BPSL1256	putative cytochrome c precursor	100	96
BPSL1257	putative cytochrome c precursor	100	96
BPSL1258	putative membrane protein	100	100
BPSL1259	putative cytochrome c oxidase subunit II related protein	100	97
BPSL1260	cytochrome c oxidase subunit 1	100	97
BPSL1261	putative cytochrome c related protein	100	94
BPSL1262	conserved hypothetical protein	100	96
BPSL1263	conserved hypothetical protein	100	98
BPSL1264	AhpC/TSA family membrane protein	99	96
BPSL1265	conserved hypothetical protein	100	94
BPSL1266	putative transport system, membrane protein	100	97
BPSL1267	putative transport system, membrane protein	100	97
BPSL1268	putative transport system, membrane protein	100	92
BPSL1269	putative IclR-family transcriptional regulatory protein	100	97
BPSL1286	conserved hypothetical protein	100	91
BPSL1291	putative exported protein	100	88
BPSL1338	putative glutathione S-transferase related protein	100	95
BPSL1382	conserved hypothetical protein	100	89
BPSL1383	putative undecaprenol kinase	100	97
BPSL1388	hypothetical protein	100	95
BPSL1389	putative phage related protein (partial)	100	96
BPSL1427	hypothetical protein	99	93
BPSL1440	putative exported protein	100	95
BPSL1449	conserved hypothetical protein	100	98
BPSL1450	2-hydroxy-3-oxopropionate reductase	100	94
BPSL1451	hydroxypyruvate isomerase	100	92
BPSL1452	glyoxylate carboligase	100	96
BPSL1453	putative LysR-family transcriptional regulator	100	98
BPSL1476	putative aminotransferase	100	97
BPSL1559	putative membrane protein	100	95
BPSL1560	hypothetical protein	100	94
BPSL1561	putative metallo-beta-lactamase family protein	100	95
BPSL1562	putative transcriptional regulatory protein	99	93
BPSL1563	putative membrane protein	100	93
BPSL1564	putative transcriptional regulatory protein	99	94
BPSL1618	hypothetical protein	100	92
BPSL1638	putative phage-related protein (partial)	99	93
BPSL1767	putative magnesium chelatase protein	100	88
BPSL1768	putative cobalamin biosynthesis-related protein	100	95
BPSL1797	putative ABC transport system, membrane protein	100	89

BPSL1798	putative exported protein	99	91
BPSL1802	multidrug efflux system putative membrane lipoprotein	100	90
BPSL1803	multidrug efflux system transporter protein AmrB	100	98
BPSL1804	multidrug efflux system putative membrane fusion protein	100	95
BPSL1805	TetR family regulatory protein	99	93
BPSL1806	hypothetical protein	100	86
BPSL1807	putative amino acid transport system, membrane protein	100	92
BPSL1808	putative amino acid transport system, membrane protein	100	88
BPSL1809	putative amino acid transport system, exported protein	100	94
BPSL1810	putative membrane protein	100	91
BPSL1811	putative membrane protein	100	92
BPSL1813	putative fimbriae-related membrane protein	100	94
BPSL1814	putative membrane protein	100	90
BPSL1815	putative fimbriae assembly-related protein	100	91
BPSL1816	putative fimbriae assembly-related protein	100	93
BPSL1817	putative lipoprotein	100	92
BPSL1818	putative fimbriae assembly-related protein	100	97
BPSL1819	putative fimbriae-assembly related protein	100	90
BPSL1820	putative fimbriae assembly-related protein	100	88
BPSL1821	putative fimbriae assembly-related protein	100	96
BPSL1825	putative ABC transport system, permease protein	100	97
BPSL1826	conserved hypothetical protein	100	98
BPSL1827	conserved hypothetical protein	100	98
BPSL1828	conserved hypothetical protein	100	99
BPSL1830	putative ribokinase	100	92
BPSL1831	putative ribose operon repressor	100	99
BPSL1832	putative ribose transport system, permease protein	100	97
BPSL1833	putative ribose transport system, ATP-binding protein	100	94
BPSL1834	putative ribose transport system, substrate-binding protein	100	98
BPSL1885A	Hfq protein	100	90
BPSL1892	putative fimbriae-related outer membrane protein	100	93
BPSL1893	putative type II/IV secretion system ATP-binding protein	100	97
BPSL1899	putative fimbriae assembly related protein	100	98
BPSL1900	hypothetical protein	100	91
BPSL1901	putative membrane protein	100	90
BPSL1936	putative membrane protein	99	87
BPSL1963	putative membrane protein	100	96
BPSL2015	putative membrane attached glycosyl hydrolase	100	93
BPSL2027	putative fimbriae-related protein	100	90
BPSL2041	conserved hypothetical protein	98	94
BPSL2045	putative lipoprotein	100	98
BPSL2046	conserved hypothetical protein	100	96
BPSL2047	conserved hypothetical protein	100	95
BPSL2048A	conserved hypothetical protein	98	86
BPSL2086	hypothetical protein	100	95
BPSL2087	conserved hypothetical protein (partial)	100	97
BPSL2109	putative glyoxylate-realted oxidoreductase	100	94
BPSL2110	conserved hypothetical protein	100	95
BPSL2111	putative LysR-family transcriptonal regulator	100	99

BPSL2112	putative amino hydrolase	100	93
BPSL2113	putative purine catabolism-related protein	100	94
BPSL2114	putative membrane protein	100	96
BPSL2229	putative siderophore non-ribosomal peptide synthase	100	86
BPSL2254	putative FkbP-type peptidyl-prolyl cis-trans isomerase	100	98
BPSL2268	putative lipoprotein	100	89
BPSL2300	dihydrolipoamide acetyltransferase component of pyruvate dehydrogenase complex	100	95
BPSL2335	aminotransferase class-III	100	92
BPSL2369	conserved hypothetical protein	100	97
BPSL2370	probable ATP-dependent transporter protein	100	93
BPSL2377	conserved hypothetical protein	99	88
BPSL2379	ubiquinol oxidase polypeptide I	100	95
BPSL2380	cytochrome o ubiquinol oxidase subunit III	100	97
BPSL2394	histidine transport system permease protein	100	95
BPSL2395	putative lipoprotein	100	96
BPSL2407	putative membrane protein	100	86
BPSL2450	haloacid dehalogenase-like hydrolase	100	97
BPSL2498	putative acyl-CoA dehydrogenase	100	97
BPSL2556	possible DNA polymerase/helicase	100	91
BPSL2557	cold shock-like protein	100	100
BPSL2598	putative acetyltransferase protein	100	98
BPSL2653	putative branched-chain amino acid transport system, permease component	100	96
BPSL2660	urease accessory protein	100	91
BPSL2722	membrane transport solute-binding protein	100	95
BPSL2741	conserved hypothetical protein	100	98
BPSL2769	putative UTP-glucose-1-phosphate uridylyltransferase	100	96
BPSL2770	putative capsule expression protein	100	91
BPSL2771	putative haloacid dehalogenase-like hydrolase protein	100	92
BPSL2772	putative 2-dehydro-3-deoxyphosphooctonate aldolase	100	92
BPSL2774	hypothetical protein	100	88
BPSL2775	putative colanic acid biosynthesis acetyltransferase	100	92
BPSL2776	hypothetical protein	100	88
BPSL2778	putative tyrosine-protein kinase involved in EPS biosynthesis	100	92
BPSL2779	putative protein-tyrosine-phosphatase (low molecular weight)	100	92
BPSL2780	putative capsular polysaccharide transport protein	100	91
BPSL2781	putative transmembrane sugar transferase	100	96
BPSL2782	putative AraC family transcriptional regulator	100	93
BPSL2783	putative membrane protein	100	86
BPSL2785	putative exported protein	100	93
BPSL2881	putative membrane protein	100	97
BPSL2924	glutamate/aspartate periplasmic binding protein precursor	100	99
BPSL2939	putative transport-related membrane protein	98	86
BPSL2944	putative ureidoglycolate hydrolase	100	96
BPSL2990	histone H1-like protein	100	92
BPSL3037	long-chain-fatty-acid--CoA ligase	100	98
BPSL3038	putative molybdopterin-containing oxidoreductase	100	93
BPSL3044	phenylacetic acid degradation protein PaaI	100	93
BPSL3105	conserved hypothetical protein	100	98
BPSL3106	conserved hypothetical protein	100	99

BPSL3107	conserved hypothetical protein	100	98
BPSL3108	putative lipoprotein	100	91
BPSL3109	putative lipoprotein	100	98
BPSL3110	conserved hypothetical protein	100	98
BPSL3111	putative membrane protein	100	98
BPSL3237	conserved hypothetical protein	100	96
BPSL3250	putative LysR-family transcriptional regulator	100	95
BPSL3251	putative inner membrane transport protein	100	91
BPSL3252	putative amino acid transport protein	100	89
BPSL3253	putative oxidoreductase	100	95
BPSL3254	conserved hypothetical protein	99	93
BPSL3254A	putative membrane protein	100	90
BPSL3254B	putative exported protein	100	88
BPSL3285	putative oxidoreductase	100	90
BPSL3293	flagellar biosynthesis protein FlhF	100	94
BPSL3297	Gly/Ala/Ser-rich lipoprotein	100	91
BPSL3306	chemotaxis two-component sensor kinase CheA	100	92
BPSL3308	chemotaxis protein MotB	100	95
BPSL3343	putative bacteriophage protein	100	98
BPSL3345	putative bacteriophage-related protein	100	98
BPSL3352	hypothetical protein	100	97
BPSL3353	putative DNA-binding protein	100	97
BPSL3372	ethanolamine ammonia-lyase heavy chain	100	97
BPSL3429	putative NADH-dependent flavin oxidoreductase	100	94
BPSS0064	putative membrane protein	99	86
BPSS0073	putative regulatory protein	100	97
BPSS0074	hypothetical protein	99	96
BPSS0077A	putative HNS-like regulatory protein	100	95
BPSS0078	conserved hypothetical protein	100	94
BPSS0079	conserved hypothetical protein	100	91
BPSS0080	conserved hypothetical protein	100	97
BPSS0080a	conserved hypothetical protein	100	95
BPSS0081	conserved hypothetical protein	100	100
BPSS0082	putative thioredoxin	100	99
BPSS0083	putative exported protein	100	98
BPSS0084	putative porin-related protein (partial)	100	100
BPSS0089	putative exported protein	100	88
BPSS0092	putative fimbria-related chaperone	100	91
BPSS0093	outer membrane usher protein	100	92
BPSS0094	putative fimbria subunit protein	100	86
BPSS0096	OmpA family membrane protein	100	86
BPSS0097	conserved hypothetical protein	100	98
BPSS0098	conserved hypothetical protein	100	99
BPSS0099	conserved hypothetical protein	100	96
BPSS0100	putative exported protein	100	94
BPSS0101	conserved hypothetical protein	100	99
BPSS0102	OmpA family membrane protein	100	95
BPSS0103	putative membrane protein	100	93
BPSS0104	hypothetical protein	100	94

BPSS0105	conserved hypothetical protein	98	98
BPSS0106	putative Rhs-like protein (pseudogene)	100	92
BPSS0108	conserved hypothetical protein	100	91
BPSS0109	putative exported protein	100	90
BPSS0110	conserved hypothetical protein	100	90
BPSS0111	conserved hypothetical protein	100	95
BPSS0112	conserved hypothetical protein	100	99
BPSS0113	conserved hypothetical protein	99	94
BPSS0114	conserved hypothetical protein	100	92
BPSS0116	putative chaperone-related protein	100	95
BPSS0117	putative histidine kinase/response regulator fusion protein	100	89
BPSS0119	putative two-component system response regulator	100	90
BPSS0124	putative response regulator	100	86
BPSS0125	putative regulatory protein	99	91
BPSS0126	putative transport system, membrane protein	100	94
BPSS0127	glycerate kinase 1	100	93
BPSS0128	pyruvate kinase	100	95
BPSS0129	putative transcriptional regulator	100	93
BPSS0130	putative peptide synthase protein	100	93
BPSS0131	putative dehydratase	100	92
BPSS0132	conserved hypothetical protein	100	94
BPSS0133	putative methyltransferase	100	90
BPSS0140	putative sugar ABC transport system, lipoprotein	100	94
BPSS0141	putative sugar ABC transport system, membrane protein	100	95
BPSS0142	putative sugar ABC transport system, ATP-binding protein	100	94
BPSS0143	putative ROK family transcriptional regulator	100	95
BPSS0144	putative amylase	100	94
BPSS0147	putative transporter protein	100	96
BPSS0216	putative membrane protein	100	88
BPSS0224	putative cation-transporting ATPase membrane protein	100	88
BPSS0265	putative porin-related protein	100	97
BPSS0282	putative GntR-family regulatory protein	100	91
BPSS0305	putative ketol-acid reductoisomerase	100	94
BPSS0330	putative amino-acid permease membrane protein	100	95
BPSS0338	putative insertion element protein	100	92
BPSS0351	putative copper-related MerR-family transcriptional regulator	100	92
BPSS0374	putative membrane protein	100	96
BPSS0376	putative membrane protein	100	91
BPSS0378	putative phage integrase	100	98
BPSS0380	putative DNA-binding regulatory protein	100	96
BPSS0381	putative DNA-binding regulatory protein	100	100
BPSS0382	hypothetical protein	100	94
BPSS0383	putative DNA-binding protein	100	100
BPSS0384	hypothetical protein	99	94
BPSS0386	transposon Tn2501 resolvase	100	99
BPSS0387	putative phage-related protein	100	99
BPSS0388	putative phage-related protein	100	99
BPSS0389	putative phage-related protein	100	96
BPSS0390	conserved hypothetical protein	100	100

BPSS0391	putative phage-related hypothetical protein	100	99
BPSS0391A	hypothetical protein	100	95
BPSS0394	conserved hypothetical protein	100	98
BPSS0396	bacteriophage protein Gp49	100	89
BPSS0401	bacteriophage protein Gp44	100	87
BPSS0408	putative exported protein	100	95
BPSS0409	hypothetical protein	100	97
BPSS0410	hypothetical protein	100	96
BPSS0411	hypothetical protein	97	93
BPSS0414	putative acetolactate synthase	100	88
BPSS0415	putative lipoprotein	100	87
BPSS0416	putative lipoprotein	100	92
BPSS0417	hypothetical protein	100	91
BPSS0418	putative transport-related membrane protein	100	91
BPSS0419	glucose-1-phosphate cytidylyltransferase (O-antigen-related)	100	98
BPSS0420	CDP-glucose 4,6-dehydratase (O-antigen-related)	100	90
BPSS0421	lipopolysaccharide biosynthesis protein (O-antigen-related)	100	94
BPSS0422	putative aminotransferase	100	93
BPSS0423	putative membrane protein	100	89
BPSS0424	putative glycosyl transferase	97	86
BPSS0426	putative heptosyltransferase (O-antigen related)	100	91
BPSS0427	putative O-acetyl transferase (O-antigen-related)	100	94
BPSS0428	putative glycosyl transferase (O-antigen related)	100	90
BPSS0429	putative membrane protein	100	90
BPSS0431	putative LysR-family transcriptional regulator	100	90
BPSS0432	conserved hypothetical protein	100	95
BPSS0446	conserved hypothetical protein	100	93
BPSS0449	putative GntR-family regulatory protein	100	93
BPSS0450	DJ-1/PfpI family protein	100	91
BPSS0451	penicillin-binding protein	100	87
BPSS0456	putative exported copper oxidase	100	97
BPSS0457	putative exported protein	99	88
BPSS0459	putative copper-resistance membrane protein	100	89
BPSS0461	putative oxidoreductase	100	93
BPSS0462	putative oxidoreductase	100	92
BPSS0463	putative exported protein	100	94
BPSS0464	putrescine transport system permease protein	100	94
BPSS0465	putrescine ABC transport system, permease protein	100	95
BPSS0466	putrescine ABC transport system, ATP-binding protein	100	97
BPSS0467	putrescine ABC transport system, binding exported protein	100	97
BPSS0468	putative aminotransferase	100	94
BPSS0469	putative glutamine synthetase	100	96
BPSS0471	hypothetical protein	100	88
BPSS0472	putative MerR-family transcriptional regulator	100	97
BPSS0473	putative aldehyde dehydrogenase	100	96
BPSS0474	agmatinase	100	93
BPSS0476	10 kDa chaperonin	100	96
BPSS0477	60 kDa chaperonin	100	97
BPSS0478	putative membrane protein	99	93

BPSS0479	putative ribonucleotide reductase protein	100	96
BPSS0481	putative CoA ligase	100	91
BPSS0482	putative exported protein	100	86
BPSS0483	putative beta-ketoacyl-ACP synthase	100	94
BPSS0484	3-oxoacyl-[acyl-carrier-protein] synthase III	100	96
BPSS0485	putative metallo-beta lactamase-related protein	100	89
BPSS0487	hypothetical protein	100	92
BPSS0488	putative membrane protein	100	88
BPSS0491	alkyl hydroperoxide reductase subunit	100	90
BPSS0516	conserved hypothetical protein	100	97
BPSS0561	conserved hypothetical protein	100	93
BPSS0572	putative short-chain dehydrogenase	100	93
BPSS0573	hypothetical protein	100	95
BPSS0574	putative FAD dependent oxidoreductase	100	93
BPSS0575	leucine-, isoleucine-, valine-, threonine-, and alanine-binding protein precursor	100	97
BPSS0576	high-affinity branched-chain amino acid transport system permease protein	100	97
BPSS0577	high-affinity branched-chain amino acid transport system permease protein	100	95
BPSS0578	high-affinity branched-chain amino acid transport ATP-binding protein	100	97
BPSS0579	high-affinity branched-chain amino acid transport ATP-binding protein	100	97
BPSS0580	putative peptidase	100	93
BPSS0581	putative salicylate biosynthesis isochorismate synthase	100	87
BPSS0582	salicylate biosynthesis protein	100	92
BPSS0583	pyochelin biosynthetic protein	100	90
BPSS0584	salicyl-AMP ligase	100	92
BPSS0585	AraC family regulatory protein	100	95
BPSS0588	pyochelin biosynthetic protein	100	88
BPSS0589	putative ATP-binding component of ABC transporter	100	90
BPSS0590	probable ATP-binding component of ABC transporter	100	88
BPSS0591	Fe(III)-pyochelin receptor precursor	100	92
BPSS0592	putative membrane protein	99	86
BPSS0593	putative iron-regulated membrane protein	100	89
BPSS0595	2Fe-2S iron-sulfur	100	98
BPSS0596	sigma-54 activated regulatory protein	100	93
BPSS0597	putative membrane protein	100	92
BPSS0598	conserved hypothetical protein	100	98
BPSS0601	putative permease protein	100	97
BPSS0603	hypothetical protein	97	90
BPSS0604	hypothetical protein	100	87
BPSS0611	putative membrane protein	100	93
BPSS0612	putative oxidoreductase	100	96
BPSS0613	conserved hypothetical protein	100	94
BPSS0614	putative membrane protein	100	95
BPSS0615	putative membrane protein	100	89
BPSS0616	AraC family regulatory protein	100	94
BPSS0617	acyl-CoA dehydrogenase	100	98
BPSS0618	AMP-binding enzyme	100	94
BPSS0619	methylmalonate-semialdehyde dehydrogenase	100	96
BPSS0620	3-hydroxyisobutyrate dehydrogenase	100	97
BPSS0621	enoyl-CoA hydratase/isomerase family protein	100	95

BPSS0622	enoyl-CoA hydratase/isomerase family	99	93
BPSS0623	outer membrane efflux protein	100	90
BPSS0624	macrolide-specific ABC-type efflux carrier	100	95
BPSS0625	putative drug-efflux protein	100	92
BPSS0626	luciferase-like monooxygenase	100	96
BPSS0627	putative ribonuclease	100	95
BPSS0646	putative membrane protein	100	90
BPSS0678	response regulator protein (partial)	100	86
BPSS0698	2,4-dihydroxyhept-2-ene-1,7-dioic acid aldolase	100	95
BPSS0703	putative transmembrane ABC transporter protein	100	95
BPSS0710	enoyl-CoA hydratase/isomerase family protein	98	94
BPSS0711	alanine racemase, catabolic	100	91
BPSS0713	conserved hypothetical protein	100	93
BPSS0714	conserved hypothetical protein	100	93
BPSS0733	putative methyl-accepting chemotaxis protein	98	94
BPSS0852	putative inosine-uridine preferring nucleoside hydrolase	100	93
BPSS0853	putative ribokinase	100	92
BPSS0855	conserved hypothetical protein	100	91
BPSS0856	conserved hypothetical protein	100	94
BPSS0919	conserved hypothetical protein	100	87
BPSS0923	putative hydrolase	100	92
BPSS0924	putative membrane protein	100	96
BPSS0925	short chain dehydrogenase	100	94
BPSS0926	flavin-binding monooxygenase-like protein	100	96
BPSS1034	putative membrane protein	100	89
BPSS1036	putative Acyl-CoA dehydrogenase	100	90
BPSS1047	hypothetical bacteriophage protein	100	100
BPSS1048	hypothetical bacteriophage protein	100	100
BPSS1051	hypothetical bacteriophage protein	100	98
BPSS1052	hypothetical bacteriophage replication protein	100	99
BPSS1053	hypothetical bacteriophage-acquired protein	100	97
BPSS1054	hypothetical bacteriophage-acquired protein	100	98
BPSS1055	putative partition protein	100	99
BPSS1056	copG family protein	98	100
BPSS1057	putative bacteriophage gp29 protein	100	99
BPSS1058	putative bacteriophage gp30 protein	100	98
BPSS1059	putative bacteriophage gp31 protein	100	98
BPSS1064	putative bacteriophage protein	100	98
BPSS1065	putative major capsid protein precursor	100	99
BPSS1066	putative bacteriophage terminase, endonuclease subunit	100	99
BPSS1067	putative bacteriophage head completion/stabilization protein	100	92
BPSS1068	hypothetical bacteriophage protein	100	98
BPSS1069	putative bacteriophage tail protein X	100	100
BPSS1070	putative bacteriophage membrane protein	100	100
BPSS1071	putative bacteriophage membrane protein	100	98
BPSS1072	putative bacteriophage-acquired protein	100	98
BPSS1073	putative bacteriophage protein	100	91
BPSS1074	putative bacteriophage tail completion protein R	100	100
BPSS1075	putative bacteriophage tail completion protein S	100	100

BPSS1076	hypothetical bacteriophage protein	100	100
BPSS1077	putative site-specific DNA methyltransferase	100	98
BPSS1078	putative bacteriophage baseplate assembly protein V	100	96
BPSS1079	putative bacteriophage baseplate assembly protein W	100	100
BPSS1080	putative bacteriophage baseplate assembly protein J	100	95
BPSS1081	putative bacteriophage tail protein I	100	99
BPSS1082	putative bacteriophage protein gp17	100	98
BPSS1083	putative bacteriophage-acquired protein	100	99
BPSS1084	putative bacteriophage major tail sheath protein	100	99
BPSS1085	putative bacteriophage major tail tube protein	100	94
BPSS1086	putative bacteriophage protein	100	100
BPSS1087	putative bacteriophage membrane protein	100	99
BPSS1088	putative bacteriophage tail-related protein	100	98
BPSS1089	putative bacteriophage late control gene D protein	100	98
BPSS1090	putative alanine dehydrogenase	100	96
BPSS1091	conserved hypothetical protein	100	97
BPSS1094	conserved hypothetical protein	100	92
BPSS1095	putative heat-shock chaperone protein	100	94
BPSS1096	putative chaperone heat-shock protein	100	91
BPSS1099	putative membrane protein	99	89
BPSS1100	putative cation transport ATPase protein	100	91
BPSS1102	conserved hypothetical protein	99	90
BPSS1103	putative periplasmic thiamine binding protein	100	92
BPSS1105	putative methyltransferase protein	100	89
BPSS1106	putative thymidylate synthase protein	100	91
BPSS1107	hypothetical protein	100	93
BPSS1115	hypothetical protein	100	88
BPSS1123	conserved hypothetical protein	100	97
BPSS1124	conserved hypothetical protein	100	98
BPSS1125	putative riboflavin biosynthesis protein	100	88
BPSS1126	putative O-methyltransferase	100	97
BPSS1127	putative glyoxylase/bleomycin resistance protein/dioxygenase superfamily protein	100	93
BPSS1128	major facilitator superfamily transporter homolog	100	97
BPSS1129	putative membrane protein	100	95
BPSS1130	hypothetical protein	100	93
BPSS1131	putative LuxR family transcriptional regulator	100	97
BPSS1132	putative membrane protein	100	96
BPSS1133	putative 2,4-dienoyl-CoA reductase	100	93
BPSS1134	PadR-like family regulatory protein	100	95
BPSS1138	conserved hypothetical protein	97	92
BPSS1139	conserved hypothetical protein	100	97
BPSS1140	putative universal stress family protein	100	89
BPSS1142	putative hydrogenase/oxidoreductase subunit	98	95
BPSS1144	putative hydrogenase/NADPH dehydrogenase subunit	100	96
BPSS1145	putative membrane protein	100	98
BPSS1146	formate hydrogenlyase subunit 4	100	97
BPSS1147	putative hydrogenase subunit	100	95
BPSS1148	putative membrane protein	100	95
BPSS1149	hypothetical protein	100	92

BPSS1150	putative phosphocarrier protein	100	93
BPSS1151	putative spermidine synthase protein	99	91
BPSS1152	putative DNA-binding protein	100	98
BPSS1154	putative nitrate/nitrite transporter	100	93
BPSS1155	putative rotamase/peptidyl-prolyl cis-trans isomerase family protein	100	87
BPSS1156	putative respiratory nitrate reductase subunit	100	91
BPSS1157	putative respiratory nitrate reductase subunit	100	90
BPSS1158	putative respiratory nitrate reductase subunit	100	96
BPSS1159	putative respiratory nitrate reductase subunit	100	94
BPSS1160	putative two component sensor regulator	100	90
BPSS1161	putative LuxR family transcriptional regulator	100	96
BPSS1162	putative response regulator receiver domain protein	100	92
BPSS1163	putative fumarate and nitrate reduction family regulatory protein	100	93
BPSS1164	conserved hypothetical protein	100	94
BPSS1165	putative alpha-ketoglutarate-dependent taurine dioxygenase	100	94
BPSS1167	putative thioesterase	100	96
BPSS1168	putative acetyltransferase protein	100	90
BPSS1169	conserved hypothetical protein	100	94
BPSS1171	putative non-ribosomal peptide synthase/polyketide synthase	100	87
BPSS1172	putative non-ribosomal peptide synthase/polyketide synthase	100	91
BPSS1173	putative non-ribosomal peptide/polyketide synthase protein	100	86
BPSS1175	putative oligopeptidase A	99	86
BPSS1176	putative autoinducer-binding transcriptional activator protein	100	95
BPSS1178	putative transmembrane antiporter Na+ or K+/H+ exchanger	100	92
BPSS1180	putative N-acyl homoserine lactone synthase	100	91
BPSS1181	putative surfactin/non-ribosomally encoded peptide/polyketide synthase	100	90
BPSS1182	putative acyl carrier protein	100	91
BPSS1183	putative non-ribosomally encoded peptide/polyketide synthase	100	97
BPSS1184	hypothetical protein	100	87
BPSS1190	conserved hypothetical protein	100	92
BPSS1191	putative 1-aminocyclopropane-1-carboxylate deaminase	100	89
BPSS1192	hypothetical protein	100	87
BPSS1193	putative non-ribosomal peptide/polyketide synthase	100	88
BPSS1196	SyrP-like regulatory protein	100	92
BPSS1198	putative exported protein	100	95
BPSS1199	hypothetical protein	100	96
BPSS1200	putative GntR family transcriptional regulatory protein	99	88
BPSS1201	conserved hypothetical protein	97	95
BPSS1203	aspartate carbomyltransferase	100	97
BPSS1204	putative iron transport receptor protein	100	95
BPSS1205	conserved hypothetical protein	99	94
BPSS1206	putative iron-regulated protein	100	96
BPSS1207	transposase (partial)	100	97
BPSS1210	conserved hypothetical protein	99	95
BPSS1211	putative membrane protein	100	93
BPSS1212	conserved hypothetical protein	100	92
BPSS1213	conserved hypothetical protein	100	93
BPSS1217	putative exported protein	100	93
BPSS1218	putative methyl-accepting chemotaxis protein	100	95

BPSS1247	putative nitrate transporter component	100	89
BPSS1253	putative LysR family transcriptional regulator	100	98
BPSS1279	putative threonine dehydratase	100	91
BPSS1280	hypothetical protein	100	89
BPSS1281	conserved hypothetical protein	100	96
BPSS1282	putative lipoprotein	100	86
BPSS1363	putative exported protein	100	86
BPSS1366	putative sarcosine oxidase gamma subunit	100	92
BPSS1367	putative sarcosine oxidase alpha subunit	100	97
BPSS1368	putative sarcosine oxidase delta subunit	100	92
BPSS1369	putative sarcosine oxidase beta subunit	100	99
BPSS1370	putative L-serine dehydratase	100	97
BPSS1373	conserved hypothetical protein	99	92
BPSS1437	putative lipoprotein	99	88
BPSS1438	hypothetical protein	100	90
BPSS1467	putative aminotransferase protein	99	95
BPSS1472	putative transposase	100	93
BPSS1574	periplasmic component of taurine ABC transporter	100	92
BPSS1575	alpha-ketoglutarate-dependent taurine dioxygenase	100	90
BPSS1614	putative type III secretion protein	99	91
BPSS1619	putative type III secretion protein	100	89
BPSS1624	putative type III secretion protein	100	87
BPSS1683	putative lipopolysaccharide biosynthesis-related membrane protein	99	90
BPSS1686	putative exported protein	100	94
BPSS1689	putative UDP-glucose 4-epimerase	100	91
BPSS1713	conserved hypothetical protein	99	92
BPSS1731	putative pigment biosynthesis-related tyrosinase protein	100	93
BPSS1733	putative membrane protein	100	93
BPSS1734	putative peptide hydrolase	100	94
BPSS1735	putative ABC transport system, membrane protein	100	97
BPSS1741	Lipase precursor	100	93
BPSS1754	putative membrane protein	100	88
BPSS1798	conserved hypothetical protein	100	88
BPSS1804	hypothetical protein	100	88
BPSS1805	putative membrane protein	100	97
BPSS1806	conserved hypothetical protein	100	88
BPSS1807	4-hydroxy-2-oxovalerate aldolase	100	96
BPSS1808	acetaldehyde dehydrogenase	100	93
BPSS1809	putative thioesterase	100	93
BPSS1810	branched-chain amino acid aminotransferase	100	97
BPSS1811	putative transferase	100	96
BPSS1812	putative non-ribosomal peptide synthesis thioesterase	100	95
BPSS1813	putative non-ribosomal peptide synthase related protein	100	98
BPSS1814	putative phosphopantetheine attachment protein	100	97
BPSS1815	putative non-ribosomal peptide synthase	100	94
BPSS1816	putative regulatory protein	100	89
BPSS1817	hypothetical protein	100	87
BPSS1818	putative membrane protein	100	97
BPSS1819	putative serine/threonine protein phosphatase	100	94

BPSS1820	conserved hypothetical protein	99	96
BPSS1821	conserved hypothetical protein	100	96
BPSS1822	putative membrane protein	100	95
BPSS1823	peptidyl-prolyl cis-trans isomerase	100	98
BPSS1825	putative glycosyltransferase	100	96
BPSS1826	putative glycosyltransferase	100	92
BPSS1827	putative membrane protein	100	96
BPSS1828	putative glycosyltransferase group 1 protein	100	92
BPSS1829	putative glycosyltransferase	100	93
BPSS1830	putative exopolysaccharide biosynthesis related tyrosine-protein kinase	100	96
BPSS1831	putative exopolysaccharide (EPS) biosynthesis related polysaccharide lipoprotein	100	97
BPSS1832	putative exopolysaccharide (EPS) biosynthesis related low molecular weight protein-tyrosine-phos	100	93
BPSS1833	UDP-glucose 6-dehydrogenase 2	100	94
BPSS1834	putative lipopolysaccharide biosynthesis related protein	100	95
BPSS1881	putative membrane protein	100	95
BPSS1937	putative ABC transport system, exported protein	100	96
BPSS1974	putative lipoprotein	100	91
BPSS1979	hypothetical protein	100	88
BPSS1981	conserved hypothetical protein	100	92
BPSS1982	putative cation efflux related membrane protein	100	89
BPSS1983	putative deoxyribonuclease	100	90
BPSS1984	hypothetical protein	100	91
BPSS1985	hypothetical protein	100	89
BPSS1986	hypothetical protein	100	92
BPSS1987	putative phosphesterase	100	90
BPSS1988	hypothetical protein	99	89
BPSS1989	conserved hypothetical protein	100	91
BPSS1990	putative aldolase	100	93
BPSS1991	conserved hypothetical protein	100	88
BPSS1992	putative exported peptidase	100	95
BPSS1993	serine metalloprotease precursor	100	89
BPSS1994	metal-related two-component system, response regulator	100	97
BPSS1995	metal-related two-component system, histidine kinase	100	94
BPSS1996	putative exported protein	100	92
BPSS1997	beta-lactamase precursor	100	92
BPSS2003	putative conserved periplasmic protein	100	91
BPSS2009	glucosamine--fructose-6-phosphate aminotransferase [isomerizing]	100	95
BPSS2010	hypothetical protein	98	90
BPSS2011	putative outer membrane protein	100	86
BPSS2012	putative membrane protein	100	88
BPSS2014	hypothetical protein	100	91
BPSS2015	putative inner membrane glycosyltransferase	100	91
BPSS2016	UDP-N-acetylglucosamine 2-epimerase	100	93
BPSS2017	putative LysR-family transcriptional regulator	99	97
BPSS2018	putative membrane protein	100	93
BPSS2021	putative decarboxylase	98	89
BPSS2022	putative outer membrane protein	100	89
BPSS2026	putative carbohydrate kinase	99	90
BPSS2028	putative TetR-family transcriptional regulator	100	91

BPSS2029	putative acyl-CoA dehydrogenase	100	93
BPSS2030	putative short-chain dehydrogenase	98	93
BPSS2031	acetyl-CoA carboxylase carboxyltransferase	100	95
BPSS2032	putative acyl-CoA dehydrogenase	100	97
BPSS2033	putative enoyl-CoA hydratase/isomerase	99	86
BPSS2034	putative acetyl-/propionyl-coenzyme A carboxylase alpha chain protein	100	89
BPSS2035	conserved hypothetical protein	100	95
BPSS2036	putative AMP-binding acetyl-CoA synthetase	100	91
BPSS2037	putative inner membrane fatty acid desaturase	100	96
BPSS2038	putative polyketide synthase subunit	100	90
BPSS2039	putative cyclopropane-fatty-acyl-phospholipid synthase	100	97
BPSS2040	putative inner membrane fatty acid desaturase	100	97
BPSS2041	conserved hypothetical protein	100	97
BPSS2042	putative inner membrane fatty-acid--CoA ligase	100	93
BPSS2043	conserved hypothetical protein	100	98
BPSS2044	conserved hypothetical protein	99	95
BPSS2045	conserved hypothetical protein	100	99
BPSS2078	conserved hypothetical protein	100	95
BPSS2116	putative D-serine dehydratase	99	88
BPSS2149	putative MmgE/Prp family protein	100	94
BPSS2150	putative citrate lyase	100	95
BPSS2151	putative Acyl-CoA transferase/carnitine dehydratase protein	100	93
BPSS2152	putative zinc-binding dehydrogenase	100	97
BPSS2153	putative isochorismatase family protein	100	96
BPSS2154	putative MFS family transporter	100	91
BPSS2155	putative MFS family transporter	100	91
BPSS2156	conserved hypothetical protein	100	93
BPSS2157	putative Acyl-CoA transferase/carnitine dehydratase protein	100	98
BPSS2172	hypothetical protein	100	94
BPSS2173	carbon starvation protein A	100	98
BPSS2174	putative cytochrome P460	100	87
BPSS2175	putative hydrolase	100	91
BPSS2176	conserved hypothetical protein	100	92
BPSS2177	putative LysR-family transcriptional regulator	100	91
BPSS2180	putative membrane protein	100	92
BPSS2181	conserved hypothetical protein	100	95
BPSS2182	putative glycosyl transferase	100	96
BPSS2183	putative membrane protein	100	87
BPSS2184	putative membrane protein	100	91
BPSS2185	putative pilus subunit protein	100	96
BPSS2186	putative pilus subunit protein	100	87
BPSS2187	putative pilus assembly-related outer membrane protein	100	91
BPSS2188	conserved hypothetical protein	100	93
BPSS2189	putative pilus assembly-related, exported protein	100	94
BPSS2190	putative outer membrane protein	100	86
BPSS2191	putative lipoprotein	100	94
BPSS2192	putative membrane protein	100	93
BPSS2193	putative membrane protein	100	92
BPSS2194	putative membrane protein	100	92

BPSS2195	putative pilus assembly-related protein	100	93
BPSS2196	putative type II/IV secretion system-related protein	100	96
BPSS2197	putative membrane protein	100	93
BPSS2198	putative membrane protein	100	96
BPSS2199	putative AsnC-family regulatory protein	100	96
BPSS2200	aromatic-amino-acid aminotransferase	100	92
BPSS2201	conserved hypothetical protein	98	94
BPSS2202	putative porin related, membrane protein	100	90
BPSS2203	putative amino acid transport, membrane protein	100	95
BPSS2204	putative GntR-family regulatory protein	100	98
BPSS2205	conserved hypothetical protein	100	95
BPSS2206	putative transport related, membrane protein	100	94
BPSS2207	putative LysR-family transcriptional regulator	100	97
BPSS2320	4'-phosphopantetheinyl transferase superfamily protein	100	87

Segment 7- Bt unique

Gene	Description
BTH_I0005	cobalamin synthesis protein/P47K family protein
BTH_I0029	flagellar biosynthesis protein
BTH_I0034	methyltransferase
BTH_I0063	hypothetical protein
BTH_I0065	hypothetical protein
BTH_I0067	hypothetical protein
BTH_I0069	lipoprotein, putative
BTH_I0074	transposase, putative
BTH_I0075	recombinase
BTH_I0076	stage 0 sporulation protein J, putative
BTH_I0077	L0013 protein
BTH_I0089	conserved hypothetical protein
BTH_I0091	phage portal protein, PBSX family
BTH_I0092	Fels-2 prophage protein
BTH_I0093	conserved hypothetical protein
BTH_I0094	Phage integrase
BTH_I0095	hypothetical protein
BTH_I0096	hypothetical protein
BTH_I0097	conserved hypothetical protein
BTH_I0098	conserved hypothetical protein
BTH_I0099	conserved hypothetical protein
BTH_I0100	conserved hypothetical protein
BTH_I0101	conserved hypothetical protein
BTH_I0102	DEAD/DEAH box helicase:Helicase, C-terminal
BTH_I0103	conserved hypothetical protein
BTH_I0104	conserved hypothetical protein
BTH_I0105	hypothetical protein
BTH_I0106	PUTATIVE HEMAGGLUTININ/HEMOLYSIN-RELATED PROTEIN, putative
BTH_I0107	possible transcriptional regulator, XRE family
BTH_I0108	hypothetical protein
BTH_I0109	conserved hypothetical protein
BTH_I0110	gp47 (Bacteriophage A118) homolog lin0084

BTH_I0111	conserved hypothetical protein
BTH_I0112	conserved hypothetical protein
BTH_I0113	Protein kinase domain protein
BTH_I0114	conserved hypothetical protein
BTH_I0115	bacteriophage phiC31 resistance protein pglY
BTH_I0116	bacteriophage phiC31 resistance protein pglZ, putative
BTH_I0117	gp31
BTH_I0118	gp30
BTH_I0119	gp29
BTH_I0132	sun protein
BTH_I0167	hypothetical protein
BTH_I0175	hypothetical protein
BTH_I0177	conserved hypothetical protein
BTH_I0184	transcriptional regulator, LysR family
BTH_I0185	dihydrodipicolinate synthetase family protein
BTH_I0186	aldehyde dehydrogenase family protein
BTH_I0187	MFS transporter, phthalate permease family
BTH_I0188	conserved domain protein
BTH_I0189	glucarate dehydratase
BTH_I0190	D-galactarate dehydratase
BTH_I0191	conserved hypothetical protein
BTH_I0195	Flagellar hook-length control protein, putative
BTH_I0204	hypothetical protein
BTH_I0207	lipoprotein, putative
BTH_I0214	conserved hypothetical protein
BTH_I0215	hypothetical protein
BTH_I0239	FlgA family family
BTH_I0256	conserved hypothetical protein
BTH_I0259	glyoxalase family protein
BTH_I0265	transposase, putative
BTH_I0266	Transposase (IS4 family)
BTH_I0268	hypothetical protein
BTH_I0269	manganese/iron transporter, NRAMP family
BTH_I0270	lipoprotein, putative
BTH_I0273	conserved hypothetical protein
BTH_I0274	DNA-binding response regulator
BTH_I0275	sensor histidine kinase
BTH_I0276	transcriptional regulator, LysR family
BTH_I0277	conserved hypothetical protein
BTH_I0278	hypothetical protein
BTH_I0280	conserved hypothetical protein
BTH_I0282	heavy-metal-associated domain protein-related protein
BTH_I0287	streptavidin,putative
BTH_I0293	hypothetical protein
BTH_I0296	hypothetical protein
BTH_I0303	related to SH3-domain protein Cyk3
BTH_I0304	hypothetical protein
BTH_I0311	Protein of unknown function (DUF1289) family
BTH_I0318	hypothetical protein

BTH_I0322	lactonase
BTH_I0324	sensor histidine kinase
BTH_I0327	PUTATIVE ATP-SENSITIVE INWARD RECTIFIER POTASSIUM CHANNEL RELATED TRANSMEMBRANE PROTEIN
BTH_I0334	conserved hypothetical protein
BTH_I0336	cutC family protein
BTH_I0351	oxidoreductase, short-chain dehydrogenase/reductase family
BTH_I0360	hypothetical protein
BTH_I0361	transcriptional regulator, IclR family
BTH_I0368	hypothetical protein
BTH_I0371	conserved hypothetical protein
BTH_I0384	conserved hypothetical protein
BTH_I0387	quinone oxidoreductase
BTH_I0388	transcriptional regulator, LysR family
BTH_I0390	hypothetical protein
BTH_I0391	conserved hypothetical protein
BTH_I0403	conserved hypothetical protein
BTH_I0414	HesA/MoeB/ThiF family protein
BTH_I0455	conserved hypothetical protein
BTH_I0467	hypothetical protein
BTH_I0499	major facilitator family transporter
BTH_I0505	transposase
BTH_I0506	carboxymuconolactone decarboxylase family protein
BTH_I0515	conserved hypothetical protein
BTH_I0516	conserved hypothetical protein
BTH_I0525	PAP2 family protein
BTH_I0527	conserved hypothetical protein
BTH_I0534	conserved hypothetical protein
BTH_I0557	thiopurine S-methyltransferase family protein
BTH_I0568	hypothetical protein
BTH_I0571	thioesterase domain protein
BTH_I0579	integral membrane protein
BTH_I0589	conserved hypothetical protein TIGR00043, putative
BTH_I0597	hypothetical protein
BTH_I0611	DgoA protein
BTH_I0612	Protein of unknown function (DUF636) family
BTH_I0616	hypothetical protein
BTH_I0619	cholesterol oxidase
BTH_I0623	Erythromycin esterase family
BTH_I0628	Bacterial protein of unknown function (DUF879) superfamily
BTH_I0629	hypothetical protein
BTH_I0630	hypothetical protein
BTH_I0636	conserved hypothetical protein
BTH_I0637	conserved hypothetical protein
BTH_I0638	Phage integrase family domain protein
BTH_I0642	DNA-binding response regulator
BTH_I0644	regulatory protein RecX, putative
BTH_I0649	Pilin (bacterial filament) subfamily
BTH_I0689	cation ABC transporter, ATP-binding protein, putative
BTH_I0691	transcriptional regulator, putative

BTH_I0694	PUTATIVE TAGATOSE 6-PHOSPHATE KINASE PROTEIN
BTH_I0711	cyclase, putative
BTH_I0714	peptide methionine sulfoxide reductase
BTH_I0715	Protein of unknown function family
BTH_I0731	UDP-N-acetylenolpyruvoylglucosamine reductase
BTH_I0753	citrate synthase family protein
BTH_I0765	conserved hypothetical protein
BTH_I0784	FscRII, putative
BTH_I0797	hypothetical protein
BTH_I0798	hypothetical protein
BTH_I0805	DNA-binding protein
BTH_I0806	hypothetical protein
BTH_I0807	PROBABLE INTEGRASE PROTEIN
BTH_I0808	transcriptional regulator
BTH_I0809	conserved hypothetical protein
BTH_I0810	TrapT dctQ-M fusion permease, dicarboxylate transport
BTH_I0811	TrapT family, dctP subunit, C4-dicarboxylate periplasmic binding protein
BTH_I0812	ribose operon repressor, putative
BTH_I0825	conserved hypothetical protein
BTH_I0839	phosphoglycerate mutase family, putative
BTH_I0853	hypothetical protein
BTH_I0868	lipoprotein, putative
BTH_I0879	endo/excinuclease domain protein
BTH_I0880	drug resistance transporter, EmrB/QacA family
BTH_I0885	transcriptional regulator, LysR family, putative
BTH_I0886	acetyltransferase, GNAT family
BTH_I0890	amino acid ABC transporter, periplasmic amino acid-binding protein
BTH_I0902	conserved hypothetical protein
BTH_I0906	lipoprotein, putative
BTH_I0909	conserved hypothetical protein
BTH_I0910	hypothetical protein
BTH_I0911	hypothetical protein
BTH_I0912	probable transposase protein
BTH_I0913	phage related protein
BTH_I0914	hypothetical protein
BTH_I0915	hypothetical protein
BTH_I0917	gp11
BTH_I0918	gp12, putative
BTH_I0919	conserved hypothetical protein
BTH_I0920	hypothetical protein
BTH_I0921	Transposase (IS4 family)
BTH_I0922	transposase, putative
BTH_I0923	hypothetical protein
BTH_I0924	gp22
BTH_I0925	lysozyme, putative
BTH_I0926	transposase, Mutator family
BTH_I0927	gp23
BTH_I0928	site-specific recombinase, phage integrase family, truncation
BTH_I0930	hypothetical protein

BTH_I0931	conserved hypothetical protein
BTH_I0932	cell wall surface anchor family protein , putative
BTH_I0933	Peptidase C39 family family
BTH_I0934	hypothetical protein
BTH_I0935	conserved hypothetical protein
BTH_I0936	sigma-54 dependent DNA-binding transcriptional regulator
BTH_I0937	rubredoxin-related protein
BTH_I0945	N-carbamyl-L-amino acid amidohydrolase
BTH_I0953	conserved hypothetical protein
BTH_I0958	conserved hypothetical protein
BTH_I0969	membrane protein, putative
BTH_I0976	cell division protein FtsK
BTH_I0978	hypothetical protein
BTH_I0981	ATP-dependent RNA helicase RhlE
BTH_I0989	glycosyl transferase, group 1 family protein
BTH_I0990	lipopolysaccharide core biosynthesis heptosyltransferase
BTH_I0992	conserved hypothetical protein
BTH_I0996	carboxymuconolactone decarboxylase family protein
BTH_I0997	RNA polymerase sigma-70 factor, ECF family
BTH_I0998	transcriptional regulator, LysR family
BTH_I0999	transcriptional regulator, LysR family
BTH_I1000	MFS permease
BTH_I1001	hypothetical protein
BTH_I1002	conserved hypothetical protein
BTH_I1003	conserved hypothetical protein
BTH_I1004	conserved hypothetical protein
BTH_I1005	phospholipase C accessory protein, putative
BTH_I1017	oxidoreductase, short chain dehydrogenase/reductase family family
BTH_I1026	Domain of unknown function (DUF333) family
BTH_I1033	major facilitator family transporter
BTH_I1082	lipoprotein, putative
BTH_I1086	enoyl-CoA hydratase/isomerase family protein
BTH_I1087	glutathione S-transferase N-terminal domain protein
BTH_I1089	conserved hypothetical protein
BTH_I1101	L0013 protein
BTH_I1102	TnpB protein
BTH_I1103	transposase, Mutator family
BTH_I1104	TnpC protein
BTH_I1126	conserved hypothetical protein
BTH_I1156	hypothetical protein
BTH_I1160	MJ0042 family finger-like domain protein
BTH_I1167	hypothetical protein
BTH_I1185	hypothetical protein
BTH_I1199	carbonic anhydrases
BTH_I1200	transcriptional regulator, LysR family
BTH_I1201	hypothetical protein
BTH_I1231	conserved hypothetical protein
BTH_I1241	hypothetical protein
BTH_I1244	hypothetical protein

BTH_I1245	lipoprotein, putative
BTH_I1249	crossover junction endodeoxyribonuclease RuvC
BTH_I1254	Xaa-Pro aminopeptidase
BTH_I1258	NUDIX domain protein
BTH_I1264	hypothetical protein
BTH_I1267	Protein of unknown function (DUF330) family
BTH_I1274	major facilitator family transporter
BTH_I1283	hypothetical protein
BTH_I1285	transcriptional regulator, putative
BTH_I1307	conserved hypothetical protein
BTH_I1323	YafJ
BTH_I1324	mannose-1-phosphate guanylyltransferase/mannose-6-phosphate isomerase
BTH_I1325	WcbA
BTH_I1326	glycosyltransferase, putative
BTH_I1327	WcbC
BTH_I1328	methyltransferase, FkbM family domain protein
BTH_I1329	glycosyltransferase, putative
BTH_I1330	possible glycosyltransferase WbpX, putative
BTH_I1331	GDP-mannose 4,6-dehydratase
BTH_I1332	GDP-6-deoxy-D-lyxo-4-hexulose reductase, putative
BTH_I1333	glycosyl transferase, group 1 family protein, putative
BTH_I1334	glycosyltransferase, putative
BTH_I1335	BexA
BTH_I1336	ctrC protein
BTH_I1337	WcbD
BTH_I1338	WcbO
BTH_I1339	oxidoreductase, short-chain dehydrogenase/reductase family
BTH_I1340	putative capsular polysaccharide biosynthesis protein WcbQ
BTH_I1341	FATTY ACID SYNTHASE TRANSMEMBRANE PROTEIN
BTH_I1342	lpxc
BTH_I1345	gp48, putative
BTH_I1346	hypothetical protein
BTH_I1348	membrane protein, putative
BTH_I1364	flagellar transcriptional activator FlhD
BTH_I1365	fatty acid desaturase family protein, putative
BTH_I1366	Proline dehydrogenase superfamily, putative
BTH_I1367	aminotransferase, class V superfamily, putative
BTH_I1368	membrane protein, putative
BTH_I1369	hypothetical protein
BTH_I1381	conserved hypothetical protein
BTH_I1382	conserved hypothetical protein
BTH_I1383	hypothetical protein
BTH_I1384	hypothetical protein
BTH_I1389	conserved hypothetical protein
BTH_I1393	EAL domain protein
BTH_I1401	nitroreductase family protein
BTH_I1408	xanthine dehydrogenase, N-terminal subunit
BTH_I1418	conserved hypothetical protein
BTH_I1419	transport-associated domain protein

BTH_I1423	hypothetical protein
BTH_I1435	conserved hypothetical protein
BTH_I1438	hypothetical protein
BTH_I1439	TnpC protein
BTH_I1441	L0013 protein
BTH_I1442	conserved hypothetical protein
BTH_I1443	Superfamily I DNA and RNA helicases
BTH_I1444	hydrolase of HAD-superfamily
BTH_I1445	L0013 protein
BTH_I1447	TnpC protein
BTH_I1449	TnpC protein
BTH_I1450	TnpC protein
BTH_I1452	L0013 protein
BTH_I1453	transposase
BTH_I1454	transposase
BTH_I1455	hypothetical protein
BTH_I1456	hypothetical protein
BTH_I1480	glycosyl transferase, group 2 family protein, putative
BTH_I1481	epimerase/dehydratase
BTH_I1505	omega-amino acid--pyruvate aminotransferase
BTH_I1515	hypothetical protein
BTH_I1516	quinone oxidoreductase
BTH_I1517	transcriptional regulator, LysR family
BTH_I1523	conserved hypothetical protein
BTH_I1532	conserved hypothetical protein
BTH_I1533	probable helicase Z1568
BTH_I1534	conserved domain protein
BTH_I1536	transposase
BTH_I1537	transposase
BTH_I1539	helicase, putative
BTH_I1540	Doc protein
BTH_I1545	BKRF1 encodes EBNA-1 protein-like
BTH_I1557	hypothetical protein
BTH_I1575	conserved hypothetical protein
BTH_I1576	metabolite:proton symporter family protein
BTH_I1578	Protein of unknown function (DUF636) family
BTH_I1579	conserved hypothetical protein
BTH_I1580	conserved hypothetical protein
BTH_I1593	hypothetical protein
BTH_I1594	cold-shock domain family protein-related protein
BTH_I1600	conserved hypothetical protein
BTH_I1601	Transglycosylase associated protein family
BTH_I1603	hypothetical protein
BTH_I1607	hypothetical protein
BTH_I1616	hypothetical protein
BTH_I1640	conserved hypothetical protein
BTH_I1647	murein transglycosylase domain protein
BTH_I1659	membrane protein, putative
BTH_I1665	pyrophosphatase, MutT/nudix family

BTH_I1674	dihydrofolate reductase
BTH_I1675	sigma-54 dependent DNA-binding transcriptional regulator
BTH_I1676	hypothetical protein
BTH_I1677	hypothetical protein
BTH_I1678	conserved hypothetical protein
BTH_I1682	transcriptional regulator, ArsR family
BTH_I1683	conserved hypothetical protein
BTH_I1690	membrane protein, putative
BTH_I1691	AzlC family protein
BTH_I1692	transcriptional regulator, AraC family, putative
BTH_I1713	Maf-like protein
BTH_I1730	ribonuclease III, putative
BTH_I1744	transposase, Mutator family
BTH_I1762	phospholipase C
BTH_I1764	hydrolase, alpha/beta fold family
BTH_I1765	luciferase-like monooxygenase
BTH_I1766	syl transferase, group 2 family protein
BTH_I1781	glutathione S-transferase, putative
BTH_I1782	transcriptional regulator, LysR family, putative
BTH_I1787	ubiquinol oxidase, subunit I
BTH_I1795	sufD domain protein
BTH_I1797	ABC transporter
BTH_I1802	membrane protein, putative
BTH_I1818	conserved hypothetical protein
BTH_I1819	phosphopantetheinyltransferase family protein
BTH_I1823	Bacterial protein of unknown function (DUF886) superfamily
BTH_I1830	conserved hypothetical protein
BTH_I1834	CoA transferase, CAIB/BAIF family
BTH_I1848	methyltransferase, FkbM family domain protein
BTH_I1858	acyltransferase family protein
BTH_I1859	DNA polymerase IV
BTH_I1871	iron-sulfur cluster binding protein
BTH_I1884	peptide chain release factor 2, programmed
BTH_I1914	integrative genetic element Gsu32, integrase, putative
BTH_I1915	Prophage CP4-57 regulatory protein (AlpA) family
BTH_I1916	hypothetical protein
BTH_I1917	hypothetical protein
BTH_I1918	pyocin R2_PP, tail formation
BTH_I1919	hypothetical protein
BTH_I1920	gp24
BTH_I1921	gp26
BTH_I1922	gp25a
BTH_I1923	gp26
BTH_I1924	conserved hypothetical protein
BTH_I1925	hypothetical protein
BTH_I1929	gp33
BTH_I1930	transposase
BTH_I1931	transposase
BTH_I1932	hypothetical protein

BTH_I1933	conserved hypothetical protein
BTH_I1952	adenylylsulfate kinase
BTH_I1969	kinase, putative
BTH_I2003	phage SPO1 DNA polymerase domain protein
BTH_I2009	conserved hypothetical protein
BTH_I2021	hypothetical protein
BTH_I2024	pseudouridine synthase family protein
BTH_I2054	conserved hypothetical protein
BTH_I2057	conserved hypothetical protein
BTH_I2059	transporter, putative
BTH_I2060	conserved hypothetical protein
BTH_I2068	conserved hypothetical protein
BTH_I2074	transcriptional regulator, LysR family
BTH_I2075	Protein of unknown function (DUF1458) family
BTH_I2083	hypothetical protein
BTH_I2084	conserved hypothetical protein
BTH_I2087	DpgD protein
BTH_I2100	unnamed protein product; Highly similar to unknown protein of Photorhabdus and some similarities with unknown protein, putative
BTH_I2101	hypothetical protein
BTH_I2105	hypothetical protein
BTH_I2106	hypothetical protein
BTH_I2107	conserved hypothetical protein
BTH_I2108	hypothetical protein
BTH_I2110	hypothetical protein
BTH_I2113	porin, interruption-N
BTH_I2117	glycerate kinase 1
BTH_I2119	hypothetical protein
BTH_I2124	sigma 54 modulation protein, putative
BTH_I2126	hypothetical protein
BTH_I2127	4-carboxymuconolactone decarboxylase domain protein
BTH_I2146	conserved hypothetical protein
BTH_I2150	acetyltransferase, GNAT family
BTH_I2169	hypothetical protein
BTH_I2170	hypothetical protein
BTH_I2177	RNA polymerase sigma-70 factor, ECF subfamily
BTH_I2178	conserved hypothetical protein
BTH_I2186	lipoprotein, NLP/P60 family
BTH_I2200	molybdopterin biosynthesis moeA protein
BTH_I2204	Rrf2 family protein
BTH_I2210	lipoic acid synthetase
BTH_I2213	ribosomal protein L31
BTH_I2219	DNA polymerase III, subunits gamma and tau, programmed
BTH_I2258	polyhydroxyalkanoate synthesis repressor PhaR
BTH_I2262	hypothetical protein
BTH_I2270	outer membrane porin OpcP
BTH_I2286	conserved hypothetical protein
BTH_I2292	membrane protein, putative
BTH_I2294	conserved hypothetical protein
BTH_I2295	conserved hypothetical protein

BTH_I2305	transcriptional regulator, AsnC family
BTH_I2313	lipoprotein, putative
BTH_I2314	conserved hypothetical protein
BTH_I2315	lipoprotein, putative
BTH_I2330	conserved hypothetical protein
BTH_I2335	alcohol dehydrogenase, iron-containing
BTH_I2336	aldose 1-epimerase
BTH_I2337	xylose operon regluatory protein
BTH_I2338	xylose isomerase
BTH_I2339	D-xylose ABC transporter, periplasmic-D xylose binding protein
BTH_I2340	sugar ABC transporter, ATP-binding protein
BTH_I2341	sugar ABC transporter, permease protein
BTH_I2342	periplasmic ribose-binding protein
BTH_I2343	ATP binding protein of ABC transporter
BTH_I2344	ribose ABC transporter, permease protein VCA0129
BTH_I2345	ribose ABC transporter, permease protein
BTH_I2346	probable transposase protein
BTH_I2347	transposase, Mutator family
BTH_I2348	transposase, Mutator family
BTH_I2349	transposase, Mutator family
BTH_I2350	ISPsy10, transposase, truncation
BTH_I2351	conserved hypothetical protein
BTH_I2352	Sea27
BTH_I2353	Arachidonate 15-lipoxygenase precursor (15-LOX)
BTH_I2354	hypothetical protein
BTH_I2355	hydrolase, alpha/beta fold family, putative
BTH_I2356	serine protease, subtilase family
BTH_I2357	thioesterase type II
BTH_I2358	lipase/esterase
BTH_I2359	pyridine nucleotide-disulphide oxidoreductase, class II, truncation
BTH_I2360	nonribosomal peptide synthetase, putative
BTH_I2361	phosphotransferase enzyme family protein, putative
BTH_I2362	acyl-CoA dehydrogenase domain protein
BTH_I2363	polyketide synthase
BTH_I2364	peptide synthetase, putative
BTH_I2365	polyketide synthase
BTH_I2366	polyketide synthase
BTH_I2367	dihydroaeruginoic acid synthetase
BTH_I2368	hypothetical protein
BTH_I2369	transcriptional regulator, AraC family domain protein
BTH_I2371	transcriptional regulator, AraC family
BTH_I2376	long-chain-fatty-acid--CoA ligase, putative
BTH_I2378	ABC transporter, periplasmic glycine/betaine-binding protein, putative
BTH_I2382	hypothetical protein
BTH_I2390	glutathione S-transferase , putative
BTH_I2397	precorrin-6Y C5,15-methyltransferase (decarboxylating)
BTH_I2402	glycosyl hydrolase, family 18
BTH_I2403	hypothetical protein
BTH_I2404	carboxylesterase, putative

BTH_I2410	CbiG
BTH_I2412	cobyrinic acid a,c-diamide synthase
BTH_I2413	conserved hypothetical protein
BTH_I2417	non-ribosomal peptide synthetase, putative
BTH_I2418	peptide synthetase homolog
BTH_I2428	conserved hypothetical protein
BTH_I2429	hypothetical protein
BTH_I2437	hypothetical protein
BTH_I2438	conserved hypothetical protein
BTH_I2447	Peptidase family M23/M37
BTH_I2463	ABC transporter permease protein
BTH_I2464	permease
BTH_I2465	taurine ABC transporter, ATP-binding protein
BTH_I2483	conserved hypothetical protein
BTH_I2488	hypothetical protein
BTH_I2492	Transcriptional regulator family
BTH_I2503	hypothetical protein
BTH_I2513	amino acid ABC transporter, permease protein
BTH_I2523	conserved hypothetical protein
BTH_I2531	conserved hypothetical protein
BTH_I2536	conserved hypothetical protein
BTH_I2568	segregation and condensation protein B
BTH_I2571	hypothetical protein
BTH_I2573	Uncharacterized ACR, COG1434 family
BTH_I2574	hypothetical protein
BTH_I2579	conserved hypothetical protein
BTH_I2582	Ata11 protein
BTH_I2584	transposase
BTH_I2585	transposase
BTH_I2586	ISBma1, transposase
BTH_I2600	hypothetical protein
BTH_I2601	putative flavoprotein reductase
BTH_I2602	conserved hypothetical protein
BTH_I2603	transcriptional regulator, LysR family
BTH_I2608	3-oxoadipate CoA-succinyl transferase alpha subunit
BTH_I2622	Ser/Thr protein phosphatase family protein
BTH_I2623	hypothetical protein
BTH_I2652	branched-chain amino acid ABC transporter, permease/ATP binding protein, putative
BTH_I2654	thioesterase family protein
BTH_I2658	PROBABLE PROTEASE SIGNAL PEPTIDE PROTEIN
BTH_I2659	Major Facilitator Superfamily subfamily, putative
BTH_I2663	lipoprotein, putative
BTH_I2673	trans-aconitate methyltransferase
BTH_I2682	hypothetical protein
BTH_I2688	TnpC protein
BTH_I2690	L0013 protein
BTH_I2691	unnamed protein product; Highly similar to unknown protein of Photorhabdus luminescens, putative
BTH_I2692	unnamed protein product; Unknown protein, putative
BTH_I2694	hypothetical protein

BTH_I2695	hypothetical protein
BTH_I2698	conserved hypothetical protein
BTH_I2699	conserved hypothetical protein
BTH_I2700	lipoprotein, putative
BTH_I2702	lipoprotein, putative
BTH_I2707	conserved hypothetical protein
BTH_I2709	transposase, IS110 family, interruption
BTH_I2710	hypothetical protein
BTH_I2712	hypothetical protein
BTH_I2714	DNA-binding protein BprA
BTH_I2715	major facilitator family transporter
BTH_I2716	transcriptional regulator, LysR family, putative
BTH_I2717	Phage integrase
BTH_I2718	probable acetyltransferase STY4148
BTH_I2719	pathogenesis-related protein
BTH_I2720	conserved hypothetical protein
BTH_I2721	outer membrane hemolysin activator protein
BTH_I2722	conserved hypothetical protein
BTH_I2723	PROBABLE HEMAGGLUTININ-RELATED PROTEIN
BTH_I2724	conserved hypothetical protein
BTH_I2725	conserved hypothetical protein
BTH_I2726	gp47 (Bacteriophage A118) homolog lin0084
BTH_I2727	hypothetical protein
BTH_I2728	conserved hypothetical protein
BTH_I2729	conserved hypothetical protein
BTH_I2730	plasmid related protein
BTH_I2731	DNA-binding protein
BTH_I2732	conserved hypothetical protein
BTH_I2733	Helix-turn-helix domain protein, putative
BTH_I2734	lipoprotein, putative
BTH_I2735	TnpC protein
BTH_I2737	L0013 protein
BTH_I2738	conserved hypothetical protein
BTH_I2739	putative DNA mismatch repair protein
BTH_I2740	type I restriction-modification system endonuclease XF2739
BTH_I2741	conserved hypothetical protein
BTH_I2742	type I restriction-modification system specificitydeterminant XF2741
BTH_I2743	type I restriction system adenine methylase
BTH_I2744	transposase
BTH_I2745	transposase
BTH_I2746	recombinase
BTH_I2747	stage 0 sporulation protein J, putative
BTH_I2748	transposase, Mutator family
BTH_I2751	Aldose 1-epimerase family
BTH_I2752	lipoprotein NlpD, putative
BTH_I2761	PAP2 family protein
BTH_I2762	GH09231p
BTH_I2764	hypothetical protein
BTH_I2793	proline/betaine transporter

BTH_I2796	conserved hypothetical protein
BTH_I2798	ATP:dephospho-CoA triphosphoribosyl transferase
BTH_I2799	hypothetical protein
BTH_I2805	malonate transporter, L subunit
BTH_I2811	hypothetical protein
BTH_I2812	hypothetical protein
BTH_I2813	conserved hypothetical protein
BTH_I2814	transcriptional regulator, GntR family
BTH_I2815	putative membrane protein
BTH_I2816	conserved hypothetical protein
BTH_I2819	conserved hypothetical protein
BTH_I2820	carotenoid 9,10-9,10 cleavage dioxygenase, putative
BTH_I2824	GGDEF domain protein
BTH_I2825	conserved hypothetical protein
BTH_I2826	conserved hypothetical protein
BTH_I2827	outer membrane porin OpcP
BTH_I2828	transcriptional regulator, LacI family
BTH_I2832	hypothetical protein
BTH_I2833	ABC transporter, permease protein
BTH_I2839	hypothetical protein
BTH_I2841	conserved hypothetical protein
BTH_I2842	conserved hypothetical protein
BTH_I2843	conserved hypothetical protein
BTH_I2850	exodeoxyribonuclease V, alpha subunit
BTH_I2860	Cysteine dioxygenase type I family
BTH_I2879	hypothetical protein
BTH_I2881	hypothetical protein
BTH_I2882	two-component hybrid sensor and regulator
BTH_I2883	aliphatic compound ABC transporter, periplasmic substrate-binding protein
BTH_I2884	conserved hypothetical protein
BTH_I2889	L0013 protein
BTH_I2890	TnpB protein
BTH_I2891	hypothetical protein
BTH_I2892	transposase, Mutator family
BTH_I2893	TnpC protein
BTH_I2918	monooxygenase family protein
BTH_I2922	Uncharacterised BCR, putative
BTH_I2928	conserved hypothetical protein
BTH_I2941	conserved hypothetical protein
BTH_I2944	conserved hypothetical protein
BTH_I2946	hypothetical protein
BTH_I2947	fructose-specific IIABC component
BTH_I2948	secretion protein, putative
BTH_I2951	hypothetical protein
BTH_I2953	hypothetical protein
BTH_I2966	PUTATIVE TRANSMEMBRANE PROTEIN
BTH_I2969	transposase
BTH_I2970	ISSo2, transposase OrfB, truncation
BTH_I2971	hypothetical protein

BTH_I2973	stringent starvation protein B
BTH_I3010	hypothetical protein
BTH_I3025	type II/III secretion system protein
BTH_I3026	conserved hypothetical protein
BTH_I3088	transposase fragment
BTH_I3089	L0013 protein
BTH_I3090	TnpB protein
BTH_I3100	phenylacetic acid degradation protein PaaD
BTH_I3109	transcriptional regulator, AraC family
BTH_I3124	lipoprotein, putative
BTH_I3126	hypothetical protein
BTH_I3128	hypothetical protein
BTH_I3130	integrase, putative
BTH_I3131	transposase, Mutator family
BTH_I3132	site-specific recombinase, phage integrase family
BTH_I3133	conserved hypothetical protein
BTH_I3134	DNA-binding protein
BTH_I3135	resolvase TnpR
BTH_I3136	conserved hypothetical protein
BTH_I3137	hypothetical protein
BTH_I3138	transposase, Mutator family
BTH_I3139	helicase domain protein
BTH_I3140	TnpC protein
BTH_I3142	L0013 protein
BTH_I3143	helicase domain protein
BTH_I3145	cytochrome c family protein
BTH_I3171	phytoene dehydrogenase, putative
BTH_I3195	ribosomal protein S21-related protein
BTH_I3199	hypothetical protein
BTH_I3200	hypothetical protein
BTH_I3203	hypothetical protein
BTH_I3208	conserved hypothetical protein
BTH_I3210	TPR domain protein
BTH_I3211	aminotransferase, DegT/DnrJ/EryC1/StrS family
BTH_I3212	WbnG
BTH_I3213	conserved hypothetical protein
BTH_I3214	transferase, putative
BTH_I3215	conserved hypothetical protein
BTH_I3216	streptogramin acetyl transferase
BTH_I3217	hypothetical protein
BTH_I3229	transposase, truncation
BTH_I3230	hypothetical protein
BTH_I3232	site-specific recombinase, phage integrase family
BTH_I3233	tRNA modification GTPase TrmE
BTH_I3242	conserved hypothetical protein
BTH_I3243	conserved hypothetical protein
BTH_I3244	transposase, Mutator family
BTH_I3245	transcriptional regulator, LysR family
BTH_I3246	drug resistance transporter, EmrB/QacA subfamily, putative

BTH_I3255	glycine cleavage system T protein
BTH_I3258	GDSL-like Lipase/Acylhydrolase domain protein
BTH_I3259	membrane protein, putative
BTH_I3265	hypothetical protein
BTH_I3266	hypothetical protein
BTH_I3267	hypothetical protein
BTH_I3268	32.7 kDa protein (ORF 301)
BTH_I3269	probable phage-related secreted protein YPO2280
BTH_I3270	hypothetical protein
BTH_I3272	Transposase (IS4 family)
BTH_I3273	transposase, putative
BTH_I3278	site-specific recombinase, phage integrase family
BTH_I3279	transposase, Mutator family
BTH_I3280	transcriptional regulator, LysR family
BTH_I3284	acyltransferase family protein
BTH_I3285	hypothetical protein
BTH_I3287	hypothetical protein
BTH_I3288	hypothetical protein
BTH_I3289	unnamed protein product
BTH_I3293	probable oxidoreductase
BTH_I3296	hypothetical protein
BTH_I3325	hypothetical protein
BTH_I3326	hypothetical protein
BTH_I3341	SET domain protein
BTH_II0007	K+-transporting ATPase, A subunit
BTH_II0008	acetyltransferase, GNAT family family
BTH_II0022	hypothetical protein
BTH_II0025	hypothetical protein
BTH_II0026	cytochrome P450-related protein
BTH_II0029	hypothetical protein
BTH_II0031	conserved hypothetical protein
BTH_II0032	hypothetical protein
BTH_II0037	hypothetical protein
BTH_II0039	conserved hypothetical protein
BTH_II0053	LysE family protein
BTH_II0054	thioredoxin, putative
BTH_II0055	sulfide:quinone oxidoreductase
BTH_II0056	PROBABLE PORIN
BTH_II0057	conserved hypothetical protein
BTH_II0058	transporter, AcrB/D/F family
BTH_II0059	efflux transporter, RND family, MFP subunit, putative
BTH_II0060	ArsR family regulatory protein
BTH_II0061	Hypothetical protein
BTH_II0062	ABC transporter, ATP-binding protein
BTH_II0063	permease, putative domain protein
BTH_II0064	efflux transporter, RND family, MFP subunit subfamily, putative
BTH_II0065	creA protein, putative
BTH_II0066	hypothetical protein
BTH_II0067	CybP

BTH_II0068	DNA-binding response regulator
BTH_II0069	cation efflux family protein
BTH_II0070	PalA
BTH_II0071	PROBABLE HEMAGGLUTININ-RELATED PROTEIN
BTH_II0072	hemolysin activator protein, HlyB family, putative
BTH_II0079	IS407A, transposase OrfA
BTH_II0080	transposase subunit
BTH_II0081	transposase subunit
BTH_II0082	Hypothetical protein
BTH_II0083	acetyltransferase, GNAT family
BTH_II0084	putrescine ABC transporter, permease protein
BTH_II0085	ABC transporter, permease protein
BTH_II0086	putrescine ABC transporter, periplasmic putrescine-binding protein, putative
BTH_II0087	transposase
BTH_II0091	conserved hypothetical protein
BTH_II0093	integrase, putative
BTH_II0094	conserved hypothetical protein
BTH_II0095	transposase
BTH_II0096	transposase
BTH_II0102	conserved hypothetical protein
BTH_II0103	outer membrane porin OpcP
BTH_II0104	transcriptional regulator, LysR family
BTH_II0105	hypothetical protein
BTH_II0106	hypothetical protein
BTH_II0107	transcriptional regulator, LysR family
BTH_II0108	outer membrane efflux protein
BTH_II0109	possible FusE-MFP/HlyD family membrane fusion protein
BTH_II0110	fusaric acid resistance protein, putative
BTH_II0111	hypothetical protein
BTH_II0112	Hep_Hag family
BTH_II0114	YfaZ precursor superfamily
BTH_II0115	frimbrial protein
BTH_II0119	hypothetical protein
BTH_II0121	Protein of unknown function (DUF770) superfamily
BTH_II0122	Protein of unknown function (DUF877) superfamily
BTH_II0133	Fimbrial protein subfamily, putative
BTH_II0139	conserved hypothetical protein
BTH_II0143	capsula synthesis response regulator transcription regulator protein
BTH_II0144	sensor protein evgS precursor
BTH_II0145	glycosyl transferase, group 2 family protein domain protein
BTH_II0146	hypothetical protein
BTH_II0147	conserved hypothetical protein
BTH_II0148	HAD-superfamily hydrolase, subfamily IB (PSPase-like) subfamily, putative
BTH_II0149	conserved hypothetical protein
BTH_II0150	hypothetical protein
BTH_II0151	flagellin D
BTH_II0152	putative membrane protein
BTH_II0153	chemotaxis MotA protein
BTH_II0154	Chemotaxis lafU protein

BTH_II0155	chemotaxis response regulator
BTH_II0156	chemotaxis protein CheA
BTH_II0157	chemotaxis protein CheW
BTH_II0158	chemotaxis protein
BTH_II0159	chemotaxis protein methyltransferase CheR
BTH_II0160	chemotaxis protein CheD
BTH_II0161	protein-glutamate methylesterase CheB
BTH_II0162	protein-glutamate methylesterase CheB
BTH_II0163	flagellar biosynthesis protein FlhA
BTH_II0164	flagellar biosynthetic protein FlhB
BTH_II0165	flagellar biosynthetic protein FliR, putative
BTH_II0166	flagellar biosynthetic protein FliQ, putative
BTH_II0167	flagellar biosynthetic protein fliP
BTH_II0168	flagellar motor switch protein FliN
BTH_II0169	conserved hypothetical protein
BTH_II0170	Flagellar hook-basal body complex protein FliE
BTH_II0171	flagellar M-ring protein FliF
BTH_II0172	flagellar motor switch protein FliG, putative
BTH_II0173	Flagellar assembly protein FliH
BTH_II0174	flagellum-specific ATP synthase FliI
BTH_II0175	conserved hypothetical protein
BTH_II0176	flagellar hook-associated protein, putative
BTH_II0177	flagellar protein FliS
BTH_II0178	hypothetical protein
BTH_II0179	Flagellar hook-length control protein, putative
BTH_II0180	RNA polymerase sigma factor for flagellar operon FliA
BTH_II0181	conserved hypothetical protein
BTH_II0182	flagellar protein FlgJ
BTH_II0183	flagella basal body P-ring formation protein FlgA
BTH_II0184	flagellar basal-body rod protein FlgB
BTH_II0185	flagellar basal-body rod protein FlgC
BTH_II0186	basal-body rod modification protein FlgD, putative
BTH_II0187	flagellar hook protein flgE
BTH_II0188	flagellar basal-body rod protein FlgF
BTH_II0189	flagellar basal-body rod protein FlgG
BTH_II0190	flagellar L-ring protein FlgH
BTH_II0191	flagellar P-ring protein FlgI
BTH_II0192	peptidoglycan hydrolase
BTH_II0193	flagellar hook-associated protein 1, putative
BTH_II0194	flagellar hook-associated protein 3, putative
BTH_II0195	conserved hypothetical protein
BTH_II0196	hypothetical protein
BTH_II0197	transcriptional regulator, putative
BTH_II0208	PROBABLE TRANSMEMBRANE PROTEIN
BTH_II0215	outer membrane porin OpcP
BTH_II0221	hypothetical protein
BTH_II0225	oxidoreductase, short-chain dehydrogenase/reductase family
BTH_II0234	class III extradiol-type catecholic dioxygenase, putative
BTH_II0235	transcriptional regulator, LysR family

BTH_II0236	conserved hypothetical protein
BTH_II0237	pdhA
BTH_II0238	pyruvate dehydrogenase E1 beta subunit
BTH_II0239	dihydrolipoamide acyltransferase
BTH_II0240	L-lactate dehydrogenase
BTH_II0241	major facilitator family transporter
BTH_II0242	isochorismatase, isochorismatase family
BTH_II0243	transcriptional regulator, LysR family
BTH_II0249	FHA domain protein
BTH_II0255	conserved hypothetical protein
BTH_II0256	serine/threonine protein kinase
BTH_II0263	Protein of unknown function (DUF1305) family
BTH_II0266	conserved hypothetical protein
BTH_II0267	rhsD protein
BTH_II0268	ISRSO7-TRANSPOSASE PROTEIN
BTH_II0269	ISRSO7-TRANSPOSASE PROTEIN
BTH_II0270	transposase fragment
BTH_II0271	transposase, Mutator family
BTH_II0272	HAD-superfamily hydrolase
BTH_II0282	transcriptional regulator, AraC family
BTH_II0283	isochorismatase family protein family
BTH_II0294	PUTATIVE TRANSCRIPTION REGULATOR PROTEIN, putative
BTH_II0295	drug resistance transporter, EmrB/QacA family
BTH_II0296	moeZ
BTH_II0297	lpqC, putative
BTH_II0298	hypothetical protein
BTH_II0299	unnamed protein product; Highly similar to oxygen-independent coproporphyrinogen III oxidase, YggW protein of Escherichia coli, pu
BTH_II0300	bcp
BTH_II0301	methlytransferase, UbiE/COQ5 family
BTH_II0302	Uncharacterized conserved protein
BTH_II0303	conserved hypothetical protein
BTH_II0304	ThiS family domain protein, putative
BTH_II0305	probable isovaleryl-CoA dehydrogenase, putative
BTH_II0306	hypothetical protein
BTH_II0307	carbohydrate kinase, FGGY family, putative
BTH_II0308	hypothetical protein
BTH_II0309	Sphingosine-1-phosphate lyase (SP-lyase) (SPL)(Sphingosine-1-phosphate aldolase)
BTH_II0311	Sphingosine-1-phosphate lyase 1 (SP-lyase) (SPL)(Sphingosine-1-phosphate aldolase)
BTH_II0312	Protein of unknown function (DUF636) family
BTH_II0313	conserved within P. aerophilum
BTH_II0316	4-hydroxyphenylacetate 3-monooxygenase, reductase component
BTH_II0317	DNA-binding response regulator, LuxR family
BTH_II0318	putative oxidoreductase, oxygen dependent, FAD-dependent protein
BTH_II0319	3-oxoacyl-acyl carrier protein reductase
BTH_II0320	pyrazinamidase/nicotinamidase
BTH_II0321	hypothetical protein
BTH_II0322	hypothetical protein
BTH_II0323	phenol hydroxylase, putative
BTH_II0324	sensory box histidine kinase, putative

BTH_II0325	putative tautomerase
BTH_II0326	Ring hydroxylating beta subunit family
BTH_II0327	benzoate 1,2-dioxygenase, alpha subunit
BTH_II0328	transcriptional regulator, LysR family
BTH_II0329	oxidoreductase
BTH_II0330	phenylacetaldehyde dehydrogenase
BTH_II0331	GMC oxidoreductase
BTH_II0332	MFS transporter, phthalate permease family, putative
BTH_II0333	outer membrane porin, putative
BTH_II0334	transcriptional regulator, LysR family
BTH_II0335	transcriptional regulator, AraC family
BTH_II0336	conserved hypothetical protein
BTH_II0337	3-hydroxyacyl-CoA dehydrogenase, putative
BTH_II0338	major facilitator family transporter
BTH_II0341	ribosomal protein L15
BTH_II0342	glycosyl transferase, group 2 family protein, putative
BTH_II0344	lipoprotein, putative
BTH_II0349	PROBABLE GLUCONATE 5-DEHYDROGENASE OXIDOREDUCTASE PROTEIN
BTH_II0350	transcriptional regulator, LysR family
BTH_II0351	conserved hypothetical protein
BTH_II0352	thermoresistant gluconokinase
BTH_II0353	major facilitator family transporter
BTH_II0354	transposase, putative
BTH_II0355	Transposase (IS4 family)
BTH_II0356	IS407A, transposase OrfA
BTH_II0357	transposase fragment
BTH_II0358	transposase, Mutator family
BTH_II0359	conserved hypothetical protein
BTH_II0360	carboxymuconolactone decarboxylase
BTH_II0361	conserved hypothetical protein
BTH_II0362	succinate dehydrogenase, iron-sulfur protein
BTH_II0363	conserved hypothetical protein
BTH_II0364	Protein of unknown function, DUF488 superfamily
BTH_II0365	RC180
BTH_II0366	transposase
BTH_II0367	transposase
BTH_II0368	conserved hypothetical protein
BTH_II0369	hypothetical protein
BTH_II0370	conserved hypothetical protein
BTH_II0372	beta-lactamase
BTH_II0377	hypothetical protein
BTH_II0378	hypothetical protein
BTH_II0392	HYPOTHETICAL SIGNAL PEPTIDE PROTEIN
BTH_II0408	Pyridoxamine 5'-phosphate oxidase family
BTH_II0415	6-phosphofructokinase
BTH_II0418	poly-beta-hydroxybutyrate polymerase
BTH_II0421	ATP synthase gene 1, putative
BTH_II0429	hypothetical protein
BTH_II0430	conserved hypothetical protein

BTH_II0431	transposase, Mutator family
BTH_II0432	hypothetical protein
BTH_II0433	hypothetical protein
BTH_II0434	cytochrome P450-related protein
BTH_II0435	transposase, putative
BTH_II0436	Transposase (IS4 family)
BTH_II0438	RND efflux system, cytoplasmic membrane extrusion protein
BTH_II0441	transcriptional regulator, TetR family
BTH_II0447	RND efflux system, outer membrane lipoprotein, NodT family
BTH_II0448	polysaccharide deacetylase domain protein
BTH_II0450	adenylylsulfate kinase
BTH_II0451	conserved hypothetical protein
BTH_II0457	cytochrome c family protein
BTH_II0462	metallo-beta-lactamase family protein
BTH_II0469	carboxylesterase family protein
BTH_II0475	conserved hypothetical protein
BTH_II0476	transcriptional regulator lysR family
BTH_II0494	acetyltransferase, GNAT family
BTH_II0499	conserved hypothetical protein
BTH_II0503	CheR methyltransferase, SAM binding/TPR domain protein
BTH_II0505	chemotaxis sensor histidine kinase, putative
BTH_II0509	CAAX amino terminal protease family protein
BTH_II0510	conserved hypothetical protein
BTH_II0512	tryptophanyl-tRNA synthetase
BTH_II0513	transcriptional regulator, AraC family
BTH_II0516	dyp-type peroxidase family protein
BTH_II0526	major facilitator family transporter
BTH_II0535	conserved hypothetical protein
BTH_II0556	conserved hypothetical protein
BTH_II0573	Transposase (IS4 family)
BTH_II0574	transposase, putative
BTH_II0578	hypothetical protein
BTH_II0580	ebsC protein, putative
BTH_II0589	CHAD domain family
BTH_II0598	transcriptional regulator, MarR family
BTH_II0603	hypothetical protein
BTH_II0607	conserved hypothetical protein
BTH_II0623	Bbp50
BTH_II0624	Putative transposase Rv3428c
BTH_II0625	transposase, Mutator family
BTH_II0637	conserved hypothetical protein
BTH_II0647	hypothetical protein
BTH_II0648	hypothetical protein
BTH_II0650	conserved hypothetical protein
BTH_II0668	hypothetical protein
BTH_II0676	probable transmembrane protein
BTH_II0692	hypothetical protein
BTH_II0696	hypothetical protein
BTH_II0702	outer membrane porin OpcP

BTH_II0705	conserved hypothetical protein
BTH_II0713	hypothetical protein
BTH_II0714	integrase, putative
BTH_II0716	transposase
BTH_II0717	transposase
BTH_II0718	hypothetical protein
BTH_II0719	streptavidin,putative
BTH_II0720	hypothetical protein
BTH_II0721	glutamyl-tRNA, putative
BTH_II0724	transcriptional regulator, IclR family, putative
BTH_II0726	4-hydroxybenzoate transporter
BTH_II0729	conserved hypothetical protein
BTH_II0734	conserved hypothetical protein
BTH_II0737	syringomycin biosynthesis enzyme, putative
BTH_II0738	Transposase (IS4 family)
BTH_II0739	transposase, putative
BTH_II0740	hypothetical protein
BTH_II0741	conserved hypothetical protein
BTH_II0746	conserved hypothetical protein
BTH_II0748	HrpB2-like protein
BTH_II0749	conserved hypothetical protein
BTH_II0753	Surface presentation of antigens (SPOA) protein domain protein
BTH_II0756	conserved hypothetical protein
BTH_II0757	hypothetical protein
BTH_II0760	hypothetical protein
BTH_II0763	hrp protein, putative
BTH_II0764	conserved hypothetical protein
BTH_II0767	type II/III secretion system family protein
BTH_II0774	type IV pilus biogenesis protein, putative
BTH_II0776	type IV prepilin
BTH_II0778	hypothetical protein
BTH_II0779	hypothetical protein
BTH_II0780	hypothetical protein
BTH_II0786	hypothetical protein
BTH_II0787	predicted ATP-dependent protease
BTH_II0788	hypothetical protein
BTH_II0789	hipB domain protein
BTH_II0790	conserved hypothetical protein
BTH_II0792	endo-1,4-D-glucanase
BTH_II0793	cellulose synthase operon protein C
BTH_II0794	conserved hypothetical protein
BTH_II0795	hypothetical protein
BTH_II0796	cellulose biosynthesis protein, putative
BTH_II0799	alpha-ketoglutarate-dependent taurine dioxygenase
BTH_II0804	N-acyl homoserine lactone synthase
BTH_II0808	conserved hypothetical protein
BTH_II0810	LysE family protein
BTH_II0817	conserved hypothetical protein
BTH_II0819	transcriptional regulator, putative

BTH_II0820	hypothetical protein
BTH_II0821	conserved hypothetical protein
BTH_II0826	type III secretion system protein BsaM
BTH_II0834	BsaU protein
BTH_II0845	conserved hypothetical protein
BTH_II0853	transcriptional regulator, araC family
BTH_II0854	ubiquitin-specific proteinase 31, putative
BTH_II0855	lipoprotein, putative
BTH_II0858	conserved hypothetical protein
BTH_II0860	lipoprotein, putative
BTH_II0861	pentapeptide repeat family protein, putative
BTH_II0862	pentapeptide repeat family protein
BTH_II0864	ATP-dependent Clp protease, ATP-binding subunit ClpB
BTH_II0867	Protein of unknown function (DUF1316) subfamily, putative
BTH_II0868	hcp protein
BTH_II0873	ImpA-related N-terminal family
BTH_II0874	conserved hypothetical protein
BTH_II0875	Hep_Hag family
BTH_II0877	N-acetylmuramoyl-L-alanine amidase domain protein
BTH_II0878	hemagglutinin domain protein
BTH_II0885	TetR-family transcriptional regulator
BTH_II0889	transcriptional regulator, DeoR family
BTH_II0892	oxidoreductase, FAD-binding
BTH_II0893	addiction module antitoxin, Axe family subfamily, putative
BTH_II0895	conserved hypothetical protein
BTH_II0899	ApbE family protein
BTH_II0900	nitrate reductase/sulfite reductase flavoprotein alpha-component, putative
BTH_II0901	conserved hypothetical protein
BTH_II0905	transcriptional regulator lacI family
BTH_II0906	PTS system, glucose-specific EIIA/HPr/phosphoenolpyruvate-protein phosphotransferase components
BTH_II0907	1-phosphofructokinase
BTH_II0908	protein-N p-phosphohistidine-sugar phosphotransferase
BTH_II0909	acetyltransferase, GNAT family
BTH_II0912	hypothetical exported protein
BTH_II0913	O-methyltransferase
BTH_II0914	transcriptional regulatory protein
BTH_II0915	hypothetical protein
BTH_II0916	chaperonin GroEL
BTH_II0917	conserved hypothetical protein
BTH_II0918	ribose-phosphate pyrophosphokinase
BTH_II0919	glutamine-dependent NAD+ synthetase
BTH_II0920	CBS domain protein
BTH_II0921	conserved hypothetical protein
BTH_II0923	hypothetical protein
BTH_II0924	heat shock protein, HSP20 family
BTH_II0925	beta-lactamase, putative
BTH_II0926	conserved hypothetical protein
BTH_II0927	acetyl-CoA synthetase
BTH_II0928	probable pyruvate dehydrogenase, E1 component, alpha subunit

BTH_II0929	pyruvate dehydrogenase complex, E1 component, pyruvate dehydrogenase beta subunit
BTH_II0930	probable pyruvate dehydrogenase, E2 component, dihydrolipoamide acetyltransferase
BTH_II0931	Predicted orf
BTH_II0932	DNA-binding response regulator, LuxR family, putative
BTH_II0933	Rhs element Vgr protein, putative
BTH_II0934	conserved hypothetical protein
BTH_II0935	transposase, putative
BTH_II0936	Transposase (IS4 family)
BTH_II0937	conserved hypothetical protein
BTH_II0938	glycogen operon protein GlgX
BTH_II0939	1,4-alpha-glucan branching enzyme (Glycogen branchingenzyme) (BE) (1,4-alpha-D-glucan:1,4-alpha-D-glucan 6-glucosyl-transferase)
BTH_II0940	glycogen synthase
BTH_II0941	glycogen phosphorylase family protein
BTH_II0942	conserved hypothetical protein
BTH_II0943	AMP-binding domain protein
BTH_II0954	YadA-like C-terminal region protein
BTH_II0955	TPR Domain domain protein
BTH_II0956	conserved hypothetical protein
BTH_II0957	outer membrane protein, putative
BTH_II0960	glyoxalase family protein family
BTH_II0963	conserved hypothetical protein
BTH_II0964	hypothetical protein
BTH_II0966	glycine betaine/L-proline ABC transporter, ATP-binding protein
BTH_II0970	hypothetical protein
BTH_II0971	Aspartyl/Asparaginyl beta-hydroxylase family
BTH_II0981	hydrolase, putative
BTH_II0982	D-3-phosphoglycerate dehydrogenase, putative
BTH_II0983	ribose operon repressor, putative
BTH_II0984	TRAP transporter, DctM subunit subfamily, putative
BTH_II0985	Bacterial extracellular solute-binding protein, family 7 superfamily
BTH_II0986	hypothetical protein
BTH_II0987	possible glutathione S-transferase P subunit
BTH_II0988	Transposase (IS4 family)
BTH_II0989	transposase, putative
BTH_II0991	transcriptional regulator, AraC family
BTH_II0993	hypothetical protein
BTH_II0994	transcriptional regulator, AraC family, putative
BTH_II1005	glyoxylate reductase
BTH_II1007	sensory box histidine kinase
BTH_II1011	integrase-like protein
BTH_II1012	gp38
BTH_II1013	gp44
BTH_II1014	gp45
BTH_II1015	gp41
BTH_II1016	gp42
BTH_II1019	gp40-related protein
BTH_II1020	hypothetical protein
BTH_II1022	gp50
BTH_II1023	gp51

BTH_II1024	gp52
BTH_II1025	gp63
BTH_II1026	gp64
BTH_II1027	gp65
BTH_II1028	MTE8-ECOLI homolog similar to methyltransferase
BTH_II1029	gp56
BTH_II1030	gp57
BTH_II1031	gp58
BTH_II1032	gp69
BTH_II1033	gp60
BTH_II1034	gp72
BTH_II1035	gp62
BTH_II1036	gp74
BTH_II1037	gp64
BTH_II1038	gp65
BTH_II1039	hypothetical protein
BTH_II1040	pANL56
BTH_II1042	59R
BTH_II1043	conserved hypothetical phage protein
BTH_II1044	phage terminase, large subunit, putative
BTH_II1045	head portal protein
BTH_II1046	ClpP protease
BTH_II1047	phage major capsid protein, HK97 family
BTH_II1048	hypothetical protein
BTH_II1049	gp7
BTH_II1050	phage head-tail adaptor, putative
BTH_II1051	phage protein, HK97 gp10 family
BTH_II1052	gp10
BTH_II1053	gp11
BTH_II1054	Phage tail assembly chaperone
BTH_II1055	gp13
BTH_II1056	gp14
BTH_II1057	Phage minor tail protein
BTH_II1058	gp16
BTH_II1059	phage minor tail protein L
BTH_II1060	gp19
BTH_II1061	Bacteriophage lambda tail assembly protein I
BTH_II1062	host specificity protein J, truncation
BTH_II1063	gp21
BTH_II1064	gp22
BTH_II1065	holin
BTH_II1066	conserved hypothetical protein
BTH_II1067	gp23
BTH_II1068	gp26
BTH_II1069	gp28
BTH_II1070	MFS transporter, putative
BTH_II1078	hypothetical protein
BTH_II1082	transposase, Mutator family
BTH_II1090	conserved hypothetical protein

BTH_II1092	di-haem cytochrome c peroxidase family protein
BTH_II1095	major facilitator family transporter
BTH_II1098	hydrolase, haloacid dehalogenase-like family, putative
BTH_II1102	hypothetical protein
BTH_II1103	hypothetical protein
BTH_II1104	conserved hypothetical protein
BTH_II1107	conserved hypothetical protein
BTH_II1116	D-aminopeptidase, putative
BTH_II1121	conserved hypothetical protein
BTH_II1125	hypothetical protein
BTH_II1127	GGDEF domain protein
BTH_II1128	conserved hypothetical protein
BTH_II1130	ATP-dependent helicase, DEAD/DEAH family
BTH_II1131	hypothetical protein
BTH_II1132	conserved hypothetical protein
BTH_II1136	conserved hypothetical protein
BTH_II1137	hypothetical protein
BTH_II1143	hypothetical protein
BTH_II1145	membrane protein
BTH_II1147	conserved hypothetical protein
BTH_II1149	Domain of unknown function (DUF802) family
BTH_II1150	conserved hypothetical protein
BTH_II1152	hypothetical protein
BTH_II1153	hypothetical protein
BTH_II1157	aspartate racemase
BTH_II1158	transcriptional regulator, LysR family
BTH_II1159	ABC transporter, periplasmic substrate-binding protein, putative
BTH_II1160	alcohol dehydrogenase, zinc-containing
BTH_II1168	uroporphyrin-III C-methyltransferase
BTH_II1183	DedA family family
BTH_II1184	heavy metal efflux pump CzcA, putative
BTH_II1185	metal ion efflux membrane fusion protein family, putative
BTH_II1186	divalent cation resistant determinant protein C, putative
BTH_II1187	conserved hypothetical protein
BTH_II1188	sensor histidine kinase, putative
BTH_II1189	response regulator, interruption-N
BTH_II1193	FF domain protein
BTH_II1200	isochorismatase family protein
BTH_II1206	conserved hypothetical protein
BTH_II1208	transcriptional regulator, GntR family
BTH_II1211	polyketide synthase
BTH_II1213	peptide synthetase homolog
BTH_II1220	transcriptional regulator, IclR family
BTH_II1221	metabolite-proton symporter
BTH_II1222	4-hydroxyphenylpyruvate dioxygenase
BTH_II1228	conserved hypothetical protein
BTH_II1230	hypothetical protein
BTH_II1233	peptide synthetase, putative
BTH_II1237	thiotemplate mechanism natural product synthetase

BTH_II1244	transcriptional regulator, Crp/Fnr family
BTH_II1246	hypothetical protein
BTH_II1247	DNA-binding response regulator, LuxR family
BTH_II1255	conserved hypothetical protein
BTH_II1265	POSSIBLE FORMATE HYDROGENASE HYCQ
BTH_II1267	conserved hypothetical protein
BTH_II1273	2,4-dienoyl-CoA reductase
BTH_II1291	HlyD family secretion protein
BTH_II1292	ABC transporter, ATP binding/permease protein
BTH_II1293	ABC transporter, ATP-binding protein
BTH_II1294	ABC transporter, ATP-binding protein
BTH_II1295	ABC transporter permease protein
BTH_II1296	conserved hypothetical protein
BTH_II1297	conserved hypothetical protein
BTH_II1300	amino acid permease
BTH_II1308	putative exported protein
BTH_II1311	Mg2+-importing ATPase, putative
BTH_II1312	conserved hypothetical protein
BTH_II1313	Uncharacterized conserved protein
BTH_II1314	Uncharacterized conserved protein
BTH_II1315	conserved hypothetical protein
BTH_II1316	hypothetical protein
BTH_II1317	hypothetical protein
BTH_II1323	ProP effector
BTH_II1327	PROBABLE PHAGE-RELATED TAIL TRANSMEMBRANE PROTEIN
BTH_II1354	pANL56
BTH_II1355	pANL12
BTH_II1364	DNA methyltransferase XF1774
BTH_II1365	gp51
BTH_II1370	cystathionine beta-synthase
BTH_II1372	hypothetical protein
BTH_II1373	heavy metal resistance protein CzcC
BTH_II1378	conserved hypothetical protein
BTH_II1384	conserved hypothetical protein
BTH_II1385	YceI like family protein
BTH_II1386	conserved hypothetical protein
BTH_II1390	CAAX protease family protein
BTH_II1392	phenylacetate-CoA ligase
BTH_II1396	conserved hypothetical protein
BTH_II1398	conserved hypothetical protein
BTH_II1399	methyltransferase, FkbM family domain protein
BTH_II1400	response regulator
BTH_II1402	transposase fragment
BTH_II1405	conserved hypothetical protein
BTH_II1411	membrane protein, putative
BTH_II1419	conserved hypothetical protein
BTH_II1420	conserved hypothetical protein
BTH_II1424	conserved hypothetical protein
BTH_II1430	hypothetical protein

BTH_II1431	conserved hypothetical protein
BTH_II1433	conserved hypothetical protein
BTH_II1434	YD repeat protein
BTH_II1435	conserved hypothetical protein
BTH_II1437	conserved hypothetical protein
BTH_II1438	hypothetical protein
BTH_II1439	hypothetical protein
BTH_II1447	transcriptional regulator, LysR family, putative
BTH_II1451	lipoprotein NlpD, putative
BTH_II1452	hypothetical protein
BTH_II1466	membrane protein, putative
BTH_II1475	transcriptional regulator, LysR family
BTH_II1476	6-aminohexanoate-cyclic-dimer hydrolase
BTH_II1479	Protein of unknown function (DUF1089) superfamily
BTH_II1480	hypothetical protein
BTH_II1482	hypothetical protein
BTH_II1518	transposase, putative
BTH_II1519	Transposase (IS4 family)
BTH_II1521	Tat (twin-arginine translocation) pathway signal sequence domain protein
BTH_II1522	transcriptional regulator, ArsR family
BTH_II1524	acetyltransferase, GNAT family
BTH_II1525	transporter, putative
BTH_II1526	phage recombinase, putative
BTH_II1527	conserved hypothetical protein
BTH_II1528	transposase, Mutator family
BTH_II1529	conserved hypothetical protein
BTH_II1530	conserved domain protein
BTH_II1531	Rhs element Vgr protein, putative
BTH_II1534	hypothetical protein
BTH_II1537	phenylacetaldehyde dehydrogenase
BTH_II1538	transcriptional regulator, LuxR family
BTH_II1540	conserved hypothetical protein
BTH_II1546	N-carbamyl-L-amino acid amidohydrolase
BTH_II1547	L-2-Amino-thiazoline-4-carboxylic acid hydrolase
BTH_II1548	amino acid ABC transporter, periplasmic amino acid-binding protein, putative
BTH_II1549	glycine cleavage system transcriptional activator
BTH_II1552	hypothetical protein
BTH_II1553	hypothetical protein
BTH_II1557	conserved hypothetical protein
BTH_II1561	hypothetical protein
BTH_II1577	conserved hypothetical protein
BTH_II1579	OmpA family domain protein
BTH_II1580	conserved hypothetical protein
BTH_II1581	hypothetical protein
BTH_II1582	conserved hypothetical protein
BTH_II1584	penicillin-binding protein 1C
BTH_II1585	hypothetical protein
BTH_II1596	conserved hypothetical protein
BTH_II1605	hydrolase

BTH_II1612	redox-sensitive transcriptional activator SoxR
BTH_II1613	efflux transporter, RND family, MFP subunit
BTH_II1615	transcriptional regulator, LysR family
BTH_II1616	C4-dicarboxylate transporter/malic acid transport protein, putative
BTH_II1617	malate dehydrogenase
BTH_II1618	PROBABLE CYTOCHROME C OXIDASE SUBUNIT TRANSMEMBRANE PROTEIN
BTH_II1619	FixOc
BTH_II1620	outer membrane nitrite reductase, putative
BTH_II1621	copper-translocating P-type ATPase
BTH_II1622	sulfite:cytochrome c oxidoreductase subunit A
BTH_II1623	conserved hypothetical protein
BTH_II1626	L-arabinose ABC transporter, permease protein
BTH_II1627	L-arabinose ABC transporter, ATP-binding protein
BTH_II1628	L-arabinose ABC transporter, periplasmic L-arabinose-binding protein
BTH_II1629	dehydrogenase
BTH_II1630	dihydrodipicolinate synthase, putative
BTH_II1631	aldehyde dehydrogenase family protein
BTH_II1632	dihydroxy-acid dehydratase
BTH_II1633	transcriptional regulator, LysR family, putative
BTH_II1642	glyoxalase family protein
BTH_II1644	conserved hypothetical protein
BTH_II1645	hypothetical protein
BTH_II1654	lipoprotein NlpD, putative
BTH_II1655	glutamate/aspartate ABC transporter, periplasmic glutamate/aspartate-binding protein, truncation
BTH_II1662	hypothetical protein
BTH_II1663	pyruvate ferredoxin/flavodoxin oxidoreductase family protein
BTH_II1664	polyketide synthase, putative
BTH_II1665	polyketide synthase, putative
BTH_II1666	polyketide synthase, putative
BTH_II1667	polyketide synthase, putative
BTH_II1668	polyketide biosynthesis enoyl-CoA hydratase
BTH_II1669	polyketide biosynthesis enoyl-CoA hydratase
BTH_II1670	polyketide biosynthesis protein, interruption-N
BTH_II1671	polyketide beta-ketoacyl synthase, putative
BTH_II1672	acyl carier protein-related protein
BTH_II1673	thiotemplate mechanism natural product synthetase
BTH_II1674	polyketide synthase
BTH_II1675	MmpIII
BTH_II1676	4-phosphopantetheinyl transferase family protein
BTH_II1677	PHENOLPTHIOCEROL SYNTHESIS TYPE-I POLYKETIDE SYNTHASE PPSA
BTH_II1678	Ser/Thr protein phosphatase family
BTH_II1679	acetyl-CoA carboxylase, carboxyl transferase, alpha subunit
BTH_II1680	hypothetical protein
BTH_II1681	autoinducer-binding transcriptional regulator, LuxR family, putative
BTH_II1682	hypothetical protein
BTH_II1685	conserved hypothetical protein
BTH_II1687	cyclic nucleotide-binding domain protein
BTH_II1688	transcriptional regulator, AraC family, putative
BTH_II1689	hypothetical protein

BTH_II1690	LysE family protein, putative
BTH_II1691	multidrug efflux membrane protein, putative
BTH_II1694	D-beta-hydroxybutyrate dehydrogenase
BTH_II1697	acetyltransferase, GNAT family , putative
BTH_II1701	hypothetical protein
BTH_II1702	transcriptional regulator, TetR family
BTH_II1703	uncharacterized domain protein
BTH_II1704	MmcH, putative
BTH_II1705	oxidoreductase, 2OG-Fe(II) oxygenase family superfamily
BTH_II1706	radical SAM domain protein protein
BTH_II1707	radical SAM domain protein protein
BTH_II1708	unnamed protein product
BTH_II1709	ThiS family domain protein, putative
BTH_II1710	carbamoyltransferase family protein
BTH_II1711	conserved hypothetical protein
BTH_II1714	Domain of unknown function
BTH_II1716	hypothetical protein
BTH_II1718	hypothetical protein
BTH_II1719	hypothetical protein
BTH_II1721	hypothetical protein
BTH_II1722	MOSC domain protein
BTH_II1724	DNA-binding response regulator TctD
BTH_II1738	MltA-interacting protein MipA family
BTH_II1744	conserved hypothetical protein
BTH_II1751	transcriptional regulator, LuxR family
BTH_II1756	acetyltransferase (GNAT) family protein
BTH_II1757	membrane protein, putative
BTH_II1765	DNA-binding response regulator
BTH_II1772	HD domain protein
BTH_II1773	HD domain protein
BTH_II1774	serine protease, subtilase family
BTH_II1775	hypothetical protein
BTH_II1776	hypothetical protein
BTH_II1777	hypothetical protein
BTH_II1778	cytochrome c
BTH_II1779	cytochrome c family protein
BTH_II1781	outer membrane protein, OMP85 family, putative
BTH_II1784	glutaminase A
BTH_II1785	transposase, mutator family, truncation-related protein
BTH_II1786	conserved hypothetical protein
BTH_II1787	gp26
BTH_II1788	glutaminase A
BTH_II1794	transcriptional regulator, TetR family domain protein
BTH_II1795	long-chain-fatty-acid--CoA ligase
BTH_II1796	long-chain-fatty-acid--CoA ligase
BTH_II1797	acyl-CoA synthetase
BTH_II1811	hypothetical protein
BTH_II1813	conserved hypothetical protein
BTH_II1815	hypothetical protein

BTH_II1817	putative integral membrane protein
BTH_II1827	pyochelin synthetase PA4225
BTH_II1828	pyochelin synthetase
BTH_II1844	hypothetical protein
BTH_II1873	levanase
BTH_II1874	conserved hypothetical protein
BTH_II1878	hypothetical protein
BTH_II1903	coenzyme A transferase
BTH_II1911	DJ-1/PfpI family protein
BTH_II1914	hydrolase, alpha/beta fold family
BTH_II1924	conserved hypothetical protein
BTH_II1927	alkyl hydroperoxide reductase subunit
BTH_II1938	chaperonin GroEL
BTH_II1940	conserved hypothetical protein
BTH_II1945	glutamine amidotransferase, class I
BTH_II1951	putrescine ABC transporter, permease protein
BTH_II1955	methyl-accepting chemotaxis protein
BTH_II1957	copper resistance protein, putative
BTH_II1960	copper tolerance protein
BTH_II1961	hypothetical protein
BTH_II1962	hypothetical protein
BTH_II1965	hypothetical protein
BTH_II1968	conserved hypothetical protein
BTH_II1971	conserved hypothetical protein
BTH_II1972	hypothetical protein
BTH_II1973	unnamed protein product; Some similarities with probable aminopeptidase
BTH_II1987	hypothetical protein
BTH_II1990	conserved hypothetical protein
BTH_II1991	hypothetical protein
BTH_II1996	unnamed protein product; Similar to bacteriophage integrase
BTH_II2004	Adenylosuccinate synthase
BTH_II2011	hypothetical protein
BTH_II2021	bacterioferritin
BTH_II2028	pirin family protein
BTH_II2042	TnpC protein
BTH_II2060	L-lactate transporter
BTH_II2061	malate synthase G
BTH_II2063	transcriptional regulator, AraC family
BTH_II2073	drug resistance transporter, EmrB/QacA family protein
BTH_II2075	conserved hypothetical protein
BTH_II2076	hypothetical protein
BTH_II2081	transcriptional regulator, GntR family
BTH_II2084	hypothetical protein
BTH_II2086	D-methionine ABC transporter, periplasmic D-methionine-binding protein
BTH_II2087	autoinducer-binding transcriptional regulator, LuxR family
BTH_II2088	thiotemplate mechanism natural product synthetase
BTH_II2090	syringomycin synthesis regulator SyrP, putative
BTH_II2093	polyketide synthase, putative
BTH_II2095	diaminopimelate decarboxylase, putative

BTH_II2101	acetyltransferase, GNAT family
BTH_II2109	transposase, putative
BTH_II2110	Transposase (IS4 family)
BTH_II2113	hypothetical protein
BTH_II2114	putative 4-oxalocrotonate tautomerase
BTH_II2115	transcriptional regulator, LysR family
BTH_II2124	O-methyltransferase family protein
BTH_II2126	beta-ketoadipyl CoA thiolase
BTH_II2128	transcriptional regulator, TetR family
BTH_II2129	major facilitator family transporter
BTH_II2132	O-antigen acetylase, putative
BTH_II2136	conserved hypothetical protein
BTH_II2138	conserved hypothetical protein
BTH_II2142	hemin ABC transporter, permease protein
BTH_II2147	L-allo-threonine aldolase
BTH_II2152	oxidoreductase, FMN-binding
BTH_II2153	oxidoreductase, aldo/keto reductase family
BTH_II2154	major facilitator family transporter
BTH_II2155	probable LysR-family transcriptional regulator
BTH_II2157	transcriptional regulator
BTH_II2158	NAD(P)H nitroreductase BH2236
BTH_II2159	lipH
BTH_II2160	transcriptional regulator, LysR family
BTH_II2161	choline dehydrogenase
BTH_II2162	aldehyde dehydrogenase (NAD) family protein superfamily
BTH_II2163	Amidohydrolase family superfamily
BTH_II2165	Domain of Unknown Function (DUF748) family
BTH_II2167	conserved hypothetical protein
BTH_II2171	hypothetical protein
BTH_II2173	hypothetical protein
BTH_II2174	hypothetical protein
BTH_II2175	hypothetical protein
BTH_II2182	conserved hypothetical protein
BTH_II2184	conserved hypothetical protein
BTH_II2185	uncharacterized domain 1 protein
BTH_II2191	methyl-accepting chemotaxis protein
BTH_II2192	conserved hypothetical protein
BTH_II2193	proline iminopeptidase
BTH_II2194	conserved hypothetical protein
BTH_II2195	glyoxalase family protein
BTH_II2197	transcriptional regulator, AraC family
BTH_II2198	conserved membrane-associated protein
BTH_II2199	conserved hypothetical protein
BTH_II2200	fumarylacetoacetate hydrolase family protein
BTH_II2201	transcriptional regulator, IclR family
BTH_II2202	transposase, degenerate
BTH_II2203	transposase, degenerate
BTH_II2209	hypothetical protein
BTH_II2222	conserved hypothetical protein

BTH_II2246	ISBm1, transposase orfB, interruption-C
BTH_II2247	transcriptional regulator, LysR family
BTH_II2248	conserved hypothetical protein
BTH_II2249	glutamate-1-semialdehyde aminotransferase
BTH_II2250	conserved hypothetical protein
BTH_II2277	transposase, putative
BTH_II2278	Transposase (IS4 family)
BTH_II2281	translation initiation inhibitor
BTH_II2284	transcriptional regulator, GntR family
BTH_II2288	conserved hypothetical protein
BTH_II2289	conserved hypothetical protein
BTH_II2291	hypothetical protein
BTH_II2293	phosphatidylserine decarboxylase, putative
BTH_II2297	membrane protein, putative
BTH_II2299	conserved hypothetical protein
BTH_II2300	conserved hypothetical protein
BTH_II2302	2-oxoisovalerate dehydrogenase, E2 component, dihydrolipamide acetyltransferase
BTH_II2305	conserved hypothetical protein
BTH_II2319	hypothetical protein
BTH_II2320	hypothetical protein
BTH_II2321	stress response protein
BTH_II2322	polysaccharide deacetylase family protein
BTH_II2323	conserved hypothetical protein
BTH_II2326	unnamed protein product; Highly similar to unknown protein of Photorhabdus and some similarities with unknown protein, putative
BTH_II2332	lipoprotein, putative
BTH_II2333	hydrophobe/amphiphile efflux family protein
BTH_II2348	polyketide synthase, putative
BTH_II2360	conserved hypothetical protein
BTH_II2361	phytoene synthase, putative
BTH_II2368	sigma-70 factor, putative
