# Supplementary material for: Bacterial genome adaptation to niches: Divergence of the potential virulence genes in three Burkholderia species of different survival strategies
Source: BMC Genomics. 2005 Dec 7;6:174. doi: 10.1186/1471-2164-6-174 (PMC1343551; doi:10.1186/1471-2164-6-174)
Supplement: Additional File 5 — Genes up- or down-expressed in vivo in Fig. 3B. [file 1471-2164-6-174-S5.pdf]

Table S3. Genes up- or down-expressed *in vivo* in Fig. 3B.

Gene	Description	*in vivo* expression	Divergence group
BMA0011	hypothetical protein	Groups 1&3	1
BMA0024	aldolase, class II	Groups 1&3	1
BMA0030	ElaA family protein	Groups 1&3	1
BMA0040	conserved hypothetical protein	Groups 1&3	1
BMA0101	8-amino-7-oxononanoate synthase	Groups 1&3	1
BMA0156	HpcH/HpaI aldolase family protein	Groups 1&3	1
BMA0305	conserved hypothetical protein, authentic point mutation	Groups 1&3	1
BMA0320	xanthine/uracil permease family protein, truncation	Groups 1&3	1
BMA0328	cation ABC transporter, periplasmic cation-binding protein, putative	Groups 1&3	1
BMA0551	conserved hypothetical protein	Groups 1&3	1
BMA0575	hypothetical protein	Groups 1&3	1
BMA0576	YdjC-like family protein	Groups 1&3	1
BMA0678	isoleucine biosynthesis transcriptional activator	Groups 1&3	1
BMA0687	iron compound ABC transporter, ATP-binding protein	Groups 1&3	1
BMA0697	cobyric acid synthase CobQ	Groups 1&3	1
BMA0824	poly(3-hydroxybutyrate) depolymerase	Groups 1&3	1
BMA0887	di-haem cytochrome c peroxidase family protein	Groups 1&3	1
BMA0906	thioesterase family protein	Groups 1&3	1
BMA0912	iolD protein	Groups 1&3	1
BMA0930	fosmidomycin resistance protein	Groups 1&3	1
BMA0967	hypothetical protein	Groups 1&3	1
BMA0988	cytochrome c family protein	Groups 1&3	1
BMA1044	conserved hypothetical protein	Groups 1&3	1
BMA1058	drug resistance transporter, EmrB/QacA family	Groups 1&3	1
BMA1163	precorrin-2 C20-methyltransferase	Groups 1&3	1
BMA1175	cob(I)alamin adenosyltransferase	Groups 1&3	1
BMA1198	ribose ABC transporter, periplasmic ribose-binding protein	Groups 1&3	1
BMA1239	alkanesulfonate monooxygenase	Groups 1&3	1
BMA1473	outer membrane porin, putative	Groups 1&3	1
BMA1505	allantoicase	Groups 1&3	1
BMA1621	cysteine synthase/cystathionine beta-synthase family protein	Groups 1&3	1
BMA1634	conserved hypothetical protein	Groups 1&3	1
BMA1637	membrane protein, putative	Groups 1&3	1
BMA1638	peptide synthetase-domain protein	Groups 1&3	1
BMA1683	phosphoesterase, putative	Groups 1&3	1
BMA1684	hydrolase, alpha/beta fold family	Groups 1&3	1
BMA1732	nitrate reductase, beta subunit	Groups 1&3	1
BMA1854	Ser/Thr protein phosphatase family protein	Groups 1&3	1
BMA1900	carboxymuconolactone decarboxylase family protein	Groups 1&3	1
BMA1959	MOSC domain protein	Groups 1&3	1
BMA2110	conserved hypothetical protein	Groups 1&3	1
BMA2131	6-phosphogluconolactonase	Groups 1&3	1
BMA2134	amino acid ABC transporter, periplasmic amino acid-binding protein	Groups 1&3	1
BMA2152	LysE family protein	Groups 1&3	1
BMA2177	branched-chain amino acid ABC transporter, permease protein, putative	Groups 1&3	1
BMA2222	phenazine biosynthesis protein phzF, putative	Groups 1&3	1
BMA2282	amino acid permease, truncation	Groups 1&3	1
BMA2589	flavodoxin domain protein	Groups 1&3	1
BMA2679	ABC transporter, ATP-binding protein	Groups 1&3	1
BMA2738	outer membrane protein, OmpW family	Groups 1&3	1

BMA2777	general secretory pathway protein K	Groups 1&3	1
BMA2847	flagellar biosynthetic protein FlhB	Groups 1&3	1
BMA2855	chemotaxis protein CheD	Groups 1&3	1
BMA2860	chemotaxis response regulator	Groups 1&3	1
BMA2908	conserved hypothetical protein	Groups 1&3	1
BMA2978	membrane protein, putative	Groups 1&3	1
BMA3132	molybdopterin oxidoreductase family protein	Groups 1&3	1
BMA3168	transcriptional regulator, GntR family	Groups 1&3	1
BMA3246	homoserine O-acetyltransferase	Groups 1&3	1
BMA3297	oxidoreductase, FAD-binding family protein	Groups 1&3	1
BMA3323	flagella basal body P-ring formation protein FlgA	Groups 1&3	1
BMA3328	flagellar hook protein FlgE	Groups 1&3	1
BMAA0154	sulfotransferase domain protein	Groups 1&3	1
BMAA0180	isochorismatase family protein	Groups 1&3	1
BMAA0188	transcriptional regulator CatR	Groups 1&3	1
BMAA0242	acyl-CoA dehydrogenase domain protein	Groups 1&3	1
BMAA0259	extracellular nuclease, putative	Groups 1&3	1
BMAA0261	conserved hypothetical protein	Groups 1&3	1
BMAA0271	conserved domain protein	Groups 1&3	1
BMAA0280	membrane protein, putative	Groups 1&3	1
BMAA0321	conserved hypothetical protein	Groups 1&3	1
BMAA0363	sigma-54 dependent DNA-binding transcriptional regulator	Groups 1&3	1
BMAA0367	acetyltransferase, GNAT family	Groups 1&3	1
BMAA0369	indole-3-acetamide hydrolase-related protein	Groups 1&3	1
BMAA0394	lipoprotein, putative	Groups 1&3	1
BMAA0407	conserved hypothetical protein	Groups 1&3	1
BMAA0410	Rhs element Vgr protein	Groups 1&3	1
BMAA0449	conserved hypothetical protein	Groups 1&3	1
BMAA0450	conserved hypothetical protein	Groups 1&3	1
BMAA0451	lipoprotein, putative	Groups 1&3	1
BMAA0454	conserved hypothetical protein	Groups 1&3	1
BMAA0462	rhamnosyltransferase II	Groups 1&3	1
BMAA0468	glutathione-independent formaldehyde dehydrogenase	Groups 1&3	1
BMAA0474	oxidoreductase, FAD/FMN-binding	Groups 1&3	1
BMAA0493	conserved hypothetical protein	Groups 1&3	1
BMAA0503	hemK protein	Groups 1&3	1
BMAA0560	arsenical resistance transcriptional regulator	Groups 1&3	1
BMAA0566	transcriptional regulator, AraC family	Groups 1&3	1
BMAA0577	2-hydroxy-3-oxopropionate reductase	Groups 1&3	1
BMAA0589	conserved hypothetical protein	Groups 1&3	1
BMAA0595	fusaric acid resistance domain protein	Groups 1&3	1
BMAA0641	hydrolase	Groups 1&3	1
BMAA0673	hypothetical protein	Groups 1&3	1
BMAA0731	hypothetical protein	Groups 1&3	1
BMAA0753	SCO1/SenC family protein	Groups 1&3	1
BMAA0755	outer membrane nitrite reductase, putative	Groups 1&3	1
BMAA0763	hypothetical protein	Groups 1&3	1
BMAA0776	transcriptional regulator, LysR family	Groups 1&3	1
BMAA0785	sensor histidine kinase	Groups 1&3	1
BMAA0848	4-hydroxyphenylpyruvate dioxygenase, putative	Groups 1&3	1
BMAA0852	aminotransferase, class V	Groups 1&3	1
BMAA0855	ABC transporter, periplasmic substrate-binding protein	Groups 1&3	1
BMAA0922	drug resistance transporter, EmrB/QacA family	Groups 1&3	1

BMAA0923	rhamnosyltransferase family protein	Groups 1&3	1
BMAA1042	conserved hypothetical protein	Groups 1&3	1
BMAA1044	efflux transporter, RND family, MFP subunit	Groups 1&3	1
BMAA1045	hydrophobe/amphiphile efflux family protein	Groups 1&3	1
BMAA1062	conserved hypothetical protein	Groups 1&3	1
BMAA1091	regulatory protein NasS, putative	Groups 1&3	1
BMAA1098	acetylpolyamine aminohydrolase, putative	Groups 1&3	1
BMAA1104	oxidoreductase, short chain dehydrogenase/reductase family	Groups 1&3	1
BMAA1128	ABC transporter, periplasmic substrate-binding protein	Groups 1&3	1
BMAA1187	RNA polymerase sigma-70 factor, ECF subfamily	Groups 1&3	1
BMAA1190	fumarylacetoacetate hydrolase family protein	Groups 1&3	1
BMAA1248	serine metalloprotease MrpA	Groups 1&3	1
BMAA1323	outer membrane lipoprotein, OmpA/SmpA/OmlA family	Groups 1&3	1
BMAA1368	conserved hypothetical protein	Groups 1&3	1
BMAA1372	conserved hypothetical protein	Groups 1&3	1
BMAA1384	hypothetical protein	Groups 1&3	1
BMAA1397	conserved hypothetical protein	Groups 1&3	1
BMAA1419	proline racemase, putative	Groups 1&3	1
BMAA1423	malate/L-lactate dehydrogenase family protein	Groups 1&3	1
BMAA1428	lactate permease family protein	Groups 1&3	1
BMAA1517	transcriptional regulator, araC family	Groups 1&3	1
BMAA1520	type III secretion chaperone BicP	Groups 1&3	1
BMAA1524	BapC protein	Groups 1&3	1
BMAA1540	type III secretion system protein BsaS	Groups 1&3	1
BMAA1621	regulatory protein HrpB	Groups 1&3	1
BMAA1622	conserved hypothetical protein	Groups 1&3	1
BMAA1624	conserved hypothetical protein	Groups 1&3	1
BMAA1660	sensory box sensor histidine kinase	Groups 1&3	1
BMAA1683	formate dehydrogenase, alpha subunit, selenocysteine-containing, authentic point mutation	Groups 1&3	1
BMAA1801	TonB protein, putative	Groups 1&3	1
BMAA1846	hypothetical protein	Groups 1&3	1
BMAA1871	propionate catabolism operon regulatory protein	Groups 1&3	1
BMAA1920	quinone oxidoreductase, putative	Groups 1&3	1
BMAA1927	conserved domain protein	Groups 1&3	1
BMAA1932	transcriptional regulator, LysR family	Groups 1&3	1
BMAA2000	2-hydroxy-3-oxopropionate reductase	Groups 1&3	1
BMAA2006	flavin reductase domain protein	Groups 1&3	1
BMAA2018	conserved hypothetical protein	Groups 1&3	1
BMAA2031	sulfate permease family protein	Groups 1&3	1
BMAA2082	hypothetical protein	Groups 1&3	1
BMA0018	hypothetical protein	Groups 1&3	1
BMA0455	glutamine amidotransferase, class I	Groups 1&3	1
BMA0656	glutamine synthetase family protein	Groups 1&3	1
BMA1051	2-oxoglutarate dehydrogenase, E2 component, dihydrolipoamide succinyltransferase	Groups 1&3	1
BMA1144	arginine/ornithine antiporter	Groups 1&3	1
BMA1148	oxidoreductase, short-chain dehydrogenase/reductase family	Groups 1&3	1
BMA1190	mbtH-like protein	Groups 1&3	1
BMA1191	RNA polymerase sigma-70 factor, ECF subfamily	Groups 1&3	1
BMA1205	cys regulon transcriptional activator	Groups 1&3	1
BMA1304	outer membrane protein, OmpW family	Groups 1&3	1
BMA2323	3-methyl-2-oxobutanoate hydroxymethyltransferase	Groups 1&3	1
BMA2726	ABC transporter, ATP-binding protein	Groups 1&3	1
BMA2751	hypothetical protein	Groups 1&3	1

BMA3176	cyd operon protein YbgT	Groups 1&3	1
BMA3179	hypothetical protein	Groups 1&3	1
BMAA0011	tartrate dehydrogenase	Groups 1&3	1
BMAA0089	membrane protein, putative	Groups 1&3	1
BMAA0198	muconolactone delta-isomerase	Groups 1&3	1
BMAA0277	hypothetical protein	Groups 1&3	1
BMAA0638	sugar ABC transporter, ATP-binding protein	Groups 1&3	1
BMAA0708	conserved hypothetical protein	Groups 1&3	1
BMAA0732	conserved hypothetical protein	Groups 1&3	1
BMAA1145	sensor histidine kinase	Groups 1&3	1
BMAA1431	cysteine-rich domain protein	Groups 1&3	1
BMAA1511	conserved hypothetical protein	Groups 1&3	1
BMAA1529	DNA-binding protein BprA	Groups 1&3	1
BMAA1547	type III secretion system protein BsaM	Groups 1&3	1
BMAA1548	type III secretion system protein BsaL	Groups 1&3	1
BMAA1549	type III secretion system protein BsaK	Groups 1&3	1
BMAA1662	response regulator	Groups 1&3	1
BMAA1785	chitin binding domain protein	Groups 1&3	1
BMAA1828	hemin ABC transporter, periplasmic hemin-binding protein	Groups 1&3	1
BMAA2034	conserved hypothetical protein	Groups 1&3	1
BMA0014	hypothetical protein	Groups 1&3	2
BMA0224	hypothetical protein	Groups 1&3	2
BMA0258	hypothetical protein	Groups 1&3	2
BMA0357	conserved domain protein	Groups 1&3	2
BMA0952	RND efflux system, outer membrane lipoprotein, NodT family	Groups 1&3	2
BMA0985	hypothetical protein	Groups 1&3	2
BMA0989	hypothetical protein	Groups 1&3	2
BMA1038	penicillin amidase, putative	Groups 1&3	2
BMA1113	conserved hypothetical protein	Groups 1&3	2
BMA1123	peptide synthetase, putative	Groups 1&3	2
BMA1639	methyltransferase, putative/adenylsulfate kinase	Groups 1&3	2
BMA1754	CoA transferase, CAIB/BAIF family	Groups 1&3	2
BMA1944	conserved hypothetical protein	Groups 1&3	2
BMA2157	conserved hypothetical protein	Groups 1&3	2
BMA2247	hypothetical protein	Groups 1&3	2
BMA2576	phenylacetic acid degradation protein PaaD	Groups 1&3	2
BMA2880	3-oxoacyl-(acyl-carrier-protein) synthase III, putative	Groups 1&3	2
BMA3058	carotenoid 9,10-9',10' cleavage dioxygenase, putative	Groups 1&3	2
BMA3068	hypothetical protein	Groups 1&3	2
BMA3158	AMP-binding domain protein	Groups 1&3	2
BMA3224	conserved hypothetical protein	Groups 1&3	2
BMAA0059	conserved hypothetical protein	Groups 1&3	2
BMAA0073	hypothetical protein	Groups 1&3	2
BMAA0098	hypothetical protein	Groups 1&3	2
BMAA0372	hypothetical protein	Groups 1&3	2
BMAA0522	hypothetical protein	Groups 1&3	2
BMAA0594	hypothetical protein	Groups 1&3	2
BMAA0616	hypothetical protein	Groups 1&3	2
BMAA0618	hypothetical protein	Groups 1&3	2
BMAA0619	transcriptional regulator, MarR family	Groups 1&3	2
BMAA0628	hypothetical protein	Groups 1&3	2
BMAA0630	aldehyde dehydrogenase (NADP) family protein	Groups 1&3	2
BMAA0646	hypothetical protein	Groups 1&3	2

BMAA0651	H-NS histone family protein	Groups 1&3	2
BMAA0664	EAL/GGDEF domain protein	Groups 1&3	2
BMAA0689	hypothetical protein	Groups 1&3	2
BMAA0752	hypothetical protein	Groups 1&3	2
BMAA0798	mulitcopper oxidase domain protein	Groups 1&3	2
BMAA0841	conserved hypothetical protein, degenerate	Groups 1&3	2
BMAA0955	hypothetical protein	Groups 1&3	2
BMAA0957	conserved hypothetical protein	Groups 1&3	2
BMAA1011	hypothetical protein	Groups 1&3	2
BMAA1032	hypothetical protein	Groups 1&3	2
BMAA1133	transcriptional regulator, AraC family	Groups 1&3	2
BMAA1149	hypothetical protein	Groups 1&3	2
BMAA1151	conserved hypothetical protein	Groups 1&3	2
BMAA1162	hypothetical protein	Groups 1&3	2
BMAA1164	membrane protein, putative	Groups 1&3	2
BMAA1184	conserved hypothetical protein	Groups 1&3	2
BMAA1202	polyketide synthase, putative, degenerate	Groups 1&3	2
BMAA1211	hypothetical protein	Groups 1&3	2
BMAA1252	hypothetical protein	Groups 1&3	2
BMAA1390	conserved hypothetical protein	Groups 1&3	2
BMAA1434	hypothetical protein	Groups 1&3	2
BMAA1486	O-methyltransferase family protein	Groups 1&3	2
BMAA1498	O-antigen acetylase, putative	Groups 1&3	2
BMAA1568	serine protease, kumamolysin	Groups 1&3	2
BMAA1617	hrp protein, putative	Groups 1&3	2
BMAA1619	hypothetical protein	Groups 1&3	2
BMAA1690	luciferase-like monooxygenase	Groups 1&3	2
BMAA1757	hemolysin activator protein, HlyB family	Groups 1&3	2
BMAA1841	glyoxalase family protein	Groups 1&3	2
BMAA1842	hypothetical protein	Groups 1&3	2
BMAA1865	conserved hypothetical protein	Groups 1&3	2
BMAA1875	hypothetical protein	Groups 1&3	2
BMAA1895	conserved domain protein	Groups 1&3	2
BMAA1902	conserved hypothetical protein	Groups 1&3	2
BMAA1912	conserved hypothetical protein	Groups 1&3	2
BMAA1925	hypothetical protein	Groups 1&3	2
BMAA1970	conserved hypothetical protein	Groups 1&3	2
BMAA1973	conserved hypothetical protein	Groups 1&3	2
BMAA1983	hypothetical protein	Groups 1&3	2
BMAA1986	ADP-heptose--LPS heptosyltransferase II, putative	Groups 1&3	2
BMAA1987	glycosyl transferase, group 2 family protein	Groups 1&3	2
BMAA1995	conserved domain protein	Groups 1&3	2
BMAA2016	hypothetical protein	Groups 1&3	2
BMAA2045	major facilitator family transporter	Groups 1&3	2
BMAA2047	molybdopterin oxidoreductase family protein	Groups 1&3	2
BMA3052	nitrite/sulfite reductase family protein	Groups 1&3	2
BMAA0023	cytochrome P450-related protein	Groups 1&3	2
BMAA0703	DNA-binding response regulator, LuxR family	Groups 1&3	2
BMAA0728	hypothetical protein	Groups 1&3	2
BMAA0751	N-acetylmuramoyl-L-alanine amidase domain protein	Groups 1&3	2
BMAA1395	cyclic nucleotide-binding domain protein	Groups 1&3	2
BMAA1488	hypothetical protein	Groups 1&3	2
BMAA2014	hypothetical protein	Groups 1&3	2

BMA0016	hypothetical protein	Groups 1&3	4
BMA1194	carbohydrate kinase, FGGY family	Groups 1&3	4
BMA0017	hypothetical protein	Groups 1&3	3
BMA0267	conserved domain protein, truncation	Groups 1&3	3
BMA0642	conserved hypothetical protein, degenerate	Groups 1&3	3
BMA0702	hypothetical protein	Groups 1&3	3
BMA2007	hypothetical protein	Groups 1&3	3
BMA3012	hypothetical protein	Groups 1&3	3
BMA3300	hypothetical protein	Groups 1&3	3
BMAA0323	hypothetical protein	Groups 1&3	3
BMAA0326	hypothetical protein	Groups 1&3	3
BMAA0386	D-serine dehydratase, authentic frameshift	Groups 1&3	3
BMAA0562	hypothetical protein	Groups 1&3	3
BMAA0597	hypothetical protein	Groups 1&3	3
BMAA0610	di-haem cytochrome c peroxidase family protein	Groups 1&3	3
BMAA0775	hypothetical protein	Groups 1&3	3
BMAA0825	hypothetical protein	Groups 1&3	3
BMAA0838	hypothetical protein	Groups 1&3	3
BMAA0895	hypothetical protein	Groups 1&3	3
BMAA0985	hypothetical protein	Groups 1&3	3
BMAA1109	hypothetical protein	Groups 1&3	3
BMAA1116	conserved hypothetical protein	Groups 1&3	3
BMAA1354	hypothetical protein	Groups 1&3	3
BMAA1383	hypothetical protein	Groups 1&3	3
BMAA1475	porin, degenerate	Groups 1&3	3
BMAA1526	BapA protein	Groups 1&3	3
BMAA1625	type III secretion inner membrane protein, authentic frameshift	Groups 1&3	3
BMAA1887	hypothetical protein	Groups 1&3	3
BMAA1935	hypothetical protein	Groups 1&3	3
BMAA1945	hypothetical protein	Groups 1&3	3
BMA0605	conserved hypothetical protein	Groups 1&3	3
BMA0001	chromosomal replication initiator protein DnaA	Group 2	1
BMA0002	DNA polymerase III, beta subunit	Group 2	1
BMA0083	conserved hypothetical protein	Group 2	1
BMA0084	arginyl-tRNA synthetase	Group 2	1
BMA0094	isocitrate dehydrogenase kinase/phosphatase	Group 2	1
BMA0097	oxidoreductase, short chain dehydrogenase/reductase family	Group 2	1
BMA0188	conserved hypothetical protein	Group 2	1
BMA0189	2-polyprenylphenol 6-hydroxylase	Group 2	1
BMA0192	conserved hypothetical protein	Group 2	1
BMA0213	lactoylglutathione lyase	Group 2	1
BMA0221	magnesium and cobalt efflux protein CorC	Group 2	1
BMA0275	succinyl-CoA synthase, beta subunit	Group 2	1
BMA0292	conserved hypothetical protein	Group 2	1
BMA0310	serine protease	Group 2	1
BMA0315	efflux transporter, RND family, MFP subunit	Group 2	1
BMA0317	RND efflux system, outer membrane lipoprotein, NodT family	Group 2	1
BMA0333	maltose/mannitol ABC transporter, periplasmic maltose/mannitol-binding protein, putative	Group 2	1
BMA0335	maltose/mannitol ABC transporter, permease protein, putative	Group 2	1
BMA0337	maltose/mannitol ABC transporter, ATP-binding protein, putative	Group 2	1
BMA0351	tryptophan 2,3-dioxygenase family protein	Group 2	1
BMA0366	conserved hypothetical protein TIGR00150	Group 2	1
BMA0371	ebsC protein, putative	Group 2	1

BMA0373	conserved hypothetical protein	Group 2	1
BMA0375	ornithine carbamoyltransferase	Group 2	1
BMA0377	ribosomal protein S20	Group 2	1
BMA0390	conserved hypothetical protein	Group 2	1
BMA0400	ribosomal protein L19	Group 2	1
BMA0403	ribosomal protein S16	Group 2	1
BMA0428	ribosomal protein S1	Group 2	1
BMA0429	cytidylate kinase	Group 2	1
BMA0433	phosphoserine aminotransferase	Group 2	1
BMA0435	DNA gyrase, A subunit	Group 2	1
BMA0436	OmpA family protein	Group 2	1
BMA0467	5-methyltetrahydropteroyltriglutamate--homocysteine S-methyltransferase	Group 2	1
BMA0469	fructose-1,6-bisphosphatase	Group 2	1
BMA0486	isocitrate dehydrogenase, NADP-dependent	Group 2	1
BMA0527	conserved hypothetical protein	Group 2	1
BMA0528	ribosomal protein L32	Group 2	1
BMA0533	acyl carrier protein	Group 2	1
BMA0536	RNA polymerase sigma-H factor	Group 2	1
BMA0541	GTP-binding protein LepA	Group 2	1
BMA0546	pyridoxal phosphate biosynthetic protein PdxJ	Group 2	1
BMA0549	sigma-54 dependent DNA-binding response regulator	Group 2	1
BMA0550	translation elongation factor P	Group 2	1
BMA0599	fatty acid desaturase domain protein	Group 2	1
BMA0606	Rrf2 family protein	Group 2	1
BMA0619	cyclic nucleotide-binding domain protein	Group 2	1
BMA0623	peptidase, U32 family	Group 2	1
BMA0625	conserved hypothetical protein	Group 2	1
BMA0626	conserved hypothetical protein	Group 2	1
BMA0672	cytosol aminopeptidase	Group 2	1
BMA0673	DNA polymerase III, chi subunit, putative	Group 2	1
BMA0683	conserved hypothetical protein	Group 2	1
BMA0699	ParA family protein	Group 2	1
BMA0704	conserved hypothetical protein	Group 2	1
BMA0714	deoxycytidine triphosphate deaminase, putative	Group 2	1
BMA0715	Orn/Lys/Arg decarboxylase	Group 2	1
BMA0741	glutamine dependent NAD+ synthetase	Group 2	1
BMA0742	nitrogen regulatory protein P-II	Group 2	1
BMA0743	outer membrane porin, putative	Group 2	1
BMA0752	hypothetical protein	Group 2	1
BMA0770	carbamoyl-phosphate synthase, small subunit	Group 2	1
BMA0771	homoserine/threonine efflux protein, putative	Group 2	1
BMA0776	ribosomal RNA large subunit methyltransferase J	Group 2	1
BMA0797	conserved hypothetical protein	Group 2	1
BMA0798	hypothetical protein	Group 2	1
BMA0864	H-NS histone family protein	Group 2	1
BMA0890	hypothetical protein	Group 2	1
BMA0904	conserved hypothetical protein	Group 2	1
BMA0926	UTP-glucose-1-phosphate uridylyltransferase	Group 2	1
BMA0976	conserved hypothetical protein	Group 2	1
BMA1052	2-oxoglutarate dehydrogenase, E1 component	Group 2	1
BMA1060	ribosome-binding factor A	Group 2	1
BMA1090	integration host factor, alpha subunit	Group 2	1
BMA1094	ribosomal protein L35	Group 2	1

BMA1099	conserved hypothetical protein	Group 2	1
BMA1145	arginine deiminase	Group 2	1
BMA1259	transcriptional regulator, GntR family	Group 2	1
BMA1263	ribonuclease R	Group 2	1
BMA1300	putrescine ABC transporter, ATP-binding protein	Group 2	1
BMA1301	putrescine ABC transporter, periplasmic putrescine-binding protein	Group 2	1
BMA1303	conserved hypothetical protein	Group 2	1
BMA1319	polyhydroxyalkanoate synthesis repressor PhaR	Group 2	1
BMA1325	competence lipoprotein ComL	Group 2	1
BMA1327	conserved hypothetical protein	Group 2	1
BMA1328	lipoprotein, putative	Group 2	1
BMA1333	adenylosuccinate synthetase	Group 2	1
BMA1340	hfq protein	Group 2	1
BMA1348	nucleoside diphosphate kinase	Group 2	1
BMA1355	lipoprotein NlpD, putative	Group 2	1
BMA1364	thioredoxin	Group 2	1
BMA1365	transcription termination factor Rho	Group 2	1
BMA1369	ribosomal protein L31	Group 2	1
BMA1378	Rrf2 family protein	Group 2	1
BMA1380	molybdopterin converting factor, subunit 2	Group 2	1
BMA1395	antioxidant, AhpC/Tsa family	Group 2	1
BMA1402	ribosomal protein S18	Group 2	1
BMA1404	ribosomal protein S6	Group 2	1
BMA1414	phosphate starvation-inducible protein	Group 2	1
BMA1417	glycosyl transferase, group 1 family protein	Group 2	1
BMA1426	lipoprotein, putative	Group 2	1
BMA1433	conserved hypothetical protein	Group 2	1
BMA1435	hypothetical protein	Group 2	1
BMA1440	conserved hypothetical protein	Group 2	1
BMA1453	peptidyl-prolyl cis-trans isomerse D, putative	Group 2	1
BMA1465	ATP-dependent Clp protease, proteolytic subunit ClpP	Group 2	1
BMA1485	DNA-binding response regulator RisA	Group 2	1
BMA1487	antioxidant, AhpC/Tsa family	Group 2	1
BMA1489	2C-methyl-D-erythritol 2,4-cyclodiphosphate synthase	Group 2	1
BMA1498	PspA/IM30 family protein	Group 2	1
BMA1512	ISBma1, transposase	Group 2	1
BMA1522	GMP synthase	Group 2	1
BMA1530	TGS domain protein	Group 2	1
BMA1533	SPFH domain/band 7 family protein	Group 2	1
BMA1545	UDP-3-O-3-hydroxymyristoyl glucosamine N-acyltransferase	Group 2	1
BMA1547	outer membrane protein, OMP85 family	Group 2	1
BMA1554	translation elongation factor Ts	Group 2	1
BMA1555	ribosomal protein S2	Group 2	1
BMA1602	NLP/P60 family protein	Group 2	1
BMA1603	hypothetical protein	Group 2	1
BMA1656	cysteinyl-tRNA synthetase	Group 2	1
BMA1660	UDP-2,3-diacylglucosamine hydrolase	Group 2	1
BMA1690	3-deoxy-8-phosphooctulonate synthase	Group 2	1
BMA1702	conserved hypothetical protein	Group 2	1
BMA1703	ferredoxin, 2Fe-2S	Group 2	1
BMA1708	cysteine desulfurase	Group 2	1
BMA1714	transcriptional regulator, IclR family	Group 2	1
BMA1717	D-alanyl-D-alanine endopeptidase, putative	Group 2	1

BMA1721	pyruvate dehydrogenase, E1 component	Group 2	1
BMA1723	DNA-binding response regulator, LuxR family	Group 2	1
BMA1726	oligopeptidase A	Group 2	1
BMA1736	DNA-binding response regulator NarL	Group 2	1
BMA1743	glutamine synthetase, type I	Group 2	1
BMA1745	molybdopterin-binding protein	Group 2	1
BMA1749	conserved hypothetical protein	Group 2	1
BMA1752	conserved hypothetical protein	Group 2	1
BMA1767	peptidyl-tRNA hydrolase, putative	Group 2	1
BMA1777	iron compound ABC transporter, periplasmic iron-compound-binding protein, putative	Group 2	1
BMA1786	conserved hypothetical protein	Group 2	1
BMA1813	conserved hypothetical protein	Group 2	1
BMA1819	NADH dehydrogenase I, K subunit	Group 2	1
BMA1820	NADH dehydrogenase I, J subunit	Group 2	1
BMA1826	NADH dehydrogenase I, D subunit	Group 2	1
BMA1829	NADH dehydrogenase I, A subunit	Group 2	1
BMA1835	ribosomal protein S15	Group 2	1
BMA1847	acetolactate synthase, small subunit	Group 2	1
BMA1857	transcriptional reguator, TetR family	Group 2	1
BMA1883	uracil phosphoribosyltransferase	Group 2	1
BMA1912	ABC transporter, permease/ATP-binding protein	Group 2	1
BMA1935	cytochrome C4 family protein, authentic frameshift	Group 2	1
BMA1947	heat shock protein HtpG	Group 2	1
BMA1981	O-antigen methyl transferase, putative	Group 2	1
BMA1984	O-antigen acetylase, putative	Group 2	1
BMA1985	polysaccharide ABC transporter, ATP-binding protein	Group 2	1
BMA1988	dTDP-4-dehydrorhamnose 3,5-epimerase	Group 2	1
BMA1996	conserved hypothetical protein TIGR00250	Group 2	1
BMA1999	rubredoxin	Group 2	1
BMA2001	chaperonin, 60 kDa	Group 2	1
BMA2002	chaperonin, 10 kDa	Group 2	1
BMA2005	transcriptional regulator family protein	Group 2	1
BMA2050	D-lactate dehydrogenase	Group 2	1
BMA2058	conserved hypothetical protein	Group 2	1
BMA2066	antioxidant, AhpC/Tsa family	Group 2	1
BMA2068	conserved hypothetical protein	Group 2	1
BMA2074	conserved hypothetical protein TIGR00244	Group 2	1
BMA2075	serine hydroxymethyltransferase	Group 2	1
BMA2078	tolQ protein	Group 2	1
BMA2082	outer membrane protein, OmpA family	Group 2	1
BMA2083	conserved hypothetical protein	Group 2	1
BMA2089	outer membrane porin, putative	Group 2	1
BMA2095	DNA-directed RNA polymerase, omega subunit	Group 2	1
BMA2097	conserved hypothetical protein TIGR00255	Group 2	1
BMA2173	protein-L-isoaspartate O-methyltransferase, putative	Group 2	1
BMA2174	rhodanese-like domain protein	Group 2	1
BMA2181	urease accessory protein UreD	Group 2	1
BMA2229	peptidyl-prolyl cis-trans isomerase, FKBP-type	Group 2	1
BMA2231	ribosomal protein L28	Group 2	1
BMA2232	ribosomal protein L33	Group 2	1
BMA2271	superoxide dismutase	Group 2	1
BMA2277	adenylate kinase	Group 2	1
BMA2279	cold-shock domain family protein	Group 2	1

BMA2280	ATP-dependent Clp protease adaptor protein ClpS	Group 2	1
BMA2281	ATP-dependent Clp protease, ATP-binding subunit ClpA	Group 2	1
BMA2321	2-amino-4-hydroxy-6-hydroxymethyldihydropteridine pyrophosphokinase	Group 2	1
BMA2326	chaperone protein DnaK	Group 2	1
BMA2331	heat-inducible transcription repressor HrcA	Group 2	1
BMA2341	ribosomal protein L13	Group 2	1
BMA2343	iron-sulfur cluster assembly accessory protein	Group 2	1
BMA2380	cytochrome b561, putative	Group 2	1
BMA2382	YceI-like family protein	Group 2	1
BMA2417	flavohemoprotein	Group 2	1
BMA2423	conserved hypothetical protein	Group 2	1
BMA2440	transcriptional regulator, LysR family	Group 2	1
BMA2442	adenylosuccinate lyase	Group 2	1
BMA2446	6-phosphogluconate dehydratase	Group 2	1
BMA2453	leucyl-tRNA synthetase	Group 2	1
BMA2457	outer membrane lipoprotein, putatitve	Group 2	1
BMA2469	transketolase	Group 2	1
BMA2471	glyoxalase family protein	Group 2	1
BMA2474	barstar family protein	Group 2	1
BMA2500	3-dehydroquinate dehydratase, type II	Group 2	1
BMA2509	ribonucleoside-diphosphate reductase, beta subunit	Group 2	1
BMA2518	pyrophosphatase, MutT/nudix family	Group 2	1
BMA2522	ribosomal protein L27	Group 2	1
BMA2523	ribosomal protein L21	Group 2	1
BMA2524	octaprenyl-diphosphate synthase	Group 2	1
BMA2535	conserved hypothetical protein	Group 2	1
BMA2536	conserved hypothetical protein	Group 2	1
BMA2545	cell division protein FtsZ	Group 2	1
BMA2550	UDP-N-acetylmuramate--alanine ligase	Group 2	1
BMA2558	cell division protein FtsL, putative	Group 2	1
BMA2587	orotate phosphoribosyltransferase	Group 2	1
BMA2604	ribosomal protein L17	Group 2	1
BMA2606	dna-directed rna polymerase alpha chain	Group 2	1
BMA2607	ribosomal protein S4	Group 2	1
BMA2609	ribosomal protein S13	Group 2	1
BMA2610	ribosomal protein L36	Group 2	1
BMA2611	translation initiation factor IF-1	Group 2	1
BMA2612	preprotein translocase, SecY subunit	Group 2	1
BMA2616	ribosomal protein L18	Group 2	1
BMA2618	ribosomal protein S8	Group 2	1
BMA2619	ribosomal protein S14	Group 2	1
BMA2620	ribosomal protein L5	Group 2	1
BMA2621	ribosomal protein L24	Group 2	1
BMA2622	ribosomal protein L14	Group 2	1
BMA2623	ribosomal protein S17	Group 2	1
BMA2624	ribosomal protein L29	Group 2	1
BMA2625	ribosomal protein L16	Group 2	1
BMA2627	ribosomal protein L22	Group 2	1
BMA2628	ribosomal protein S19	Group 2	1
BMA2629	ribosomal protein L2	Group 2	1
BMA2630	ribosomal protein L23	Group 2	1
BMA2632	ribosomal protein L3	Group 2	1
BMA2633	ribosomal protein S10	Group 2	1

BMA2634	translation elongation factor Tu	Group 2	1
BMA2635	translation elongation factor G	Group 2	1
BMA2637	ribosomal protein S12	Group 2	1
BMA2640	DNA-directed RNA polymerase, beta subunit	Group 2	1
BMA2642	ribosomal protein L7/L12	Group 2	1
BMA2643	ribosomal protein L10	Group 2	1
BMA2647	preprotein translocase, SecE subunit	Group 2	1
BMA2649	translation elongation factor Tu	Group 2	1
BMA2695	stringent starvation protein A	Group 2	1
BMA2696	ubiquinol-cytochrome c reductase, cytochrome c1	Group 2	1
BMA2703	twin-arginine translocation protein, TatA/E family	Group 2	1
BMA2705	membrane protein, putative	Group 2	1
BMA2706	phosphoribosyl-ATP pyrophosphohydrolase	Group 2	1
BMA2707	phosphoribosyl-AMP cyclohydrolase	Group 2	1
BMA2709	phosphoribosylformimino-5-aminoimidazole carboxamide ribotide isomerase	Group 2	1
BMA2712	imidazoleglycerol-phosphate dehydratase	Group 2	1
BMA2717	BolA/YrbA family protein	Group 2	1
BMA2722	conserved hypothetical protein	Group 2	1
BMA2728	thiamin biosynthesis ThiG	Group 2	1
BMA2754	conserved hypothetical protein	Group 2	1
BMA2787	ipgF protein, putative	Group 2	1
BMA2791	cold-shock domain family protein	Group 2	1
BMA2840	5,10-methylenetetrahydrofolate reductase	Group 2	1
BMA2841	membrane protein, putative	Group 2	1
BMA2842	adenosylhomocysteinase	Group 2	1
BMA2872	ribosomal protein S21	Group 2	1
BMA2905	H-NS histone family protein	Group 2	1
BMA2913	DNA-binding protein HU, form B	Group 2	1
BMA2917	conserved hypothetical protein	Group 2	1
BMA2918	transcriptional regulator, MarR family	Group 2	1
BMA2926	pterin-4-alpha-carbinolamine dehydratase, putative	Group 2	1
BMA2927	phenylalanine-4-hydroxylase	Group 2	1
BMA2944	glucose-inhibited division protein A	Group 2	1
BMA2946	sporulation initiation inhibitor protein Soj	Group 2	1
BMA2952	ATP synthase F0, C subunit	Group 2	1
BMA2953	ATP synthase F0, B subunit	Group 2	1
BMA2955	ATP synthase F1, alpha subunit	Group 2	1
BMA2956	ATP synthase F1, gamma subunit	Group 2	1
BMA2957	ATP synthase F1, beta subunit	Group 2	1
BMA2958	ATP synthase F1, epsilon subunit	Group 2	1
BMA2975	transcriptional regulator, merR family	Group 2	1
BMA2994	glycine cleavage system H protein	Group 2	1
BMA3090	thiamine biosynthesis protein ThiC	Group 2	1
BMA3109	ribosomal subunit interface protein	Group 2	1
BMA3113	ATP-dependent protease La domain protein	Group 2	1
BMA3120	ribose-phosphate pyrophosphokinase	Group 2	1
BMA3124	ferredoxin	Group 2	1
BMA3125	pantetheine-phosphate adenylyltransferase	Group 2	1
BMA3138	RNA polymerase sigma-32 factor	Group 2	1
BMA3178	cytochrome d ubiquinol oxidase, subunit I	Group 2	1
BMA3195	cytochrome c oxidase assembly protein ctaG, putative	Group 2	1
BMA3205	protein-export protein SecB	Group 2	1
BMA3207	rhodanese-like domain protein	Group 2	1

BMA3210	HesA/MoeB/ThiF family protein	Group 2	1
BMA3211	phosphoenolpyruvate-protein phosphotransferase	Group 2	1
BMA3212	phosphocarrier protein HPr	Group 2	1
BMA3213	PTS system, fructose-specific IIA component	Group 2	1
BMA3219	conserved hypothetical protein	Group 2	1
BMA3251	sensor histidine kinase	Group 2	1
BMA3252	response regulator	Group 2	1
BMA3255	DnaK suppressor protein	Group 2	1
BMA3263	hypothetical protein	Group 2	1
BMA3292	ferredoxin--NADP reductase	Group 2	1
BMA3333	flagellar protein FlgJ	Group 2	1
BMA3341	hypothetical protein	Group 2	1
BMA3342	alkylphosphonate utilization operon protein PhnA, putative	Group 2	1
BMA3370	LemA family protein	Group 2	1
BMA3379	glucosamine--fructose-6-phosphate aminotransferase, isomerizing	Group 2	1
BMA3381	conserved hypothetical protein	Group 2	1
BMA3396	hypothetical protein	Group 2	1
BMA3400	ribosomal protein L34	Group 2	1
BMAA0002	phage integrase family protein	Group 2	1
BMAA0017	D-beta-hydroxybutyrate dehydrogenase	Group 2	1
BMAA0018	acetoacetate decarboxylase	Group 2	1
BMAA0024	hypothetical protein	Group 2	1
BMAA0031	transcriptional regulator, Crp/Fnr family	Group 2	1
BMAA0040	hypothetical protein	Group 2	1
BMAA0195	ubiquinol oxidase, subunit I	Group 2	1
BMAA0256	conserved hypothetical protein	Group 2	1
BMAA0292	tautomerase enzyme family protein	Group 2	1
BMAA0302	hypothetical protein	Group 2	1
BMAA0303	organic hydroperoxide resistance protein	Group 2	1
BMAA0309	AMP nucleosidase	Group 2	1
BMAA0318	decarboxylase family protein	Group 2	1
BMAA0329	geranyltranstransferase	Group 2	1
BMAA0339	RNA polymerase sigma factor RpoD	Group 2	1
BMAA0365	H-NS histone family protein	Group 2	1
BMAA0416	hypothetical protein	Group 2	1
BMAA0422	conserved hypothetical protein	Group 2	1
BMAA0424	membrane protein, putative	Group 2	1
BMAA0445	Rhs element Vgr protein	Group 2	1
BMAA0505	glutamyl-tRNA reductase	Group 2	1
BMAA0519	GTP-binding protein YchF	Group 2	1
BMAA0525	thiol:disulfide interchange protein DsbC, putative	Group 2	1
BMAA0660	amino acid ABC transporter, periplasmic amino acid-binding protein, putative	Group 2	1
BMAA0699	hypothetical protein	Group 2	1
BMAA0719	DNA-binding protein	Group 2	1
BMAA0773	transcriptional regulator, TetR family, putative	Group 2	1
BMAA0802	isovaleryl-CoA dehydrogenase	Group 2	1
BMAA0875	oxidoreductase, aldo/keto reductase family	Group 2	1
BMAA0883	iron permease, FTR1 family	Group 2	1
BMAA0896	ribosomal protein S21	Group 2	1
BMAA0899	cold-shock domain family protein	Group 2	1
BMAA0937	transcriptional regulator, ArsR family	Group 2	1
BMAA0943	conserved hypothetical protein	Group 2	1
BMAA0996	gamma-glutamyltranspeptidase	Group 2	1

BMAA0998	hypothetical protein	Group 2	1
BMAA1046	RND efflux system, outer membrane lipoprotein, NodT family	Group 2	1
BMAA1141	homoprotocatechuate degradative operon repressor	Group 2	1
BMAA1232	hypothetical protein	Group 2	1
BMAA1261	bacterial extracellular solute-binding protein, family 5	Group 2	1
BMAA1286	outer membrane porin, putative	Group 2	1
BMAA1350	CBS domain protein	Group 2	1
BMAA1353	outer membrane porin OpcP	Group 2	1
BMAA1370	oxidoreductase, aldo/keto reductase family	Group 2	1
BMAA1462	transcriptional regulator, GntR family	Group 2	1
BMAA1496	amino acid ABC transporter, periplasmic amino acid-binding protein	Group 2	1
BMAA1723	tRNA pseudouridine synthase A	Group 2	1
BMAA1744	citrate synthase I	Group 2	1
BMAA1745	conserved hypothetical protein	Group 2	1
BMAA1749	succinate dehydrogenase, cytochrome b556 subunit	Group 2	1
BMAA1781	conserved hypothetical protein	Group 2	1
BMAA1794	acetyl-coenzyme A synthetase	Group 2	1
BMAA1797	hydro-lyase, Fe-S type, tartrate/fumarate family	Group 2	1
BMAA1798	bacterioferritin	Group 2	1
BMAA1963	radical SAM domain protein	Group 2	1
BMAA2035	stress response protein	Group 2	1
BMAA2098	glutamine ABC transporter, periplasmic glutamine-binding protein	Group 2	1
BMAA2110	NADPH-dependent FMN reductase domain protein	Group 2	1
BMAA2111	sigma-70 factor, putative	Group 2	1
BMAA2113	arsenate reductase	Group 2	1
BMA0256	conserved hypothetical protein	Group 2	2
BMA0376	conserved hypothetical protein	Group 2	2
BMA1120	histidinol-phosphate aminotransferase, putative, authentic point mutation	Group 2	2
BMA1818	NADH dehydrogenase I, L subunit	Group 2	2
BMA2202	translation initiation factor IF-1	Group 2	2
BMA2298	capsular polysaccharide biosynthesis protein, putative	Group 2	2
BMA2302	capsular polysaccharide biosynthesis protein, putative	Group 2	2
BMA2303	glycosyl transferase, group 1 family protein	Group 2	2
BMA2306	capsular polysaccharide export inner-membrane protein	Group 2	2
BMA2544	AhpC/TSA family protein	Group 2	2
BMA2689	conserved hypothetical protein	Group 2	2
BMA2690	conserved hypothetical protein	Group 2	2
BMA2884	hypothetical protein	Group 2	2
BMA3088	conserved hypothetical protein	Group 2	2
BMAA0064	conserved hypothetical protein	Group 2	2
BMAA0162	hypothetical protein	Group 2	2
BMAA0234	hypothetical protein	Group 2	2
BMAA0237	hypothetical protein	Group 2	2
BMAA0238	hypothetical protein	Group 2	2
BMAA0361	hypothetical protein	Group 2	2
BMAA0537	hypothetical protein	Group 2	2
BMAA0548	hypothetical protein	Group 2	2
BMAA0727	GTP cyclohydrolase I	Group 2	2
BMAA0853	hypothetical protein	Group 2	2
BMAA0866	hypothetical protein	Group 2	2
BMAA0873	hypothetical protein	Group 2	2
BMAA1092	GTP cyclohydrolase I	Group 2	2
BMAA1315	hypothetical protein	Group 2	2

BMAA1318	hypothetical protein	Group 2	2
BMAA1490	beta-ketoadipyl CoA thiolase	Group 2	2
BMAA1507	hypothetical protein	Group 2	2
BMAA1514	hypothetical protein	Group 2	2
BMAA1515	hypothetical protein	Group 2	2
BMAA1546	hypothetical protein	Group 2	2
BMAA1555	hypothetical protein	Group 2	2
BMAA1575	hypothetical protein	Group 2	2
BMAA1646	peptide synthetase, putative	Group 2	2
BMAA1805	hypothetical protein	Group 2	2
BMAA1984	sigma-54 dependent transcriptional regulator, authentic point mutation	Group 2	2
BMA0497	site-specific recombinase, phage integrase family, truncation	Group 2	3
BMA0792	hypothetical protein	Group 2	3
BMA1021	type-1 fimbrial protein, authentic frameshift	Group 2	3
BMA1037	hypothetical protein	Group 2	3
BMA1117	transposase, IS3 family, truncation	Group 2	3
BMA1649	hypothetical protein	Group 2	3
BMA1673	peptidyl-prolyl cis-trans isomerase, FKBP-type, authentic frameshift	Group 2	3
BMA1787	transcriptional regulator, putative	Group 2	3
BMA1843	2-isopropylmalate synthase, authentic frameshift	Group 2	3
BMA3005	site-specific recombinase, phage integrase family	Group 2	3
BMA3009	lipoprotein, putative	Group 2	3
BMAA0038	hypothetical protein	Group 2	3
BMAA0081	conserved domain protein	Group 2	3
BMAA0871	hypothetical protein	Group 2	3
BMAA1189	transcriptional regulator, AraC family	Group 2	3
BMAA1592	hipB domain protein	Group 2	3
BMAA1790	conserved hypothetical protein, truncation	Group 2	3
BMAA1838	conserved hypothetical protein	Group 2	3
