# Supplementary material for: Bacterial genome adaptation to niches: Divergence of the potential virulence genes in three Burkholderia species of different survival strategies
Source: BMC Genomics. 2005 Dec 7;6:174. doi: 10.1186/1471-2164-6-174 (PMC1343551; doi:10.1186/1471-2164-6-174)
Supplement: Additional File 6 — The 30 in vivo-expressed genes that are degenerated or unique to Bm in Fig. 3B. [file 1471-2164-6-174-S6.pdf]

Sup_Table_4. The 30 in vivo-expressed genes that are degenerated or unique to Bm in Fig. 3B.

Gene	Description	location	non-SSR	SSR	Truncation	Point mutation	in frame	Frame shift
BMA0017	hypothetical protein	5'-end	G insertion					Yes
BMA0267	conserved domain protein, truncation	3'-end			Yes			Yes
BMA0605	conserved hypothetical protein (Ralstonia solanacearum hemerythrin-like protein)	5'-end		$(ACCCGAGCTGAA)_2$ insertion			Yes	
BMA0642	conserved hypothetical protein, degenerate	5'-end	A deletion					Yes
BMA1561	hypothetical protein	middle		GC				Yes
BMA0702	hypothetical protein	5'-end, middle		5 different kinds				Yes
BMA2007	hypothetical protein	5'-end	54 bp deletion					Yes
BMA3012	hypothetical protein	5'-end	C insertion	GGAGACGCATC deletion				Yes
BMA3300	hypothetical protein	middle		GCCGAC			Yes	
BMAA0323	hypothetical protein	middle, 3'-end				3	Yes	
BMAA0326	hypothetical protein	middle				1	Yes	
BMAA0386	D-serine dehydratase, authentic frameshift	middlle	A deletion			1		Yes
BMAA0562	hypothetical protein	5'-end		CGCACCG insertion				Yes
BMAA0597	hypothetical protein	middle	G insertion	$(CGCCGTATGT)_2$ deletion				Yes
BMAA0610	di-haem cytochrome c peroxidase family protein				entire gene			
BMAA0775	hypothetical protein	middle, 3'-end		GCGCTCGCCCCCGAC deletion, GGCGCC deletion				Yes- but only between the two
BMAA0825	hypothetical protein	middle		GC deletion				Yes
BMAA0838	hypothetical protein	middle		CGGCG deletion				Yes
BMAA0895	hypothetical protein	middle		C deletion in $(C)_4$				Yes
BMAA0985	hypothetical protein	5', 3', middle		many of them				Yes
BMAA1109	hypothetical protein	middle		C deletion in $(C)_7$				Yes
BMAA1116	conserved hypothetical protein	middle, 3'-end		$(GCGCCT)_{10}$ deletion, CGG deletion			Yes	
BMAA1354	hypothetical protein	3'-end		A inserion in $(A)_2$				Yes
BMAA1383	hypothetical protein	3'-end		C inserion to make $(C)_2$				Yes
BMAA1475	porin, degenerate	3'-end	A insertion					Yes
BMAA1526	BapA protein	middle		$(CATCAGGCGTCA)_2$ insertion			Yes	
BMAA1625	type III secretion inner membrane protein, authentic frameshift	middle		GCGGC deletion				Yes
BMAA1887	hypothetical protein	5'-end		GTCCTCGAT deletion			Yes	
BMAA1935	hypothetical protein	5'-end		$(CGGCTT)_4$ insertion			Yes	
BMAA1945	hypothetical protein	5'-end, middle		many of them				Yes
